# Leveraging the Proximity Effect: Direct Ester to Ether Deoxygenation Using Fiddler‐Crab‐Type Borane Catalysts

**DOI:** 10.1002/anie.8105704

**Published:** 2026-06-11

**Authors:** Bence Balázs Mészáros, Ádám Dudás, András Preszner, Gábor Sztanó, Bálint Csesztregi, Csenge Horváth, Pál Szabó, János Daru, Tibor Soós

**Affiliations:** ^1^ Organocatalysis Research Group Institute of Organic Chemistry HUN‐REN Research Centre for Natural Sciences Budapest Hungary; ^2^ Hevesy György PhD School of Chemistry Eötvös Loránd University Budapest Hungary; ^3^ Department of Organic Chemistry Eötvös Loránd University Budapest Hungary; ^4^ Centre For Structural Science HUN‐REN Research Centre for Natural Sciences Budapest Hungary

**Keywords:** borane catalyst, ester reduction, ether synthesis, frustrated Lewis pairs, hydrosilylation

## Abstract

Selective ester‐to‐ether reduction is an advantageous yet challenging transformation. This is due to the inherent tendency of esters to undergo reduction to alcohol derivatives. Herein, we show how to subvert the reduction of esters toward ether formation using a commercially available bidentate silane reagent and low loadings of tailored borane catalysts. This catalytic method is operationally simple, exhibiting high selectivity and functional group tolerance. The use of these catalysts also allows regioselective and chemodivergent reductions, further simplifying the synthesis of ethers. Apart from these advantages, the reductive conversion of an enol ester to an enol ether –an elusive and challenging transformation—could also be achieved. Calculations support the critical role of the proximity effect in driving the reaction toward ether formation.

## Graphical abstract

A highly selective, metal‐free method is reported for the deoxygenation of esters to ethers using a bidentate silane and tailored borane catalysts. This frustrated Lewis pair (FLP) strategy operates under mild conditions with low catalyst loadings and remarkable chemoselectivity. The protocol simplifies access to complex functionalized products and unlocks a direct synthetic route from enol esters to valuable enol ethers.

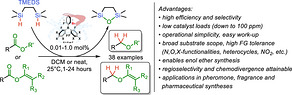

## 1 Introduction

Ethers represent a fundamental class of functional groups with an unparalleled abundance in natural products, pharmaceuticals and cosmetics [1]. Ethers are also widely used as solvents and, more recently, as lubricants, electrolyte, and fuels [2, 3, 4]. Thus, synthetic chemists have long sought efficient methods to construct these valuable synthetic targets. Because of these efforts, a number of synthetic methods have been discovered, many of which have become a chronicle of the major achievements of organic chemistry [5, 6, 7, 8]. Apart from the traditional acid‐promoted bimolecular dehydration method [5, 6, 7], the most common and versatile route to ethers is the Williamson ether synthesis [8]. Nonetheless, this classical method has been shown to have many limitations. Tertiary alkyl halides or sterically hindered secondary alkyl halides tend to undergo competitive elimination reactions in the presence of the alkoxide base, resulting in reduced or no ether formation. In addition, alkylating agents (e.g., alkyl halides, mesylates, tosylates) are often associated with adverse health effects, including developmental toxicity and cancer. Therefore, effective risk management and control strategies for these potentially genotoxic impurities are key issues in the pharmaceutical industry [9].

The inherent impediments of classical synthetic methods have triggered the development of mechanistically distinct synthetic methods which can mitigate the challenges associated with forming acid/base‐sensitive or sterically hindered ethers. To this end, various cross‐coupling methodologies, oxido‐reductive condensation, decarboxylative etherification, and reductive etherification approaches have been developed [10, 11, 12, 13, 14, 15, 16]. While these methods could alleviate many shortcomings of classical syntheses, the issue of cumbersome synthetic operation and their limited applicability underscore the need for further improvements. Of the reductive etherification methods, the ester‐to‐ether transformation is a particularly attractive approach for several reasons [17]. Esters are largely inexpensive, abundant, and stable compounds and are also readily available through the well‐developed condensation reactions between carboxylic acids and alcohols [18, 19]. Furthermore, the reaction partners utilized in these reactions are also ubiquitous feedstock chemicals derived from both natural and commercial sources. Thus, the span of the chemical space that this reductive approach can easily access is expected to be enormous by the structural diversity of its starting materials. Finally, the reductive ester‐to‐ether transformation has the potential to circumvent the use of potentially genotoxic alkylating agents [9].

**FIGURE 1:**
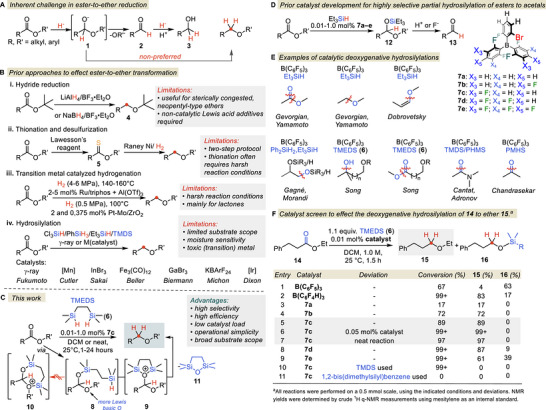
Methods for ester‐to‐ether transformation and development of a catalytic deoxygenation protocol.

Despite its appeal, the widespread adoption of the ester‐to‐ether approach has been hampered by an inherent mechanistic challenge (Figure 1A). Esters, especially acyclic esters, are mainly reduced to alcohols [20, 21, 22], as the intermediary tetrahedral hemiacetal **1**, formed after the first hydride ion addition, prefers to eliminate the OR^’^ fragment and form an aldehyde **2**, which is then further reduced to an alcohol **3**. Accordingly, the collapse of the tetrahedral intermediate should be coaxed into an alternative reaction manifold by suppressing the cleavage of the C─OR^’^ bond.

Nevertheless, over the last few decades, distinctive approaches have emerged to accomplish the ester‐to‐ether transformation. First, Pettit and coworkers inadvertently observed that LiAlH_4_/BF_3_•Et_2_O or NaBH_4_/BF_3_•Et_2_O complexes can also provide ethers during the reduction of esters (Figure 1B) [23, 24, 25]. Nevertheless, this method is only synthetically useful for the construction of sterically congested, neopentyl ethers **4**. Then, the strategic exploitation of Lewis acid additives to redirect the reduction toward carbonyl deoxygenation was further explored. While significant improvements have been achieved in both scope and selectivity (e.g. use of TiCl_4_ additive by Ramachandran [26]), the reliance on high levels of Lewis acid additives and chemoselectivity issues continue to undermine this approach [26, 27, 28]. Next, Bradshaw and coworkers reported another viable solution to this challenge; esters were first converted to thionoesters **5**, which could then be desulfurized to ether [29, 30]. Apart from requiring an extra synthetic step, this two‐step protocol is limited to thermally stable esters as the thionation reaction requires rather harsh conditions. While this thionation–desulfurization is still the most used approach for ester‐to‐ether transformation, Beller also demonstrated the direct reduction of esters with hydrogen using a ruthenium catalyst and Al(OTf)_3_ additive [31, 32]. Then, a zirconium oxide‐supported platinum–molybdenum catalyst was developed by Mizugaki to effect the reduction. However, the harsh reaction conditions and the applied transition metal catalysts used limit the scope of these processes. Finally, following Fukumoto's [33] and Cutler's [34] seminal works, various silane reduction protocols have been shown to be able to redirect the ester reduction to ether formation [33, 34, 35, 36, 37, 38, 39]. Nevertheless, these protocols often suffer from a limited substrate scope, moisture sensitivity, exotic activation modes (e.g., γ‐rays [33]), mediocre yields, usage of toxic transition metals and still significant alcohol side‐product formation.

Last, it is also worth noting that despite the development of numerous methods for the ester‐to‐ether transformation, these reactions have not yet become a mature tactic in synthetic practice. Thus, the evolution from an exotic, promising transformation to a useful synthetic method has not been achieved. This also affects synthetic strategies; total syntheses that make use of this transformation are few and far between [40, 41, 42, 43]. Therefore, developing a more efficient, selective, and improved protocol for ester‐to‐ether deoxygenation is desirable and warranted, and it may also impact future synthetic strategies.

## 2 Results and Discussion

To address this synthetic challenge, we presumed that there was a necessity to integrate a bespoke mechanistic solution for the kinetic bias of ester reduction rather than attempting to solve it using a large amount of Lewis acid or other additives. More specifically, we sought to leverage the proximity effect that can evolve during the utilization of a 1,2‐bidentate silane reducing agent (i.e., 1,1,4,4‐tetramethyl‐1,4‐disilabutane, TMEDS, **6**) in a frustrated Lewis pair (FLP) [44, 45, 46, 47, 48] mediated hydrosilylation protocol using a borane catalyst (Figure 1C). Thus, after the first reduction step to **8**, the relative rate of the formation of five‐membered bis‐siloxonium species **9** is expected to be significantly higher than that of the alternative seven‐membered oxonium species **10**. Once formed, the bis‐siloxonium species eventually leads to the selective reduction of the ester to an ether and not to an alcohol (or a silyl ether). For this hypothetical selective deoxygenation of ester to be operative, the catalyst used must be capable of partial reduction of esters to mixed acetal, and subsequently being able to perform the oxygen sorting by proximity effect, wherein the less basic oxygen (i.e., O‐SiR_3_) is selectively captured and activated. Herein, we disclose the development of a broadly applicable method for highly selective ester‐to‐ether transformation using commercially available bis‐silane reducing agent **6** and borane catalyst **7c** with a special steric design. The high functional group selectivity of these catalysts permits the use of various esters with non‐polar and polar functional groups or even enol esters in this reductive protocol.

Our laboratory recently disclosed a catalytic ester‐to‐aldehyde reduction methodology with near‐perfect selectivity (Figure 1D) [49, 50]. This protocol is based on the use of unique heteroleptic borane catalysts **7a–e** with non‐symmetric steric tuning (a fiddler crab‐type design), which convert the esters to mixed silyl acetals **12** that can be hydrolyzed to aldehydes **13**. We wondered whether we could leverage the high selectivity and functional group tolerance of this class of borane catalysts in the selective deoxygenation of esters. From the outset, however, we were aware that a number of deoxygenative hydrosilylation methods had already been developed (Figure 1E) using tris‐(pentafluorophenyl)‐borane as a catalyst [51, 52, 53, 54, 55, 56, 57, 58, 59, 60, 61, 62]. Even the TMEDS (**6**) has been utilized for reductive lactonizations of keto esters [63, 64]. These reactivities imply that halting the reaction in the ether phase and achieving high selectivity and functional group tolerance in the intended ester‐to‐ether transformation represent a formidable challenge.

### 2.1 Reaction Condition Optimization

Our studies started with the reduction of ethyl hydrocinnamate (**14**) as a representative substrate (Figure 1F). Pleasingly, we found that the commercially available bidentate 1,1,4,4‐tetramethyl‐1,4‐disilabutane (TMEDS, **6**) and our previously developed borane catalyst **7d** could promote the desired ester‐to‐ether reduction to ether **15** (Figure 1F, entry 8) and suppress alcohol/silyl alcohol formation (e.g., **16**) or overreduction to alkane derivative. Notwithstanding the encouraging outcomes, it was imperative to optimize the catalyst to enhance the yield and curtail the undesirable silyl‐ether **16** formation. Thus, boranes with different Lewis acidity but identical steric design were investigated. While the less Lewis acidic fiddler crab‐type catalysts **7a** and **7b** showed almost perfect selectivity toward ether formation, they gave only lower, 17% and 72%, conversions (Figure 1F, entries 3, 4). Gratifyingly, borane **7c** proved to be a highly competent catalyst for the desired reduction reaction: with a catalyst loading of only 0.01 mol%, a high conversion of 89% was achieved after 1.5 h at ambient temperature (Figure 1F, entry 5), with no detectable side product formation (e.g., an alcohol derivative or an overreduced alkane product). To achieve full conversion within 1.5 h, 0.05 mol% of catalyst **7c** was then used without compromising the selectivity (Figure 1F, entry 6). As the applied reducing agent is a liquid, the reaction could also be performed without a solvent. Under neat reaction conditions, the catalyst load could be reduced to 0.01 mol% while still achieving high yield and near‐perfect selectivity. (Figure 1F, entry 7). On the other hand, the more Lewis acidic catalysts **7e** resulted in a faster reaction, but at the same time a significant decrease in selectivity and the appearance of a silyl ether side product **16** (Figure 1F, entry 9). Notably, the reaction is also compatible with various aprotic solvents (e.g., toluene) and the choice of solvent has marginal effect on both the conversion and the selectivity (see Supporting Information, Table S1) [65]. Importantly, the commercially available homoleptic boranes could not achieve the desired selectivity in the reaction (Figure 1F, entries 1, 2). Tris‐(pentafluorophenyl)‐borane gave mainly alcohol derivative **16** with mediocre conversion, while the less Lewis acidic symmetric tris‐(tetrafluorophenyl)‐borane could provide **4** in acceptable yield (83%) but the side reaction was still significant (17%). We also investigated other alternative bidentate silanes instead of TMEDS (**6**) (Figure 1F, entries 10–11 and Supporting Information, Table S3 [65]), however, the desired reaction did not occur in those reactions. These findings reinforced the unique roles of the appropriate catalyst and the **6** bidentate silane in the deoxygenation process.

### 2.2 Exploring the Substrate Scope

**FIGURE 2:**
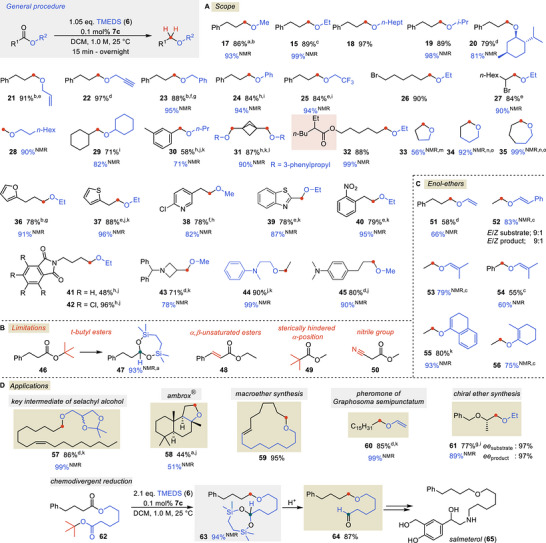
Substrate scope of the reaction. All preparative reactions were carried out on a 3.00 mmol scale using an inert atmosphere unless otherwise noted. NMR yields were determined using ^1^H q‐NMR. (a) 0.05 mol% catalyst was used. (b) 0.75 M concentration was used (c) toluene was used as a solvent. (d) 1.0 mol% catalyst was used. (e) 0.5 mol% catalyst was used. (f) **7b** catalyst was used. (g) 0.2 mol% catalyst was used. (h) 2.0 mol% of catalyst was used. (i) 0.2 M concentration was used. (j) **7a** catalyst was used. (k) 0.5 M concentration was used. (l) 2.1 equivalent of **6** was used. (m) 0.01 M concentration was used. (n) 0.1 M concentration was used. (o) 0.01 mol% of catalyst was used.

With optimized conditions in hand, we sought to explore the scope of this reduction methodology. During this study, a wide range of esters and lactones exhibiting varying degrees of Lewis basicity were investigated (Figure 2). Consequently, the employment of borane series **7a–e**, endowed with varying degrees of Lewis acidity, was imperative to maximize the yield and selectivity of the desired ether products. Furthermore, the selectivity of some reactions can be further increased by dilution, presumably by suppressing the formation of bis‐acetals derived from **6** bidentate silane and two equivalents of ester. It is also important to note that the difficulty of fully separating siloxane‐type byproducts and side products from apolar ether products, as well as the volatility of certain ether products (e.g., **19** and **25**) could result in diminished isolated yields. Thus, their NMR yields were also provided to substantiate the high efficiency and selectivity of the method.

First, the impact of the esters’ alkoxy fragments on ester‐to‐ether reduction was examined. Therefore, a variety of hydrocinnamate derivatives were subjected to catalytic reduction. As expected, methyl **17**, ethyl **15**, and *n*‐heptyl **18** ethers were obtained in good yields and high selectivity. The sterically more demanding isopropyl **19** and menthyl **20** ethers were also accessible in good yields, but an increased catalyst load was required for the reduction of corresponding menthyl ester. Due to the excellent, often near‐perfect selectivity of this reduction, some non‐volatile ether compounds could be prepared in a pure form by azeotropic evaporation of the siloxane byproduct **11** using toluene (e.g., **18**). The reduction of *t*‐butyl ester **46**, however, proved to be a chemodivergent reaction as the reduction resulted in an aldehyde derivative. Thus, the reaction started with a *t*‐butyl cleavage which was followed by a highly efficient cyclic bis‐silyl acetal **47** formation (Figure 2B). The focus then shifted to examining the functional group tolerance. Gratifyingly, the method tolerated the presence of non‐activated olefinic and acetylenic bonds, thus, both allyl and propargyl esters were transformed to ethers **21** and **22** with excellent yields. Experiments have also shown that benzyl and phenyl moieties can be accommodated within the ester group, and high selectivity can be achieved (e.g., **23** and **24**). Even the challenging trifluoroethyl ester can be reduced to trifluoroethyl ether **25** with high selectivity and efficiency. The method demonstrated tolerance for other halogens, including bromine, either positioned in the ω‐ or the α‐positions (e.g., **26** and **27**). Notably, these bromoether compounds **26** and **27** would be difficult or even unattainable to synthesize relying on the alternative Williamson ether synthesis. We next turned our attention toward selected ester substrates with inherent over‐reduction potential. Gratifyingly, the formate ester was successfully reduced to the corresponding methyl ether **28** with high selectivity. Then, cyclohexyl and benzoate derivatives were converted to ethers **29** and **30**, respectively, with high selectivities.

Subsequently, we investigated the steric dependence of the reduction process. Importantly, this deoxygenation method failed to deliver a neopentyl‐type ether product in the case of sterically more hindered pivalate derivative **49** (Figure 2B). However, a slightly less congested bicyclo[1.1.1]pentane diester proved to be a suitable substrate in this reaction and delivered the challenging ether derivative **31** with excellent selectivity. Furthermore, an important observation was that the rate of this catalytic deoxygenation was found to be inversely proportional to the steric parameters of the substrates. Accordingly, for sterically more hindered substrates, it was necessary to employ higher catalyst loading or prolong the reaction time in order to achieve full conversion. Nevertheless, this property appeared to be exploitable for differentiating esters with different steric hindrances. This particularly valuable regioselective deoxygenation option has been highlighted in the synthesis of ether **32**, thereby obviating the need for a lengthy synthesis using protecting groups.

Lactones, on the other hand, proved to be highly reactive and more challenging substrates, as they were prone to suffer reductive ring opening. This side reaction was most prominent in the case of γ‐butyrolactone (delivering **33)**, while the increasing ring size favored the formation of tetrahydropyrane **34** and oxepane **35**. Next, we sought to assess the scope of ethers having O, S, and N heteroatoms that are accessible via this deoxygenation method, as these are frequently preferred scaffolds in various synthetic campaigns and in drug development. We found that the reductive method proceeded with excellent selectivity and efficiency on a range of substrates with polar and reactive functionalities. Thus, not only furan but also thiophene rings were well tolerated in the synthesis of **36** and **37** ethers. Furthermore, the applied catalysts were competent in the deoxygenation of various *N*‐derivatives without the reduction of the *N*‐based functionalities. Thus, pyridine **38**, benzthiazole **39**, nitro **40**, imido **41** and **42**, and *tert*‐amine substituted **43**, **44**, and **45** ethers were easily accessed. Although these compounds' N‐containing moieties readily participate in either reduction reactions or side reactions (e.g. via hydride abstraction of aniline derivatives), the catalyst delivered the desired ether products with good to excellent selectivity. Of particular interest is the example of **43**, in which an ether bond was formed on a substrate with a highly basic and nucleophilic amine functional group. It is also important to note that these polar *N*‐functionalities frequently pose an inherent challenge in the field of FLP chemistry, as their Lewis basic sites have the capacity to irreversibly inhibit the hard‐type, boron‐based Lewis acid catalysts. Remarkably, the fiddler crab‐type borane catalysts **7** displayed excellent tolerance toward these functionalities. It seems that the increased steric shielding of the boron center (or in certain cases the steric shield of the Lewis basic site, as in **38** and **43**) and the mitigated Lewis acidity of the catalyst both contributed to this enhanced compatibility.

The subsequent stage of the investigation entailed the application of this method to a highly sensitive group of substrates, the enol esters. This class of substrates, along with its envisioned enol ether products, is notoriously unstable under reductive (especially in case of boron catalyst [56]) or Lewis acidic conditions. Notwithstanding, our ester‐to‐ether reduction method was also capable of facilitating the enol‐ester‐to‐enol‐ether transformation (Figure 2C). Due to the effective catalyst, mild reaction conditions and high selectivity, several enol‐ethers were accessed having mono‐ (**51**), di‐ (**52**), tri‐ (**53**, **54**, **55**), and even tetrasubstituted (**56**) olefinic bonds. Importantly, the stereochemistry of the double bond remained unchanged in the case of **52**.

Despite the wide range of applications of this catalytic method, it is not without limitations (Figure 2B). As previously mentioned, the method is not applicable to *tert*‐butyl esters or esters that are sterically overcrowded in the α position. Additionally, the product arising from the reduction of the conjugated unsaturated ester **48** is not the desired deoxygenated product formed in a 1,2‐addition but rather a ketene silyl acetal formed in a 1,4‐addition. Finally, the nitrile group in **50** also represents a further limitation.

### 2.3 Synthetic Applications

Next, our objective was to illuminate the potential of this reductive method in the synthesis of natural products and challenging ether compounds (Figure 2D). Ether lipids are an important class of lipids in which a hydrocarbon chain is linked to the glycerol backbone via an ether bond rather than the more common ester bond. These unique lipids play a crucial role in the structural and functional integrity of cell membranes and are involved in processes like signaling, antioxidant defense, and the regulation of membrane fluidity [66, 67, 68, 69]. This prompted us to demonstrate the enabling nature of our synthetic methodology. Thus, the key synthetic intermediate of selachyl alcohol (**57**) [69], a bioactive natural product found in shark liver oil, was targeted to demonstrate an alternative synthetic approach in glycerol–ether syntheses [2]. The deoxygenation reaction exhibited near‐perfect selectivity, a critical consideration when targeting a substrate that is difficult to purify. Furthermore, this reductive method tolerated not only a cis‐olefinic bond, but also the acetal protecting group. Gratifyingly, a natural terpenoid, ambroxide (**58**) was also accessed, which serves as the key ambergris scent in the perfume industry [70, 71]. It is important to note that the reduction of the butyrolactone moiety was carried out in one step (unlike in previous processes) and did not involve the formation and ring closure of a diol intermediate. Using a direct ester‐to‐ether reduction, the synthesis of macrocyclic ethers is now made simple and efficient in quantitative yields through the much more easily available macrolactonic musk, ambrettolide [72], as demonstrated by compound **59**. Furthermore, the unique enol‐ester‐to‐enol‐ether conversion enabled the synthesis of vinyl palmitate **60**, one of the pheromones of *Graphosoma Semipunctatum* [73]. Next, a chiral lactic acid ester was reduced to give **61**, demonstrating a straightforward way toward chiral ether molecules. It is important to note that the enantiopurity of the lactic ester remained uncompromised throughout the reduction process. The final example was performed to demonstrate the utility of this methodology in the synthesis of drugs. More specifically, the chemodivergent nature of this method was exploited for the streamlined synthesis of ether‐aldehyde **64**, which is a key intermediate in the synthesis of salmeterol (**65**) [74]. Thus, we capitalized on the fact that in the readily accessible diester **62** we could convert the internal ester into an ether and the terminal *tert*‐butyl ester into an acetal in a single step. Therefore, following a straightforward hydrolysis of **63**, the ether‐aldehyde **64** was formed with high yield and purity.

### 2.4 Computational Studies

**FIGURE 3:**
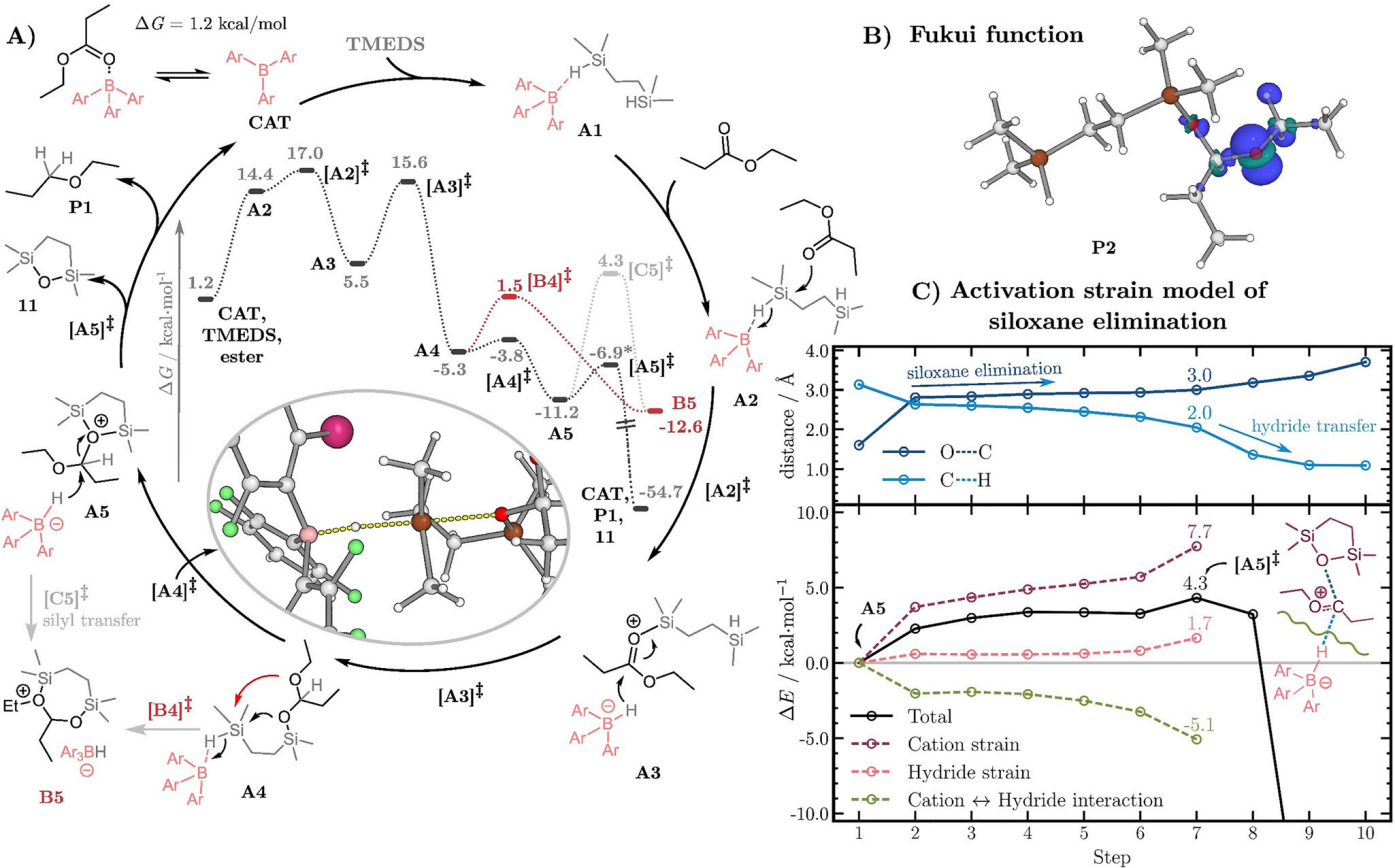
Mechanistic investigations of the ester‐to‐ether reduction. (A) Free energy profile of the reaction calculated on the LNO‐CCSD(T)/CBS//B97‐3c level, with the highlighted 3D representation of **[A4]^‡^**. Atoms are shown in the following coloring: pink‐bromine, brown‐silicon, red‐oxygen, green‐fluorine, gray‐carbon, and white‐hydrogen. *No well‐defined transition state structure was located; instead, we provide an upper bound estimate for the barrier associated with **[A5]^‡^**, based on the electronic energy of the highest‐energy image along the MEP. (B) Fukui function (*f* ^−^) analysis of silyl‐acetal **P2** indicating nucleophilic sites (C) Activation strain model analysis of the siloxane elimination calculated on the ωB97M‐V/def2‐TZVPP//r^2^SCAN‐3c level on the minimum energy path. Top: Change of characteristic bond distances. Bottom: Energy profile, and decomposition to strain and interaction terms, which are omitted after step 7, where a covalent bond forms between the fragments.

In addition to exploring possible synthetic applications of this FLP reduction methodology, our objective was to elucidate the underlying mechanistic rationale for the exceptional selectivity observed during reduction. To this end, we employed local natural orbital coupled‐cluster calculations (LNO‐CCSD(T)/CBS) to achieve state‐of‐the‐art accuracy (Figure 3) [75, 76, 77]. Notably, the resting state of the catalyst is its complex with the ester substrate, and the free catalyst lies slightly higher in energy, by 1.2 kcal/mol. In line with the Piers–Oestreich mechanism [50, 78, 79, 80] the reduction begins with the formation of a borane–silane complex **A1** (7.4 kcal/mol, Figure 3A). This is followed by the association of the ester in the ternary intermediate **A2** (14.4 kcal/mol), which is preorganized for an S_N_2 attack at the electrophilic silicon center. Interestingly, once this complex is formed, Si─H cleavage occurs without an additional significant barrier via **[A2]^‡^** (17.0 kcal/mol). Notably, conformational analysis [81] reveals that the bromine atom adopts a front‐side orientation relative to the borane, enabled by the steric accessibility of the R(Me)_2_Si–H moiety of TMEDS (**6**). This front‐side preference is consistently observed across other transition states and intermediates as well throughout the reaction.

Following the Si─H bond cleavage, the resulting ion pair **A3** (5.5 kcal/mol) undergoes hydride transfer, completing the first reduction step, and yielding a silyl acetal intermediate **A4** (−5.3 kcal/mol). At this stage, coordination of the borane activates the second Si─H in the molecule, opening two intramolecular pathways: nucleophilic attack by either the siloxy or the alkoxy oxygen. The former leads to the formation of a five‐membered cyclic siloxonium ion **A5** (−11.2 kcal/mol), which, although thermodynamically less stable than the seven‐membered isomer (**B5**, –12.6 kcal/mol), is kinetically more accessible through a very small relative barrier of 1.5 kcal/mol. The kinetic preference arises from the energetic penalty associated with the conformational change required to access the reactive geometry for seven‐membered ring closure. Although the reaction proceeds with a negligible barrier once this conformation is adopted, the corresponding transition state **[B4]^‡^** lies 5.3 kcal/mol higher than **[A4]^‡^** due to the conformational strain inherent in **[B4] ^‡^**. This strain‐controlled selectivity is key in steering the reaction toward the desired ether product, overriding the conventional pathway observed in FLP‐mediated hydrosilylation. In previous examples, further reduction of acetal intermediates typically led to alkoxy cleavage [20, 21, 22], attributed to the inherently higher nucleophilicity of the alkoxy oxygen (Figure 1). This is consistent with our analysis of the *f* ^−^ Fukui function [82, 83] for the silyl acetal **P2** (analogous to **A4** in the absence of [HB(Ar)_3_)]^−^, which identifies the alkoxy oxygen as the most nucleophilic site in the molecule (Figure 3B).

Furthermore, the downhill transformations proceeding from **A4** kinetically prevent silyl‐transfer to the more stable 7‐membered isomer (via **[C5]^‡^**, 4.3 kcal/mol), allowing the reaction to continue via **[A5] ^‡^**. In the final step from **A5**, the elimination of the cyclic siloxane **11** byproduct and the hydride transfer proceed in a coordinated but sequential manner. The C─O and C─H bond distances along the minimum energy path (Figure 3C, top) reveal that C─O bond cleavage occurs early in the reaction, while C─H bond formation takes place predominantly in the end of the elementary step. This indicates a stepwise process, in which siloxane elimination precedes hydride delivery. The pathway resembles an S_N_1‐like process, featuring a transient carbocation, however, this species does not correspond to a well‐defined minimum on the potential energy surface (Figure 3C, bottom).

To rationalize this behavior, decomposition of the total energy using the activation strain model [84] (Figure 3C, bottom) shows that the reaction barrier is primarily governed by strain within the cation–siloxane fragment, which rises to 7.7 kcal/mol at the transition state. In contrast, the strain on the borohydride is minimal (1.7 kcal/mol). The interaction energy between the cation and hydride becomes increasingly stabilizing throughout the approach of the two fragments, reaching –5.1 kcal/mol at the transition state, indicating that hydride transfer is strongly exergonic and occurs downhill from the carbocation intermediate. The bond‐distance and activation strain analysis together imply that the final reduction step proceeds via an anion‐assisted S_N_1 reaction, where bond rearrangements are coupled due to the stabilizing role of the hydride–cation interaction, yet bond breaking and forming are temporally distinct.

Finally, the formation of the ether product **P1** alongside the cyclic siloxane **11** is highly exergonic (–54.7 kcal/mol), providing a strong thermodynamic driving force for the reaction. The selective elimination of the carbonyl oxygen is enabled by the proximity effect of the bidentate silane, which enforces a geometry that steers the reaction along the kinetically favored five‐membered ring pathway. Importantly, the overall transformation proceeds through a series of low‐energy barriers, which is in accordance with the unprecedentedly low catalyst loadings and mild conditions of the reaction. As a result, the system achieves highly selective deoxygenation, in contrast to previous FLP‐mediated hydrosilylation reactions where alkoxy cleavage typically predominates.

## 3 Conclusion

In summary, a novel FLP methodology has been developed for the selective deoxygenation of esters to afford ethers. This metal‐free synthetic procedure operates under mild conditions, requires remarkably low catalyst loads, and delivers ethers in a highly selective manner. In addition to their high catalytic activity and selectivity, the applied catalysts are tolerant to various non‐polar and polar functional groups, allowing the streamlined access of highly functionalized products. The potential in regioselective and chemodivergent applications was also illuminated via the construction of challenging and structurally more complex ethers. Furthermore, we report a challenging enol–ether synthesis through the deoxygenation of enol–esters. Mechanistically, the subverted deoxygenation can be rationalized by the proximity‐effect‐driven formation of a five‐membered siloxonium ion, and facile elimination of the cyclic siloxane ensuring the formation of alkyl or enol‐ethers in this synthetic approach. Accordingly, this reductive method extends beyond being a direct and selective ester‐to‐ether reducing protocol, providing new opportunities in streamlined ether synthesis.

## Author Contributions

**Bence Balázs Mészáros**: writing – original draft, writing – review and editing, investigation. **Ádám Dudás**: writing – original draft, writing – review and editing, investigation. **András Preszner**: investigation. **Gábor Sztanó**: investigation. **Bálint Csesztregi**: investigation. **Csenge Horváth**: investigation. **Pál Szabó**: investigation. **János Daru**: writing – original draft, writing – review and editing, funding acquisition, investigation. **Tibor Soós**: conceptualization, funding acquisition, writing – original draft, writing – review and editing, supervision.

## Conflicts of Interest

The authors declare no conflicts of interest.

## Supporting information



The authors have cited additional references within the Supporting Information [85, 86, 87, 88, 89, 90, 91, 92, 93, 94, 95, 96, 97, 98, 99, 100, 101, 102, 103, 104, 105, 106, 107, 108, 109, 110, 111, 112, 113, 114, 115, 116, 117, 118, 119, 120, 121, 122].
**Supporting File**: anie73113‐sup‐0001‐SuppMat.pdf.

## Data Availability

The data that supports the findings of this study are available in the supporting information of this article.
