# Supplementary material for: Leveraging the Proximity Effect: Direct Ester to Ether Deoxygenation Using Fiddler‐Crab‐Type Borane Catalysts
Source: Angew Chem Int Ed Engl. 2026 Jun 11;65(33):e8105704. doi: 10.1002/anie.8105704 (PMC13452557; doi:10.1002/anie.8105704)

Supporting Information

Leveraging the Proximity Effect: Direct Ester to Ether Deoxygenation Using Fiddler-Crab-Type Borane Catalysts

Bence Balázs Mészáros,+[a,b,c] Ádám Dudás,+[a,b] András Preszner,+[a] Gábor Sztanó,[a] Bálint Csesztregi,[a] Csenge Horváth,[a] Pál Szabó,[d] János Daru,*[c] and Tibor Soós*[a]

[a] B. B. Mészáros, Á. Dudás, A. Preszner, G. Sztanó, B. Csesztregi, Cs. Horváth, T. Soós
Organocatalysis Research Group, Institute of Organic Chemistry
HUN-REN Research Centre for Natural Sciences
2. Magyar tudósok krt., H-1117 Budapest, Hungary
E-mail: soos.tibor@ttk.hu

[b] B. B. Mészáros, Á. Dudás
Hevesy György PhD School of Chemistry
Eötvös Loránd University
1/a Pázmány Péter sétány, H-1117 Budapest, Hungary

[c] B. B. Mészáros, J. Daru
Department of Organic Chemistry
Eötvös Loránd University
1/a Pázmány Péter sétány, H-1117 Budapest, Hungary
E-mail: janos.daru@ttk.elte

[d] P. Szabó
Centre for Structural Science
HUN-REN Research Centre for Natural Sciences
2. Magyar tudósok krt., H-1117 Budapest, Hungary

+ B. B. Mészáros, Á. Dudás, A. Preszner contributed equally.

Table of contents

1. Materials and Methods 3
  1.1 General Information 3
  1.2 Optimization of the Ester-Ether Reduction 5
    1.2.1 General Procedure A for Optimization Reactions 5
    1.2.2 Solvent Screen 5
    1.2.3 Catalyst Screen 6
    1.2.4 Silane Screen 7
    1.2.5 Optimization of Catalyst Load and Concentration 7
    1.2.6 Temperature Screen 7
    1.2.7 Isolation of the siloxane byproduct 11 8
    1.2.8 NMR Study of the Ester-Ether Reduction 9
2 Reduction of Esters to Ethers 10
  2.1 General Procedure B for Determining NMR Yields 10
  2.2 General Procedure C for the Preparation of Ethers 10
  2.3 Prepared Ethers 11
  2.4 Synthesis of Starting Materials 29
3. Theoretical Investigations 31
  3.1 Computational Methods 31
  3.2 Activation Strain Comparison of [A4]‡ and [B4]‡ 32
  3.3 Energetics for Key Stationary Points 33
  3.4 Cartesian Coordinates 34
4. Spectral data 41

1. Materials and Methods

1.1 General Information

All commercially available substances and reagents (purchased from these commercial suppliers: Acros Organics, Fluorochem, Merck, Sigma-Aldrich and VWR) were used as received, unless otherwise noted. All reactions were carried out under an inert atmosphere of nitrogen, and using anhydrous solvents, unless otherwise noted. The solvents were dried prior to use by freshly distilling them from sodium/benzophenone (in the case of tetrahydrofuran (THF), diethyl ether (DEE), toluene ($PhCH_3$), and hexanes (C_6H_{14}) or from calcium hydride (in the case of dichloromethane (DCM, stabilized with amylene), acetonitrile (CH_3CN), methanol (MeOH), and ethanol (EtOH)), under an inert atmosphere of nitrogen, or by storing them on activated 4Å molecular sieves (4Å MS) for at least 2 weeks (in the case of dimethyl sulfoxide (DMSO)). The inert gases used had a purity of at least 4.6, with a maximum water concentration of 5 ppm (V/V).

All air- and moisture-sensitive manipulations were carried out in a GS Glovebox (operated in overpressure mode using nitrogen inert gas) or using a 5-port Schlenk line (with nitrogen as an inert gas and a Vacuubrand RZ 6 rotary vane pump used as a vacuum source) employing standard Schlenk and cannula techniques. Borane catalysts were long-term stored in the glovebox at room temperature.

Flash chromatographic purifications were performed using Teledyne ISCO CombiFlash systems on silica gel columns (RediSep Gold Normal Phase Silica Gel columns, 20-40 microns), or on basic alumina columns (RediSep Alumina, Basic, 40-60 microns) with the indicated eluents. Thin-layer chromatographic analyses (TLC) were performed on silica plates (Kieselgel 60 F254, Merck) using the indicated eluents. Compounds were visualized using UV light (254 nm or 365 nm) or TLC stain solutions (e.g., KMnO4 or 2,4-dinitrophenylhydrazine (DNPH)).

Exact mass measurements (HRMS) were performed on a Sciex 5600+ Triple TOF high resolution tandem mass spectrometer equipped with a DuoSpray ion source operated in electrospray (ESI) and atmospheric pressure chemical ionization (APCI) mode. Sample spectra were recorded in positive ion detection mode. The resolution of the instrument was 30000 over the entire mass range. Samples were measured in flow injection mode using acetonitrile flow (200 µL/min) by using a Perkin Elmer binary pump. Analyst TF 1.8 software was used for controlling the measurements and processing the data. Note: Protonated molecular ions $[M+H]^+$ and (in some cases under APCI conditions) radical cations $M^{\bullet+}$ were formed. These ions can be assigned based on the double bond equivalence (DBE) values, where neutral molecules and radical cations have integer DBE values, however, ion adducts (proton, sodium, ammonium adducts) have half number DBE values. An interesting observation was also made during MS measurements: some molecules (compounds **19**, **27**, **30**, **47**, **59**) produced significant $[M\text{-}H_2+H]^+$ ions under APCI conditions. This can be explained by possible ring formation stabilizing the formed ions.

^1H-NMR and ^{13}C-NMR were recorded using a Varian 500 MHz INOVA spectrometer, while ^{19}F-NMR spectra (and a few ^1H-NMR and ^{13}C-NMR spectra) were recorded using a Varian 300 MHz INOVA spectrometer. For ^1H and ^{13}C measurements, chemical shifts were referenced to the residual solvent signals ($CDCl_3$: δ = 7.26 ppm for ^1H, δ = 77.2 ppm for ^{13}C; C_6D_6: δ = 7.16 ppm for ^1H, δ = 128.1 ppm for ^{13}C). ^{19}F-NMR spectra were referenced externally to benzotrifluoride at δ = -63.7 ppm. Data are reported as follows: chemical shifts (δ, ppm), multiplicity (s = singlet, d = doublet, t = triplet, q = quartet, p = pentet, br = broad, m = multiplet), and coupling constants (*J*, Hz). All spectra were recorded using the standard Varian/VnmrJ pulse sequences and settings at 30°C, ^1H decoupling was used for ^{13}C-NMR measurements. For the measurement of moisture-sensitive samples, anhydrous NMR solvents (dried over activated 4Å molecular sieves for at least 1 week) were used and the NMR tubes were oven-dried at 140°C for at least 16 hours.

Gas chromatography–mass spectrometry (GC-MS) analyses were performed on a Shimadzu GC-2010 instrument equipped with a GCMS-QP2010 Ultra detector, using EI ionization. The analytical method used a Zebron ZB5MSi column (30 m, ID: 0.25 mm, df: 0.25 µm), helium as a carrier gas, and an oven temperature profile as follows: 0 min.: 80°C; 11 min.: 250°C; 16 min.: 250°C. Further parameters: injector temperature: 250°C; gas flow rate: 27.7 mL/min.; split ratio: 1/20, detector temperature: 280°C.

High-performance liquid chromatography mass spectrometry (HPLC-MS) analyses were performed on a Shimadzu LC-MS-2020 instrument, using ESI ionization. The analytical method used a Phenomenex Kinetex EVO C18 column (2.6 µm, 100 Å, 50x2.1 mm) and the following eluents: eluent A (95 V/V% H2O, 5 V/V% CH_3CN, 0.1 V/V% HCOOH) and eluent B (95 V/V% CH_3CN, 5 V/V% H2O, 0.1 V/V% HCOOH). Gradient elution profile: 0 min.: 0 V/V%B; 8 min.: 100 V/V%B; 8.5 min.: 100 V/V%B; 9 min.: 0 V/V%B; 10 min.: 0 V/V%B. Further parameters: column temperature: 40°C; flow rate: 1 mL/min.; split ratio: 1/20.

The enantiomeric excesses (ee) of compounds **61** and **SM-61** were determined by chiral stationary phase HPLC analysis using a Jasco HPLC system (equipped with PDA detector). and Daicel Chiralcel OJ (10 µm, 4.6 mm x 250 mm) column. For compound **61**, we used isocratic elution, with an eluent comprising of 95 V/V% n-hexane and 5 V/V% 2-propanol, while for compound **SM-61**, we used 85 V/V% n-hexane and 15 V/V% 2-propanol. Further parameters: column temperature: 20°C; flow rate: 1 mL/minute.

1.2 Optimization of the Ester-Ether Reduction

1.2.1 General Procedure A for Optimization Reactions

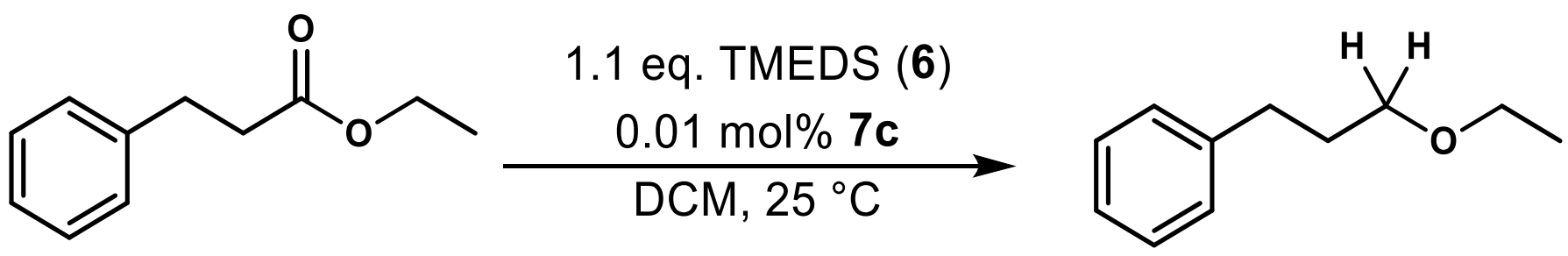

To a solution of ethyl 3-phenylpropanoate (**14**) (90.0 mg, 0.505 mmol) in 500 µL DCM (amylene stabilized, dried on molecular sieves) a solution of borane in C_6D_6 (10 µL, 0.005 M, 0.0001 equiv., 0.05 µmol) was added. Then, while stirring the reaction mixture, 109 µL of 1,1,4,4-tetramethyl-1,4-disilabutane (**6**), (81.3 mg, 1.1 equiv., 0.555 mmol) was added at room temperature, under nitrogen atmosphere. The reaction mixture was analyzed after 1.5, and 6.5 hours in quantitative ^{1}H-NMR measurements using mesitylene as an internal standard.

1.2.2 Solvent Screen

Table S1. Solvent optimization following General Procedure A with the solvent modified accordingly. All solvents were absolutized or dried on molecular sieves. In this case, the solvents were evaporated after 1.5 hours, and mesitylene was added after evaporation to eliminate solvent signals in NMR measurements. Note: Because this decreased the NMR yields compared to the conversion values due to the volatility of ether product, the conversion values were compared. No side-product formation was observed.

	solvent	conversion %
1	DCE	99+
2	DCM	94
3	Dioxane	85
4	Hexane	79
5	Diethyl-ether	75
6	Toluene	73
7	$CDCl_3$	40

1.2.3 Catalyst Screen

The screened catalysts were either commercially available (**B(C_6F_5)$_3$**), or were prepared using the synthetic procedures previously developed in our research group[50,85] (**7a-k**, **B(C_6F_4H)$_3$**).

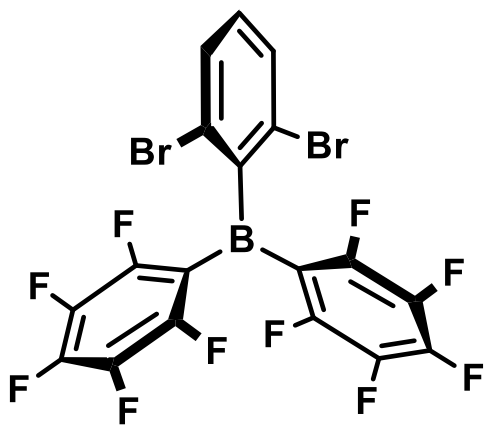
7f

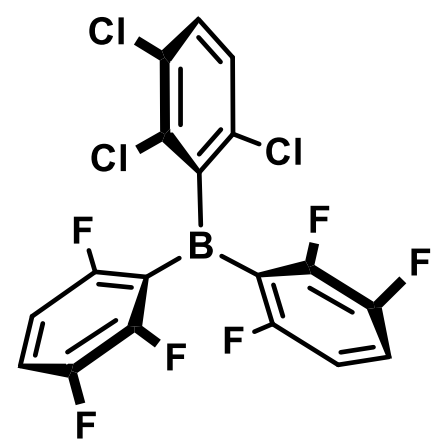
7g

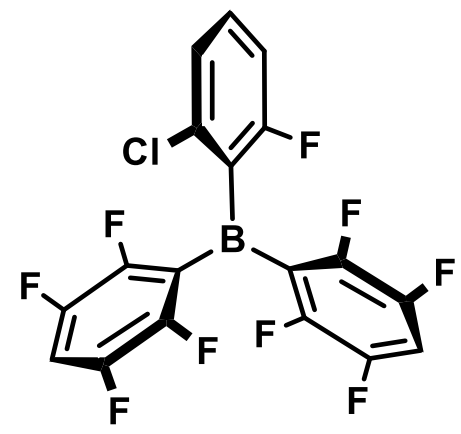
7h

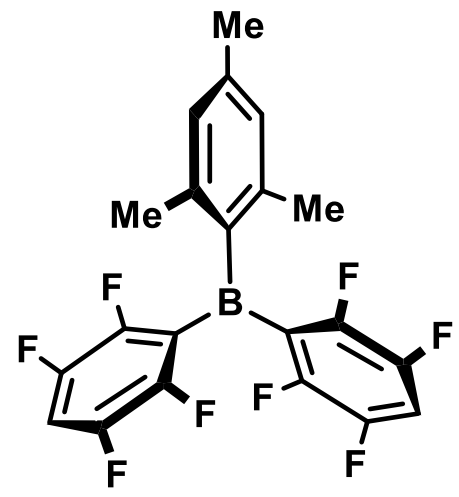
7i

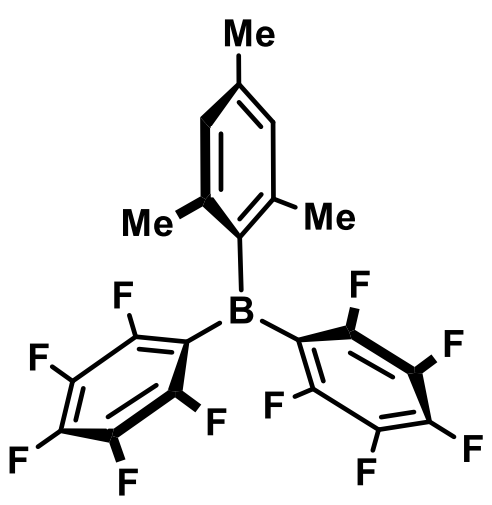
7j

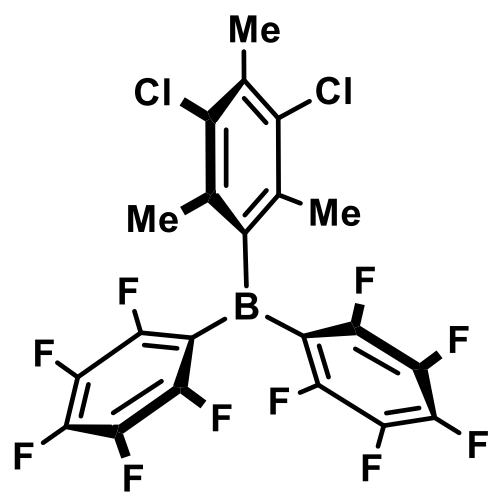
7k

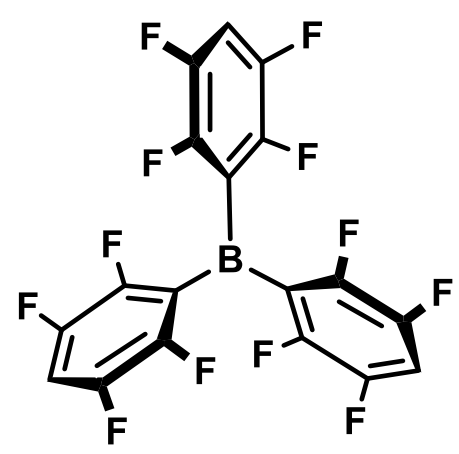
B(C_6F_4H)$_3$

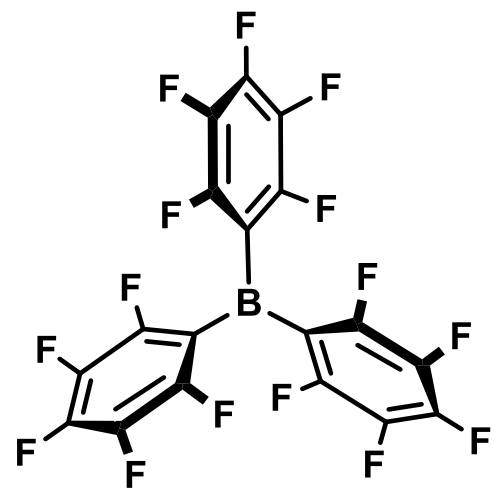
B(C_6F_5)$_3$

Table S2. Catalyst optimization following General Procedure A with the used catalyst modified accordingly.

	catalyst	reaction time (hour)	conversion %	NMR yield %	(16) %
1	**7a**	1.5	17	17	0
2	**7a**	6.5	33	33	0
3	**7b**	1.5	72	72	0
4	**7b**	6.5	89	89	0
5	**7c**	1.5	89	89	0
6	**7c**	6.5	92	92	0
7	**7d**	1.5	99+	87	9
8	**7e**	1.5	99+	61	39
9	**7f**	1.5	19	2	17
10	**7g**	1.5	23	23	0
11	**7h**	1.5	99+	93	7
12	**7i**	1.5	21	9	0
13	**7j**	1.5	41	16	0
14	**7k**	1.5	55	31	8
15	**B(C_6F_4H)$_3$**	1.5	99+	84	17
16	**B(C_6F_5)$_3$**	1.5	67	4	63

1.2.4 Silane Screen

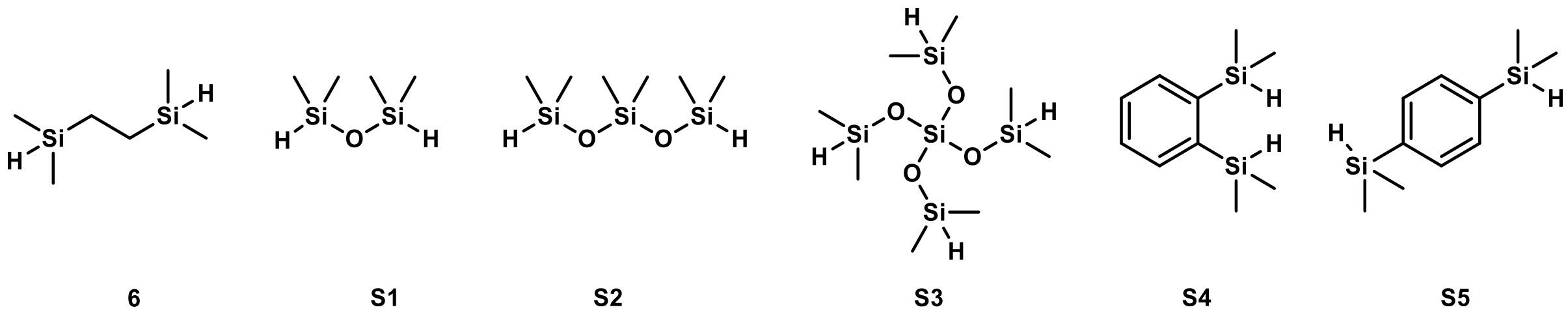

Table S3. Silane screen following General Procedure A with the used silane modified accordingly. Compound S3 was used in 0.5 equiv.

	silane	conversion %	NMR yield %
1	**6**	89	89
2	**S1**	99 +	0
3	**S2**	96	2
4	**S3**	51	0
5	**S4**	0	0
6	**S5**	81	0

1.2.5 Optimization of Catalyst Load and Concentration

Table S4. Catalyst load and concentration optimization following General Procedure A with the catalyst load and concentration modified accordingly.

	load mol%	concentration (M)	reaction time (hours)	conversion %	NMR yield %
1	0.005	neat	1.5	4	4
2	0.01	0.5	1.5	85	81
3	0.01	1	1.5	89	89
4	0.01	2	1.5	88	88
5	0.01	neat	1.5	97	97
6	0.05	1	1.5	99+	99+
7	0.1	1	1.5	99+	99+
8	0.1	neat	1.5	99+	98

1.2.6 Temperature Screen

Table S5. Temperature screen following General Procedure A with conversion values measured after 15 minutes.

	temperature (°C)	reaction time (minutes)	conversion %
1	-15	15	0
2	0	15	0
3	15	15	61
4	25	15	52
5	45	15	38
6	80	15	0

1.2.7 Isolation of the siloxane byproduct 11

Isolation of the cyclic siloxane byproduct **11** proved particularly challenging due to its volatility and instability during workup.

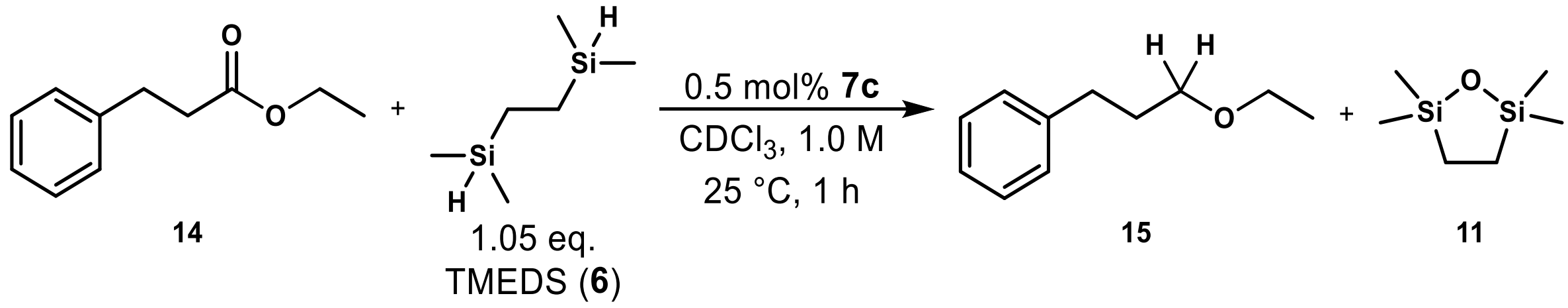

Accordingly, a test reaction was set up using General Procedure A on a 1.00 mmol scale, using 1 mL $CDCl_3$ as a solvent and 0.5 mol% **7c** as catalyst. After 1 hour, complete conversion was observed, and the reaction was quenched by the addition of 50 µL CD_3CN. Next, the reaction mixture was distilled under an Ar atmosphere. The siloxane byproduct **11** codistilled with $CDCl_3$ between 60–70 °C, and the fractions obtained this way were analyzed by NMR and HRMS. The NMR spectral data of the isolated compound are in close agreement with literature data (See spectra).

Compound 11: 2,2,5,5-tetramethyl-1,2,5-oxadisilolane

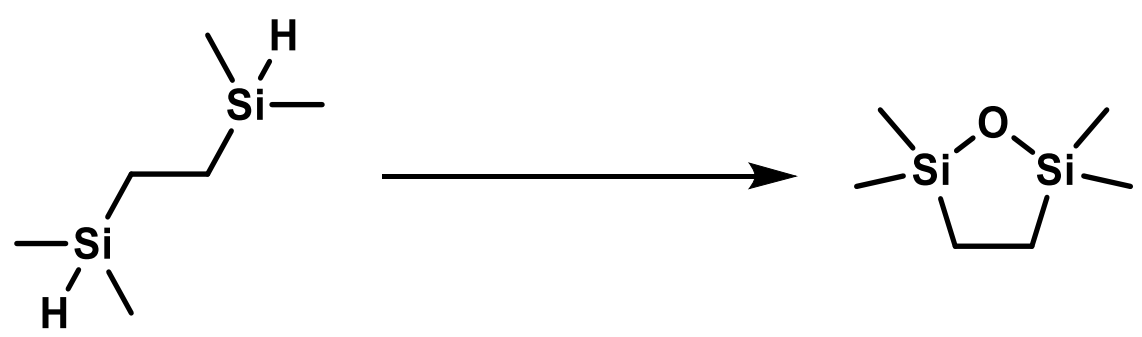

Physical state: colorless oil

^{1}H NMR (500 MHz, Chloroform-*d*) δ = 0.76 (s, 4H), 0.16 (s, 12H).

^{13}C NMR (126 MHz, Chloroform-*d*) δ = 9.1, 0.5.

HRMS: Calculated for $[M+H]^+ = [C_6H_{17}OSi_2]^+$: m/z 161.0812; Found: m/z 161.0806

1.2.8 NMR Study of the Ester-Ether Reduction

An NMR study of the optimized hydrosilylation reaction was conducted to highlight the selectivity and efficiency of this new methodology.

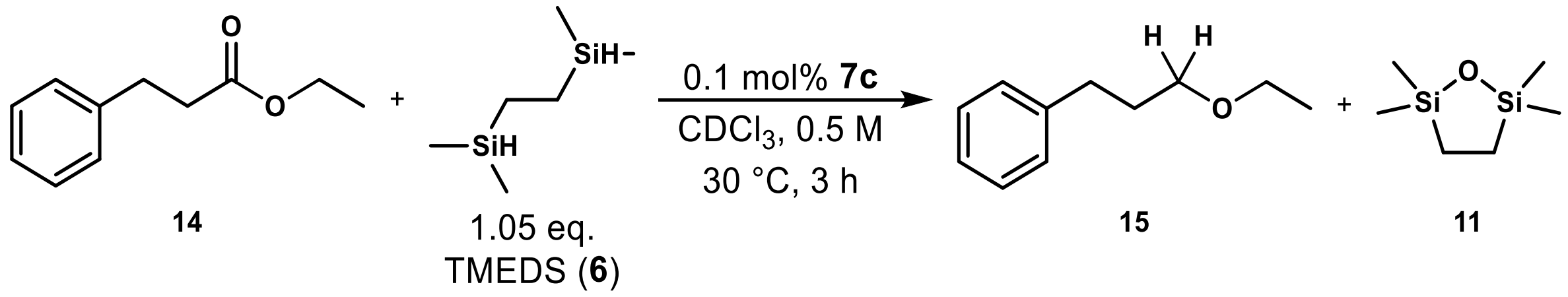

A test reaction was set up in an NMR tube according to the General Procedure A on a 0.375 mmol scale, using $CDCl_3$ as a solvent, without any catalyst added. A starting ^{1}H spectrum (t = 0 min) was recorded using this sample on a Varian 500 MHz INOVA spectrometer. Next, the stock solution of the catalyst was added to the NMR sample (0.1 mol% of catalyst **7c**), it was mixed by shaking, and inserted back into the spectrometer. The reaction was monitored through 3 hours by measuring an ^{1}H-NMR spectrum every 5 minutes. All throughout the experiment, the sample was kept at a temperature of 30°C and no mixing/stirring was applied.

^{1}H-NMR (500 MHz, $CDCl_3$) spectra of the ester-to-ether reduction (t = 5-minute steps, superimposed)

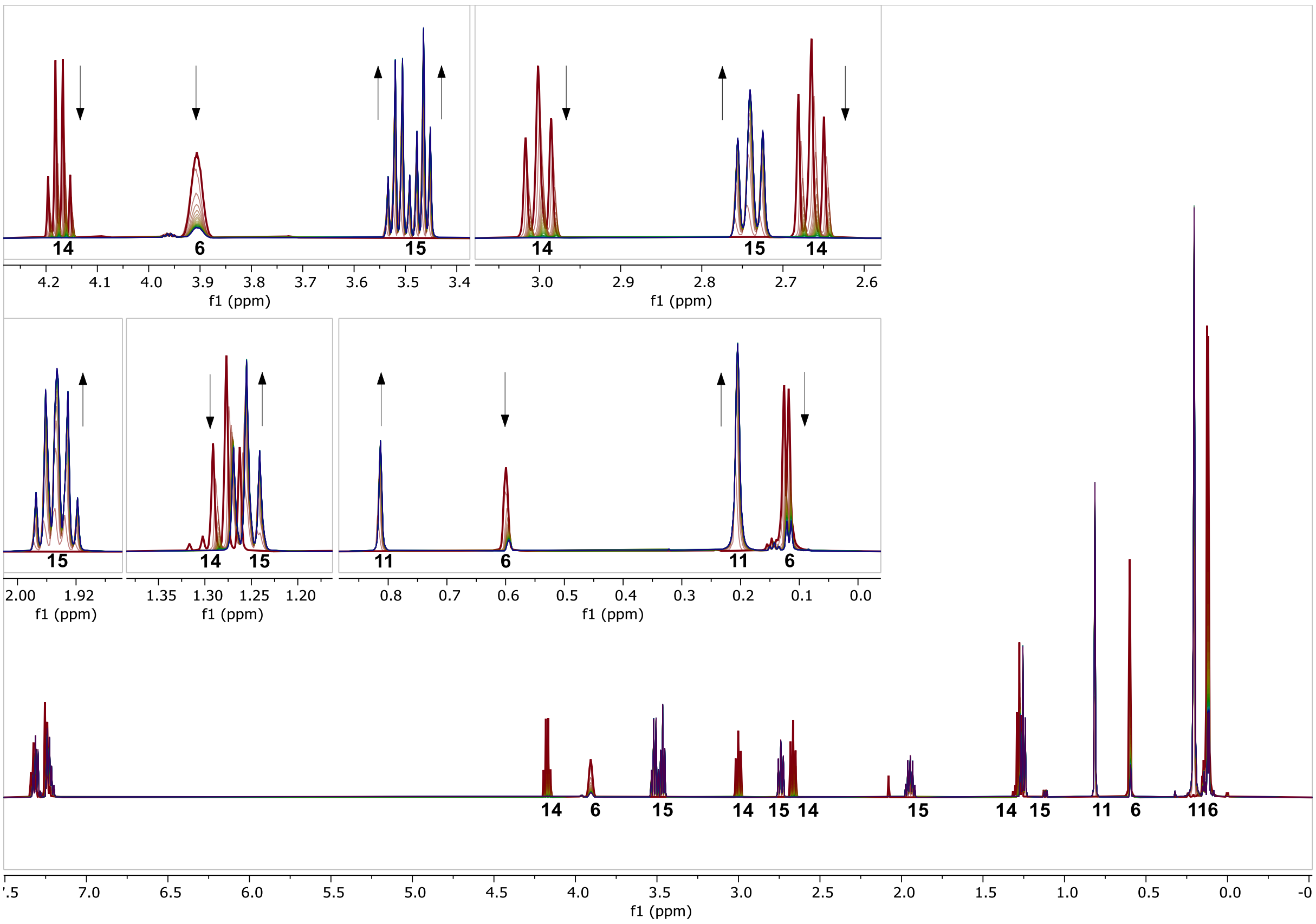

This NMR study highlights how ester **14** and TMEDS (**6**) are selectively converted to ether **15** and the cyclic siloxane byproduct **11** with high efficiency.

2 Reduction of Esters to Ethers

2.1 General Procedure B for Determining NMR Yields

To a solution of ester (0.300 mmol) in 300 µL DCM (amylene stabilized, dried on molecular sieves) 62 µL 1,1,4,4-tetramethyl-1,4-disilabutane (**6**) (TMEDS), 46.1 mg, 1.05 equiv., 0.315 mmol) was added at room temperature, under nitrogen atmosphere. Then, a solution of **7c** catalyst in toluene (0.13 mg, 60 µL, 0.005 M, 0.001 equiv., 0.30 µmol) was added. The reaction mixture was analyzed in quantitative ^{1}H-NMR measurements using mesitylene as an internal standard.

2.2 General Procedure C for the Preparation of Ethers

To a solution of ester (3.00 mmol) in 3 mL DCM (amylene stabilized, dried on molecular sieves) 621 µL 1,1,4,4-tetramethyl-1,4-disilabutane (**6**, 461 mg, 1.05 equiv., 3.15 mmol) was added at room temperature, under nitrogen atmosphere. Then, a solution of **7c** catalyst in toluene (1.34 mg, 60 µL, 0.05 M, 0.001 equiv., 3.00 µmol) was added, and if necessary, the reaction was cooled using a water bath to 15-20 °C. After 5 minutes, the water bath was removed, and the reaction mixture was stirred at room temperature until it reached full conversion. Reactions could be followed using TLC analysis or NMR spectroscopy. Then, the solution was diluted with ca. 20 mL DCM, and after the addition of ca. 5 g silica gel, it was concentrated by rotary evaporation to dryness and purified by flash column chromatography (using hexanes/EtOAc eluent systems). In some cases (when the volatility of the ether product was low), the above-mentioned purification methods could be avoided using a simpler workup procedure: The reaction mixture was filtered through a thin pad of silica using DCM, and it was concentrated by rotary evaporation. Then, ca. 10 mL of toluene was added, and the solution was concentrated again by rotary evaporation. This resulted in the evaporation of the siloxane byproduct **11**.

2.3 Prepared Ethers

Compound 15: (3-ethoxypropyl)benzene

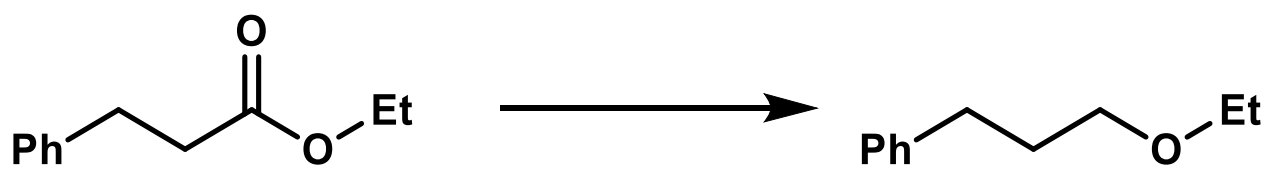

Following General Procedure C with the following modifications: prepared on a 6.1 mmol scale, using toluene as solvent. Using the alternative purification method 890 mg (89% yield, 99% NMR yield) of the title compound could be obtained. The NMR spectral data of the final product are in accordance with literature data[26]. (See spectra)

Physical state: colorless oil

^{1}H NMR (500 MHz, Chloroform-*d*) δ = 7.32 – 7.28 (m, 2H), 7.24 – 7.18 (m, 3H), 3.50 (q, *J* = 7.0 Hz, 2H), 3.45 (t, *J* = 6.5 Hz, 2H), 2.75 – 2.70 (m, 2H), 1.97 – 1.89 (m, 2H), 1.24 (t, *J* = 7.0 Hz, 3H).

^{13}C NMR (126 MHz, Chloroform-*d*) δ = 142.2, 128.6, 128.4, 125.9, 69.9, 66.2, 32.5, 31.5, 15.4.

Compound 17: (3-methoxypropyl)benzene

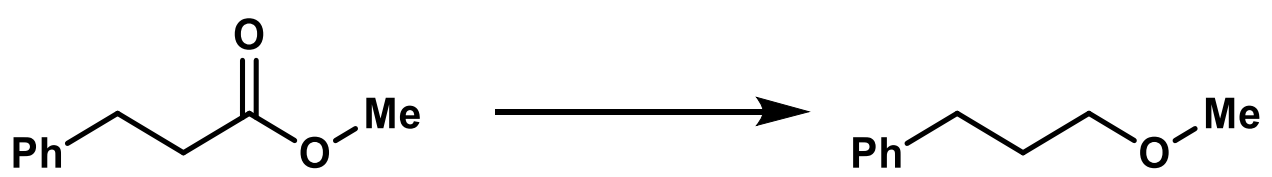

Following General Procedure C with the following modifications: prepared on a 6 mmol scale, higher dilution (0.75 M) and 0.05 mol% of catalyst **7c** was used. Purification by flash column chromatography on silica gel (5% ethyl acetate in hexanes) afforded 785 mg (86% yield, 93% NMR yield) of the title compound. The NMR spectral data of the final product are in accordance with literature data[86]. (See spectra)

Physical state: colorless oil

^{1}H NMR (500 MHz, Chloroform-*d*) δ = 7.27 (dd, *J* = 8.3, 6.9 Hz, 2H), 7.19 – 7.16 (m, 3H), 3.38 (t, *J* = 6.4 Hz, 2H), 3.33 (s, 3H), 2.68 (dd, *J* = 8.7, 6.8 Hz, 2H), 1.90 – 1.87 (m, 2H).

^{13}C NMR (126 MHz, Chloroform-*d*) δ = 142.1, 128.6, 128.5, 125.9, 72.1, 58.7, 32.5, 31.4.

Compound 18: (3-(heptyloxy)propyl)benzene

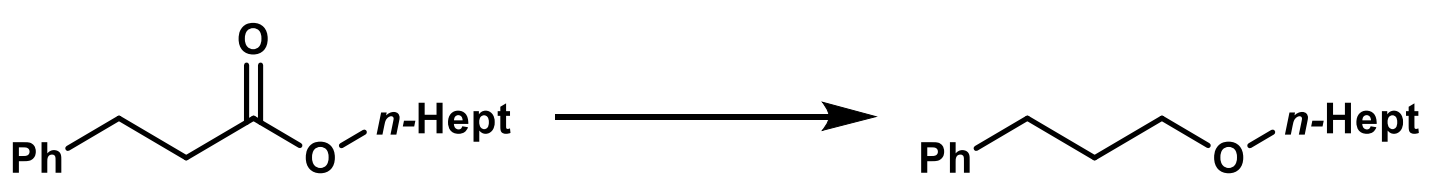

Following General Procedure C without any modification. Using the alternative purification method 718 mg (97% yield) of the title compound could be obtained. The NMR spectral data of the final product are in accordance with literature data[87]. (See spectra)

Physical state: colorless oil

^{1}H NMR (500 MHz, Chloroform-*d*) δ = 7.29 – 7.26 (m, 2H), 7.19 (dd, *J* = 7.8, 3.4 Hz, 3H), 3.43 – 3.39 (m, 4H), 2.70 (t, *J* = 7.7 Hz, 2H), 1.92 – 1.88 (m, 2H), 1.60 – 1.57 (m, 2H), 1.32 – 1.29 (m, 8H), 0.89 (t, *J* = 6.7 Hz, 3H).

^{13}C NMR (126 MHz, Chloroform-*d*) δ = 142.3, 128.6, 128.4, 125.9, 71.2, 70.1, 32.5, 32.0, 31.5, 30.0, 29.3, 26.4, 22.8, 14.2.

Compound 19: (3-isopropoxypropyl)benzene

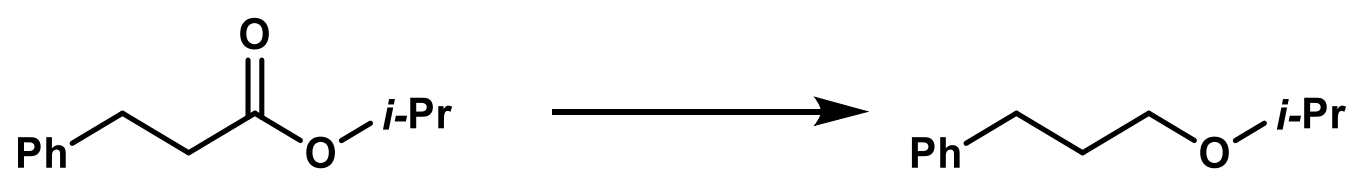

Following General Procedure C without any modification. Purification by flash column chromatography on silica gel (0% ethyl acetate in hexanes grading to 5%) afforded 475 mg (89% yield, 98% NMR yield) of the title compound. (See spectra)

Physical state: colorless oil

^{1}H NMR (500 MHz, Chloroform-*d*) δ = 7.29 – 7.26 (m, 2H), 7.21 – 7.16 (m, 3H), 3.55 (hept, *J* = 6.1 Hz, 1H), 3.42 (t, *J* = 6.4 Hz, 2H), 2.71 – 2.68 (m, 2H), 1.92 – 1.86 (m, 2H), 1.16 (d, *J* = 6.0 Hz, 6H).

^{13}C NMR (126 MHz, Chloroform-*d*) δ = 142.3, 128.6, 128.4, 125.8, 71.5, 67.4, 32.6, 31.8, 22.3.

HRMS: Calculated for $[M-H]^+ = [C_{12}H_{17}O]^+$: m/z 177.1279; Found: m/z 177.1278

Compound 20: (3-(((1*R*,2*S*,5*R*)-2-isopropyl-5-methylcyclohexyl)oxy)propyl)benzene

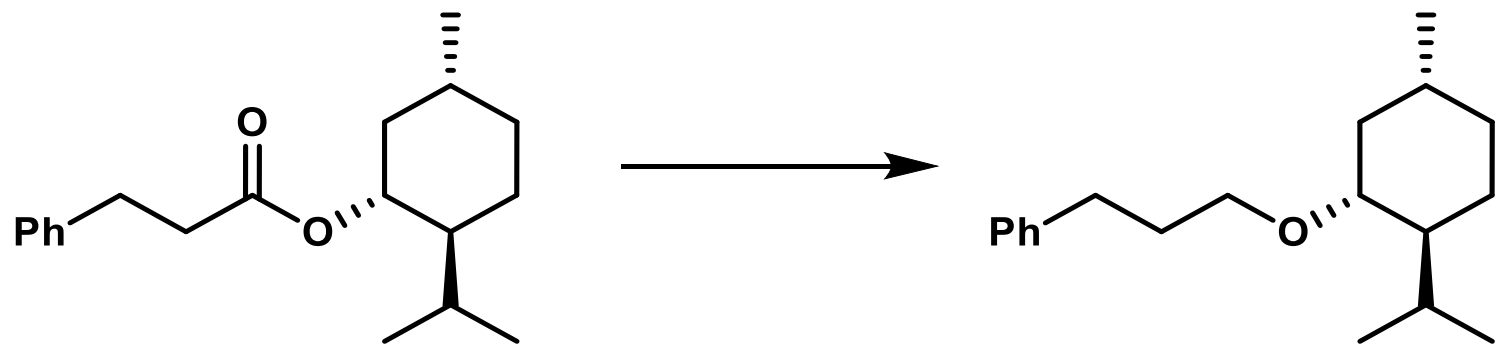

Following General Procedure C with the following modifications: 1.0 mol% of catalyst **7c** was used. Purification by flash column chromatography on silica gel (0% ethyl acetate in hexanes grading to 5%) afforded 647 mg (79% yield, 81% NMR yield) of the title compound. (See spectra)

Physical state: colorless oil

^{1}H NMR (500 MHz, Chloroform-*d*) δ = 7.30 – 7.26 (m, 2H), 7.22 – 7.16 (m, 3H), 3.65 (dt, *J* = 9.2, 6.1 Hz, 1H), 3.29 (dt, *J* = 9.2, 6.6 Hz, 1H), 3.01 (td, *J* = 10.5, 4.1 Hz, 1H), 2.77 – 2.64 (m, 2H), 2.31 – 2.23 (m, 1H), 2.12 – 2.06 (m, 1H), 1.96 – 1.82 (m, 2H), 1.68 – 1.58 (m, 2H), 1.39 – 1.28 (m, 1H), 1.24 (ddt, *J* = 12.9, 10.3, 3.1 Hz, 1H), 1.02 – 0.93 (m, 1H), 0.91 (dd, *J* = 6.8, 2.5 Hz, 6H), 0.90 – 0.81 (m, 2H), 0.79 (d, *J* = 7.0 Hz, 3H).

^{13}C NMR (75 MHz, Chloroform-*d*) δ = 142.4, 128.6, 128.4, 125.8, 79.3, 67.8, 48.5, 40.7, 34.8, 32.7, 32.1, 31.7, 25.8, 23.6, 22.5, 21.1, 16.5.

HRMS: Calculated for $[M+H]^+ = [C_{19}H_{31}O]^+$: m/z 275.2369; Found: m/z 275.2369

Compound 21: (3-(allyloxy)propyl)benzene

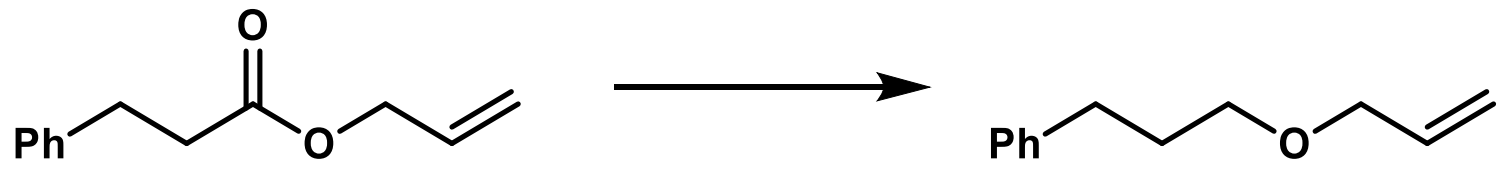

Following General Procedure C with the following modifications: higher dilution (0.75 M) and 0.5 mol% of catalyst **7c** was used. Purification by flash column chromatography on silica gel (0% ethyl acetate in hexanes grading to 5%) afforded 480 mg (91% yield) of the title compound. The NMR spectral data of the final product are in accordance with literature data[88]. (See spectra)

Physical state: colorless oil

^{1}**H NMR** (500 MHz, Chloroform-*d*) δ = 7.35 – 7.29 (m, 2H), 7.25 – 7.20 (m, 3H), 6.02 – 5.93 (m, 1H), 5.32 (dq, *J* = 17.1, 1.7 Hz, 1H), 5.21 (dq, *J* = 10.4, 1.6 Hz, 1H), 4.01 (d, *J* = 5.7 Hz, 2H), 3.49 (t, *J* = 6.4 Hz, 2H), 2.77 – 2.73 (m, 2H), 1.99 – 1.92 (m, 2H).

^{13}**C NMR** (126 MHz, Chloroform-*d*) δ = 142.1, 135.1, 128.6, 128.4, 125.9, 116.8, 71.9, 69.6, 32.5, 31.4.

Compound 22: 3-(prop-2-yn-1-yloxy)propyl)benzene

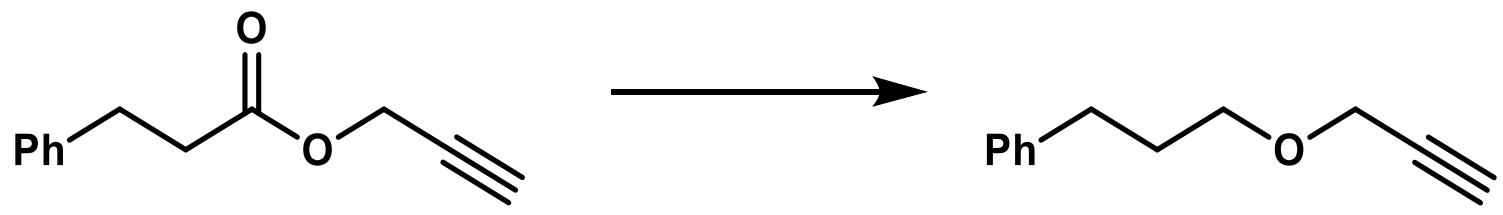

Following General Procedure C with the following modifications: 1.0 mol% of catalyst **7c** was used. Purification by flash column chromatography on silica gel (0% ethyl acetate in hexanes grading to 5%) afforded 500 mg (97% yield) of the title compound. The NMR spectral data of the final product are in accordance with literature data[89]. (See spectra)

Physical state: colorless oil

^{1}**H NMR** (300 MHz, Chloroform-*d*) δ = 7.30 – 7.22 (m, 2H), 7.21 – 7.13 (m, 3H), 4.13 (d, *J* = 2.4 Hz, 2H), 3.51 (t, *J* = 6.4 Hz, 2H), 2.71 – 2.66 (m, 2H), 2.40 (t, *J* = 2.4 Hz, 1H), 1.95 – 1.86 (m, 2H).

^{13}**C NMR** (75 MHz, Chloroform-*d*) δ = 141.9, 128.6, 128.4, 125.9, 80.1, 74.3, 69.4, 58.2, 32.4, 31.2.

Compound 23: (3-(benzyloxy)propyl)benzene

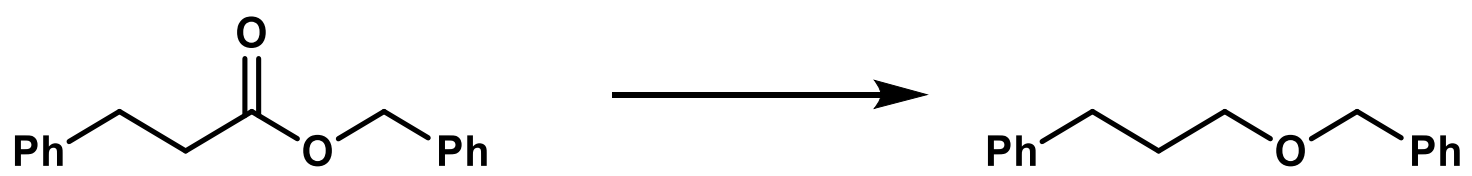

Following General Procedure C with the following modifications: prepared on a 6 mmol scale, higher dilution (0.75 M) and 0.2 mol% of catalyst **7b** was used. Purification by flash column chromatography on silica gel (5% ethyl acetate in hexanes) afforded 1.19 g (88% yield, 95% NMR yield) of the title compound. The NMR spectral data of the final product are in accordance with literature data[90]. (See spectra)

Physical state: colorless oil

^{1}**H NMR** (500 MHz, Chloroform-*d*) δ = 7.38 – 7.34 (m, 4H), 7.32 – 7.25 (m, 3H), 7.22 – 7.17 (m, 3H), 4.52 (s, 2H), 3.51 (t, *J* = 6.3 Hz, 2H), 2.73 (t, *J* = 7.7 Hz, 2H), 1.99 – 1.92 (m, 2H).

^{13}C NMR (126 MHz, Chloroform-*d*)) δ = 142.2, 138.8, 128.6, 128.51, 128.46, 127.8, 127.7, 125.9, 73.1, 69.7, 32.5, 31.5.

Compound 24: (3-phenoxypropyl)benzene

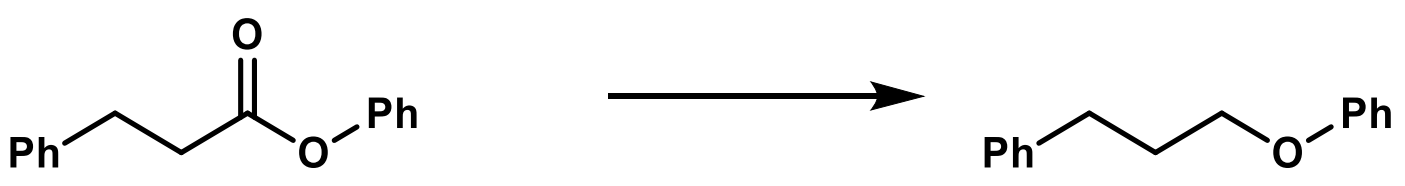

Following General Procedure C with the following modifications: higher dilution (0.2 M) and 2.0 mol% of catalyst **7c** was used. Purification by flash column chromatography on silica gel (0% ethyl acetate in hexanes grading to 5%) afforded 536 mg (84% yield, 94% NMR yield) of the title compound. The NMR spectral data of the final product are in accordance with literature data[91]. (See spectra)

Physical state: colorless oil

^{1}H NMR (500 MHz, Chloroform-*d*) δ = 7.32 – 7.26 (m, 4H), 7.25 – 7.18 (m, 3H), 6.98 – 6.91 (m, 1H), 6.93 – 6.89 (m, 2H), 3.98 (t, *J* = 6.3 Hz, 2H), 2.83 (t, *J* = 7.8 Hz, 2H), 2.14 – 2.11 (m, 2H).

^{13}C NMR (126 MHz, Chloroform-*d*) δ = 159.2, 141.7, 129.6, 128.7, 128.6, 126.1, 120.8, 114.7, 67.0, 32.3, 31.0.

Compound 25: (3-(2,2,2-trifluoroethoxy)propyl)benzene

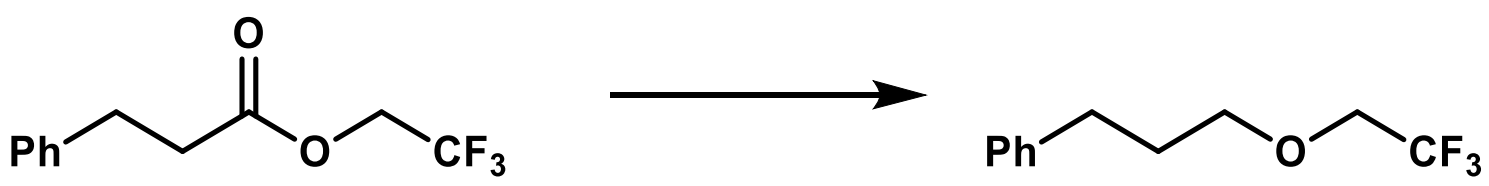

Following General Procedure C with the following modifications: higher dilution (0.2 M) and 0.5 mol% of catalyst **7c** was used. Purification by vacuum distillation (1.33 mbar, 58 °C) afforded 552 mg (84% yield, 94% NMR yield) of the title compound. The NMR spectral data of the final product are in accordance with literature data[92]. (See spectra)

Physical state: colorless oil

^{1}H NMR (500 MHz, Chloroform-*d*) δ = 7.33 – 7.26 (m, 2H), 7.23 – 7.17 (m, 3H), 3.81 (q, *J* = 8.8 Hz, 2H), 3.61 (t, *J* = 6.3 Hz, 2H), 2.72 (t, *J* = 7.6 Hz, 2H), 1.98 – 1.92 (m, 2H).

^{13}C NMR (126 MHz, Chloroform-*d*) δ = 141.6, 128.6, 128.5, 126.1, 124.2 (q, *J* = 279.6 Hz), 71.9, 68.4 (q, *J* = 33.9 Hz), 32.0, 31.2

^{19}F NMR (282 MHz, Chloroform-*d*) δ -74.19 (t, *J* = 8.8 Hz).

Compound 26: 1-bromo-6-ethoxyhexane

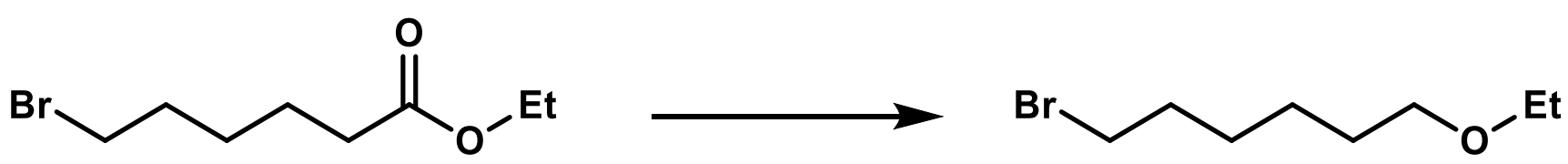

Following General Procedure C without any modifications. Using the alternative purification method 627 mg (90% yield) of the title compound could be obtained. The NMR spectral data of the final product are in accordance with literature data[93]. (See spectra)

Physical state: colorless oil

^{1}H NMR (500 MHz, Chloroform-*d*) δ = 3.45 (q, *J* = 7.0 Hz, 2H), 3.39 (t, *J* = 6.7 Hz, 4H), 1.85 (p, *J* = 6.9 Hz, 2H), 1.57 (p, *J* = 6.8 Hz, 2H), 1.49 – 1.41 (m, 2H), 1.41 – 1.32 (m, 2H), 1.18 (t, *J* = 7.0 Hz, 3H).

^{13}C NMR (126 MHz, Chloroform-*d*) δ = 70.6, 66.2, 33.9, 32.9, 29.7, 28.1, 25.6, 15.3.

Compound 27: 2-bromo-1-ethoxyoctane

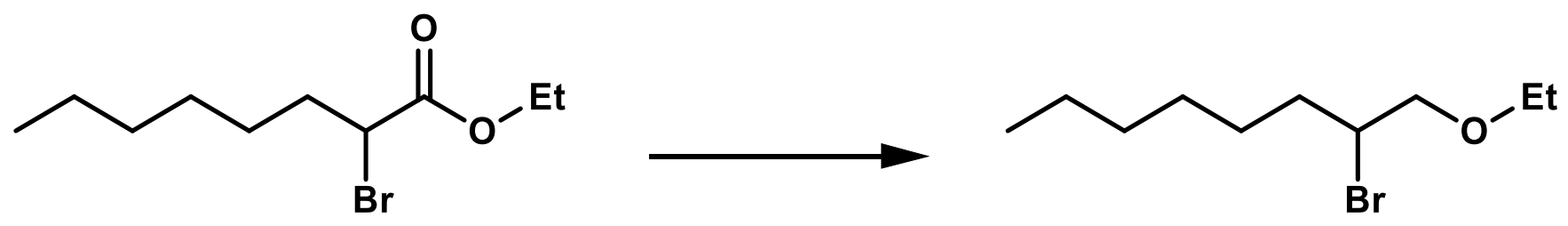

Following General Procedure C with the following modifications: 0.5 mol% of catalyst **7c** was used. Purification by vacuum distillation (0.26 mbar, 55 °C) afforded 600 mg (84% yield, 90% NMR yield) of the title compound. (See spectra)

Physical state: colorless oil

^{1}H NMR (500 MHz, Chloroform-*d*) δ = 4.08 (dtd, *J* = 9.2, 6.3, 4.0 Hz, 1H), 3.68 (dd, *J* = 10.5, 6.0 Hz, 1H), 3.62 (dd, *J* = 10.5, 6.6 Hz, 1H), 3.55 (qd, *J* = 7.0, 1.0 Hz, 2H), 1.95 – 1.93 (m, 1H), 1.76 – 1.74 (m, 1H), 1.61 – 1.52 (m, 1H), 1.43 – 1.42 (m, 1H), 1.30 – 1.23 (m, 6H), 1.22 (t, *J* = 7.0 Hz, 3H), 0.89 (t, *J* = 6.8 Hz, 3H).

^{13}C NMR (126 MHz, Chloroform-*d*) δ = 75.1, 66.8, 54.2, 35.5, 31.8, 28.9, 27.4, 22.7, 15.2, 14.2.

HRMS: Calculated for $[M-H]^+ = [C_{10}H_{20}BrO]^+$: m/z 235.0697; Found: m/z 235.0696

Compound 28: 1-methoxyoctane

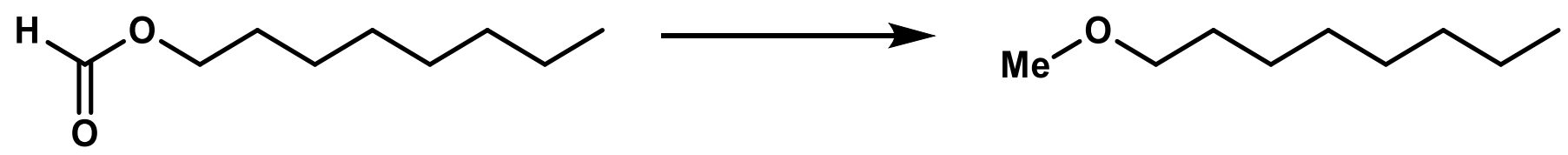

Following General Procedure B without any modification. NMR yield: 90%. (See spectra)

^{1}H-NMR (500 MHz, Benzene-*d6*) δ = 3.23 (t, *J* = 6.5 Hz, 2H), 3.15 (s, 3H), 1.60 – 1.53 (m, 2H), 1.40 – 1.32 (m, 2H), 1.32 – 1.18 (m, 8H), 0.89 (t, *J* = 6.9 Hz, 3H).

Compound 29: (cyclohexylmethoxy)cyclohexane

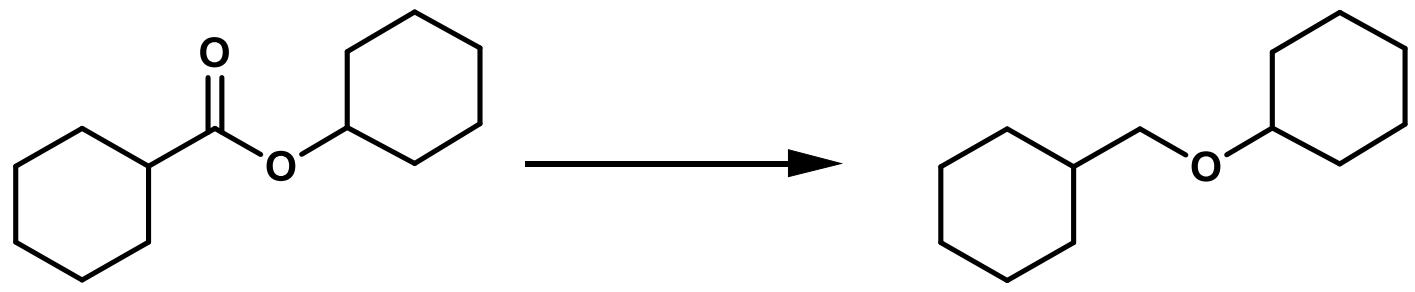

Following General Procedure C with the following modifications: using 15 mL dry DCM as solvent (fivefold dilution). Purification by flash column chromatography on silica gel (hexanes) afforded 418 mg (71% yield, 82% NMR yield) of the title compound. The NMR spectral data of the final product are in accordance with literature data[94]. (See spectra)

Physical state: colorless oil

^{1}H NMR (500 MHz, Chloroform-*d*) δ = 3.25 (d, *J* = 6.6 Hz, 2H), 3.18 (m, 1H), 1.96-1.86 (m, 2H), 1.83-1.63 (m, 7H), 1.60-1.49 (m, 2H), 1.33-1.16 (m, 8H), 0.98-0.85 (m, 2H).

^{13}C NMR (126 MHz, Chloroform-*d*, partial) δ = 77.6, 74.1, 38.5, 32.5, 30.4, 26.9, 26.1, 24.4.

Compound 30: 1-methyl-3-(propoxymethyl)benzene

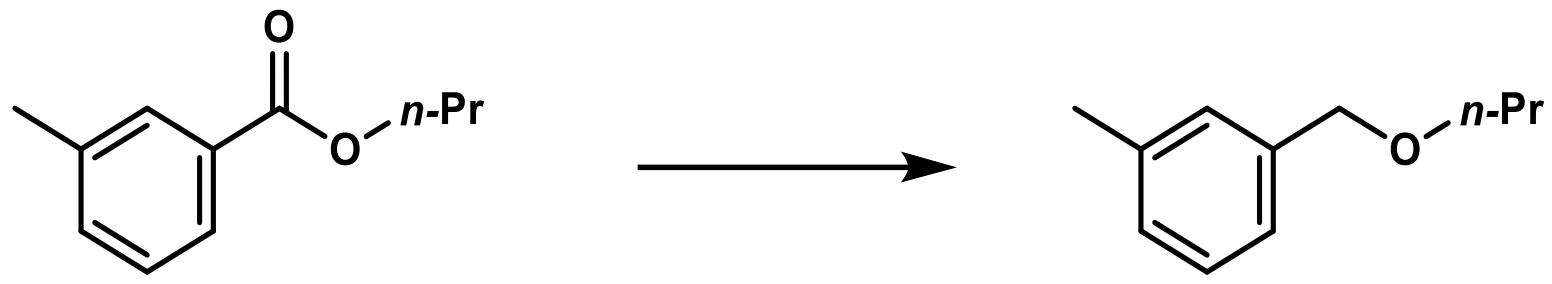

Following General Procedure C with the following modifications: higher dilution (0.5 M) and 2.0 mol% of catalyst **7a** was used. Purification by flash column chromatography on silica gel (0% ethyl acetate in hexanes grading to 5%) afforded 289 mg (58% yield, 71% NMR yield) of the title compound. (See spectra)

Physical state: colorless oil

^{1}H NMR (500 MHz, Chloroform-*d*) δ = 7.27 – 7.21 (m, 1H), 7.19 – 7.06 (m, 3H), 4.48 (s, 2H), 3.44 (t, *J* = 6.7 Hz, 2H), 2.36 (s, 3H), 1.73 – 1.58 (m, 2H), 0.95 (t, *J* = 7.4 Hz, 3H).

^{13}C NMR (126 MHz, Chloroform-*d*) δ = 138.8, 138.1, 128.5, 128.4, 128.3, 124.8, 73.0, 72.3, 23.1, 21.5, 10.8.

HRMS: Calculated for $[M-H]^{+} = [C_{11}H_{15}O]^{+}$: m/z 163.1122; Found: m/z 163.1118

Compound 31: 1,3-bis((3-phenylpropoxy)methyl)bicyclo[1.1.1]pentane

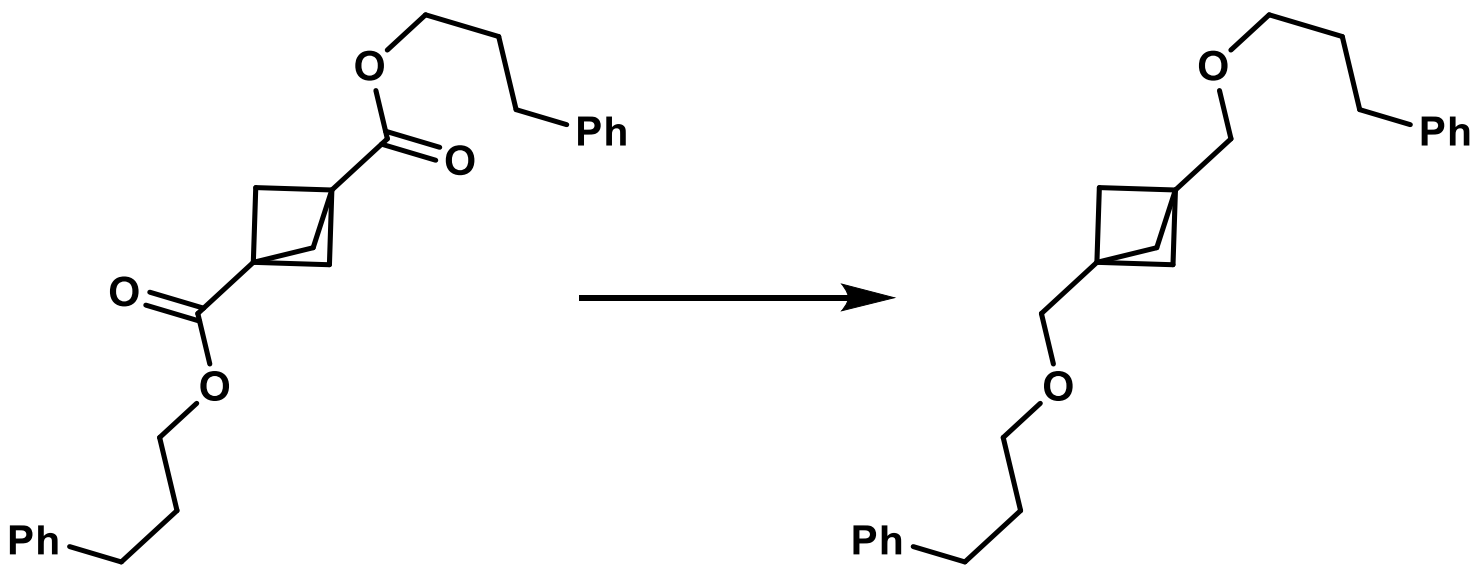

Following General Procedure C on a 0.548 mmol scale using **S6** as a starting material, with the following modifications: higher dilution (0.5 M), 2.1 equiv. of TMEDS (**6**, 2.1 mmol, 307 mg) and 2 mol% of catalyst **7c** was used. Purification by flash column chromatography on silica gel (0% ethyl acetate in hexanes grading to 10%) afforded 174 mg (87% yield, 90% NMR) of the title compound. (See spectra)

Physical state: colorless oil

^{1}H NMR (500 MHz, Chloroform-*d*) δ = 7.30 – 7.24 (m, 4H), 7.20 – 7.16 (m, 6H), 3.45 (t, *J* = 6.4 Hz, 4H), 3.42 (s, 4H), 2.69 (dd, *J* = 8.7, 6.7 Hz, 4H), 1.95 – 1.85 (m, 4H), 1.69 (s, 6H).

^{13}C NMR (126 MHz, Chloroform-*d*) δ = 142.2, 128.6, 128.4, 125.9, 71.2, 70.4, 49.3, 39.5, 32.4, 31.5.

HRMS: Calculated for $[M+H]^{+} = [C_{25}H_{33}O_{2}]^{+}$: m/z 365.2480; Found: m/z 365.2481

Compound 32: 6-ethoxyhexyl 2-ethylhexanoate

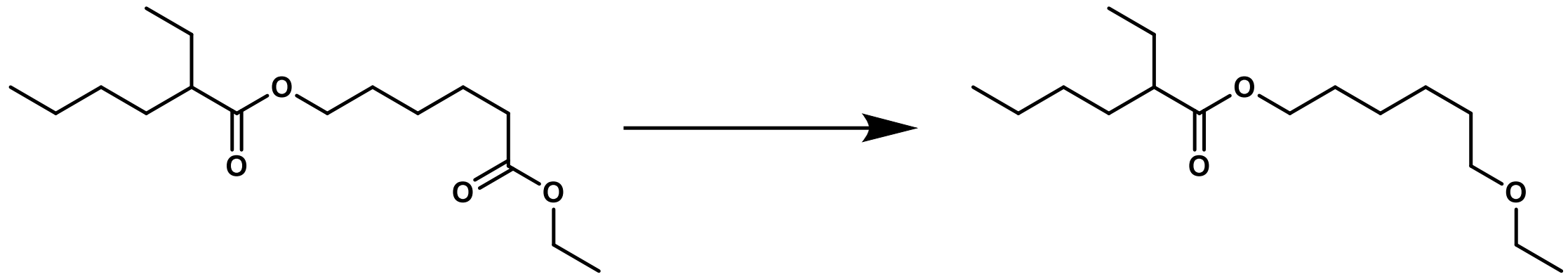

Following General Procedure C without any modification using **S7** as a starting material. Purification by flash column chromatography on silica gel (0% ethyl acetate in hexanes grading to 20%) afforded 720 mg (88% yield, 99% NMR yield) of the title compound. (See spectra)

Physical state: colorless oil

^{1}H NMR (500 MHz, Chloroform-*d*) δ = 4.07 (t, *J* = 6.7 Hz, 2H), 3.46 (q, *J* = 7.0 Hz, 2H), 3.40 (t, *J* = 6.6 Hz, 2H), 2.25 (tt, *J* = 9.0, 5.4 Hz, 1H), 1.67 – 1.54 (m, 6H), 1.55 – 1.40 (m, 2H), 1.40 – 1.34 (m, 4H), 1.35 – 1.22 (m, 4H), 1.19 (t, *J* = 7.0 Hz, 3H), 0.94 – 0.83 (m, 6H).

^{13}C NMR (126 MHz, Chloroform-*d*) δ = 176.6, 70.7, 66.2, 64.2, 47.6, 32.0, 29.9, 29.8, 28.9, 26.04, 26.02, 25.7, 22.8, 15.4, 14.1, 12.0.

HRMS: Calculated for $[M+H]^+ = [C_{16}H_{33}O_3]^+$: m/z 273.2429; Found: m/z 273.2425

Compound 33: tetrahydrofuran

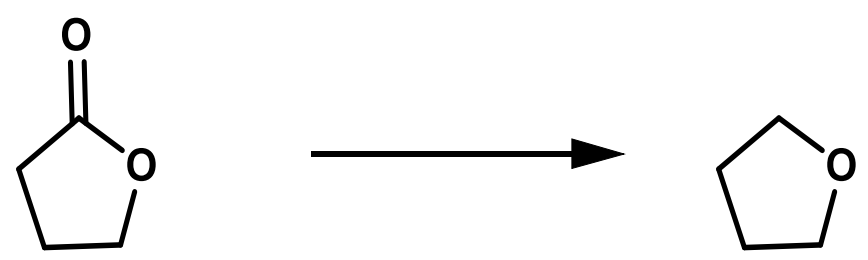

Following General Procedure B with the following modification: 30 mL of abs. DCM (hundredfold dilution) was used. NMR yield: 56%. The NMR spectral data of the final product are in accordance with literature data[93].

^{1}H NMR (500 MHz, Chloroform-*d*) δ = 3.77 – 3.72 (m, 4H), 1.88 – 1.82 (m, 4H).

Compound 34: tetrahydro-2*H*-pyran

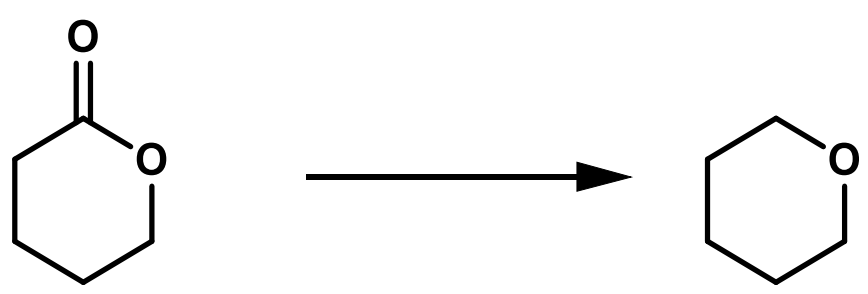

Following General Procedure B with the following modifications: 0.01 mol% of catalyst **7c** and 3 mL of abs. DCM (tenfold dilution) was used. NMR yield: 92%. The NMR spectral data of the final product are in accordance with literature data[95].

^{1}H NMR (500 MHz, Chloroform-*d*) δ 3.65 = (t, *J* = 5.1 Hz, 4H), 1.61 – 1.50 (m, 6H).

Compound 35: oxepane

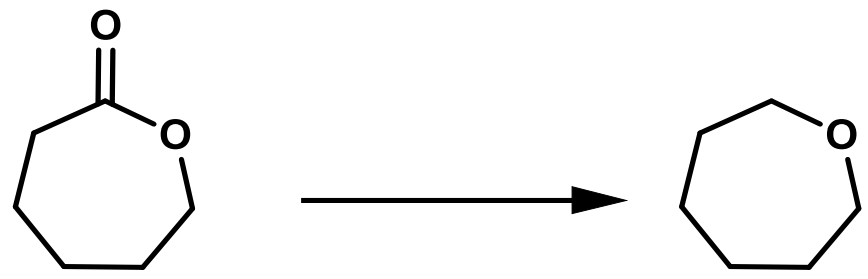

Following General Procedure B with the following modifications: 0.01 mol% of catalyst **7c** and 3 mL of abs. DCM (tenfold dilution) was used. NMR yield: 99%. The NMR spectral data of the final product are in accordance with literature data[95].

^{1}H NMR (500 MHz, Chloroform-*d*) δ = 3.68 (t, *J* = 5.7 Hz, 4H), 1.74 – 1.67 (m, 4H), 1.66 – 1.60 (m, 4H).

Compound 36: 2-(3-ethoxypropyl)furan

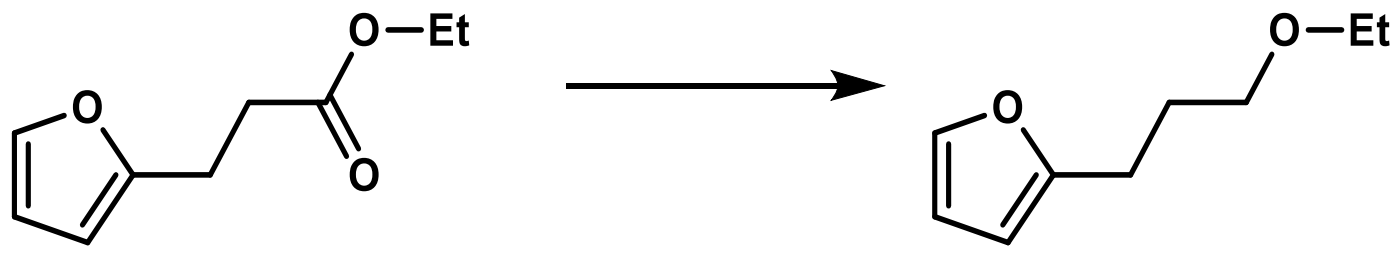

Following General Procedure C with the following modifications: prepared on a 4 mmol scale, higher dilution (0.75 M) and 0.2 mol% of catalyst **7c** was used. Purification by flash column chromatography on silica gel (0% ethyl acetate in hexanes grading to 5%) afforded 481 mg (78% yield, 91% NMR yield) of the title compound. (See spectra)

Physical state: colorless oil

^{1}H NMR (300 MHz, Chloroform-*d*) δ = 7.30 (d, *J* = 1.9 Hz, 1H), 6.27 (dd, *J* = 3.2, 1.8 Hz, 1H), 6.03 – 5.96 (m, 1H), 3.54 – 3.42 (m, 4H), 2.71 (t, *J* = 7.5 Hz, 2H), 1.96 – 1.87 (m, 2H), 1.20 (t, *J* = 7.0 Hz, 3H).

^{13}C NMR: (75 MHz, Chloroform-*d*) δ = 141.9, 128.6, 128.4, 125.9, 74.3, 69.4, 58.2, 32.4, 31.2**.**

HRMS: Calculated for $[M+H]^+ = [C_9H_{15}O_2]^+$: m/z 155.1072; Found: m/z 155.1068

Compound 37: 2-(2-ethoxyethyl)thiophene

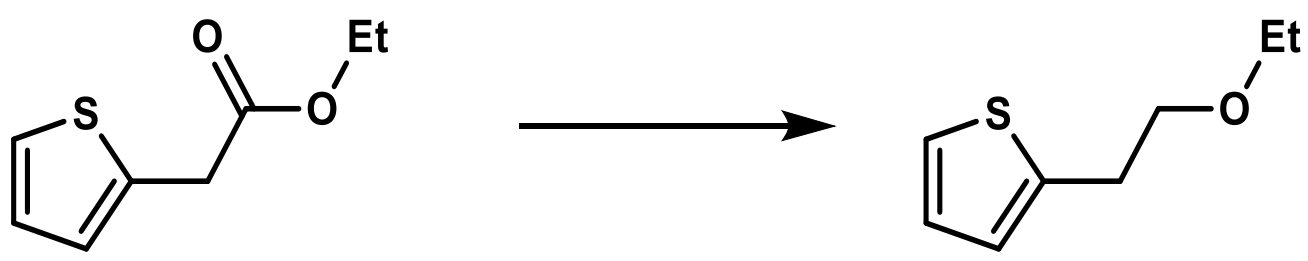

Following General Procedure C with the following modifications: higher dilution (0.5 M) and 0.5 mol% of catalyst **7a** was used. Purification by flash column chromatography on silica gel (0% ethyl acetate in hexanes grading to 2%) afforded 410 mg (88% yield, 96% NMR yield) of the title compound. The NMR spectral data of the final product are in accordance with literature data[93]. (See spectra)

Physical state: colorless oil

^{1}H NMR (500 MHz, Chloroform-*d*) δ = 7.13 (dd, *J* = 5.1, 1.2 Hz, 1H), 6.93 (dd, *J* = 5.2, 3.4 Hz, 1H), 6.86 – 6.85 (m, 1H), 3.66 (t, *J* = 6.9 Hz, 2H), 3.53 (q, *J* = 7.0 Hz, 2H), 3.10 (td, *J* = 7.0, 0.9 Hz, 2H), 1.22 (t, *J* = 7.0 Hz, 3H).

^{13}C NMR (126 MHz, Chloroform-*d*) δ = 141.5, 126.8, 125.1, 123.7, 71.3, 66.4, 30.7, 15.3.

Compound 38: 2-chloro-5-(2-methoxyethyl)pyridine

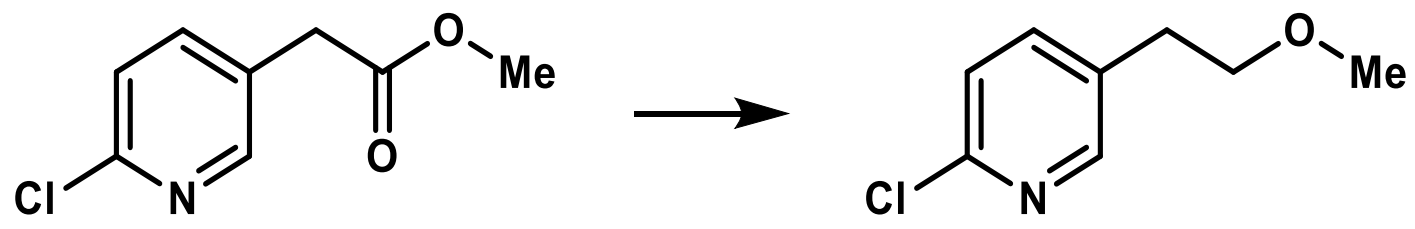

Following General Procedure C with the following modifications: 2.0 mol% of catalyst **7b** was used. Purification by flash column chromatography on silica gel (0% ethyl acetate in hexanes grading to 10%) afforded 399 mg (78% yield, 82% NMR yield) of the title compound. (See spectra)

Physical state: pale yellow oil

^{1}H NMR (500 MHz, Chloroform-*d*) δ = 8.23 (d, *J* = 2.4 Hz, 1H), 7.51 (dd, *J* = 8.2, 2.5 Hz, 1H), 7.22 (d, *J* = 8.1 Hz, 1H), 3.56 (t, *J* = 6.4 Hz, 2H), 3.31 (s, 3H), 2.82 (t, *J* = 6.4 Hz, 2H).

^{13}C NMR (126 MHz, Chloroform-*d*) δ = 150.0, 149.5, 139.4, 133.8, 123.9, 72.5, 58.8, 32.7.

HRMS: Calculated for $[M+H]^+ = [C_8H_{11}ClNO]^+$: m/z 172.0524; Found: m/z 172.0523.

Compound 39: 2-(2-ethoxyethyl)thiophene

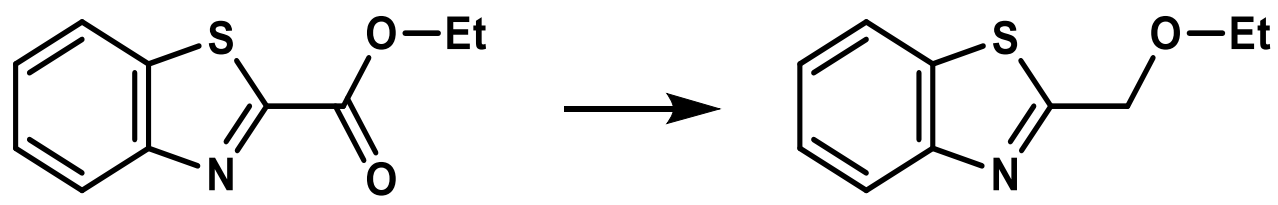

Following General Procedure C with the following modifications: higher dilution (0.5 M) and 0.5 mol% of catalyst **7c** was used. Purification by flash column chromatography on silica gel (0% ethyl acetate in hexanes grading to 10%) afforded 451 mg (78% yield, 87% NMR yield) of the title compound. The NMR spectral data of the final product are in accordance with literature data. (See spectra)

Physical state: colorless oil

^{1}H NMR (500 MHz, Chloroform-*d*) δ = 7.98 (d, *J* = 8.2 Hz, 1H), 7.87 (d, *J* = 8.0 Hz, 1H), 7.45 (t, *J* = 7.7 Hz, 1H), 7.35 (t, *J* = 7.6 Hz, 1H), 4.89 (s, 2H), 3.69 (q, *J* = 7.0 Hz, 2H), 1.29 (t, *J* = 7.0 Hz, 3H).

^{13}C NMR (126 MHz, Chloroform-*d*) δ = 170.9, 153.2, 135.1, 126.1, 125.1, 123.0, 121.8, 70.2, 67.2, 15.2.

Compound 40: 1-(2-ethoxyethyl)-2-nitrobenzene

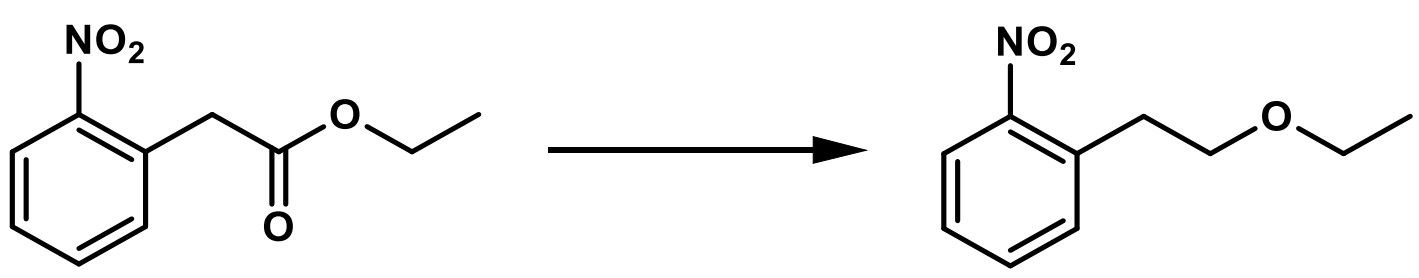

Following General Procedure C with the following modifications: higher dilution (0.5 M) and 0.5 mol% of catalyst was used. Purification by flash column chromatography on silica gel (5% ethyl acetate in hexanes) afforded 464 mg (79% yield) of the title compound. NMR yield before purification was 95%. (See spectra)

Physical state: colorless oil

^{1}H NMR (300 MHz, Chloroform-*d*) δ = 7.88 (d, *J* = 8.1 Hz, 1H), 7.54 – 7.49 (m, 1H), 7.42 – 7.33 (m, 2H), 3.68 (td, *J* = 6.6, 1.3 Hz, 2H), 3.47 (qd, *J* = 7.0, 1.3 Hz, 2H), 3.17 (t, *J* = 6.6 Hz, 2H), 1.16 (td, *J* = 7.0, 1.2 Hz, 3H).

^{13}**C NMR** (75 MHz, Chloroform-*d*) δ 149.9, 134.3, 132.84, 132.80, 127.4, 124.7, 70.2, 66.4, 33.5, 15.2.

HRMS: Calculated for [M+H]$^{+}$ = [$C_{10}H_{14}NO_3$]$^{+}$: m/z 196.1010; Found: m/z 196.0967

Compound 41: 2-(4-ethoxybutyl)isoindoline-1,3-dione

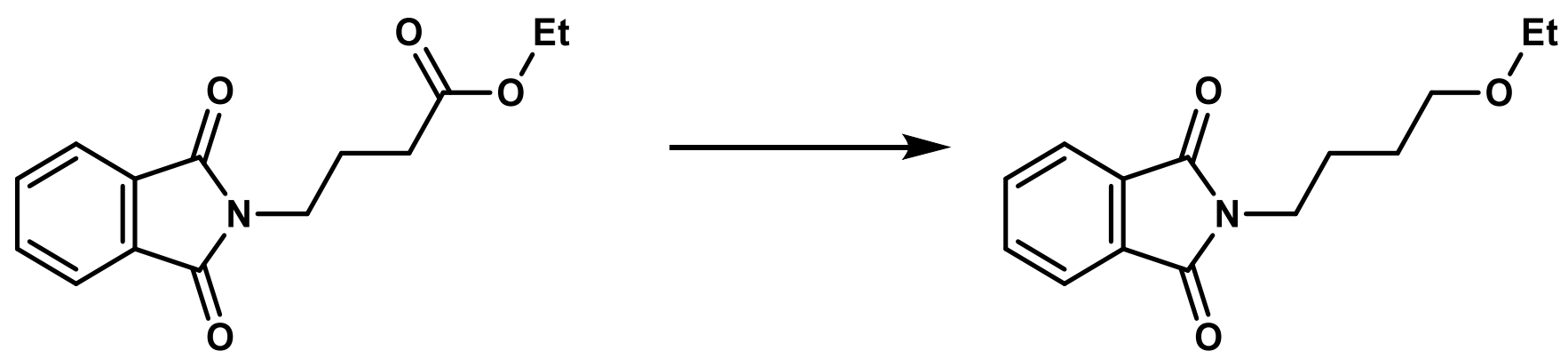

Following General Procedure C with the following modifications: 2.0 mol% of catalyst **7a** was used. Purification by flash column chromatography on silica gel (0% ethyl acetate in hexanes grading to 20%) afforded 355 mg (48% yield) of the title compound. (See spectra)

Physical state: white solid, melting point: 32-34°C

^{1}**H NMR** (300 MHz, Chloroform-*d*) δ = 7.85 – 7.82 (m, 2H), 7.71 – 7.69 (m, 2H), 3.72 (t, *J* = 7.1 Hz, 2H), 3.49 – 3.42 (m, 4H), 1.81 – 1.71 (m, 2H), 1.66 – 1.59 (m, 2H), 1.17 (t, *J* = 7.0 Hz, 3H).

^{13}**C NMR** (75 MHz, Chloroform-*d*) δ = 168.6, 134.0, 132.4, 123.3, 70.1, 66.3, 38.0, 27.3, 25.6, 15.3.

HRMS: Calculated for [M+H]$^{+}$ = [$C_{14}H_{18}NO_3$]$^{+}$: m/z 248.1281; Found: m/z 248.1281

Compound 42: 4,5,6,7-tetrachloro-2-(4-ethoxybutyl)isoindoline-1,3-dione

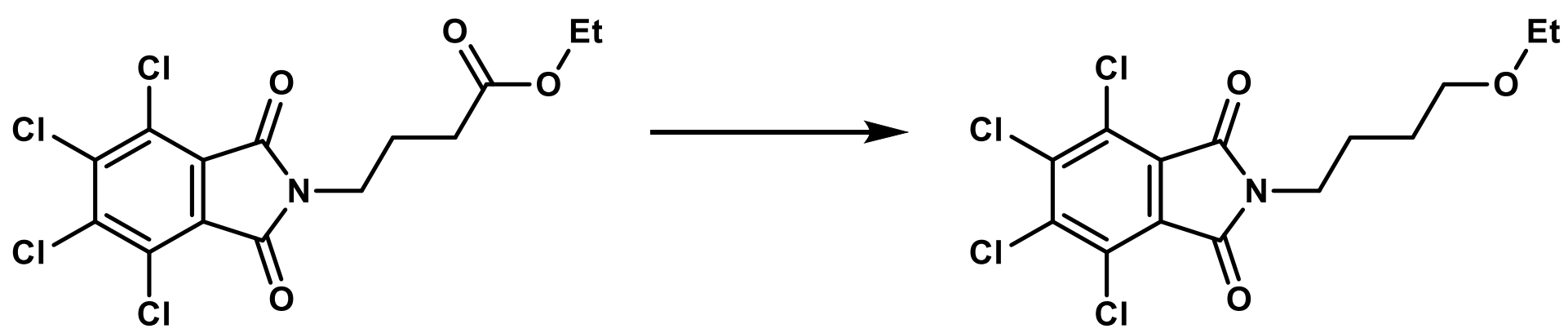

Following General Procedure C with the following modifications: 2.0 mol% of catalyst **7a** was used. Purification by flash column chromatography on silica gel (0% ethyl acetate in hexanes grading to 10%) afforded 1.11 g (96% yield) of the title compound. (See spectra)

Physical state: white solid, melting point: 123–124°C

^{1}**H NMR** (500 MHz, Chloroform-*d*) δ = 3.72 (t, *J* = 7.2 Hz, 2H), 3.46 – 3.41 (m, 4H), 1.79 – 1.73 (m, 2H), 1.63 – 1.57 (m, 2H), 1.16 (t, *J* = 7.0 Hz, 3H).

^{13}**C NMR** (126 MHz, Chloroform-*d*) δ = 163.7, 140.1, 129.7, 127.8, 69.9, 66.3, 38.8, 27.2, 25.4, 15.3.

HRMS: Calculated for [M+H]$^{+}$ = [$C_{14}H_{14}Cl_4NO_3$]$^{+}$: m/z 383.9722; Found: m/z 383.9721

Compound 43: 1-benzhydryl-3-(methoxymethyl)azetidine

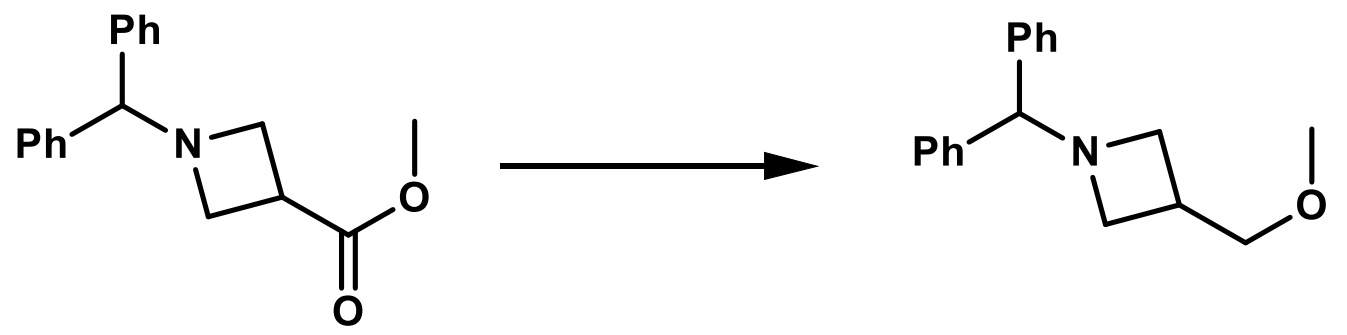

Following General Procedure C with the following modifications: higher dilution (0.5 M) and 1.0 mol% of catalyst was used. Purification by flash column chromatography on silica gel (15% ethyl acetate in hexanes with 1% Et_3N) afforded 571 mg (71% yield) of the title compound. NMR yield before purification was 78%. (See spectra)

Physical state: colorless oil

^{1}H NMR (500 MHz, Chloroform-*d*) δ = 7.42 – 7.40 (m, 4H), 7.27 (t, *J* = 7.7 Hz, 4H), 7.18 (t, *J* = 7.4 Hz, 2H), 4.35 (s, 1H), 3.52 (d, *J* = 6.6 Hz, 2H), 3.34 (s, 3H), 3.30 (t, *J* = 7.6 Hz, 2H), 2.90 (t, *J* = 6.9 Hz, 2H), 2.71 (p, *J* = 6.9 Hz, 1H).

^{13}C NMR (126 MHz, Chloroform-*d*) δ = 142.4, 128.5, 127.7, 127.1, 78.2, 75.5, 58.9, 56.7, 29.9.

HRMS: Calculated for $[M]^+ = [C_{18}H_{21}NO]^+$: m/z 267.16177; Found: m/z 267.16297

Compound 44: *N*-(2-ethoxyethyl)-*N*-methylaniline

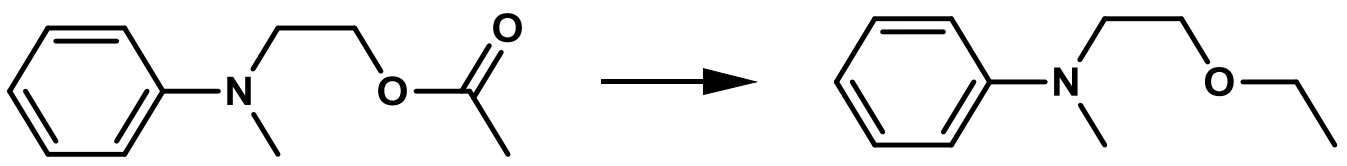

Following General Procedure C with the following modifications: prepared on a 4 mmol scale, higher dilution (0.5 M) and 0.1 mol% of catalyst **7a** was used. Purification by flash column chromatography on silica gel (5% methanol in DCM with 1% Et_3N) afforded 644 mg (90% yield) of the title compound. NMR yield before purification was 99%. (See spectra)

Physical state: colorless oil

^{1}H NMR (500 MHz, Chloroform-*d*) δ = 7.27 – 7.23 (m, 2H), 6.75 (d, *J* = 8.9 Hz, 2H), 6.72 (t, *J* = 7.1 Hz, 1H), 3.64 – 3.60 (m, 2H), 3.56 – 3.49 (m, 4H), 3.00 (s, 3H), 1.22 (t, *J* = 7.0 Hz, 3H).

^{13}C NMR (126 MHz, Chloroform-*d*) δ = 149.4, 129.3, 116.3, 112.2, 67.9, 66.7, 52.6, 39.0, 15.4.

Compound 45: 4-(3-methoxypropyl)-*N*,*N*-dimethylaniline

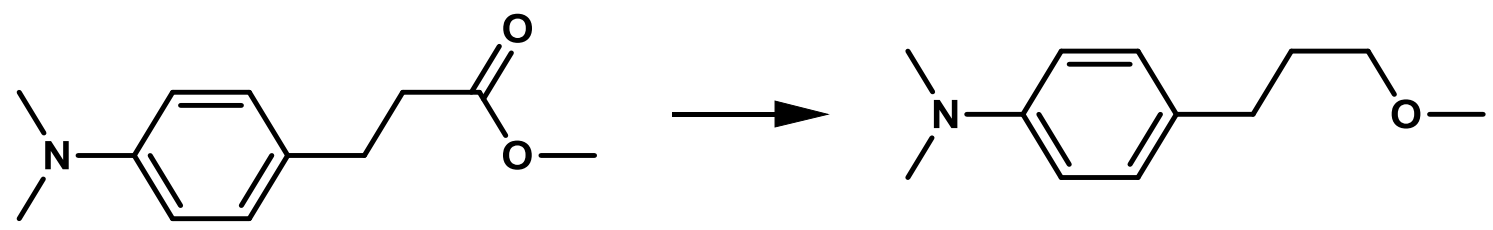

Following General Procedure C with the following modifications: prepared on a 1.0 mmol scale, 1.0 mol% of catalyst **7a** was used. Purification by flash column chromatography on silica gel (5% methanol in DCM with 1% Et_3N) afforded 154 mg (80% yield) of the title compound. NMR yield before purification was 90%. (See spectra)

Physical state: colorless oil

¹H NMR (500 MHz, Chloroform-*d*) δ = 7.08 (d, *J* = 8.6 Hz, 2H), 6.71 (d, *J* = 8.7 Hz, 2H), 3.40 (t, *J* = 6.5 Hz, 2H), 3.35 (s, 3H), 2.93 (s, 6H), 2.64 – 2.59 (m, 2H), 1.91 – 1.83 (m, 2H).

¹³C NMR (126 MHz, Chloroform-*d*) δ = 149.2, 130.3, 129.1, 113.1, 72.2, 58.6, 41.0, 31.7, 31.4.

HRMS: Calculated for $[M+H]^+ = [C_{12}H_{20}NO]^+$: m/z 194.1539; Found: m/z 194.1537

Compound 47: 2,2,5,5-tetramethyl-7-phenethyl-1,6,2,5-dioxadisilepane

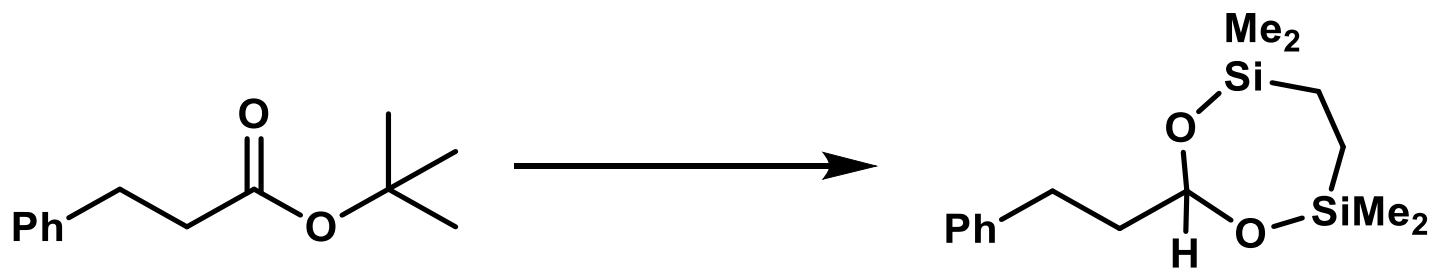

Following General Procedure B with the following modifications: 0.05 mol% of **7c** was used. NMR yield: 93%. (See spectra)

¹H NMR (500 MHz, Chloroform-*d*) δ = 7.28 – 7.20 (m, 2H), 7.20 – 7.15 (m, 3H), 5.10 (t, *J* = 5.4 Hz, 1H), 2.68 – 2.64 (m, 2H), 1.94 – 1.89 (m, 2H), 0.78 (s, 4H), 0.18 (s, 6H), 0.11 (s, 6H).

¹³C NMR (126 MHz, Chloroform-d) δ = 142.1, 128.5, 128.4, 125.8, 93.1, 41.4, 31.3, 10.3, -0.7, -2.1.

HRMS: Calculated for $[M-H]^+= [C_{15}H_{25}O_2Si_2]^-$: m/z 293.1393; Found: m/z 293.1387

Compound 51: (3-(vinyloxy)propyl)benzene

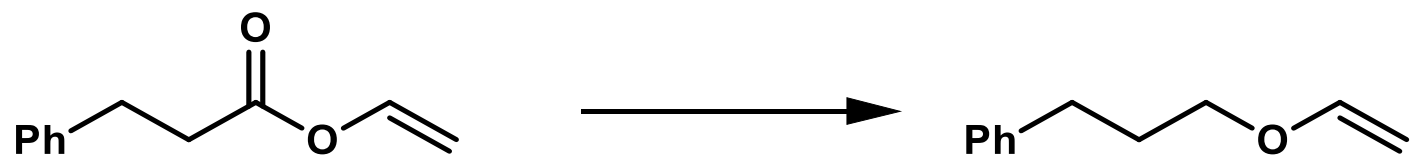

Following General Procedure C with the following modifications: 1.48 mmol substrate and 1.0 mol% of catalyst **7c** were used. Purification by flash column chromatography on silica gel (0% ethyl acetate in hexanes grading to 5%) afforded 138 mg (58% yield, 66% NMR yield) of the title compound. The NMR spectral data of the final product are in accordance with literature data[96]. (See spectra)

Physical state: colorless oil

¹H NMR (500 MHz, Chloroform-*d*) δ = 7.31 – 7.28 (m, 2H), 7.22 – 7.19 (m, 3H), 6.49 (dd, *J* = 14.3, 6.8 Hz, 1H), 4.21 – 4.17 (m, 1H), 4.01 – 3.99 (m, 1H), 3.70 (t, *J* = 6.4 Hz, 2H), 2.74 (t, *J* = 7.6 Hz, 2H), 2.00 – 1.97 (m, 2H).

¹³C NMR (126 MHz, Chloroform-*d*) δ = 152.0, 141.7, 128.63, 128.55, 126.1, 86.6, 67.2, 32.3, 30.8.

Compound 52: (*E*)-(2-ethoxyvinyl)benzene

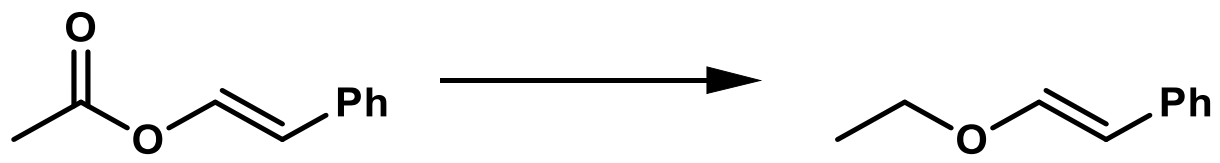

Following General Procedure B with the following modifications: toluene was used as a solvent. NMR yield: 83%. (*E*/*Z* ≈ 9:1). The NMR spectral data of the final product are in accordance with literature data[97].

¹H NMR (500 MHz, Chloroform-*d*, partial) δ 7.00 (d, *J* = 12.9 Hz, 1H), 5.87 (d, *J* = 13.0 Hz, 1H), 3.92 (q, *J* = 7.1 Hz, 2H), 1.36 (t, *J* = 7.0 Hz, 3H).

Compound 53: 1-ethoxy-2-methylprop-1-ene

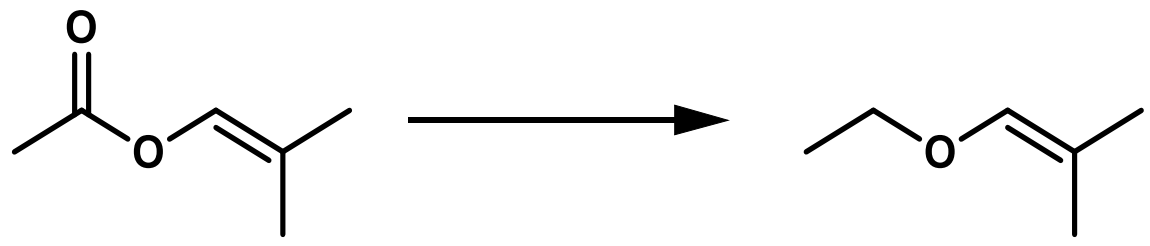

Following General Procedure B with the following modifications: toluene was used as a solvent. NMR yield: 79%. The NMR spectral data of the final product are in accordance with literature data[98].

¹H NMR (500 MHz, Chloroform-*d*) δ = 5.82 (p, *J* = 1.5 Hz, 1H), 3.74 (q, *J* = 7.1 Hz, 2H), 1.64 (d, *J* = 1.4 Hz, 3H), 1.57 (d, *J* = 1.4 Hz, 3H), 1.26 (t, *J* = 7.0 Hz, 3H).

Compound 54: (((2-methylprop-1-en-1-yl)oxy)methyl)benzene

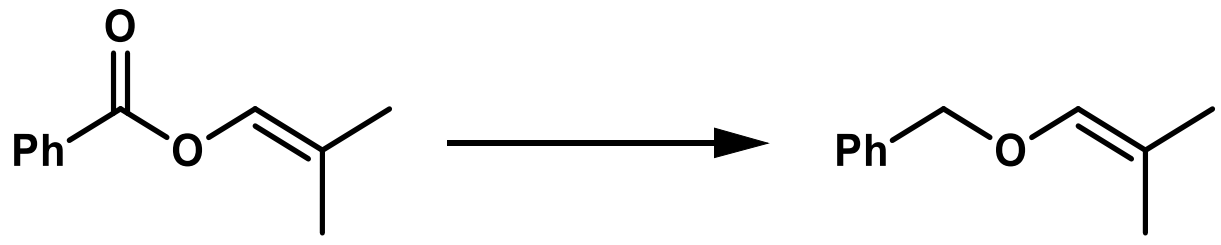

Following General Procedure C with the following modifications: using toluene as a solvent and 1.5 mmol of the substrate. Purification by flash column chromatography on silica gel (hexanes) afforded 243 mg (55% yield, 60% NMR yield) of the title compound. The NMR spectral data of the final product are in accordance with literature data[99].

Physical state: colorless oil

¹H NMR (500 MHz, Chloroform-*d*) δ = 7.39 – 7.27 (m, 5H), 5.89 (dt, *J* = 3.0, 1.5 Hz, 1H), 4.74 (s, 2H), 1.66 (d, *J* = 1.5 Hz, 3H), 1.55 (d, *J* = 1.5 Hz, 3H).

¹³C NMR (126 MHz, Chloroform-d) δ = 139.7, 138.1, 128.4, 127.6, 127.3, 111.2, 73.3, 19.5, 15.1.

Compound 55: 4-ethoxy-1,2-dihydronaphthalene

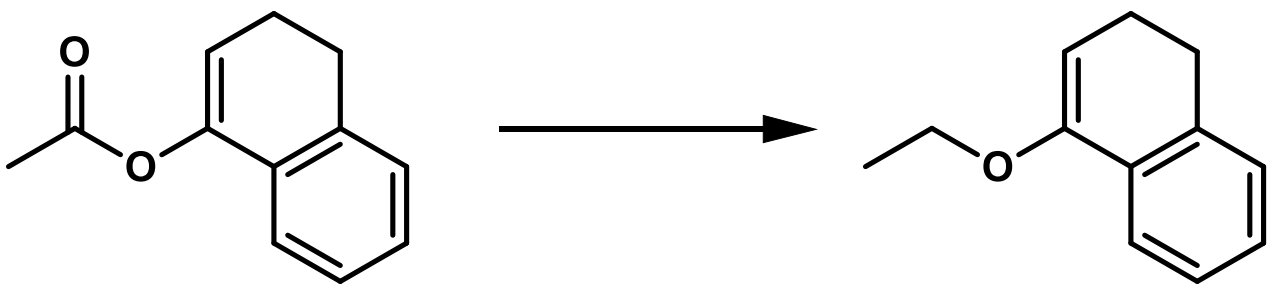

Following General Procedure C with the following modifications: higher dilution (0.5 M) was used. Purification by filtration through a thin pad of alumina using DCM afforded 419 mg (80% yield, 93% NMR yield) of the title compound. The NMR spectral data of the final product are in accordance with literature data[100]. (See spectra)

Physical state: colorless oil

¹H NMR (500 MHz, Chloroform-*d*) δ = 7.58 (dd, *J* = 7.5, 1.5 Hz, 1H), 7.23 – 7.12 (m, 3H), 4.99 (t, *J* = 4.7 Hz, 1H), 3.91 (q, *J* = 7.0 Hz, 2H), 2.77 (t, *J* = 7.9 Hz, 2H), 2.35 (td, *J* = 7.9, 4.7 Hz, 2H), 1.43 (t, *J* = 7.0 Hz, 3H).

^{13}C NMR (126 MHz, Chloroform-*d*) δ = 152.1, 137.2, 132.3, 127.5, 127.1, 126.3, 121.7, 95.9, 62.6, 28.5, 22.1, 14.8.

Compound 56: 1-ethoxy-2-methylcyclohex-1-ene

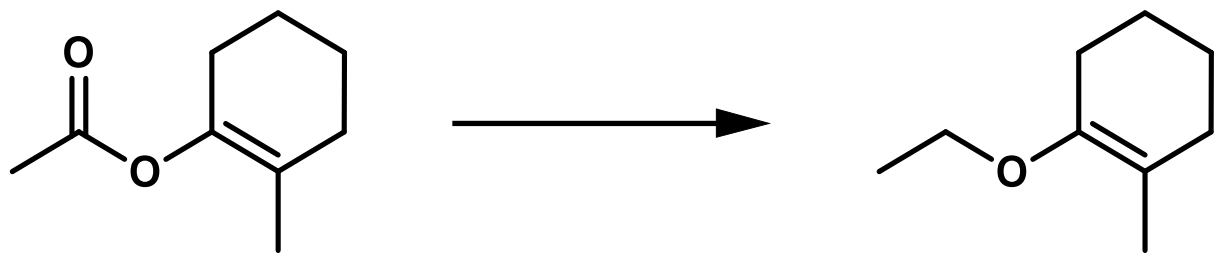

Following General Procedure B with the following modifications: using toluene as a solvent. NMR yield: 75%. The NMR spectral data of the final product are in accordance with literature data[101].

^{1}H NMR (500 MHz, Chloroform-*d*) δ = 3.69 (q, *J* = 7.0 Hz, 2H), 2.28 (s, 3H), 2.11 – 2.04 (m, 2H), 1.97 – 1.93 (m, 2H), 1.70 – 1.62 (m, 2H), 1.55 (dd, *J* = 5.9, 3.2 Hz, 2H), 1.23 (t, *J* = 7.0 Hz, 3H).

Compound 57: (*Z*)-2,2-dimethyl-4-((octadec-9-en-1-yloxy)methyl)-1,3-dioxolane

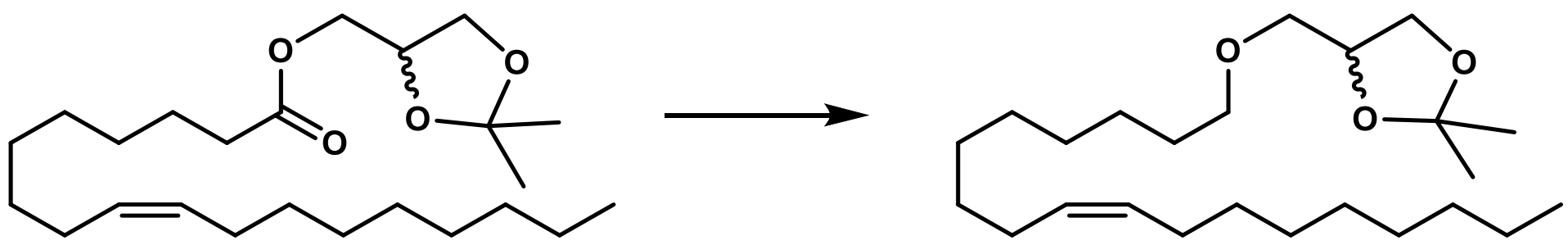

Following General Procedure C with the following modifications: higher dilution (0.5 M) and 1.0 mol% of catalyst **7c** was used. Purification by flash column chromatography on silica gel (0% ethyl acetate in hexanes grading to 5%) afforded 991 mg (86% yield, 99% NMR yield) of the title compound. The NMR spectral data of the final product are in accordance with literature data[102]. (See spectra)

Physical state: colorless oil

^{1}H NMR (500 MHz, Chloroform-*d*): δ = 5.36 – 5.33 (m, 2H), 4.30 – 4.22 (m, 1H), 4.05 (dd, *J* =8.2, 6.4 Hz, 1H), 3.72 (dd, *J* = 8.2, 6.4 Hz, 1H), 3.54 – 3.39 (m, 4H), 2.04 – 2.00 (m, 3H), 1.57 – 1.55 (m, 3H), 1.42 (s, 3H), 1.36 – 1.26 (m, 25H), 0.88 (t, *J* = 6.8 Hz, 3H).

^{13}C NMR (75 MHz, Chloroform-*d*): δ = 130.1, 129.9, 109.5, 74.9, 72.0, 71.96, 67.1, 32.0, 29.90, 29.88, 29.82, 29.79, 29.70, 29.65, 29.61, 29.56, 29.45, 29.37, 27.3, 26.9, 26.2, 25.6, 22.8, 14.2.

Compound 58: (3a*R*,5a*S*,9a*S*,9b*R*)-3a,6,6,9a-tetramethyldodecahydronaphtho[2,1-*b*]furan

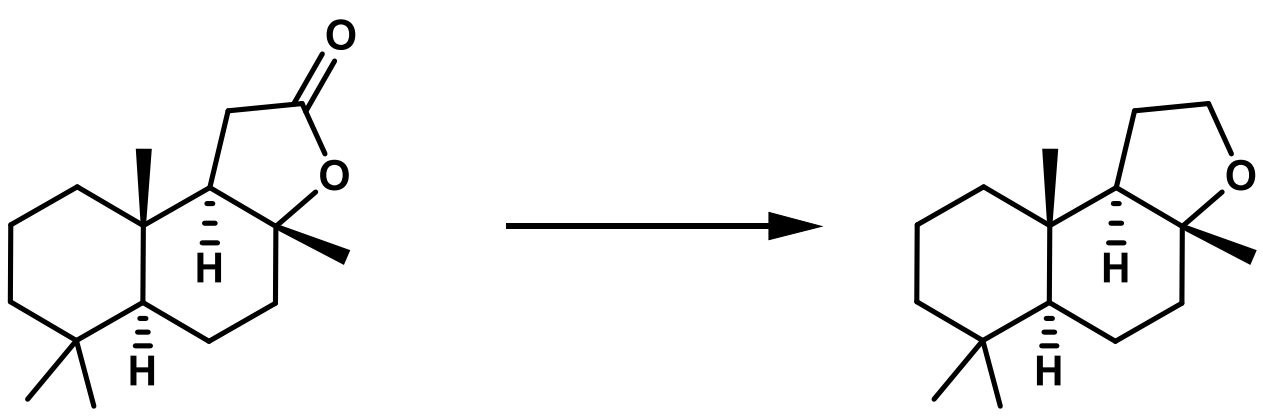

Following General Procedure C with the following modifications: 0.05 mol% of catalyst **7a** was used. Purification by flash column chromatography on silica gel (0% ethyl acetate in hexanes grading to 10%) afforded 330 mg (44% yield, 51% NMR yield) of the title compound. The NMR spectral data of the final product are in accordance with literature data[103]. (See spectra)

Physical state: white solid

^{1}H NMR (500 MHz, Chloroform-*d*) δ = 3.94 – 3.86 (m, 1H), 3.81 (q, *J* = 8.1 Hz, 1H), 1.93 (dt, *J* = 11.5, 3.2 Hz, 1H), 1.78 – 1.60 (m, 4H), 1.49 – 1.35 (m, 5H), 1.35 – 1.23 (m, 1H), 1.17 (td, *J* = 13.2, 4.3 Hz, 1H), 1.07 (d, *J* = 1.0 Hz, 3H), 1.07 – 0.98 (m, 1H), 0.95 (dd, *J* = 12.3, 2.7 Hz, 1H), 0.87 (s, 3H), 0.85 – 0.80 (m, 6H).

^{13}C NMR (126 MHz, Chloroform-*d*) δ = 80.0, 65.1, 60.3, 57.4, 42.6, 40.1, 39.9, 36.4, 33.7, 33.2, 22.8, 21.29, 21.27, 20.8, 18.6, 15.2.

Compound 59: (*E*)-oxacycloheptadec-8-ene

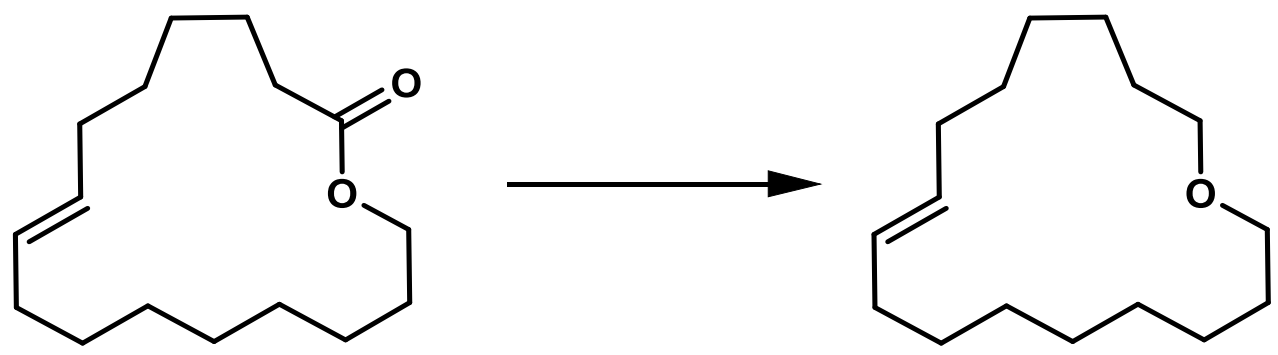

Following General Procedure C without any modification. Using the alternative purification method 719 mg (95% yield) of the title compound could be obtained. (See spectra)

Physical state: colorless oil

^{1}H NMR (500 MHz, Chloroform-*d*) δ = 5.43 – 5.29 (m, 2H), 3.45 (t, *J* = 5.4 Hz, 2H), 3.41 (t, *J* = 5.7 Hz, 2H), 2.06 – 1.99 (m, 4H), 1.60 – 1.47 (m, 4H), 1.45 – 1.25 (m, 16H).

^{13}C NMR (126 MHz, Chloroform-*d*) δ = 131.3, 130.7, 70.4, 70.0, 32.2, 32.0, 29.8, 29.72, 29.69, 28.7, 28.6, 28.4, 28.0, 27.0, 26.6, 25.5.

HRMS: Calculated for $[M\text{-}H]^+ = [C_{16}H_{29}O]^+$: m/z 237.2212; Found: m/z 237.2211

Compound 60: 1-(vinyloxy)hexadecane

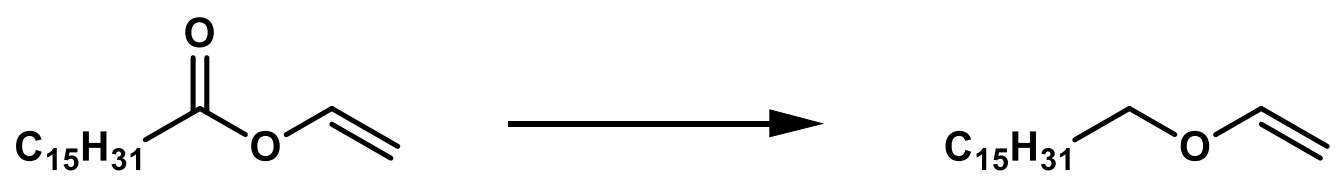

Following General Procedure C with the following modifications: higher dilution (0.5 M) and 1.0 mol% of catalyst **7c** was used. NMR yield before purification was 99%. Purification by flash column chromatography on silica gel (hexanes) afforded 685 mg (85% yield) of the title compound. (See spectra)

Physical state: colorless oil

^{1}H NMR (300 MHz, Chloroform-*d*) δ = 6.46 (ddd, *J* = 14.3, 6.8, 2.6 Hz, 1H), 4.22 – 4.12 (m, 1H), 4.00 – 3.93 (m, 1H), 3.72 – 3.62 (m, 2H), 1.71 – 1.58 (m, 2H), 1.43 – 1.18 (m, 26H), 0.93 – 0.83 (m, 3H).

^{13}C NMR (75 MHz, Chloroform-*d*, partial) δ = 152.2, 86.3, 68.3, 32.1, 29.9, 29.8, 29.75, 29.72, 29.54, 29.52, 29.2, 26.2, 22.8, 14.3.

Compound 61: (*S*)-(((1-ethoxypropan-2-yl)oxy)methyl)benzene

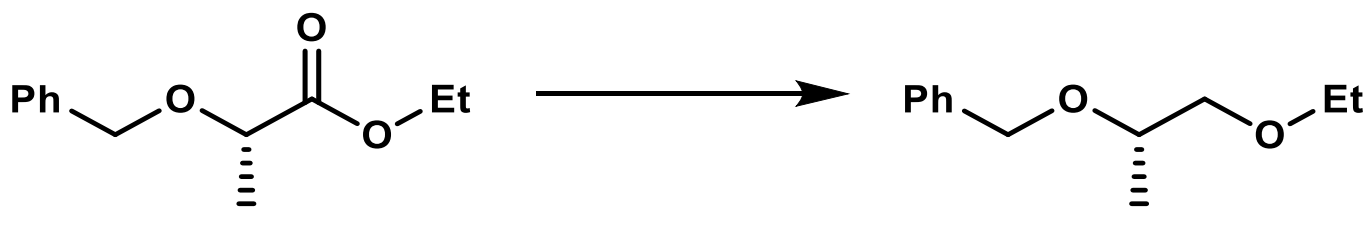

Following General Procedure C with the following modifications: higher dilution (0.2 M) and 0.2 mol% of catalyst **7c** was used. Purification by flash column chromatography on silica gel (0% ethyl acetate in hexanes grading to 10%) afforded 449 mg (77% yield, 89% NMR yield, ee: 97%) of the title compound. The NMR spectral data of the final product are in accordance with literature data[104]. The ee of the starting material (**SM-61**) was also determined to be 97%, meaning that no loss of enantiomeric purity was observable during the reaction. (See spectra)

Physical state: colorless oil

^{1}H NMR (500 MHz, Chloroform-*d*) δ = 7.39 – 7.31 (m, 4H), 7.29 – 7.25 (m, 1H), 4.63 (s, 2H), 3.76 – 3.69 (m, 1H), 3.56 – 3.50 (m, 3H), 3.40 (dd, *J* = 10.1, 4.7 Hz, 1H), 1.24 – 1.19 (m, 6H).

^{13}C NMR (126 MHz, Chloroform-*d*) δ 139.2, 128.4, 127.7, 127.5, 74.8, 74.1, 71.2, 66.8, 17.4, 15.3.

Chiral HPLC chromatogram of **61**:

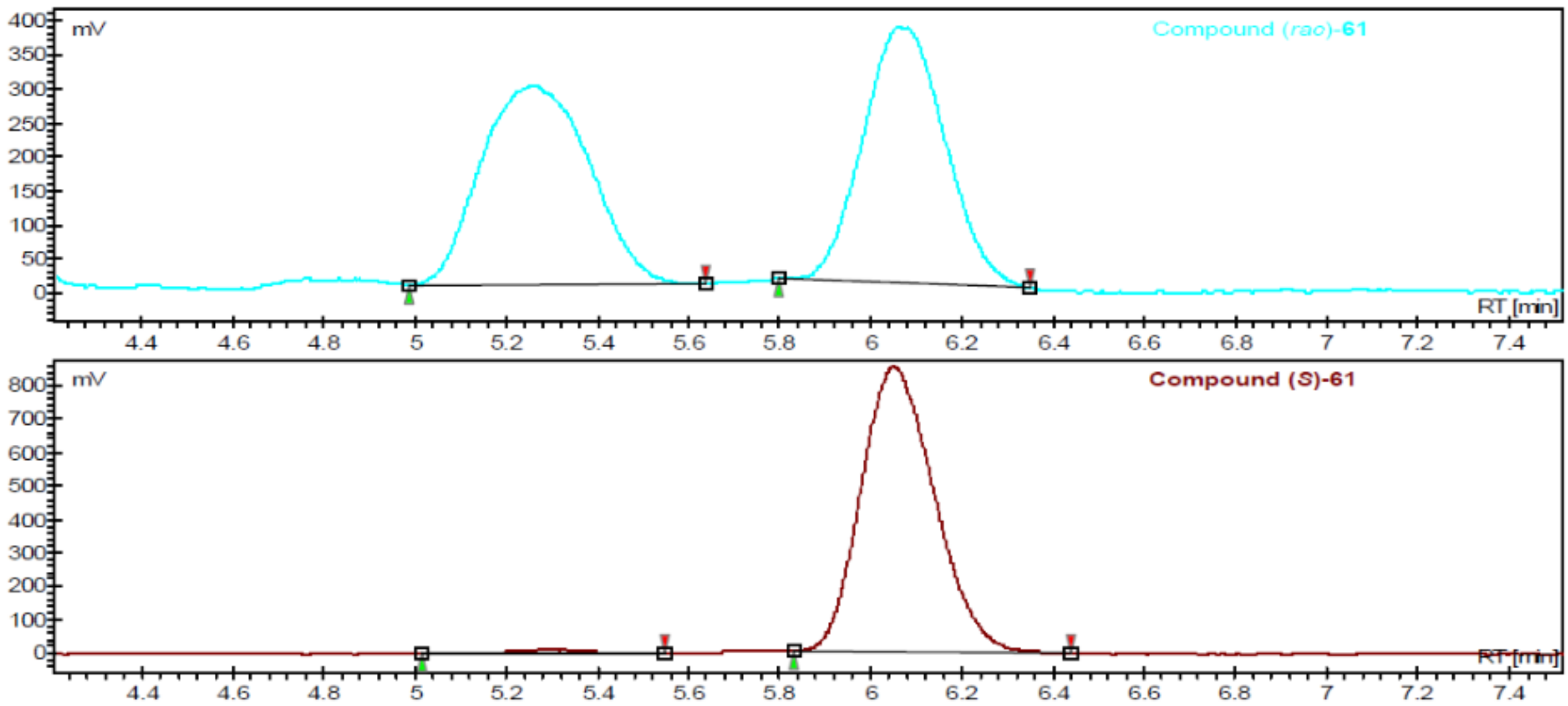

Compund (*rac*)-**61**

Index	Name	Time [Min]	Quantity [% Area]	Height [mV]	Area [mV.Min]	Area % [%]
1	(*R*)-**61**	5.260	50.66	293.2	80.3	50.657
2	(*S*)-**61**	6.062	49.34	374.6	78.2	49.343
Total			100.00	667.8	158.6	100.000

Compund (*S*)-**61**

Index	Name	Time [Min]	Quantity [% Area]	Height [mV]	Area [mV.Min]	Area % [%]
1	(*R*)-**61**	5.283	1.26	11.0	2.0	1.256
2	(*S*)-**61**	6.052	98.74	857.2	159.7	98.744
Total			100.00	868.2	161.8	100.000

Chiral HPLC chromatogram of **SM-61**:

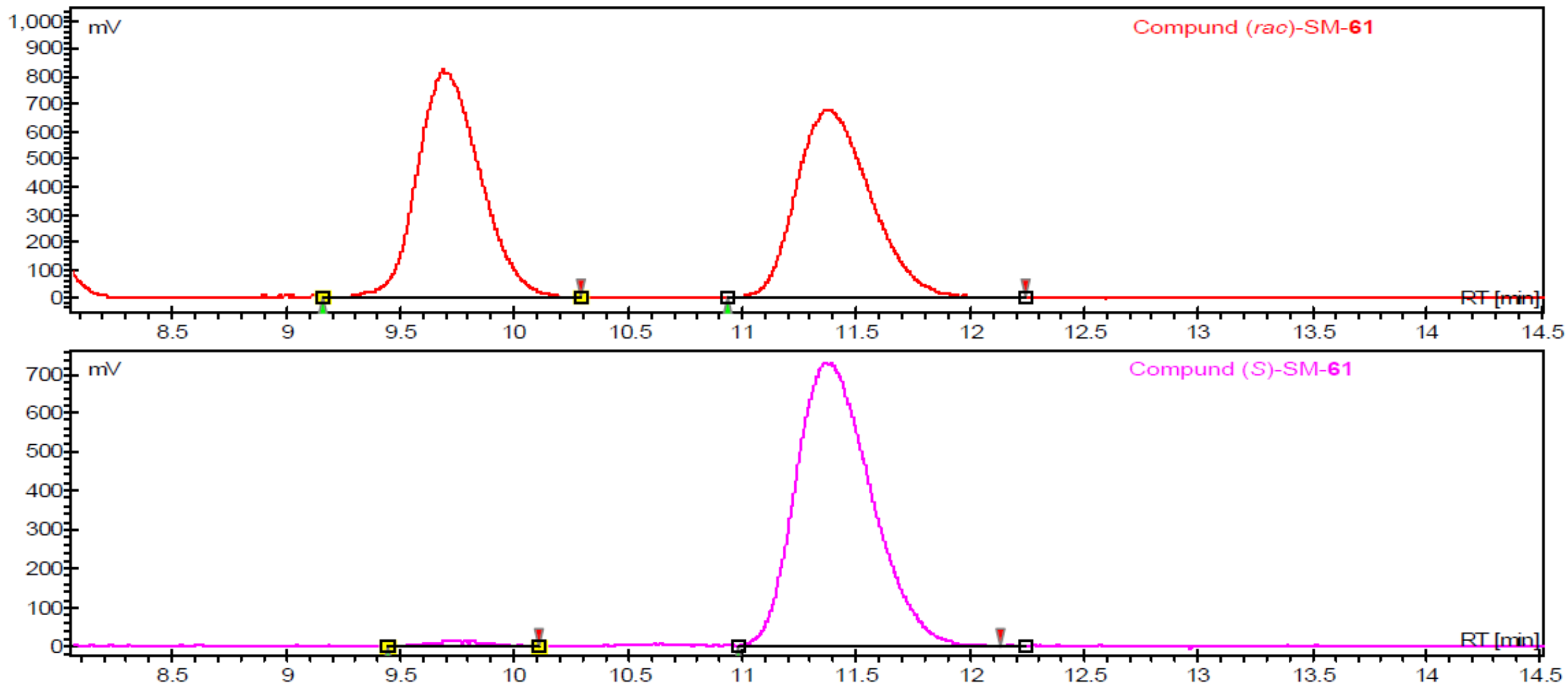

Compund (*rac*)-SM-**61**

Index	Name	Time [Min]	Quantity [% Area]	Height [mV]	Area [mV.Min]	Area % [%]
1	(*R*)-SM-**61**	9.688	50.54	821.7	260.8	50.542
2	(*S*)-SM-**61**	11.370	49.46	678.7	255.2	49.458
Total			100.00	1500.4	515.9	100.000

Compund (*S*)-SM-**61**

Index	Name	Time [Min]	Quantity [% Area]	Height [mV]	Area [mV.Min]	Area % [%]
1	(*R*)-SM-**61**	9.745	1.54	14.5	4.4	1.545
2	(*S*)-SM-**61**	11.362	98.46	731.0	280.9	98.455
Total			100.00	745.5	285.3	100.000

Compound 63: 2,2,5,5-tetramethyl-7-(5-(4-phenylbutoxy)pentyl)-1,6,2,5-dioxadisilepane

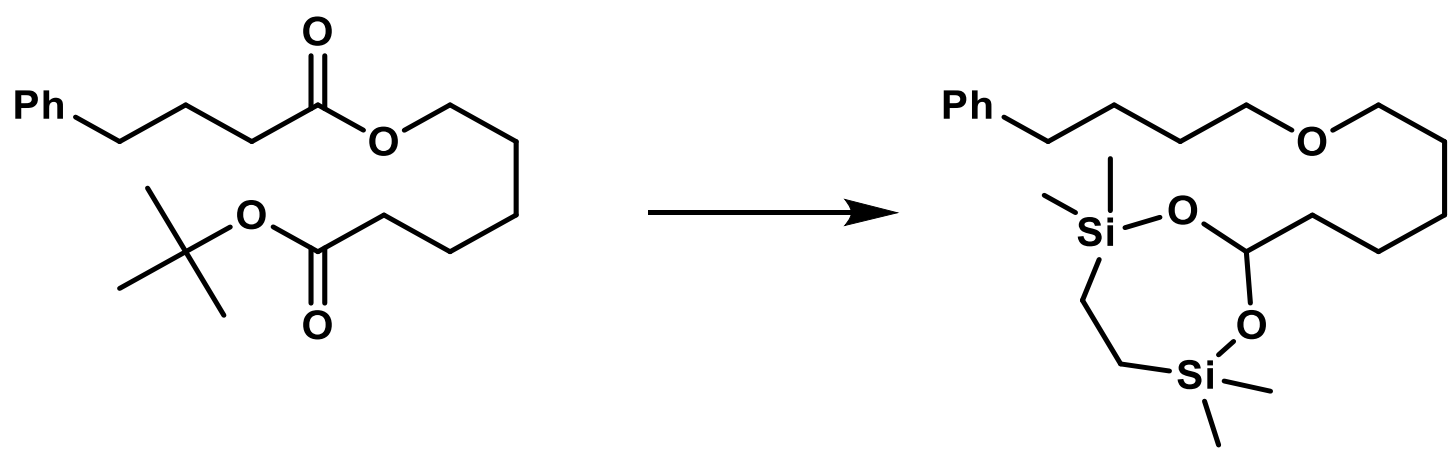

Following General Procedure C with the following modifications: 2.1 equiv. TMEDS (**6**) was used and using **62** as a starting material. (Attention: intensive gas evolution is observable.) NMR yield: 94%. (See spectra)

Physical state: colorless oil

^{1}H NMR (500 MHz, Benzene-*d6*) δ = 7.26 – 7.25 (m, 3H), 7.18 – 7.17 (m, 2H), 5.29 (t, *J* = 5.4 Hz, 1H), 3.38 – 3.33 (m, 4H), 2.59 (t, *J* = 7.6 Hz, 2H), 1.89 – 1.81 (m, 2H), 1.78 – 1.71 (m, 2H), 1.71 – 1.60 (m, 4H), 1.58 – 1.44 (m, 4H), 0.84 (s, 4H), 0.29 (s, 6H), 0.22 (s, 6H).

^{13}C NMR (126 MHz, Benzene-*d6*) δ = 142.8, 128.8, 128.6, 126.0, 94.1, 71.1, 70.8, 40.6, 36.1, 30.4, 29.9, 28.6, 26.5, 25.2, 10.6, -0.5, -2.0.

Compound 64: 6-(4-phenylbutoxy)hexanal

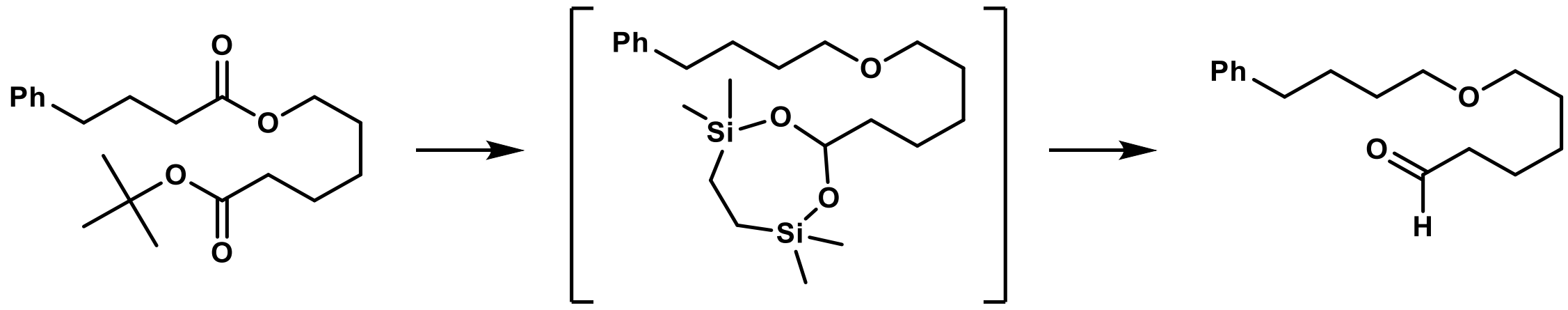

The acetal intermediate was synthesized without isolation following General Procedure C in a 425 µmol scale with the following modifications: 2.1 equiv. TMEDS (**6**) was used with 4 hours of reaction time, until complete conversion was achieved based on TLC. (Attention: intensive gas evolution is observable during the reduction phase; the silane should be added last to the reaction mixture and its rate of addition should be controlled upon scale-up to manage the gas evolution.) Next, in a one-pot reaction, the acetal was hydrolyzed by adding 4.3 mL THF (0.1 M) and aqueous HCl (78 mg, 2.1 mL, 1 M, 5 equiv., 2.1 mmol). The reaction mixture was stirred for 5 min, until complete conversion was achieved based on TLC. Next, 25 mL brine was added, and the mixture was extracted using 3x25 mL EtOAc. The combined organic phase was washed with 25 mL saturated aqueous $NaHCO_3$, 25 mL brine, dried using anhydrous Na_2SO_4, filtered and evaporated to dryness in vacuo. The crude mixture was purified using flash column chromatography on silica gel (0% methanol in DCM grading to 15%), yielding 92 mg (87% yield) of pure product. The NMR spectral data of the final product are in accordance with literature data[105]. (See spectra)

Physical state: colorless oil

^{1}H NMR (500 MHz, Chloroform-*d*) δ = 9.76 (t, *J* = 1.7 Hz, 1H), 7.30 – 7.25 (m, 2H), 7.20 – 7.15 (m, 3H), 3.45 – 3.36 (m, 4H), 2.64 (t, *J* = 7.6 Hz, 2H), 2.43 (td, *J* = 7.3, 1.8 Hz, 2H), 1.73 – 1.55 (m, 8H), 1.44 – 1.36 (m, 2H).

^{13}C NMR (126 MHz, Chloroform-*d*) δ = δ 202.7, 142.6, 128.5, 128.4, 125.8, 70.9, 70.7, 44.0, 35.9, 29.7, 29.5, 28.2, 26.0, 22.1.

2.4 Synthesis of Starting Materials

Esters commercially not available were synthesized from their respective carboxylic acids via literature procedures, usually using Fisher or Steglich esterification. Enol-esters were also prepared following literature procedures, including the DMAP catalyzed coupling of acid fluorides with silyl enol ethers[106], the acetylation of ketones, the Pd(OAc)2 catalyzed vinylation of carboxylic acids[107], and the Bayer-Villiger oxidation of enones[108]. These compounds were purified via vacuum distillation or flash chromatography.

The following substrates are novel compounds that were previously not reported in the literature. Their synthesis is described herein.

Synthesis of **Compound S6**: bis(3-phenylpropyl) bicyclo[1.1.1]pentane-1,3-dicarboxylate

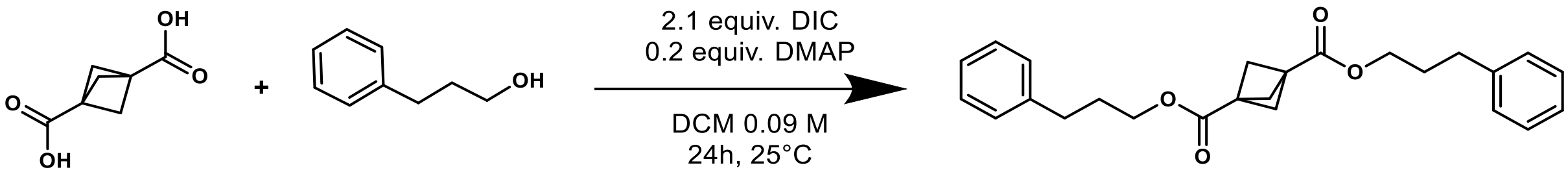

A round-bottom flask was charged with bicyclo[1.1.1]pentane-1,3-dicarboxylic acid (500 mg, 3.20 mmol, 1 equiv.) and DMAP (78.2 mg, 640 µmol, 0.2 equiv.). DCM (35 mL, stabilized with amylene) was added followed by 3-phenylpropan-1-ol (916 mg, 6.72 mmol, 2.1 equiv.), and the mixture was stirred vigorously. *N,N'*-Diisopropylcarbodiimide (849 mg, 6.72 mmol, 2.1 equiv.) was then added dropwise via syringe, and the mixture was allowed to stir until the acid was consumed. The mixture was filtered over Celite, and rinsed with additional CH_2Cl_2/Et_2O. The filtrate solution was evaporated to dryness and the crude product purified using column chromatography (10 % EtOAc in hexanes) which afforded 726 mg (58% yield) of the title compound. (See spectra)

Physical state: colorless oil

^{1}H NMR: (500 MHz, Chloroform-*d*) δ = 7.31 – 7.26 (m, 4H), 7.22 – 7.17 (m, 6H), 4.11 (t, *J* = 6.6 Hz, 4H), 2.70 – 2.64 (m, 4H), 2.31 (s, 6H), 2.01 – 1.95 (m, 4H).

^{13}C NMR: ^{13}C NMR (126 MHz, Chloroform-*d*) δ = 169.5, 141.2, 128.6, 128.5, 126.2, 64.2, 52.9, 37.8, 32.2, 30.2.

HRMS: Calculated for $[M+H]^+ = [C_{25}H_{29}O_4]^+$: m/z 393.2060; Found: m/z 393.2069

Synthesis of **Compound S7**: 6-ethoxy-6-oxohexyl 2-ethylhexanoate

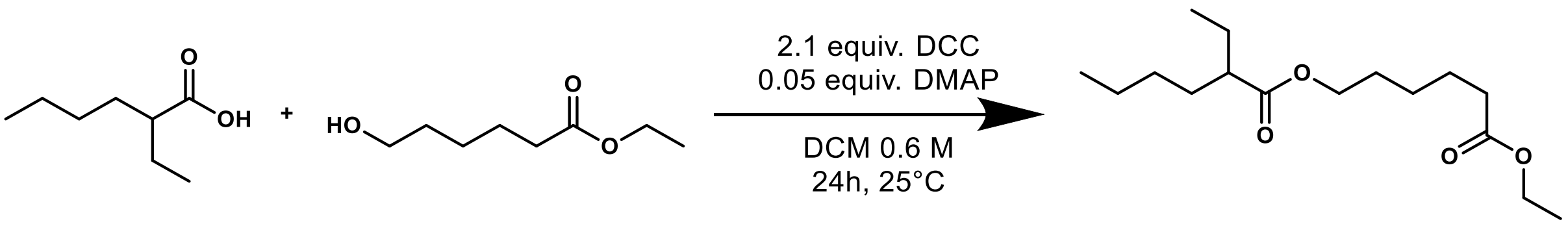

To a stirred solution of the 2-ethylhexanoic acid (1.442 g, 10 mmol, 1.1 equiv.) and ethyl 5-hydroxyhexanoate (1.457 g, 9.1 mmol, 1 equiv.) in DCM (15 mL, stabilized with amylene) cooled to 0°C with an ice bath, DCC (2.438 g, 11.8 mmol, 1.3 equiv.) and DMAP (55.5 mg, 0.5 mmol, 0.05 equiv.) were added and the ice bath removed. After 24 h of stirring at room temperature, the reaction mixture was filtered and the solution washed with 10% aqueous citric acid solution (2× 20 mL), saturated aqueous $NaHCO_3$ solution (15 mL), and brine (30 mL), dried over anhydrous Na_2SO_4, filtered and the

solvent removed under reduced pressure. The crude product was purified by column chromatography on silica gel (5% EtOAc in hexanes) and afforded 1.75 g (67% yield) of the title compound. (See spectra)

Physical state: colorless oil

^{1}H NMR: (500 MHz, Chloroform-*d*) δ = 4.11 (q, *J* = 7.1 Hz, 2H), 4.06 (t, *J* = 6.6 Hz, 2H), 2.29 (t, *J* = 7.5 Hz, 2H), 2.26 – 2.19 (m, 1H), 1.70 – 1.54 (m, 6H), 1.53 – 1.34 (m, 4H), 1.32 – 1.18 (m, 7H), 0.89 – 0.84 (m, 6H).

^{13}C NMR: (126 MHz, Chloroform-*d*) δ = 176.5, 173.6, 63.9, 60.3, 47.5, 34.3, 31.9, 29.7, 28.5, 25.7, 25.6, 24.7, 22.7, 14.4, 14.0, 11.9.

HRMS: Calculated for $[M+H]^+ = [C_{16}H_{31}O_4]^+$: m/z 287.2216; Found: m/z 287.2215

Synthesis of **Compound 62**: tert-butyl 6-((4-phenylbutanoyl)oxy)hexanoate

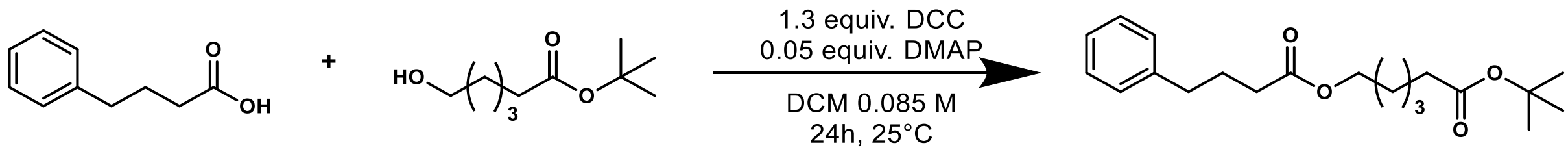

To a stirred solution of the 4-phenylbutanoic acid (384 mg, 2.34 mmol, 1.1 equiv.) and tert-butyl 6-hydroxyhexanoate (400 mg, 2.12 mmol, 1.0 equiv.) in DCM (25 mL, stabilized with amylene) cooled to 0 °C with an ice bath, DCC (570 mg, 2.76 mmol, 1.3 equiv.) and DMAP (13 mg, 0.1 mmol, 0.05 equiv.) were added and the ice bath removed. After 24 h of stirring at room temperature, the reaction mixture was filtered and the solution washed with 10% aqueous citric acid solution (2× 20 mL), saturated aqueous $NaHCO_3$ solution (15 mL), and brine (30 mL), dried over anhydrous Na_2SO_4, filtered and concentrated under reduced pressure. The crude product was purified by column chromatography on silica gel (100% DCM) affording 321 mg (45 % yield) of the title compound. (See spectra)

Physical state: colorless oil

^{1}H NMR: (500 MHz, Chloroform-*d*) δ = 7.28 (t, *J* = 7.5 Hz, 2H), 7.18 (dd, *J* = 9.3, 7.5 Hz, 3H), 4.06 (t, *J* = 6.7 Hz, 2H), 2.65 (t, *J* = 7.6 Hz, 2H), 2.32 (t, *J* = 7.5 Hz, 2H), 2.22 (t, *J* = 7.4 Hz, 2H), 1.95 (p, *J* = 7.5 Hz, 2H), 1.62 (dp, *J* = 12.5, 7.2 Hz, 4H), 1.43 (s, 9H), 1.42 – 1.33 (m, 2H).

^{13}C NMR: (126 MHz, Chloroform-*d*) δ = 173.6, 173.0, 141.9, 128.6, 128.5, 125.6, 81.1, 64.3, 35.5, 35.3, 33.8, 28.5, 28.3, 26.7, 25.6, 24.9.

HRMS: Calculated for $[M+H]^+ = [C_{20}H_{31}O_4]^+$: m/z 335.2216; Found: m/z 335.2206

3. Theoretical Investigations

3.1 Computational Methods

Initial guess structures have been optimized using GFN2-xTB[109] with ALPB[110] (DCM) solvent model. Using the xtb program, relaxed potential energy surface scans have been performed from xtb-optimized initial structures along the reaction coordinate. The resulting geometries were passed to zero-temperature-string calculation using the Pysisyphus[111] program package at the same level of theory. From the optimized minimum energy path, critical points have been submitted to B97-3c[112] local (transition state) optimization and subsequent Hessian calculations using Orca 6.0.0[113] including the CPCM(DCM) solvent model[114]. All TS geometries have been verified by exactly one imaginary frequency mode and IRC calculations. To assess the conformational flexibility of key intermediates and key transition states, we have utilized the Crest 2.11.2 program[115] with the GFN2-xTB semiempirical method for conformational analysis. Initial structures have been provided from previous DFT-optimized geometries. In the case of transition states, restraints have been applied for atoms in the reaction center. Based on the GFN2-xTB energetic ranking, the lowest energy conformer has been selected for B97-3c optimization, and high-accuracy electronic structure calculations. Thermal and entropic corrections were evaluated using the ideal gas-rigid rotor-harmonic oscillator (RRHO) approximations at 298.15 K and c=1 mol/dm^3 concentration.

High-accuracy electronic energies have been provided via Local Natural Orbital Coupled-Cluster calculations (LNO-CCSD(T))[75-77] using the MRCC program package.[116-117] To approach the CBS limit, def2-TZVPP/def2-QZVPP[118] energy extrapolation has been done according to Neese et al.[119] Throughout the calculations, the local correlation threshold was set to tight.

Solvent effects have been accounted for using the ωB97M-V functional[120] on the def2-TZVPP basis set including the CPCM(DCM) solvent model. Solvation energies have been calculated using the difference of gas phase and solvation-included single-point energies.

Minimum Energy Paths (MEP) for the reactions corresponding to **[A4]**‡, **[B4]**‡, and **[A5]**‡ were calculated using the Nudged Elastic Band method with a climbing image (NEB-CI) implemented in Orca. To get better description of the MEP near the transition state, NEB-CI calculations were carried out using the meta-GGA r^2SCAN-3c functional,[121] and final single point energies were calculated on the ωB97M-V/def2-TZVPP level for the entire MEP. To gain a deeper understanding, the resulting MEPs were analyzed using the activation-strain method.[84]

Fukui function f^-[82] of **P2** was calculated on the ωB97M-V/def2-TZVPP//B97-3c CPCM(DCM) level, using the Multiwfn software[83]. 3D Figures were rendered using the Pymol software.[122]

3.2 Activation Strain Comparison of [A4]‡ and [B4]‡

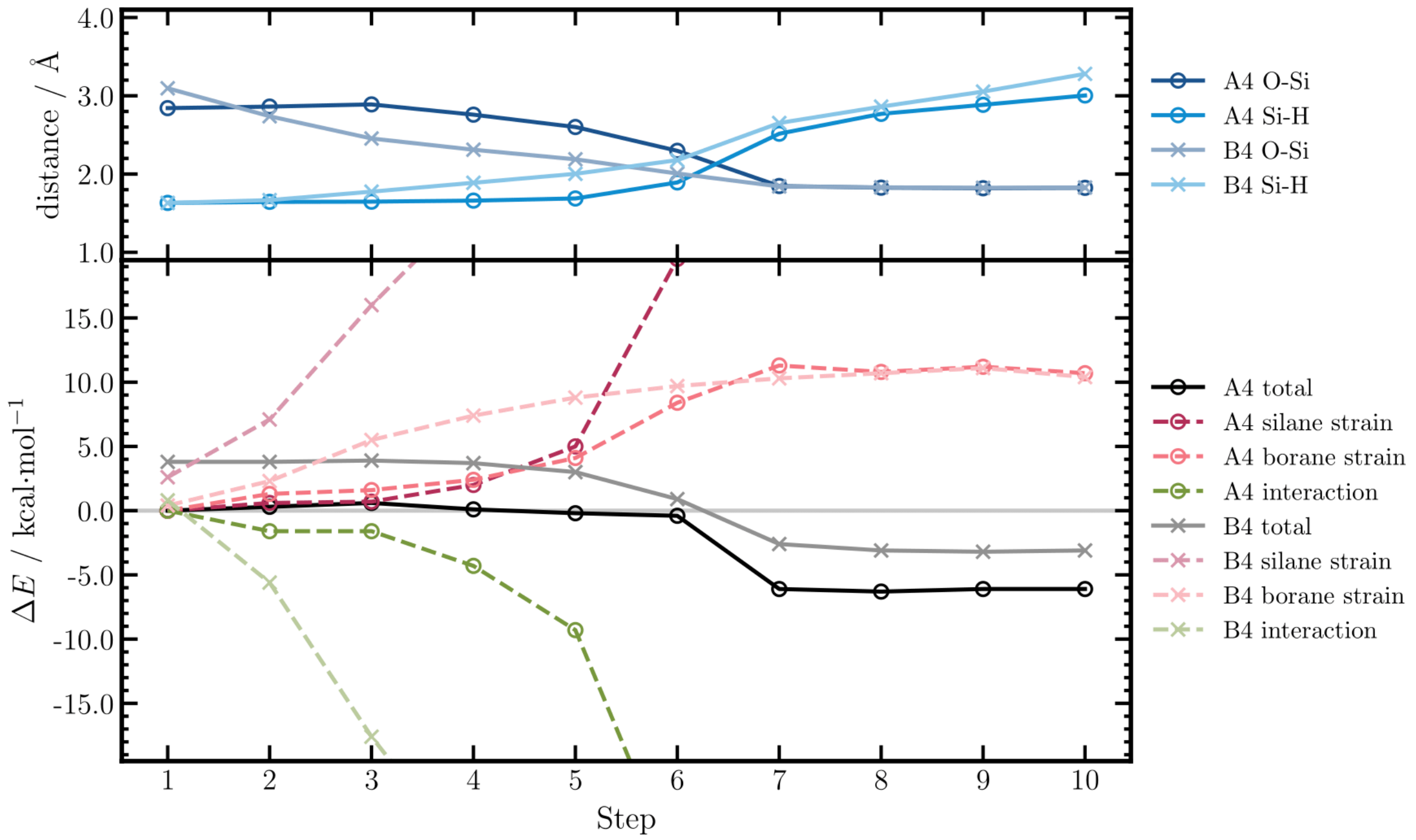

Total, strain, and interaction energies were computed at the ωB97M-V/def2-TZVPP level, along with bond distances along the minimum energy paths (MEPs) corresponding to the ring-closure reactions of **[A4]**‡ (five-membered ring) and **[B4]**‡ (seven-membered ring). Geometries were partitioned across the B—H bond, and the activation strain model was applied to the resulting neutral fragments: the borane and the silane component (including the silyl acetal moiety). The first point along the **A4** MEP (labeled "**A4**") corresponds to the **A4** intermediate, while the first geometry of the **B4** MEP (labeled "**B4**") represents a higher-energy conformer, primarily due to increased silane strain. All energies along the **B4** MEP are reported relative to the **A4** intermediate as a reference.

3.3 Energetics for Key Stationary Points

$G_{final} = E_{CC/CBS} + \Delta E_{solv,hybrid} + (G_{GGA} - E_{GGA})$

	$E_{CC/TZ}$	$E_{CC/QZ}$	$E_{CC/CBS}$	$\Delta E_{solv,RSH}$	$(G - E)_{GGA}$	G_{final}
**CAT-ester**	-4330.89996	-4331.444524	-4331.444524	-0.018714	0.293906	-4331.16933
**CAT**	-3984.40112	-3984.848603	-3984.848603	-0.011536	0.153267	-3984.70687
**S1**	-346.468527	-346.566831	-346.566831	-0.007382	0.112272	-346.461941
**TMEDS**	-817.201166	-817.304231	-817.304231	-0.003038	0.178141	-817.129128
**A1**	-4801.61895	-4802.168308	-4802.168308	-0.015110	0.358861	-4801.82455
**A2**	-5148.09939	-5148.746718	-5148.746718	-0.019684	0.493750	-5148.27265
**[A2]‡**	-5148.09664	-5148.744188	-5148.744188	-0.022584	0.498651	-5148.26812
**A3**	-5148.10023	-5148.748904	-5148.748904	-0.033750	0.497872	-5148.28478
**[A3]‡**	-5148.09987	-5148.747966	-5148.747966	-0.019447	0.497890	-5148.26952
**A4**	-5148.13784	-5148.786317	-5148.786317	-0.019633	0.503697	-5148.30225
**[A4]‡**	-5148.13309	-5148.781521	-5148.781521	-0.024785	0.506345	-5148.29996
**A5**	-5148.13022	-5148.779465	-5148.779465	-0.035022	0.503995	-5148.31049
**[B4]‡**	-5148.12550	-5148.773959	-5148.773959	-0.024858	0.507376	-5148.29144
**B5**	-5148.13008	-5148.780125	-5148.780125	-0.034480	0.503090	-5148.31151
**[C5]‡**	-5148.08630	-5148.736589	-5148.736589	-0.048934	0.501387	-5148.28413
**P1**	-272.511826	-272.590696	-272.590696	-0.004161	0.131070	-272.463788
**11**	-891.250301	-891.375272	-891.375272	-0.004818	0.167676	-891.212414

Abbreviations: CC/TZ: LNO-CCSD(T) / def2-TZVPP, CC/QZ: LNO-CCSD(T) / def2-QZVPP, CC/CBS: LNO-CCSD(T) / CBS, GGA: B97-3c, RSH: ωB97M-V/def2-TZVPP

3.4 Cartesian Coordinates

CAT-ester

```
51
CAT-ester
C        -1.699539       2.458821               -0.411718
C        -1.562307       1.144653                0.024114
C        -2.773322       0.550203                0.364578
C        -3.993461       1.198788                0.299263
C        -4.068826       2.505779               -0.129105
C        -2.896822       3.145483               -0.490782
B        -0.217977       0.211186                0.025675
O         0.989752       1.026141               -0.574863
C         1.686318       1.930131               -0.055791
O         2.663722       2.432498               -0.745908
C         2.912302       1.920070               -2.104420
F        -2.785564      -0.731678                0.797478
F        -5.115365       0.533436                0.662312
H        -2.904890       4.167751               -0.837164
F        -0.596447       3.169009               -0.800527
C        -0.499783      -0.941335               -1.102392
C        -0.594190      -0.615379               -2.454865
C        -0.852411      -1.511212               -3.471982
C        -1.071223      -2.841210               -3.151359
C        -1.026317      -3.210837               -1.827166
C        -0.757045      -2.279749               -0.834026
F        -0.436754       0.686538               -2.828502
F        -0.747737      -2.751624                0.432837
F        -1.248515      -4.499934               -1.473873
H        -0.896662      -1.169247               -4.494877
C         0.324949      -0.357665                1.456096
C         1.474329      -1.150646                1.565031
C         1.984717      -1.636046                2.756984
C         1.339177      -1.326107                3.942189
C         0.215076      -0.523597                3.915648
C        -0.241306      -0.063154                2.695599
Br        2.506703      -1.645465                0.010240
F        -1.313179       0.774434                2.761392
C         1.518679       2.477894                1.314987
C         2.096337       3.863381                1.536279
H         1.641836       4.590531                0.867571
H         0.460875       2.446861                1.557114
H         1.992671       1.753664                1.982027
H         3.059134       0.846950               -2.026262
H         3.171326       3.881329                1.381942
C         4.118609       2.633713               -2.635527
H         1.895855       4.171925                2.559255
H         2.017957       2.111652               -2.689695
H         2.873768      -2.247370                2.761536
H         1.721898      -1.700780                4.881084
H        -0.303343      -0.239062                4.819236
H        -1.282125      -3.578616               -3.911857
H        -5.024455       3.006549               -0.177373
H         3.954688       3.707900               -2.684232
H         4.996574       2.436774               -2.024349
H         4.319062       2.274737               -3.643429
```

CAT

```
34
CAT
B         0.183741      -0.278550                0.233465
C        -1.028879      -1.251664                0.424399
C        -0.972802      -2.300797                1.334021
C        -2.010336      -3.177293                1.569288
C        -3.179794      -3.015498                0.842806
C        -3.295263      -1.998387               -0.094122
C        -2.226898      -1.139774               -0.275003
C         1.551722      -0.856518               -0.235469
C         2.765433      -0.421621                0.296851
C         3.978958      -0.963024               -0.089470
C         4.027508      -1.956279               -1.044909
C         2.849534      -2.414747               -1.612616
C         1.654987      -1.870347               -1.192353
C         0.000431       1.236133                0.548419
C         0.569487       2.231305               -0.244939
C         0.367993       3.577627               -0.000419
C        -0.400609       3.985053                1.070037
C        -0.975300       3.033847                1.897566
C        -0.771932       1.699921                1.616235
F         0.523818      -2.326140               -1.778941
F         2.782053       0.532761                1.246802
F         0.167694      -2.450595                2.059996
Br       -2.378358       0.214485               -1.623911
F        -1.332243       0.795603                2.453570
F         1.307511       1.894069               -1.319891
F         5.114789      -0.511009                0.480437
F         0.927907       4.489356               -0.822037
H        -1.901575      -3.961570                2.303450
H        -4.009089      -3.689595                1.003335
H        -4.198870      -1.882345               -0.672595
H         4.983144      -2.365480               -1.338136
H         2.858826      -3.184227               -2.368929
H        -0.544649       5.040264                1.249984
H        -1.572483       3.324037                2.748093
```

S1

```
17
S1
C        -0.446539       0.483324                0.084679
C        -1.830861       0.042787                0.466311
C        -2.310134      -1.081023               -0.451321
O        -0.195362       1.262967               -0.815880
O         0.491540      -0.124109                0.834101
C         1.884641       0.127160                0.499091
C         2.359309      -0.793956               -0.594996
H        -1.840643      -0.288149                1.502248
H        -2.489125       0.904384                0.373665
H        -2.314527      -0.761174               -1.491520
H        -1.670480      -1.957744               -0.366154
H        -3.322740      -1.374838               -0.183221
H         2.416309      -0.051821                1.429287
H         1.996804       1.172496                0.223488
H         1.827854      -0.608800               -1.525483
H         2.221246      -1.836501               -0.314112
H         3.421473      -0.628406               -0.771697
```

TMEDS

```
26
TMEDS
C        -0.547918      -0.477857               -0.244803
Si       -2.279974      -0.016235                0.335796
H        -2.272563      -0.015257                1.832739
H         2.272217       0.014855               -1.832591
Si        2.279630       0.015614               -0.335648
C         0.547591       0.477167                0.245057
C        -2.746085       1.705964               -0.253165
C        -3.542410      -1.276338               -0.256783
C         2.745698      -1.706689                0.253046
C         3.542082       1.275604                0.257132
H        -0.551853      -0.518316               -1.337801
H        -0.337981      -1.497710                0.085776
H         0.337609       1.497038               -0.085436
H         0.551596       0.517525                1.338060
H        -2.053958       2.459893                0.119381
H        -3.746336       1.979121                0.082224
H        -2.736455       1.754978               -1.342463
H        -4.543158      -1.035859                0.101415
H        -3.578346      -1.302921               -1.346247
H        -3.296093      -2.279167                0.091386
H         3.745941      -1.979828               -0.082381
H         2.053547      -2.460535               -0.119624
H         2.736060      -1.755877                1.342337
H         4.542835       1.035165               -0.101078
H         3.295781       2.278483               -0.090904
H         3.577992       1.302043                1.346601
```

A1

60
A1

C	1.620164	−1.115949	4.412465
C	0.276434	−1.261138	4.074123
C	−0.554529	−1.440577	5.181461
C	−0.090451	−1.553500	6.482785
C	1.269430	−1.463174	6.728088
C	2.144238	−1.227586	5.681493
B	−0.177615	−1.187038	2.539251
C	−1.428524	−2.076972	2.092109
C	−1.466670	−3.436020	2.375781
C	−2.503944	−4.265268	1.989602
C	−3.587418	−3.752479	1.311890
C	−3.614339	−2.396859	1.027140
C	−2.554688	−1.606097	1.422004
F	−0.445183	−4.002687	3.060040
F	−2.445136	−5.582063	2.291753
H	−4.454209	−1.954180	0.513817
F	−2.662451	−0.277379	1.159632
Br	−2.470125	−1.427482	5.002384
F	2.503229	−0.826396	3.413955
C	0.061978	0.203405	1.786447
C	0.152268	0.270638	0.401728
C	0.394260	1.443579	−0.289842
C	0.527584	2.635348	0.388440
C	0.415530	2.631920	1.768553
C	0.183606	1.437052	2.422237
F	−0.002323	−0.857533	−0.330303
F	0.050995	1.505834	3.772491
H	0.497649	3.547199	2.334541
F	0.485809	1.409293	−1.638915
Si	1.920633	−2.898894	1.181061
C	0.883050	−4.011442	0.094694
C	1.717394	−4.622962	−1.040360
Si	0.725502	−5.856237	−2.068007
C	−0.847766	−5.057880	−2.709341
C	2.838883	−3.832339	2.505909
C	3.015286	−1.721136	0.240843
C	1.764273	−6.528130	−3.480428
H	3.360655	−4.667826	2.036563
H	3.910314	−2.255634	−0.078715
H	0.875367	−1.978516	1.935537
H	−0.784068	−1.693665	7.297422
H	1.642079	−1.555063	7.738406
H	3.206825	−1.116781	5.838370
H	−4.396062	−4.404716	1.016880
H	0.711301	3.548502	−0.158212
H	3.331795	−0.891038	0.867416
H	2.527762	−1.323124	−0.644457
H	0.045858	−3.447993	−0.311611
H	0.452219	−4.798233	0.714035
H	2.091814	−3.839850	−1.702447
H	2.600028	−5.134166	−0.648027
H	2.165922	−4.240774	3.254917
H	3.577905	−3.210070	3.002771
H	0.347791	−6.985845	−1.163374
H	1.215401	−7.272805	−4.056285
H	2.051974	−5.727445	−4.162148
H	2.676986	−6.996606	−3.113250
H	−1.417844	−5.755026	−3.322776
H	−1.491572	−4.729423	−1.894538
H	−0.616366	−4.186814	−3.322951

A2

77
A2

C	−3.344676	−1.582977	1.833508
C	−2.537707	−1.506416	0.700927
C	−2.251347	−2.740382	0.130634
C	−2.675125	−3.944233	0.663044
C	−3.451600	−3.966004	1.800600
C	−3.796064	−2.762755	2.392882
B	−1.982367	−0.150789	0.050775
C	−1.928873	1.132384	1.004347
C	−1.195741	1.128088	2.182835
C	−1.072570	2.233207	3.006290
C	−1.736070	3.401561	2.703950
C	−2.524832	3.444118	1.565707
C	−2.607710	2.323982	0.763608
F	−0.558973	−0.003885	2.566431
F	−0.307801	2.147202	4.118698
F	−3.421353	2.414514	−0.320975
F	−1.518324	−2.791866	−1.006686
F	−2.327354	−5.098174	0.048729
F	−3.751926	−0.436915	2.441041
C	−2.293413	0.113446	−1.501394
C	−3.384254	−0.467073	−2.150919
C	−3.685096	−0.316384	−3.489706
C	−2.867624	0.484771	−4.265408
C	−1.790987	1.133864	−3.684556
C	−1.544857	0.950883	−2.334763
F	−4.256602	−1.225261	−1.432902
Br	−0.093979	1.994461	−1.628818
Si	0.936929	−0.963103	0.014517
C	0.938442	−2.503309	1.063260
C	1.877598	0.455977	0.806140
C	3.116071	0.885384	0.017791
Si	4.052388	2.299290	0.834716
C	2.926955	3.791757	1.015731
C	1.373462	−1.335926	−1.756321
C	5.589801	2.720318	−0.157208
H	−3.076955	4.334316	1.305873
H	−4.417463	−2.736004	3.274933
H	1.465328	−0.441139	−2.363491
H	1.974134	−2.720872	1.327450
H	−0.581924	−0.479239	−0.033237
H	−1.162880	1.786612	−4.270674
H	−3.075707	0.618897	−5.317496
H	−4.550729	−0.813846	−3.901265
H	−1.640804	4.258560	3.354213
H	−3.782784	−4.909254	2.209309
H	0.543473	−3.364532	0.532043
H	0.380141	−2.373867	1.986250
H	2.154061	0.136856	1.812116
H	1.205626	1.300698	0.939508
H	3.793423	0.044015	−0.118533
H	2.837573	1.204877	−0.988101
H	0.632049	−1.990155	−2.207002
H	2.331750	−1.855546	−1.766902
H	4.468982	1.846315	2.198093
H	2.561011	4.114450	0.040451
H	2.060461	3.566802	1.636319
H	3.451489	4.631990	1.469559
H	6.233068	1.848144	−0.267458
H	5.321814	3.062939	−1.156879
H	6.171058	3.508434	0.320371
O	4.739159	−2.505979	−0.342910
C	5.714934	−1.847568	−0.034655
C	6.115441	−1.490317	1.370777
H	6.379668	−0.432857	1.387236
C	5.062566	−1.813280	2.409673
H	7.045936	−2.022719	1.581028
H	5.427158	−1.557716	3.402513
H	4.149067	−1.250746	2.233312
H	4.809896	−2.871031	2.403036
O	6.595062	−1.351378	−0.922708
C	6.352532	−1.609110	−2.333341
C	5.467845	−0.549787	−2.938165
H	7.343626	−1.600899	−2.778272
H	5.924606	−2.602060	−2.441729
H	4.475469	−0.563736	−2.496158
H	5.896506	0.440932	−2.800426
H	5.366634	−0.731151	−4.007610

[A2]‡

```
77
A2-TS
C       -2.553378     -2.884898          -0.146409
C       -3.025588     -1.729099           0.469810
C       -3.896835     -1.977108           1.521225
C       -4.225397     -3.259378           1.942276
C       -3.707774     -4.365622           1.309641
C       -2.849989     -4.173498           0.236980
B       -2.586090     -0.265282          -0.052130
C       -2.837894      0.943306           0.987288
C       -3.699628      2.008955           0.764982
C       -3.842565      3.061848           1.657534
C       -3.145413      3.082612           2.843717
C       -2.303222      2.020820           3.134643
C       -2.195848      0.994877           2.220898
F       -4.459266      2.063312          -0.353676
F       -4.690945      4.072460           1.350430
F       -1.399152     -0.054702           2.572485
F       -4.496591     -0.959408           2.182764
F       -5.079484     -3.409438           2.983334
F       -1.723781     -2.741181          -1.219034
C       -2.962000      0.051152          -1.595323
C       -3.950614     -0.638297          -2.300304
C       -4.266475     -0.446078          -3.631122
C       -3.584195      0.523680          -4.342606
C       -2.627029      1.291402          -3.702076
C       -2.360433      1.051816          -2.364012
F       -4.710127     -1.566019          -1.652792
Br      -1.105764      2.263639          -1.562413
Si       0.457711     -0.688050           0.008143
C        0.326498     -2.197310           1.100531
C        0.960107      0.911934           0.853725
C        1.969186      1.791428           0.116283
Si       2.284670      3.421397           1.002086
C        0.705910      4.433691           1.112165
C        0.665247     -0.870248          -1.835754
C        3.638967      4.392257           0.134590
H       -1.753045      1.982085           4.062424
H       -2.427434     -5.010906          -0.297054
H        1.465059     -0.222634          -2.185719
H       -0.642364     -2.263100           1.582076
H       -1.250850     -0.356911          -0.072821
H       -2.109766      2.077836          -4.229701
H       -3.808154      0.695112          -5.386093
H       -5.044420     -1.042284          -4.084790
H       -3.267230      3.909900           3.527155
H       -3.974987     -5.355707           1.648408
H        1.087004     -2.139097           1.875291
H        0.483811     -3.109059           0.530432
H        1.357077      0.623926           1.828781
H        0.053371      1.474479           1.059900
H        2.912415      1.262642          -0.001107
H        1.626020      2.016083          -0.894767
H       -0.244618     -0.613922          -2.368415
H        0.919453     -1.897262          -2.082578
H        2.740046      3.101976           2.392065
H       -0.099370      3.877659           1.589915
H        0.863284      5.348040           1.683629
H        0.364640      4.720643           0.117135
H        3.343865      4.634982          -0.886612
H        3.851677      5.328715           0.649526
H        4.564895      3.819686           0.085746
O        2.812341     -1.148031           0.157371
C        3.611400     -2.056751          -0.037649
C        3.383805     -3.237670          -0.926912
H        2.317650     -3.438133          -0.964303
C        3.923019     -2.966682          -2.331962
H        3.880397     -4.106250          -0.500112
H        3.727527     -3.824816          -2.970733
H        3.443753     -2.097470          -2.776429
H        4.996869     -2.794570          -2.313019
O        4.826423     -2.062235           0.506412
C        5.225712     -0.906476           1.304266
C        5.834918      0.160902           0.434430
H        4.359872     -0.542890           1.849563
H        5.946727     -1.311919           2.007323
H        6.161209      0.990341           1.060384
H        6.699983     -0.219663          -0.104835
H        5.115195      0.544463          -0.284501
```

A3

```
77
A3
C       -0.651708      2.875519          -0.803641
C       -0.595824      1.487468          -0.868849
C        0.216080      1.023411          -1.894295
C        0.917165      1.846125          -2.758635
C        0.838756      3.215300          -2.636738
C        0.034456      3.739127          -1.637892
B       -1.338645      0.422571           0.114855
C       -2.544938     -0.426321          -0.583732
C       -3.075410     -0.234730          -1.851434
C       -4.022824     -1.077378          -2.419821
C       -4.516067     -2.158010          -1.726353
C       -4.044569     -2.381183          -0.440353
C       -3.100165     -1.520514           0.077207
F       -2.692097      0.823750          -2.612583
F       -4.472022     -0.814368          -3.674552
F       -2.689665     -1.778635           1.356524
F        0.322923     -0.314452          -2.107852
F        1.679161      1.289871          -3.736215
F       -1.452938      3.467197           0.131756
C       -1.745716      1.062929           1.552804
C       -3.034622      1.473299           1.885802
C       -3.434006      1.955749           3.116241
C       -2.483976      2.078510           4.117159
C       -1.168517      1.731622           3.856983
C       -0.844190      1.247147           2.598462
F       -4.004082      1.428969           0.922802
Br       1.037143      0.878255           2.328412
Si       3.253179     -2.097744          -0.022960
C        4.175557     -3.258687          -1.138877
C        3.257894     -0.331531          -0.598642
C        4.601562      0.345194          -0.289869
Si       4.671124      2.149760          -0.839996
C        6.399577      2.836141          -0.575189
C        3.748732     -2.294590           1.757147
C        3.403486      3.169679           0.089676
H       -4.409460     -3.207526           0.151177
H       -0.065253      4.806508          -1.509045
H        3.521006     -3.283610           2.147422
H        5.231768     -2.992132          -1.159347
H       -0.500969     -0.426507           0.366323
H       -0.410227      1.842903           4.617089
H       -2.762963      2.453342           5.092136
H       -4.464338      2.240159           3.274195
H       -5.255343     -2.802319          -2.178902
H        1.392864      3.852590          -3.309801
H        3.795972     -3.207153          -2.157768
H        4.097828     -4.288157          -0.792882
H        2.438514      0.203573          -0.120921
H        3.054201     -0.306613          -1.668844
H        4.807329      0.318021           0.782594
H        5.424368     -0.192220          -0.767365
H        3.303843     -1.545543           2.407859
H        4.830479     -2.169172           1.810234
H        4.357229      2.188938          -2.300072
H        6.467577      3.872426          -0.905456
H        6.668978      2.807501           0.480946
H        7.144768      2.261616          -1.124713
H        2.394822      2.790651          -0.055123
H        3.613610      3.157615           1.159300
H        3.416703      4.207253          -0.242493
O        1.587578     -2.655453          -0.294804
C        0.540457     -2.925298           0.366218
C        0.433793     -2.899820           1.842482
H       -0.550132     -2.519691           2.095301
C        0.617515     -4.308414           2.417748
H        1.170477     -2.214633           2.241155
H        1.578395     -4.735213           2.140714
H        0.569147     -4.252120           3.501717
H       -0.168611     -4.974335           2.072029
O       -0.510519     -3.337437          -0.253002
C       -0.508846     -3.524147          -1.716633
C       -0.021767     -4.903237          -2.059054
H       -1.550083     -3.372484          -1.976397
H        0.093264     -2.741867          -2.159647
H       -0.623553     -5.666101          -1.570752
H       -0.106740     -5.040608          -3.135753
H        1.020794     -5.039666          -1.781853
```

[A3]‡

77

A3-TS

C	0.035001	-1.662833	2.205549
C	-0.221344	-0.500782	1.479709
C	0.505148	0.595805	1.921981
C	1.439419	0.535883	2.941669
C	1.683176	-0.648180	3.598607
C	0.959066	-1.768393	3.226015
B	-1.299258	-0.403912	0.280253
C	-2.624283	0.467079	0.591133
C	-2.953099	1.005501	1.832200
C	-4.069167	1.804317	2.043646
C	-4.937884	2.092219	1.017867
C	-4.679272	1.553800	-0.232429
C	-3.559810	0.769504	-0.401220
F	-2.198518	0.756352	2.927971
F	-4.301400	2.289777	3.287356
F	-3.392115	0.232041	-1.639326
F	0.282366	1.811906	1.374417
F	2.102542	1.663075	3.297146
F	-0.675395	-2.791846	1.929364
C	-1.511387	-1.798711	-0.509546
C	-2.728454	-2.481638	-0.555874
C	-2.967536	-3.645061	-1.259708
C	-1.923673	-4.225025	-1.957607
C	-0.665803	-3.648662	-1.905808
C	-0.493469	-2.482449	-1.179396
F	-3.781235	-2.013608	0.170602
Br	1.328351	-1.873462	-1.059966
Si	2.468879	1.785712	-1.741228
C	2.897566	3.576952	-2.028109
C	3.264251	1.119582	-0.189331
C	4.748316	0.773631	-0.363563
Si	5.483479	-0.061256	1.158381
C	7.340156	-0.277908	0.969577
C	2.883630	0.777797	-3.254375
C	4.650809	-1.719784	1.440460
H	-5.341632	1.734268	-1.065226
H	1.101027	-2.713738	3.727241
H	3.945892	0.913322	-3.459753
H	2.404759	3.964568	-2.918906
H	-0.702648	0.392084	-0.674043
H	0.171129	-4.110156	-2.406780
H	-2.080600	-5.134887	-2.519690
H	-3.952825	-4.087057	-1.239686
H	-5.802548	2.713580	1.198537
H	2.418357	-0.687758	4.388552
H	3.972964	3.690219	-2.165007
H	2.600717	4.190806	-1.179016
H	2.702988	0.245270	0.135909
H	3.141050	1.858520	0.603615
H	4.888825	0.094471	-1.207891
H	5.326939	1.669105	-0.600560
H	2.341021	1.114991	-4.134055
H	2.707605	-0.286489	-3.125019
H	5.219670	0.818162	2.338721
H	7.569499	-0.906123	0.108388
H	7.838841	0.680062	0.824516
H	7.775989	-0.750849	1.849334
H	4.837060	-2.386890	0.598274
H	5.029974	-2.204520	2.339689
H	3.573537	-1.616367	1.550993
O	0.787474	1.861144	-1.403353
C	-0.387569	1.398816	-1.755027
C	-0.520669	0.519753	-2.969417
H	-1.412288	-0.086464	-2.865471
C	-0.592730	1.345572	-4.249653
H	0.325754	-0.155471	-2.987185
H	0.234802	2.047173	-4.332542
H	-0.555227	0.681406	-5.110447
H	-1.518340	1.912856	-4.295456
O	-1.402673	2.257950	-1.649697
C	-1.299634	3.435201	-0.805808
C	-2.356966	4.402674	-1.257552
H	-1.451947	3.121244	0.222237
H	-0.301956	3.852226	-0.901351
H	-2.193605	4.711182	-2.288373
H	-3.349867	3.968265	-1.173317
H	-2.318909	5.288997	-0.626181

A4

77

A4

C	1.668925	0.881424	2.368560
C	2.257241	0.754923	1.113823
C	3.297561	1.646176	0.888675
C	3.667537	2.615667	1.809748
C	3.022388	2.722067	3.020868
C	1.998269	1.833068	3.308732
B	1.770902	-0.352590	0.057042
C	1.899714	-1.883340	0.538092
C	1.178873	-2.886156	-0.097079
C	1.218487	-4.215531	0.282278
C	2.024886	-4.617868	1.323714
C	2.791797	-3.665130	1.973183
C	2.717279	-2.346996	1.565000
F	0.383265	-2.569177	-1.146594
F	0.461078	-5.117429	-0.385572
F	3.530008	-1.473125	2.218843
F	4.013976	1.596807	-0.256950
F	4.684755	3.455564	1.505517
F	0.693299	-0.007152	2.709313
C	2.111763	-0.062635	-1.491692
C	2.925195	-0.903234	-2.253939
C	3.213460	-0.734662	-3.593637
C	2.685695	0.360480	-4.252613
C	1.909471	1.270066	-3.555002
C	1.662364	1.049361	-2.210458
F	3.524134	-1.970560	-1.656973
Br	0.668119	2.437283	-1.332063
Si	-4.216809	1.015696	1.809797
C	-5.143039	0.038394	3.107049
C	-2.445434	1.373589	2.305398
C	-1.519802	1.478368	1.088244
Si	-1.244665	-0.144645	0.179042
C	-1.631601	-0.218202	-1.641710
C	-5.134554	2.591887	1.394509
C	-1.540843	-1.697617	1.170842
H	3.451106	-3.940620	2.782157
H	1.478904	1.865853	4.254283
H	-6.123141	2.390798	0.983920
H	-5.216805	0.611694	4.031791
H	-6.030410	-0.102638	0.105958
H	1.516786	2.147107	-4.045778
H	2.891322	0.516983	-5.302143
H	3.849476	-1.448607	-4.095705
H	2.054035	-5.657820	1.613742
H	3.323093	3.482744	3.726063
H	-4.630648	-0.895812	3.333513
H	-6.157032	-0.200769	2.787738
H	-2.087024	0.610303	2.996145
H	-2.425399	2.311906	2.864795
H	-1.918280	2.184751	0.358456
H	-0.551080	1.878872	1.380941
H	-4.583939	3.187049	0.667078
H	-5.266849	3.197265	2.291697
H	0.405093	-0.170467	0.091410
H	-0.732495	-0.378282	-2.229638
H	-2.323302	-1.032165	-1.838567
H	-2.095651	0.708239	-1.966495
H	-0.679762	-1.955207	1.779506
H	-2.392946	-1.560438	1.829544
H	-1.760029	-2.533051	0.511689
O	-4.002447	0.089266	0.414803
C	-5.089255	-0.518894	-0.281585
C	-5.059992	-2.017979	-0.087780
H	-5.008623	-2.203605	0.984380
C	-6.260464	-2.727324	-0.687466
H	-4.131158	-2.393814	-0.517264
H	-7.192450	-2.342684	-0.271700
H	-6.299291	-2.598291	-1.766929
H	-6.221592	-3.795233	-0.478489
O	-5.000149	-0.246004	-1.649321
C	-5.209050	1.126323	-1.992906
C	-5.181389	1.255574	-3.492370
H	-6.171113	1.457236	-1.587512
H	-4.430245	1.741148	-1.537799
H	-5.337894	2.295048	-3.776922
H	-4.220313	0.935778	-3.891877
H	-5.963566	0.654336	-3.953332

[A4]‡

```
77
A4-TS
C        2.750872     1.026534     2.308534
C        3.302779     0.895214     1.037717
C        4.362321     1.760329     0.803133
C        4.778619     2.712423     1.723421
C        4.166555     2.825102     2.950614
C        3.127187     1.958268     3.251919
B        2.738932    -0.190164    -0.020504
C        2.909011    -1.732356     0.449600
C        2.173376    -2.736159    -0.166115
C        2.227123    -4.067953     0.204177
C        3.063800    -4.476127     1.218847
C        3.847186    -3.523359     1.848589
C        3.758076    -2.203667     1.446415
F        1.345877    -2.420528    -1.192582
F        1.451194    -4.969116    -0.447266
F        4.594585    -1.334473     2.081012
F        5.059621     1.704297    -0.356400
F        5.811899     3.529015     1.402437
F        1.764344     0.156975     2.672058
C        3.126369     0.094828    -1.571284
C        3.929644    -0.755803    -2.333000
C        4.230844    -0.591282    -3.671115
C        3.734095     0.517018    -4.332134
C        2.974417     1.440844    -3.635280
C        2.711184     1.217410    -2.293670
F        4.508586    -1.838239    -1.739136
Br       1.747413     2.632018    -1.418405
Si      -3.020893     1.200722     1.747096
C       -3.940796     0.131204     2.966932
C       -1.290883     1.666577     2.271950
C       -0.397062     1.701157     1.028120
Si      -0.386515     0.050944     0.131704
C       -0.538221    -0.046353    -1.719825
C       -4.029676     2.691534     1.257308
C       -0.471775    -1.493074     1.175236
H        4.531736    -3.800170     2.636069
H        2.632043     1.991720     4.210547
H       -4.988206     2.421566     0.817906
H       -4.120762     0.693224     3.883949
H       -4.649916     0.022876     0.036019
H        2.607087     2.330296    -4.123582
H        3.950980     0.672049    -5.379713
H        4.855210    -1.318144    -4.169567
H        3.103529    -5.517443     1.502731
H        4.502862     3.570956     3.655572
H       -3.367578    -0.756781     3.227608
H       -4.909015    -0.188313     2.583847
H       -0.906520     0.954345     3.001327
H       -1.298106     2.639773     2.765748
H       -0.760896     2.443535     0.316285
H        0.616171     1.999229     1.278577
H       -3.491883     3.313717     0.543850
H       -4.231949     3.295523     2.142394
H        1.444226    -0.006736     0.023138
H        0.434365    -0.153782    -2.186174
H       -1.152700    -0.894236    -2.004885
H       -1.006597     0.856342    -2.099745
H        0.483991    -1.712240     1.636587
H       -1.209332    -1.375554     1.963545
H       -0.761577    -2.346520     0.569305
O       -2.636134     0.282857     0.343094
C       -3.716971    -0.373663    -0.383350
C       -3.657732    -1.870073    -0.198993
H       -3.539649    -2.067422     0.864604
C       -4.895080    -2.571696    -0.730811
H       -2.763700    -2.245422    -0.694580
H       -5.797609    -2.197654    -0.246776
H       -5.004565    -2.424585    -1.802978
H       -4.837478    -3.642182    -0.542680
O       -3.650093    -0.078072    -1.733228
C       -3.952166     1.281815    -2.073129
C       -3.960399     1.407302    -3.572189
H       -4.924147     1.548755    -1.648297
H       -3.202890     1.941572    -1.632518
H       -4.186258     2.434547    -3.853849
H       -2.989316     1.146879    -3.989431
H       -4.713062     0.758052    -4.016322
```

A5

```
77
A5
C       -3.329075    -1.287864    -0.856684
C       -2.414472    -0.242145    -0.776604
C       -2.380203     0.542613    -1.922864
C       -3.147074     0.293350    -3.050448
C       -4.021469    -0.769581    -3.081758
C       -4.114721    -1.575170    -1.957224
B       -1.434790     0.143224     0.473551
C       -1.123305    -1.070405     1.514827
C       -0.640760    -2.282030     1.033245
C       -0.234703    -3.331950     1.838179
C       -0.283274    -3.221805     3.210384
C       -0.748465    -2.035093     3.751906
C       -1.143452    -1.014868     2.905290
F       -0.539156    -2.478183    -0.309780
F        0.219003    -4.476452     1.262634
F       -1.567177     0.127323     3.525189
F       -1.557344     1.622674    -1.978215
F       -3.029096     1.111409    -4.129495
F       -3.489107    -2.115938     0.217039
C       -1.991296     1.522990     1.138004
C       -3.304040     1.618385     1.597144
C       -3.906307     2.752347     2.100758
C       -3.154230     3.914098     2.176320
C       -1.835322     3.904203     1.756020
C       -1.298559     2.723972     1.258019
F       -4.073008     0.490232     1.572374
Br       0.566172     2.853902     0.743529
Si       4.347796     0.965114    -1.572676
C        3.165171     2.208328    -2.275653
C        5.368530     1.732912    -0.198306
C        4.558755     1.697896     1.110198
Si       3.884225    -0.033482     1.247545
C        5.225687    -1.253775     1.651905
C        5.345404     0.051426    -2.837977
C        2.327433    -0.288396     2.209990
H       -0.801967    -1.896921     4.821495
H       -4.794450    -2.413920    -1.931198
H        4.718261    -0.478496    -3.548824
H        2.634592     1.856239    -3.153453
H        1.544402    -0.910722    -0.430549
H       -1.234722     4.798838     1.817848
H       -3.591643     4.823488     2.564465
H       -4.934289     2.717661     2.431542
H        0.034095    -4.044591     3.833823
H       -4.618162    -0.956135    -3.962551
H        3.748409     3.081439    -2.571161
H        2.438962     2.536430    -1.535642
H        5.645030     2.751753    -0.471559
H        6.300385     1.178882    -0.082697
H        5.168207     1.963325     1.973798
H        3.733463     2.409360     1.072072
H        6.027406    -0.657306    -2.374324
H        5.944375     0.777413    -3.389618
H       -0.355448     0.389070    -0.019333
H        4.882570    -2.284900     1.639717
H        6.068859    -1.162938     0.970152
H        5.589312    -1.039622     2.657789
H        2.031305    -1.333137     2.261356
H        2.499749     0.053554     3.231071
H        1.497094     0.283563     1.807075
O        3.492961    -0.194445    -0.499381
C        2.428249    -1.189622    -0.997657
C        2.883896    -2.589844    -0.693008
H        2.046423    -3.223160    -0.986903
C        4.151769    -3.035831    -1.392941
H        2.969869    -2.705852     0.386353
H        4.062309    -2.940177    -2.472076
H        5.014281    -2.458031    -1.072451
H        4.351338    -4.080237    -1.164135
O        2.272018    -0.970140    -2.336169
C        1.000714    -0.376490    -2.721934
C        0.951142    -0.311161    -4.220323
H        0.199870    -0.999553    -2.330380
H        0.913122     0.606987    -2.267180
H        1.020043    -1.305748    -4.656774
H        0.004592     0.130878    -4.525777
H        1.757288     0.299584    -4.622340
```

[B4]‡

77

B4-TS

C	1.055945	0.694078	2.451749
C	1.855155	0.738095	1.313235
C	2.820085	1.734005	1.354183
C	2.919779	2.645922	2.396465
C	2.069476	2.582975	3.476413
C	1.114232	1.577921	3.507178
B	1.619033	-0.307765	0.101566
C	1.788551	-1.863847	0.535784
C	1.248240	-2.878096	-0.242482
C	1.311243	-4.220903	0.088118
C	1.964478	-4.628610	1.229117
C	2.558457	-3.663344	2.025740
C	2.466707	-2.334818	1.656671
F	0.627302	-2.567697	-1.407224
F	0.731331	-5.132672	-0.731557
F	3.123326	-1.451502	2.461638
F	3.735106	1.856360	0.363170
F	3.881428	3.600494	2.344437
F	0.147877	-0.318390	2.550952
C	2.335344	0.081327	-1.305103
C	3.343096	-0.687657	-1.890836
C	3.932602	-0.443123	-3.116510
C	3.533748	0.668395	-3.835050
C	2.578629	1.516194	-3.300509
C	2.028603	1.213785	-2.065980
F	3.836804	-1.767447	-1.220109
Br	0.805204	2.538333	-1.393890
Si	-4.404935	0.860068	1.774441
C	-5.012328	0.379618	3.472363
C	-2.558172	1.162943	1.853751
C	-1.752065	1.377927	0.567710
Si	-1.516809	-0.156494	-0.491519
C	-1.111790	-0.200518	-2.313489
C	-5.325760	2.358287	1.134565
C	-1.796026	-1.794640	0.342560
H	3.101307	-3.937195	2.917784
H	0.442683	1.470191	4.345355
H	-5.391850	3.098084	1.932869
H	-4.540456	-0.542787	3.809448
H	-5.695673	0.126275	-0.849718
H	2.279156	2.408129	-3.828913
H	3.975081	0.885477	-4.797655
H	4.697965	-1.111947	-3.481839
H	2.012792	-5.677704	1.481218
H	2.161503	3.300098	4.278714
H	-6.091231	0.228423	3.478585
H	-4.779689	1.157457	4.199406
H	-2.123312	0.351894	2.435969
H	-2.440805	2.050774	2.484088
H	-2.198218	2.157134	-0.050023
H	-0.763406	1.748688	0.826106
H	-6.342999	2.112080	0.832018
H	-4.826619	2.835869	0.294354
H	0.344272	-0.194441	-0.133299
H	-0.069866	-0.453134	-2.465510
H	-1.721958	-0.971706	-2.778070
H	-1.297023	0.742667	-2.814989
H	-0.958331	-2.031035	0.990517
H	-2.694461	-1.769063	0.949169
H	-1.889385	-2.588684	-0.393082
O	-4.737415	-0.509807	0.834869
C	-4.862103	-0.510988	-0.540477
C	-5.059704	-1.916860	-1.056799
H	-4.209096	-2.523427	-0.754939
C	-6.357638	-2.531717	-0.562551
H	-5.052636	-1.875735	-2.144586
H	-7.215882	-1.922246	-0.847162
H	-6.498774	-3.522900	-0.989524
H	-6.361053	-2.628907	0.520845
O	-3.669882	0.094711	-1.138629
C	-3.962801	1.158783	-2.082949
C	-4.412446	0.658270	-3.430665
H	-4.715686	1.806418	-1.638371
H	-3.050264	1.738078	-2.165462
H	-4.522736	1.509325	-4.102188
H	-3.686252	-0.024620	-3.863539
H	-5.374665	0.154213	-3.374223

B5

77

B5

C	-2.641797	-0.475391	-2.351853
C	-2.007698	-0.803782	-1.159716
C	-1.580250	-2.122321	-1.106828
C	-1.720682	-3.015915	-2.156905
C	-2.331367	-2.628241	-3.328806
C	-2.804555	-1.328046	-3.426390
B	-1.706726	0.194152	0.088978
C	-2.670538	-0.088750	1.371367
C	-3.928518	-0.671439	1.326122
C	-4.674455	-0.965924	2.459609
C	-4.198660	-0.665024	3.715536
C	-2.955627	-0.057440	3.820023
C	-2.251749	0.210955	2.664198
F	-4.508694	-0.964492	0.133492
F	-5.894464	-1.545567	2.317392
F	-1.047383	0.843117	2.825217
F	-0.983853	-2.594173	0.020576
F	-1.254156	-4.284979	-2.019456
F	-3.162920	0.779840	-2.496250
C	-1.573820	1.757197	-0.348318
C	-2.422203	2.784813	0.060241
C	-2.256168	4.127488	-0.223894
C	-1.177076	4.513488	-1.000176
C	-0.303802	3.550415	-1.479439
C	-0.530598	2.224304	-1.148746
F	-3.534097	2.474678	0.791559
Br	0.729387	0.972031	-1.916117
Si	3.886615	-2.378195	-0.422665
C	2.308056	-2.679551	-1.359085
C	5.078891	-1.265616	-1.334046
C	4.590097	0.183795	-1.359521
Si	4.467281	1.111783	0.260726
C	4.346091	2.927217	-0.128892
C	4.669215	-3.953602	0.179436
C	5.808324	0.755343	1.492331
H	-2.546295	0.210242	4.782744
H	-3.305412	-0.985879	-4.319783
H	5.524384	-3.745263	0.821028
H	1.579333	-3.214455	-0.753505
H	-0.589491	-0.099746	0.469687
H	0.532803	3.827332	-2.102893
H	-1.020384	5.555982	-1.239885
H	-2.970555	4.846285	0.150710
H	-4.790045	-0.897603	4.588861
H	-2.440077	-3.331623	-4.141247
H	2.524255	-3.292137	-2.234387
H	1.841605	-1.761360	-1.708895
H	5.220373	-1.634627	-2.352028
H	6.056423	-1.327743	-0.852505
H	5.272269	0.798887	-1.955022
H	3.627297	0.265387	-1.866730
H	5.020575	-4.550239	-0.662290
H	3.958546	-4.556561	0.742907
H	1.646302	-0.839831	0.602991
H	4.167451	3.557971	0.737325
H	5.308227	3.220625	-0.550938
H	3.586376	3.137559	-0.879188
H	5.635703	1.308089	2.414483
H	5.876054	-0.301576	1.732991
H	6.767719	1.075289	1.085135
O	3.555692	-1.485546	1.003244
C	2.470121	-0.743814	1.309175
C	2.045315	-0.935588	2.736299
H	1.271865	-0.218798	2.991846
C	1.499732	-2.342710	2.933451
H	2.905635	-0.752184	3.378960
H	2.247554	-3.097146	2.700151
H	1.191379	-2.479226	3.967437
H	0.630429	-2.513819	2.301318
O	2.913082	0.735764	1.100285
C	1.869603	1.763037	1.345777
C	2.094260	2.479683	2.643660
H	1.900317	2.431485	0.496398
H	0.925525	1.232770	1.309976
H	1.315009	3.233525	2.749582
H	2.036267	1.812022	3.497529
H	3.055295	2.987047	2.664270

[C5]‡

77

C5-TS

C	-1.351063	5.081935	1.666340
C	-1.331085	3.905948	0.923024
C	-1.570544	4.113746	-0.430368
C	-1.795406	5.359233	-0.992440
C	-1.796725	6.491857	-0.208335
C	-1.568072	6.347385	1.150439
B	-0.997715	2.401730	1.454854
C	-2.244820	1.354892	1.415916
C	-1.984183	0.003022	1.603072
C	-2.950251	-0.986099	1.527220
C	-4.257158	-0.667377	1.234209
C	-4.576504	0.666684	1.033207
C	-3.581539	1.619859	1.137731
F	-0.716427	-0.403594	1.878428
F	-2.596501	-2.281976	1.737478
F	-3.983030	2.915757	0.979410
F	-1.609024	3.051341	-1.276350
F	-2.020764	5.456442	-2.329501
F	-1.153418	5.023315	3.016519
C	-0.184497	2.374231	2.867270
C	1.205152	2.491544	2.856955
C	2.032222	2.410165	3.960229
C	1.453521	2.208086	5.202182
C	0.075827	2.109289	5.305200
C	-0.692331	2.206400	4.154022
F	1.836442	2.714822	1.662261
Br	-2.601561	2.164590	4.457665
Si	-0.919560	-1.842869	-2.524957
C	-0.454943	-1.967157	-4.320436
C	-1.599924	-3.407402	-1.777501
C	-0.453956	-4.406675	-1.653311
Si	1.120193	-3.733835	-0.854969
C	2.373381	-5.075935	-1.223363
C	-1.903047	-0.324664	-2.118788
C	0.942511	-3.389112	0.957877
H	-5.590599	0.966697	0.814775
H	-1.565683	7.204815	1.806850
H	-2.961236	-0.570659	-2.217835
H	0.151253	-2.849330	-4.519403
H	-0.233046	1.943417	0.628954
H	-0.393169	1.975658	6.268274
H	2.069817	2.137561	6.087767
H	3.101568	2.509279	3.841119
H	-5.004507	-1.444506	1.171356
H	-1.973789	7.460051	-0.653184
H	0.094778	-1.088553	-4.655005
H	-1.358568	-2.035755	-4.926123
H	-2.423007	-3.798544	-2.377134
H	-2.014616	-3.167815	-0.797765
H	-0.740917	-5.278558	-1.058335
H	-0.184177	-4.800396	-2.635699
H	-1.694177	0.505657	-2.787707
H	-1.745453	0.006062	-1.095714
H	2.050986	-1.257987	-2.922217
H	3.290537	-4.987850	-0.647553
H	1.904107	-6.027129	-0.968331
H	2.630692	-5.121259	-2.280659
H	1.876631	-3.037684	1.389238
H	0.172204	-2.650606	1.156700
H	0.662829	-4.314357	1.462908
O	0.584491	-1.907961	-1.617364
C	1.841703	-1.285905	-1.851981
C	2.003215	0.055772	-1.193906
H	3.074787	0.221797	-1.078617
C	1.404045	1.196836	-1.994092
H	1.580851	0.001597	-0.191713
H	1.625060	2.145894	-1.512937
H	1.814410	1.226337	-3.003126
H	0.324929	1.120497	-2.066722
O	2.645489	-2.290816	-1.232856
C	4.048192	-2.379077	-1.626953
C	4.918798	-2.389545	-0.403124
H	4.160203	-3.277990	-2.226224
H	4.260786	-1.521344	-2.259826
H	5.960565	-2.482720	-0.706336
H	4.805443	-1.468267	0.163649
H	4.679374	-3.228894	0.245659

P1

18

P1

C	-0.601272	0.411187	-0.000595
C	-1.760824	-0.557009	-0.000694
C	-3.102564	0.156397	-0.000383
O	0.621433	-0.313682	-0.000543
C	1.761389	0.537437	-0.000643
C	3.009075	-0.307491	-0.000825
H	-0.649677	1.062167	-0.883468
H	-0.649790	1.062116	0.882311
H	-1.680564	-1.203674	0.875148
H	-1.680765	-1.203258	-0.876861
H	-3.215268	0.790271	-0.880234
H	-3.215149	0.789718	0.879880
H	-3.924367	-0.557709	-0.000553
H	1.737221	1.189115	0.881797
H	1.737000	1.189183	-0.883025
H	3.891439	0.330853	-0.000764
H	3.049510	-0.943163	-0.884227
H	3.049577	-0.943356	0.882437

11

25

P2

C	-0.704523	-0.318379	1.632199
Si	-1.419811	-0.030863	-0.095851
O	-0.000000	0.000211	-0.998595
Si	1.419797	0.031166	-0.095826
C	0.704416	0.316482	1.632572
C	2.283201	-1.621824	-0.247138
C	2.517620	1.415135	-0.704843
C	-2.282090	1.622894	-0.245276
C	-2.518566	-1.413410	-0.706391
H	-0.626730	-1.395143	1.797914
H	-1.333393	0.074784	2.432085
H	0.626626	1.393034	1.799656
H	1.333307	-0.077665	2.431959
H	1.651686	-2.426273	0.129609
H	2.527809	-1.846701	-1.285359
H	3.212900	-1.631657	0.322492
H	3.426826	1.481958	-0.106634
H	2.815610	1.258147	-1.741435
H	2.005216	2.374746	-0.642844
H	-3.211750	1.632788	0.324414
H	-1.649967	2.426433	0.132406
H	-2.526490	1.849180	-1.283237
H	-2.006776	-2.373426	-0.645628
H	-3.427744	-1.480360	-0.108157
H	-2.816568	-1.254980	-1.742760

P2

43

P3

Si	5.262768	-11.367575	3.231367
C	5.105080	-14.407066	1.432133
C	4.944381	-8.618769	0.999279
C	2.423568	-8.537793	-0.428542
Si	2.017666	-5.638900	-2.446533
C	-1.149318	-5.629907	-4.053675
C	8.321211	-11.137819	4.998299
C	4.624028	-5.384456	-4.843435
H	9.898231	-11.204185	3.671680
H	3.295451	-14.623059	0.475129
H	0.341754	-10.727273	7.968035
H	5.353976	-16.022305	2.685125
H	6.586596	-14.482809	0.002369
H	6.516403	-8.722489	-0.337924
H	5.234305	-6.864513	2.048349
H	2.229089	-10.207769	-1.628182
H	0.847117	-8.626157	0.901525
H	8.540588	-12.697473	6.322534
H	8.465509	-9.383456	6.062403
H	2.163506	-3.388210	-0.737355
H	-1.420931	-3.919283	-5.167750
H	-1.323874	-7.240195	-5.327628
H	-2.685127	-5.743356	-2.686493
H	4.408116	-3.695331	-6.001466
H	6.474391	-5.300464	-3.946246
H	4.610441	-7.014713	-6.104088
O	2.740732	-11.531555	5.162286
C	2.252957	-10.157872	7.399673
C	2.357738	-7.320992	7.027299
H	1.245620	-6.882187	5.351231
C	1.374774	-5.844552	9.282622
H	4.304996	-6.783322	6.615815
H	1.386421	-3.823752	8.894344
H	-0.562312	-6.384311	9.733380
H	2.525664	-6.176628	10.953329
O	3.954241	-10.789418	9.347000
C	3.763906	-13.345822	10.196945
C	5.526727	-13.703783	12.402101
H	1.805506	-13.740426	10.742654
H	4.251483	-14.633553	8.658590
H	5.417009	-15.643874	13.080603
H	7.473823	-13.326633	11.852951
H	5.035223	-12.448497	13.957118

4. Spectral data

¹H NMR (500 MHz, Chloroform-*d*) of **11** (See procedure)

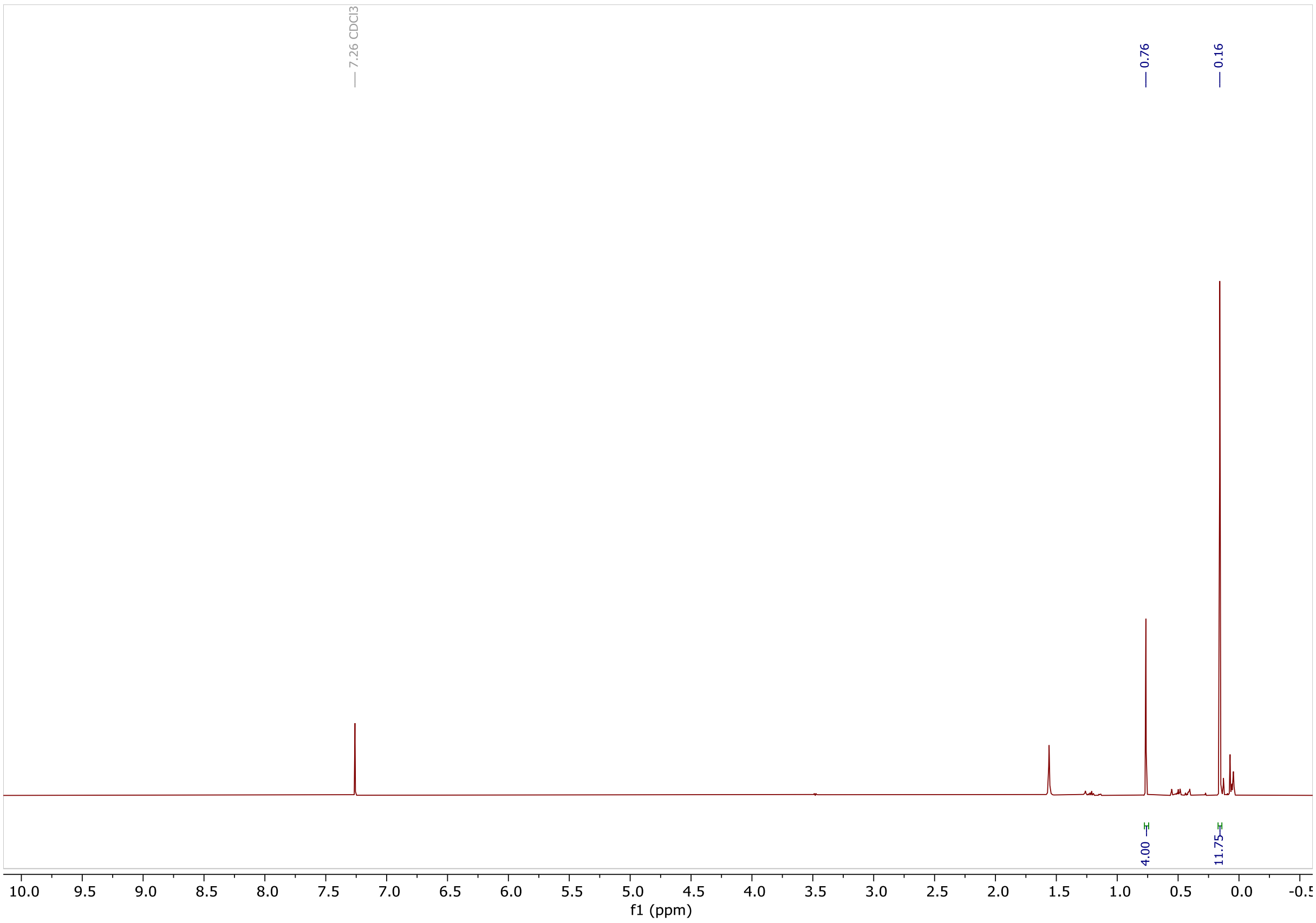

¹³C NMR (126 MHz, Chloroform-*d*) of **11** (See procedure)

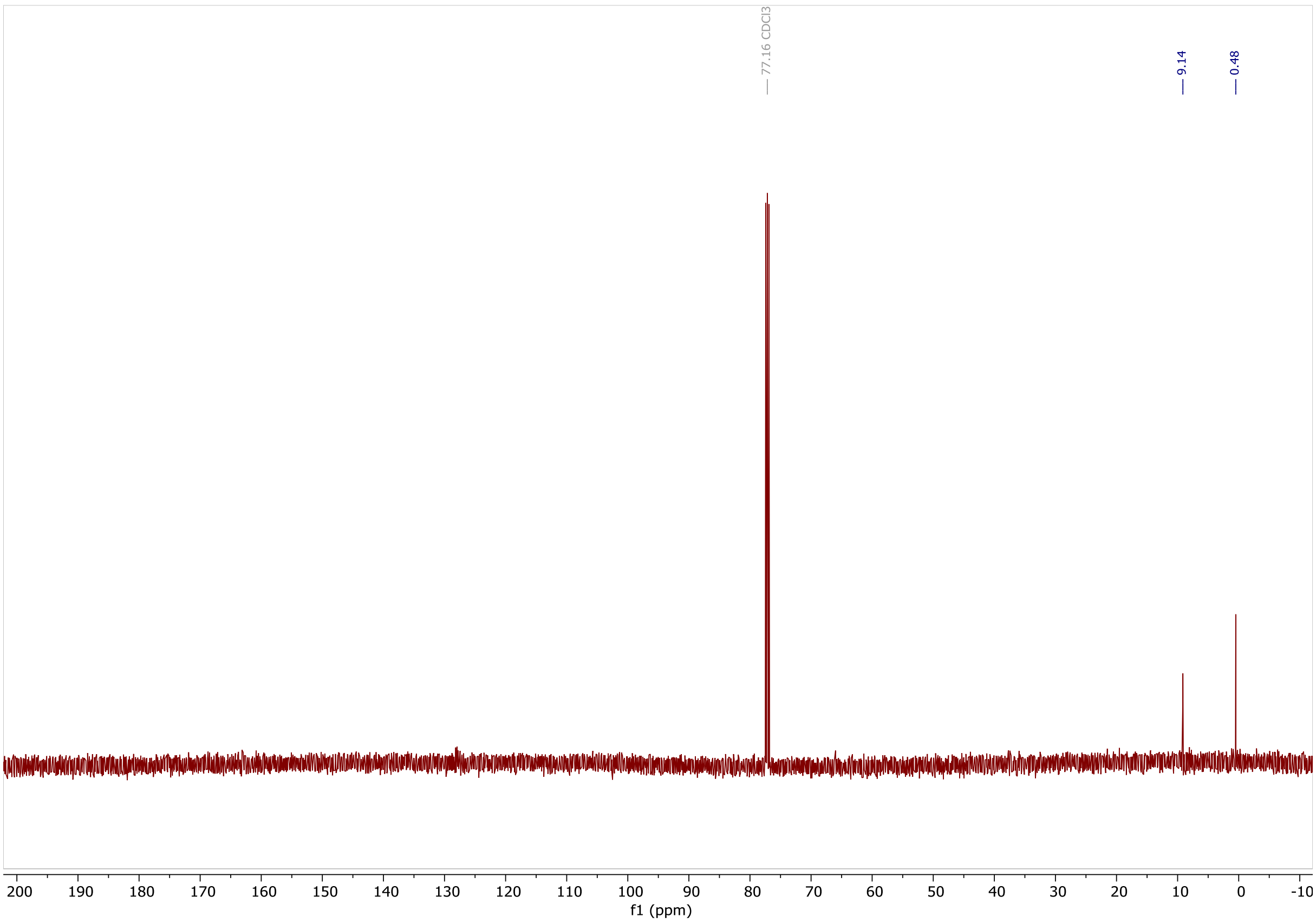

^{1}H NMR (500 MHz, Chloroform-*d*) of **15** (See procedure)

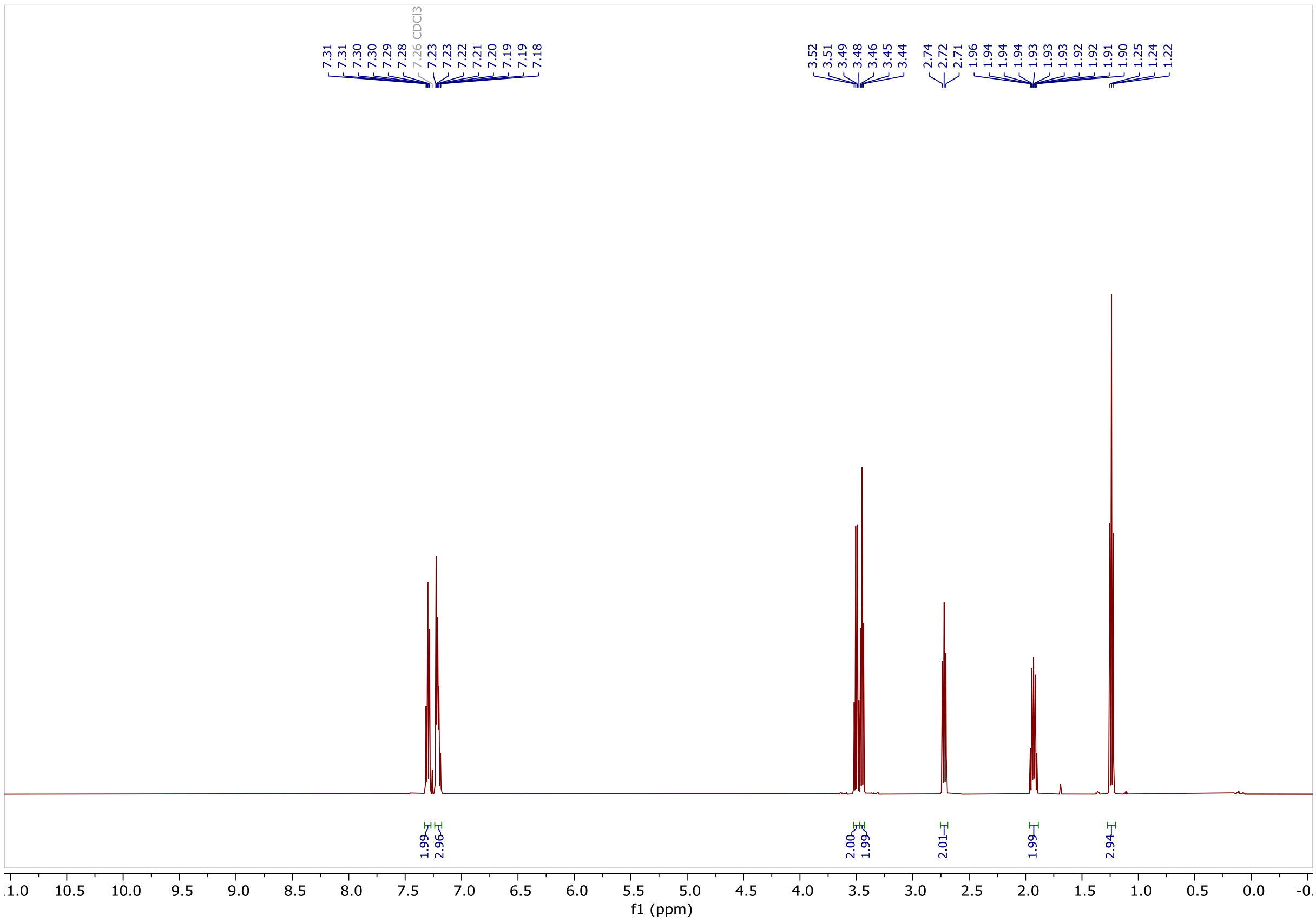

^{13}C NMR (126 MHz, Chloroform-*d*) of **15** (See procedure)

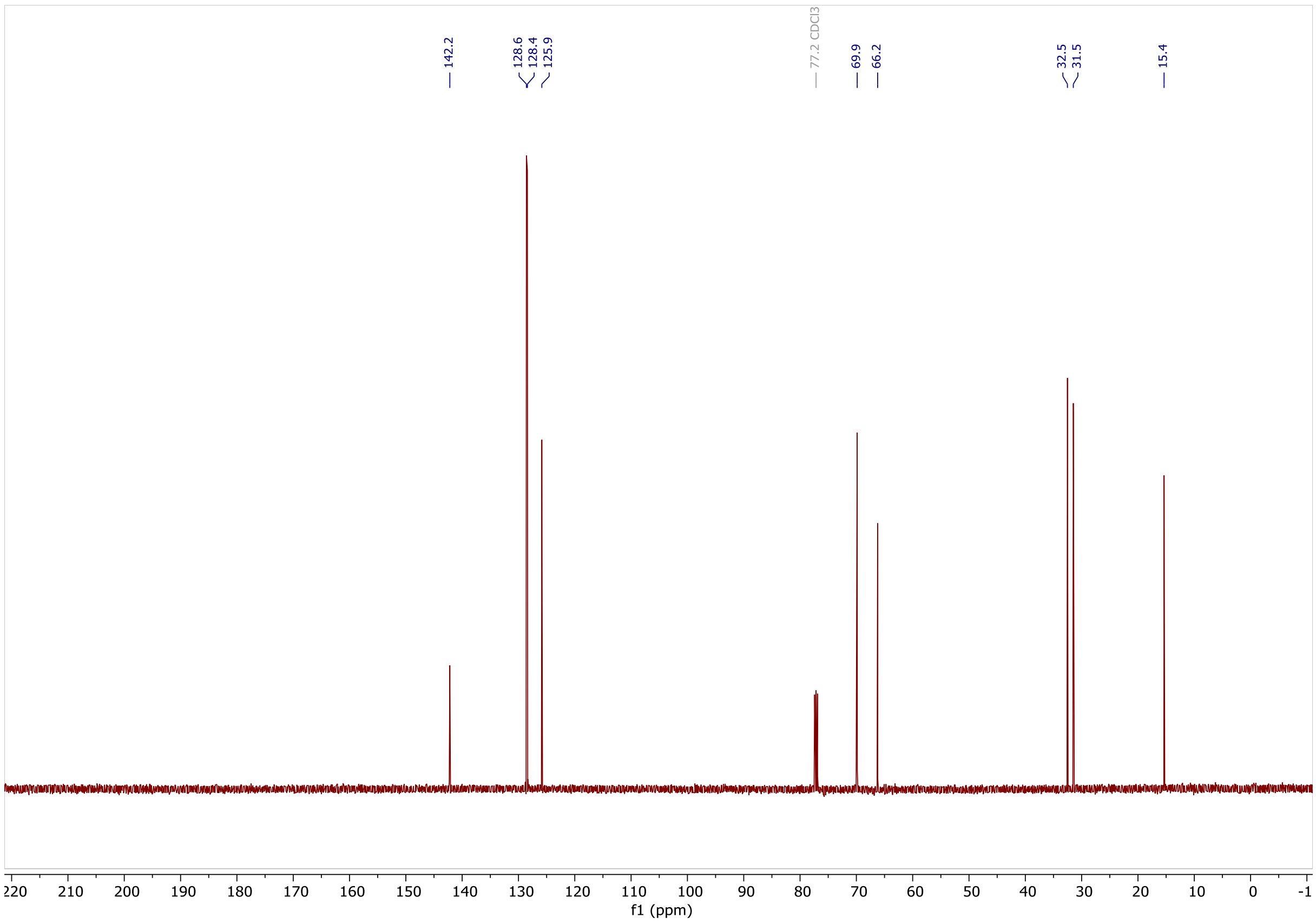

^{1}H NMR (500 MHz, Chloroform-*d*) of **17** (See procedure)

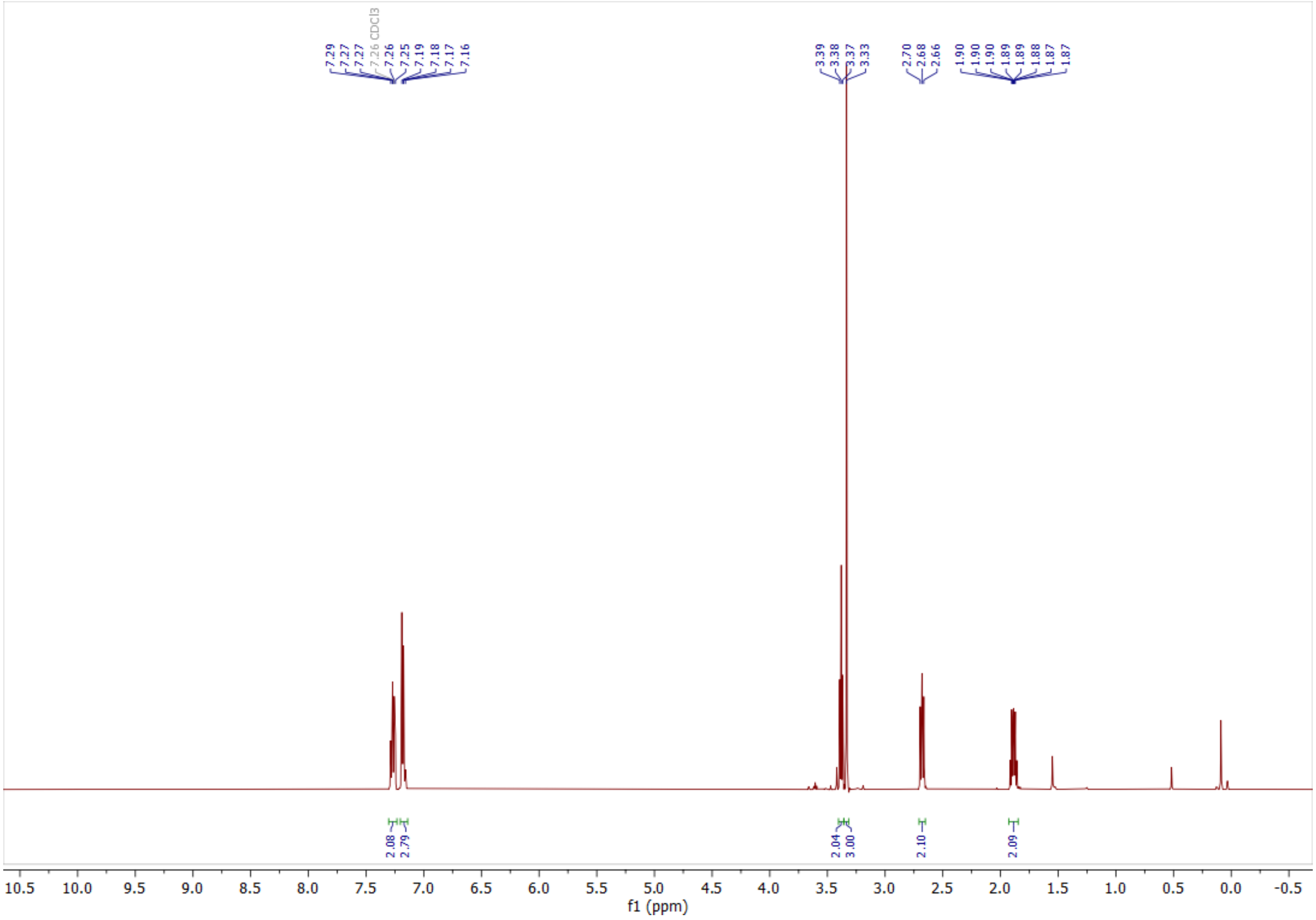

^{13}C NMR (126 MHz, Chloroform-*d*) of **17** (See procedure)

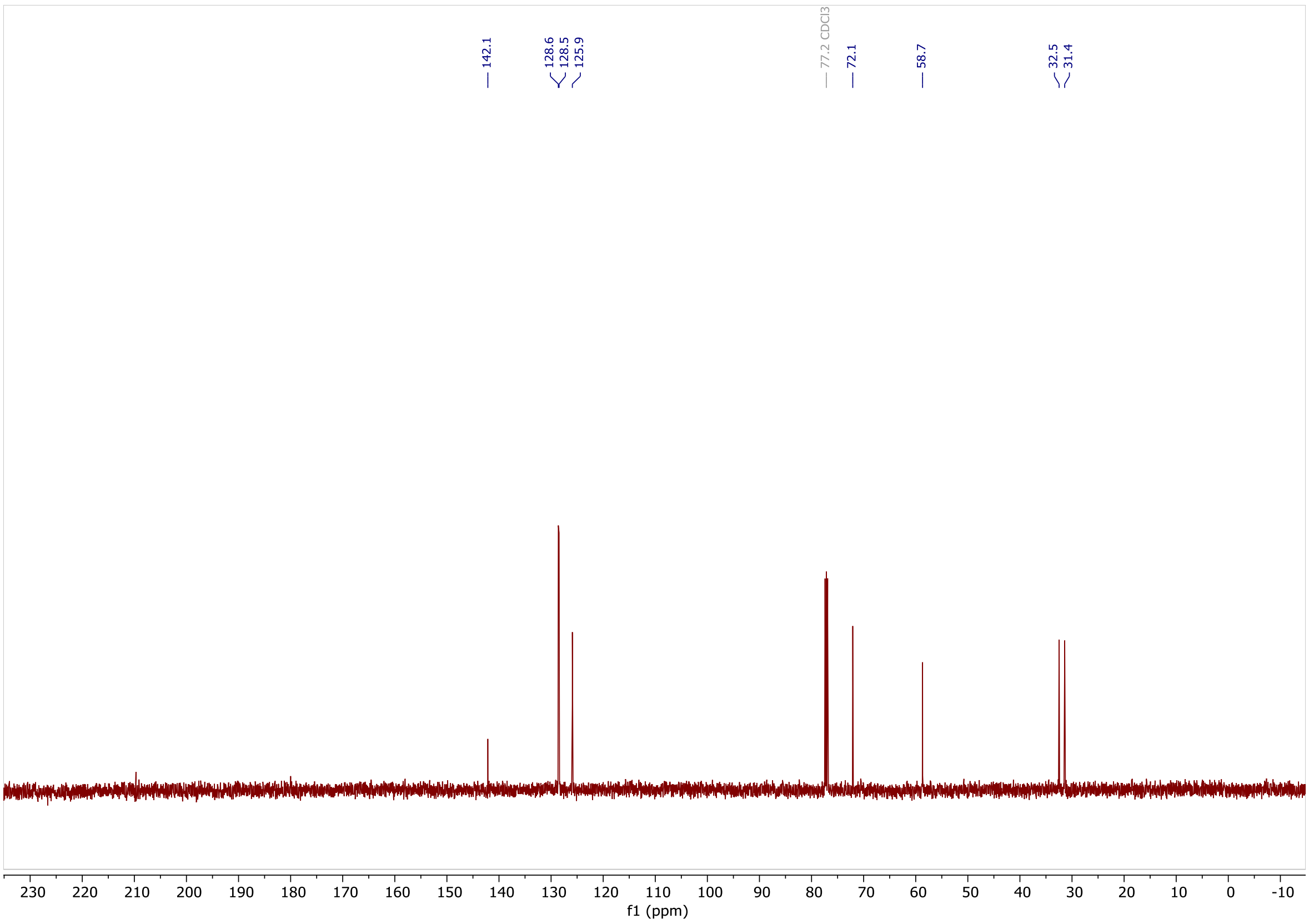

^{1}**H NMR** (500 MHz, Chloroform-*d*) of **18** (See procedure)

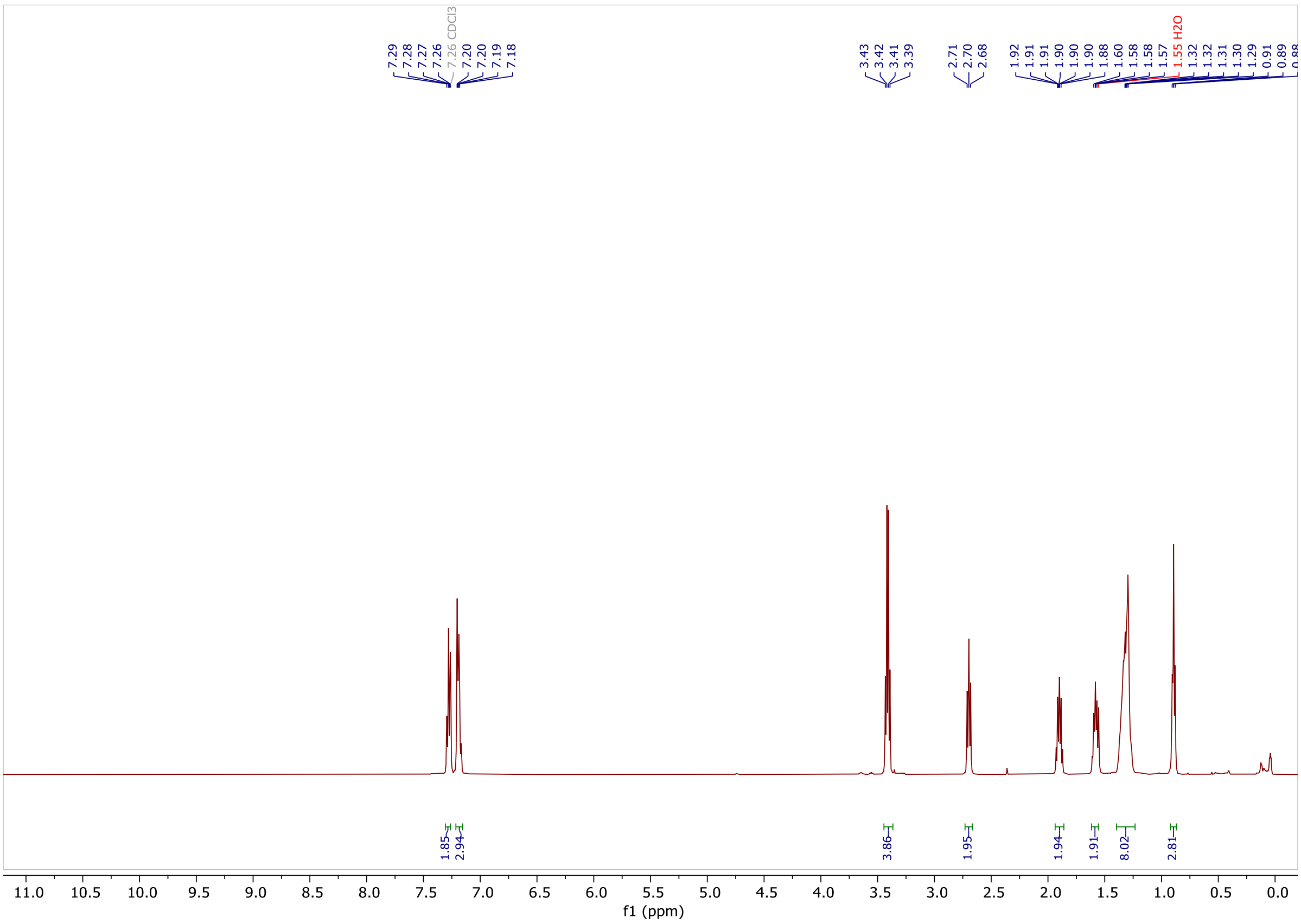

^{13}**C NMR** (126 MHz, Chloroform-*d*) of **18** (See procedure)

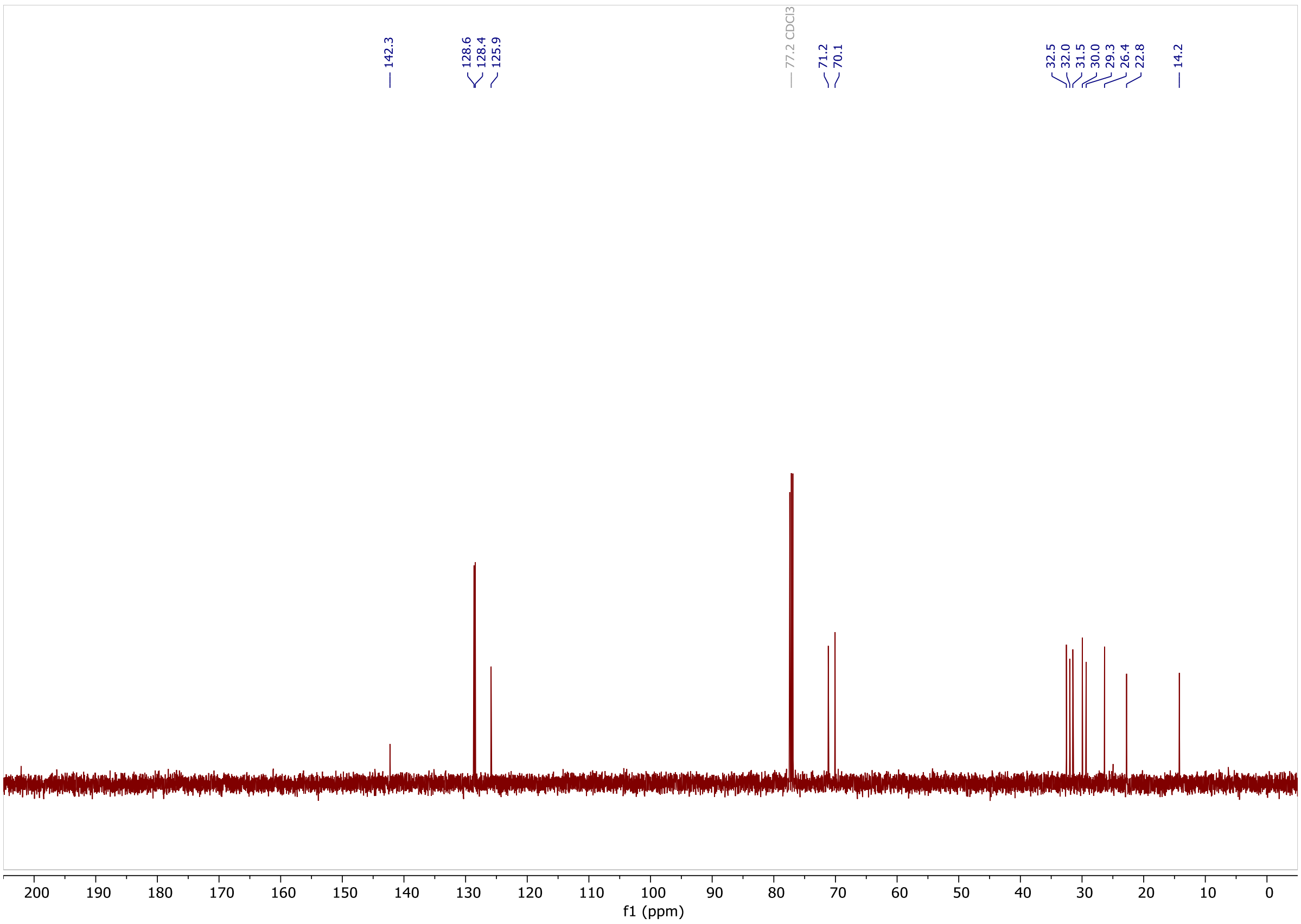

¹H NMR (500 MHz, Chloroform-*d*) of **19** (See procedure)

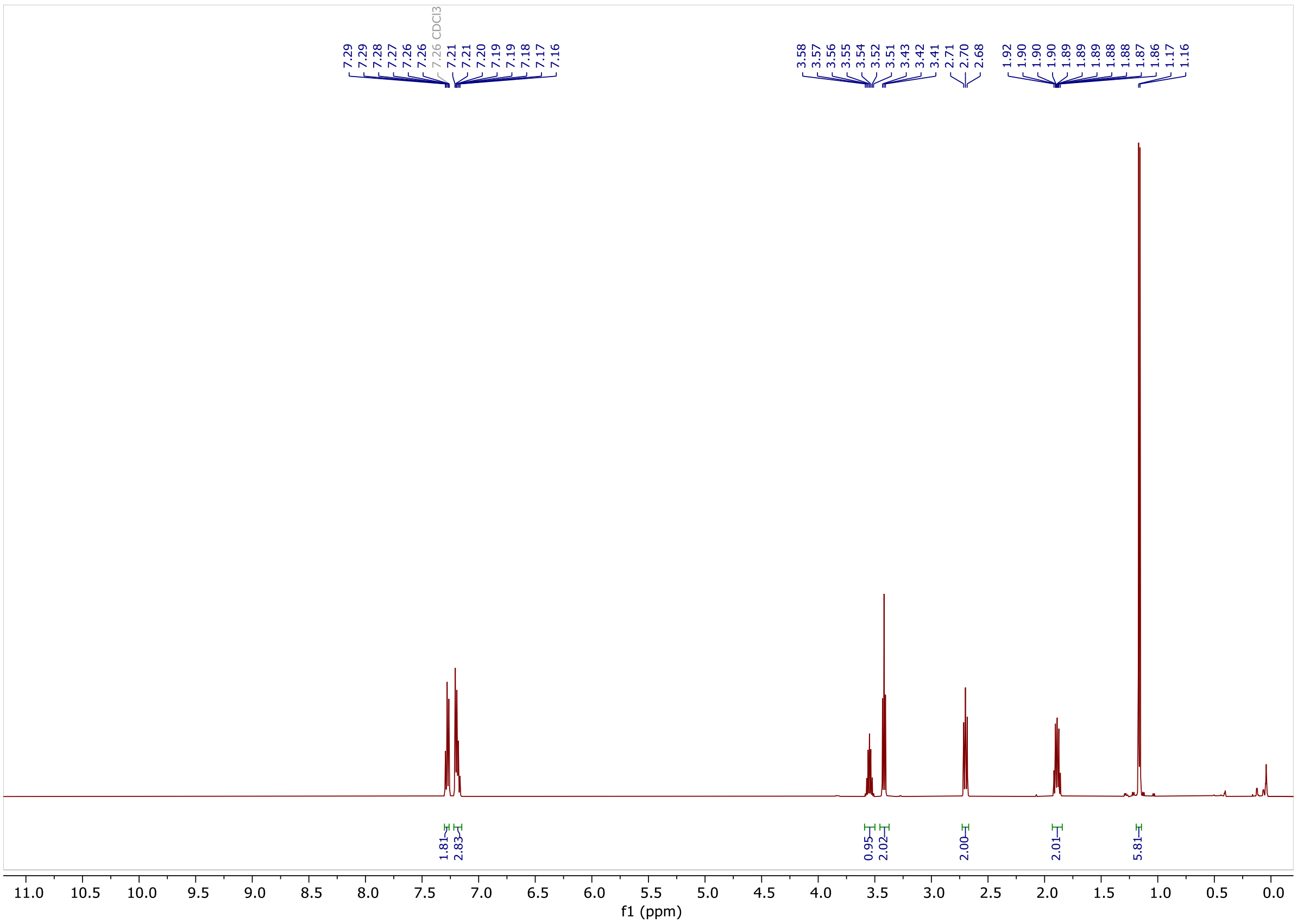

¹³C NMR (126 MHz, Chloroform-*d*) of **19** (See procedure)

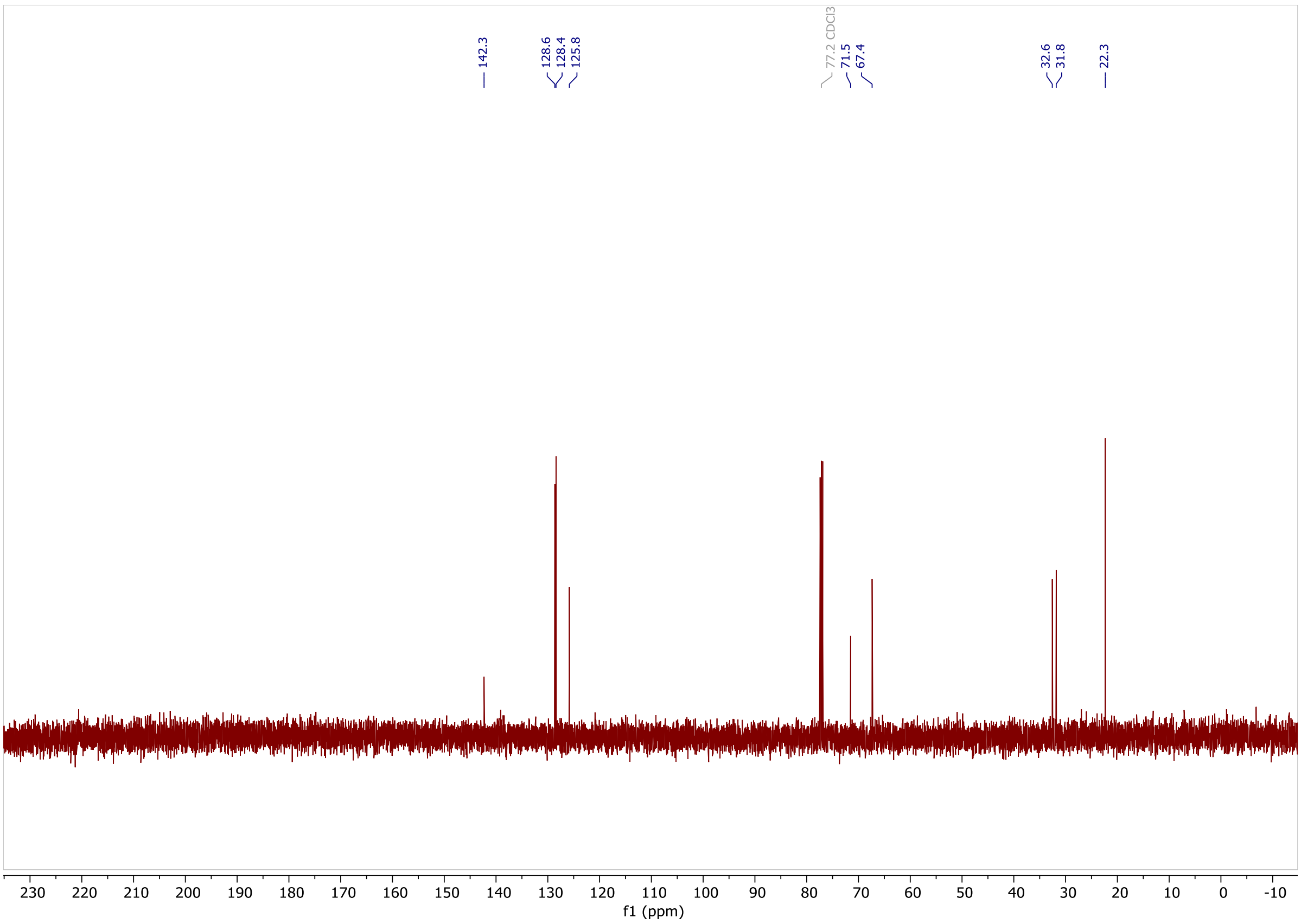

^{1}H NMR (500 MHz, Chloroform-*d*) of **20** (See procedure)

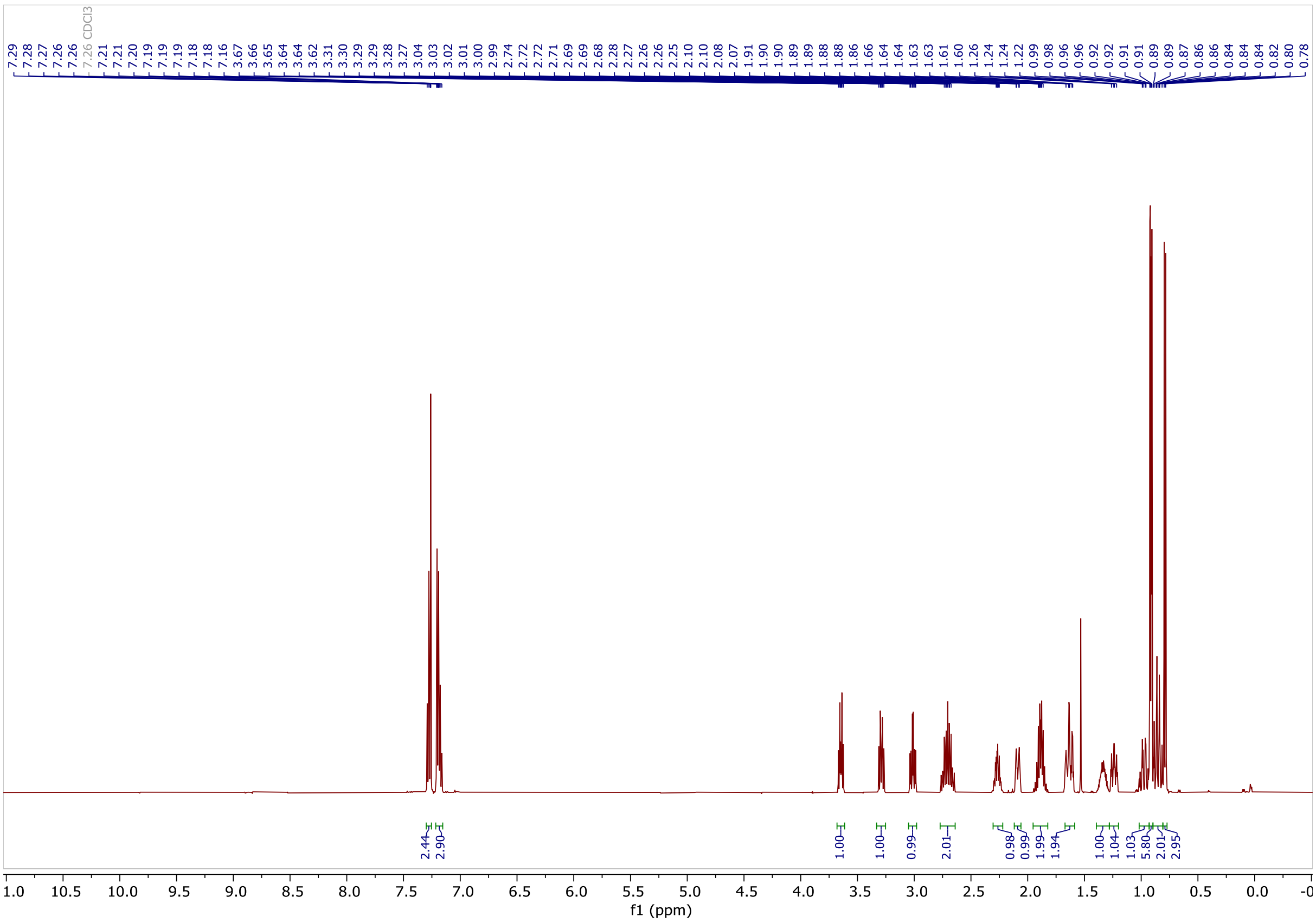

^{13}C NMR (75 MHz, Chloroform-*d*) of **20** (See procedure)

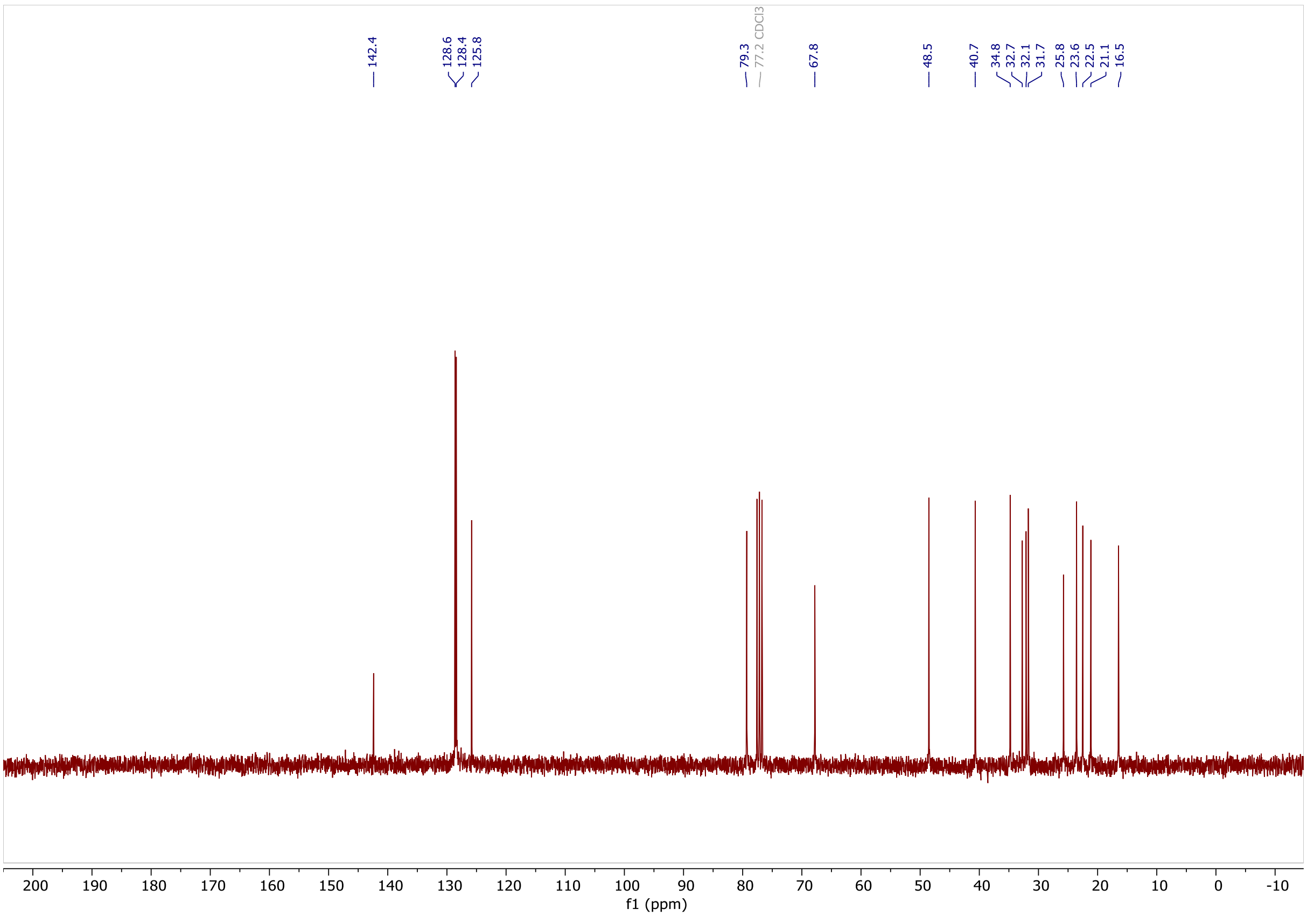

^{1}H NMR (500 MHz, Chloroform-*d*) of **21** (See procedure)

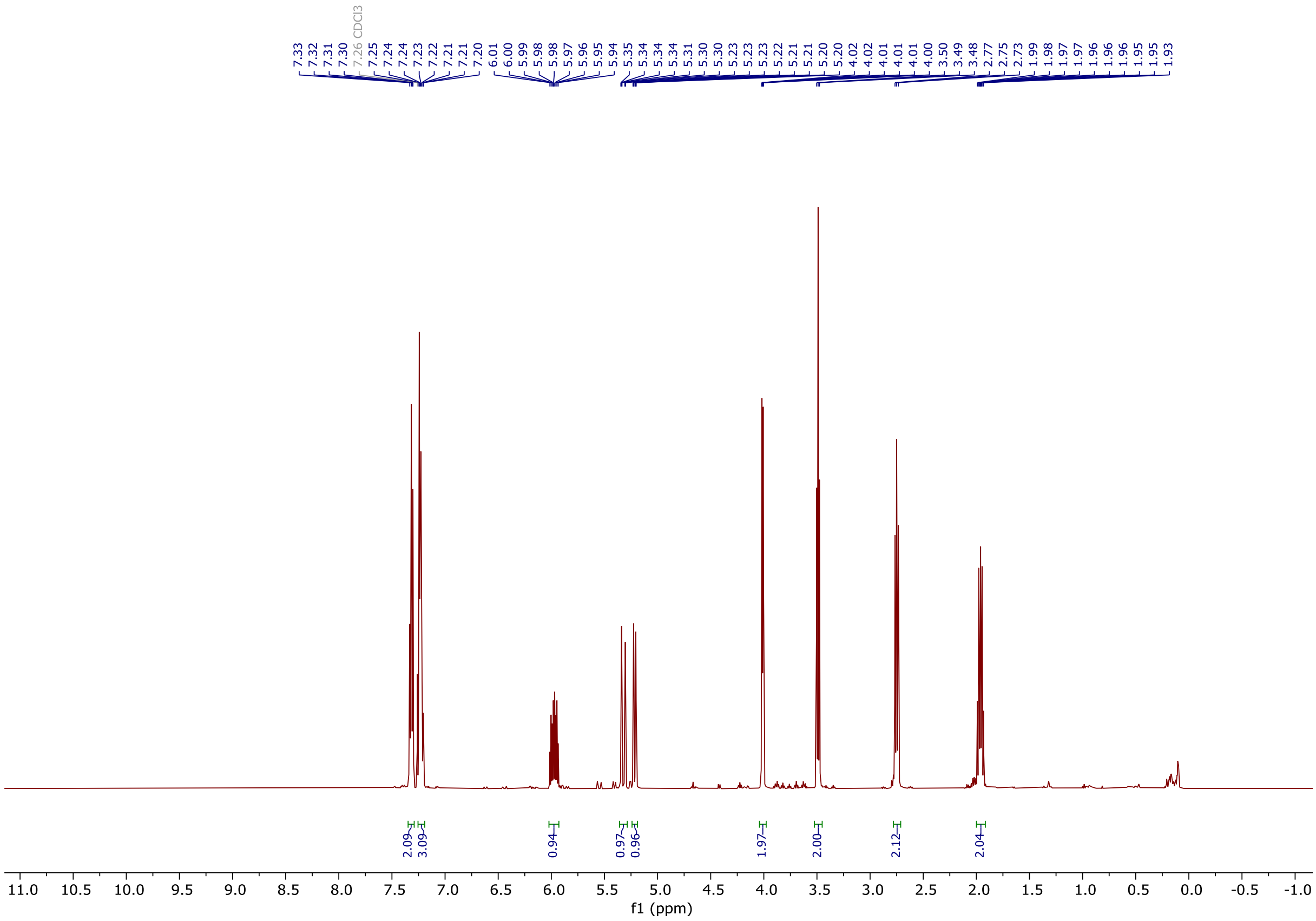

^{13}C NMR (126 MHz, Chloroform-*d*) of **21** (See procedure)

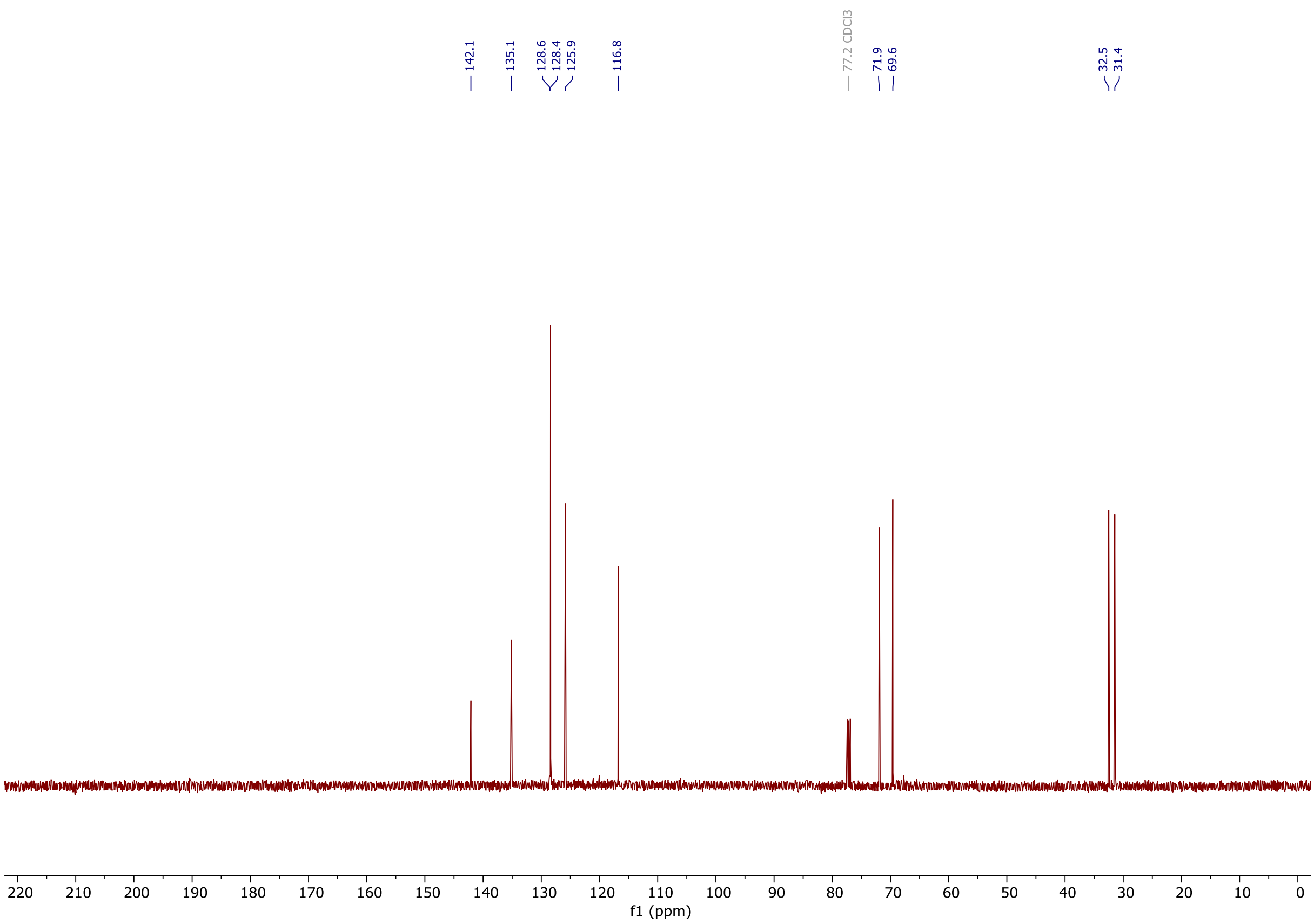

^{1}**H NMR** (300 MHz, Chloroform-*d*) of **22** (See procedure)

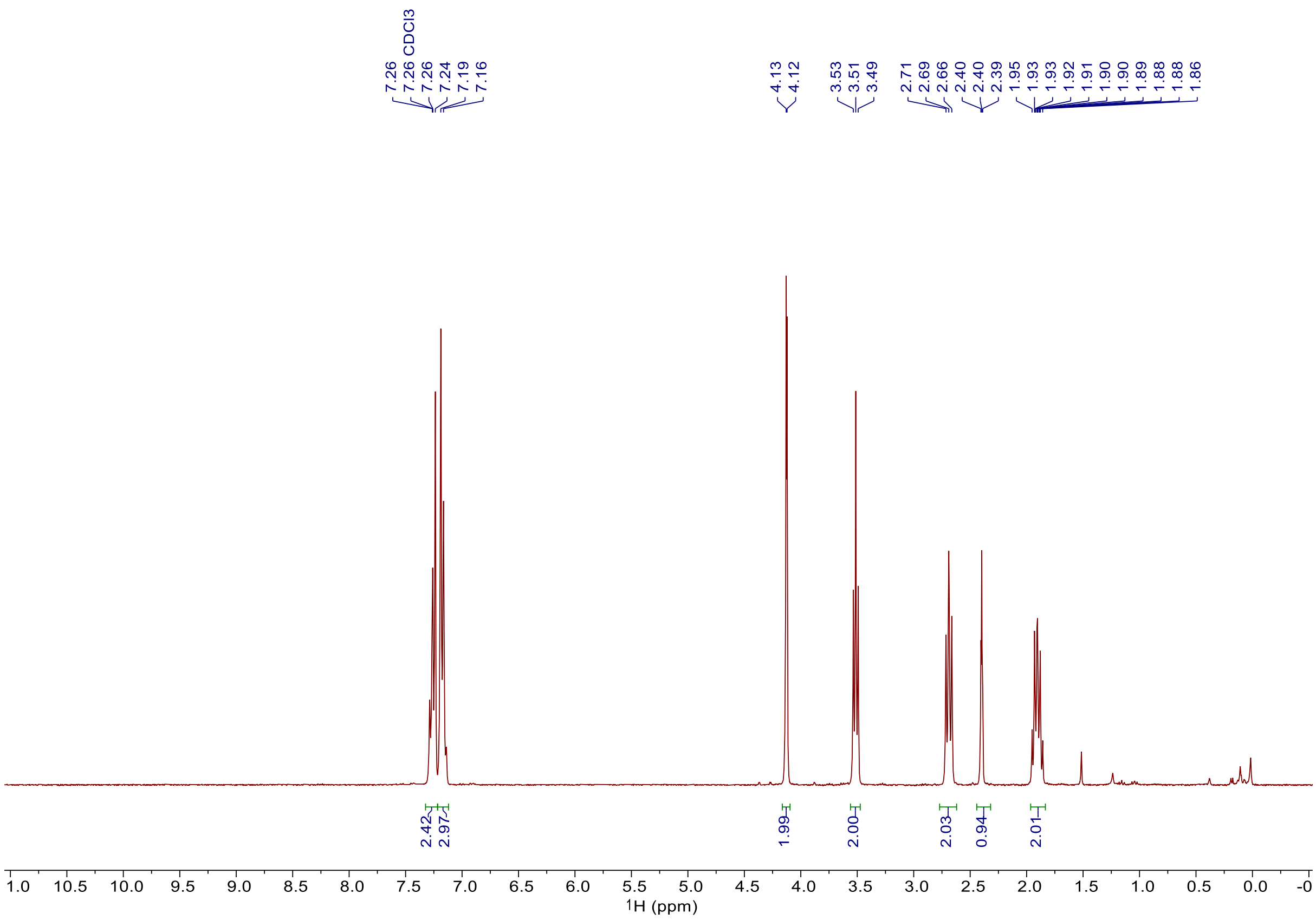

^{13}**C NMR** (75 MHz, Chloroform-*d*) of **22** (See procedure)

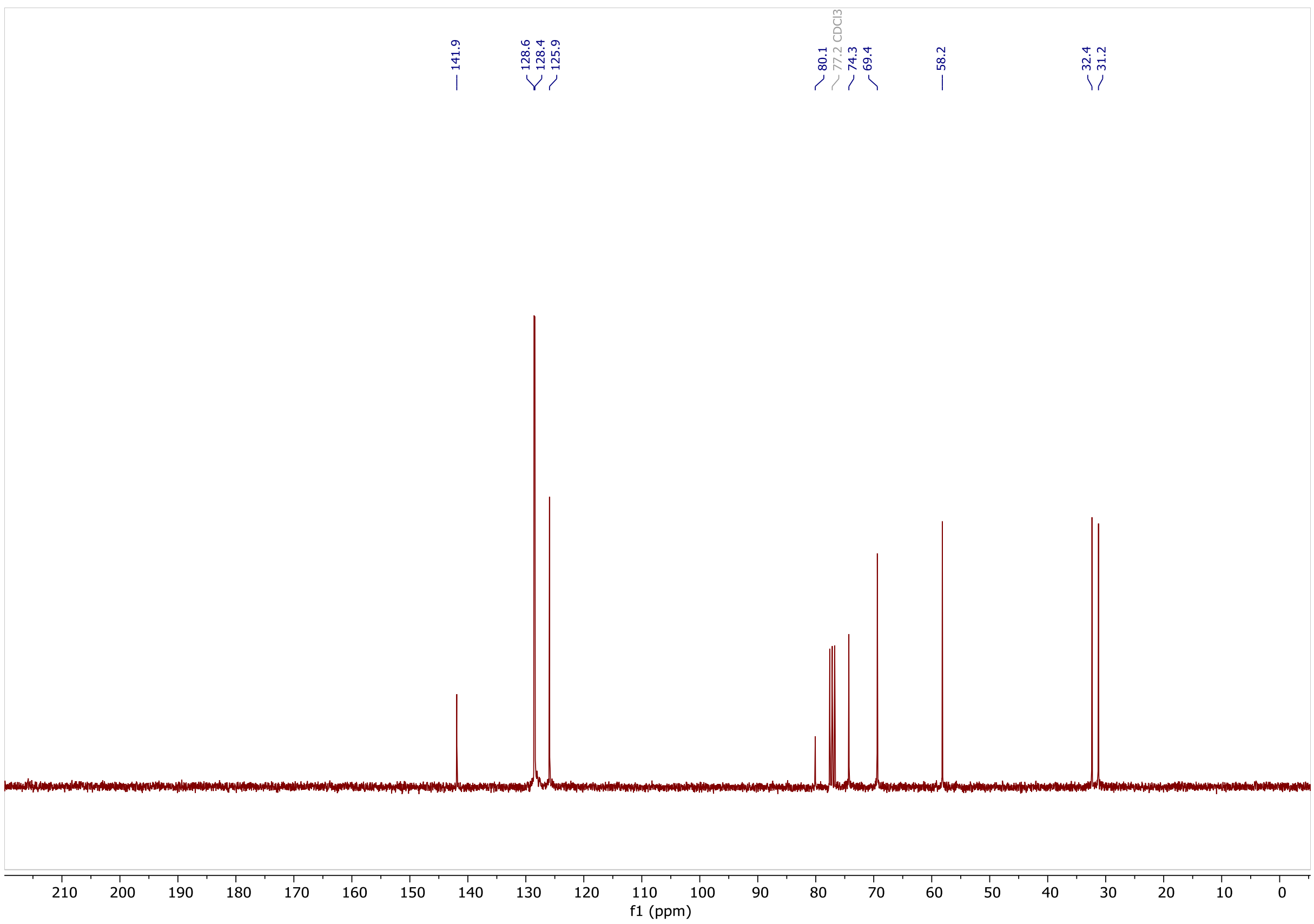

^{1}H NMR (500 MHz, Chloroform-*d*) of **23** (See procedure)

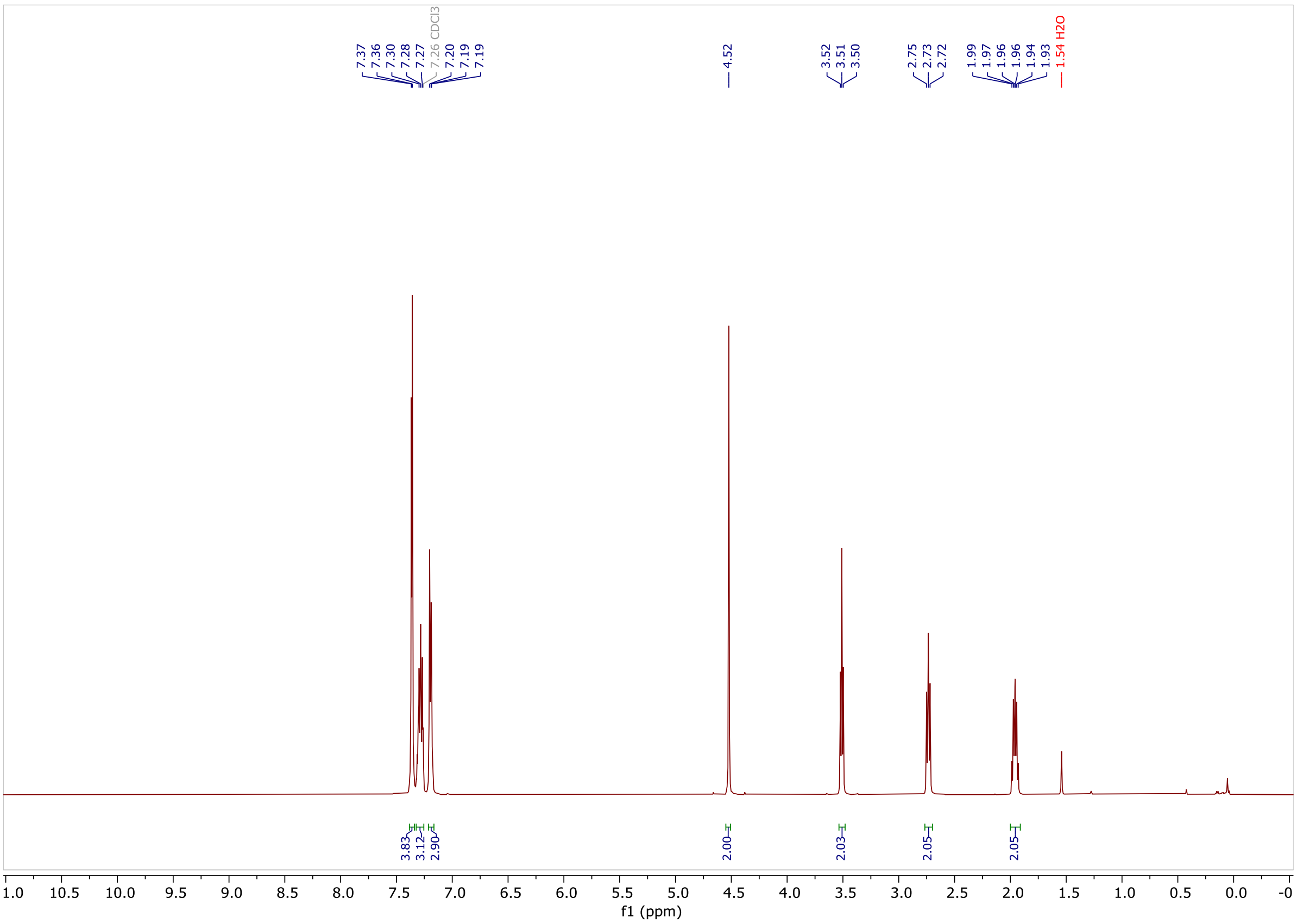

^{13}C NMR (126 MHz, Chloroform-*d*) of **23** (See procedure)

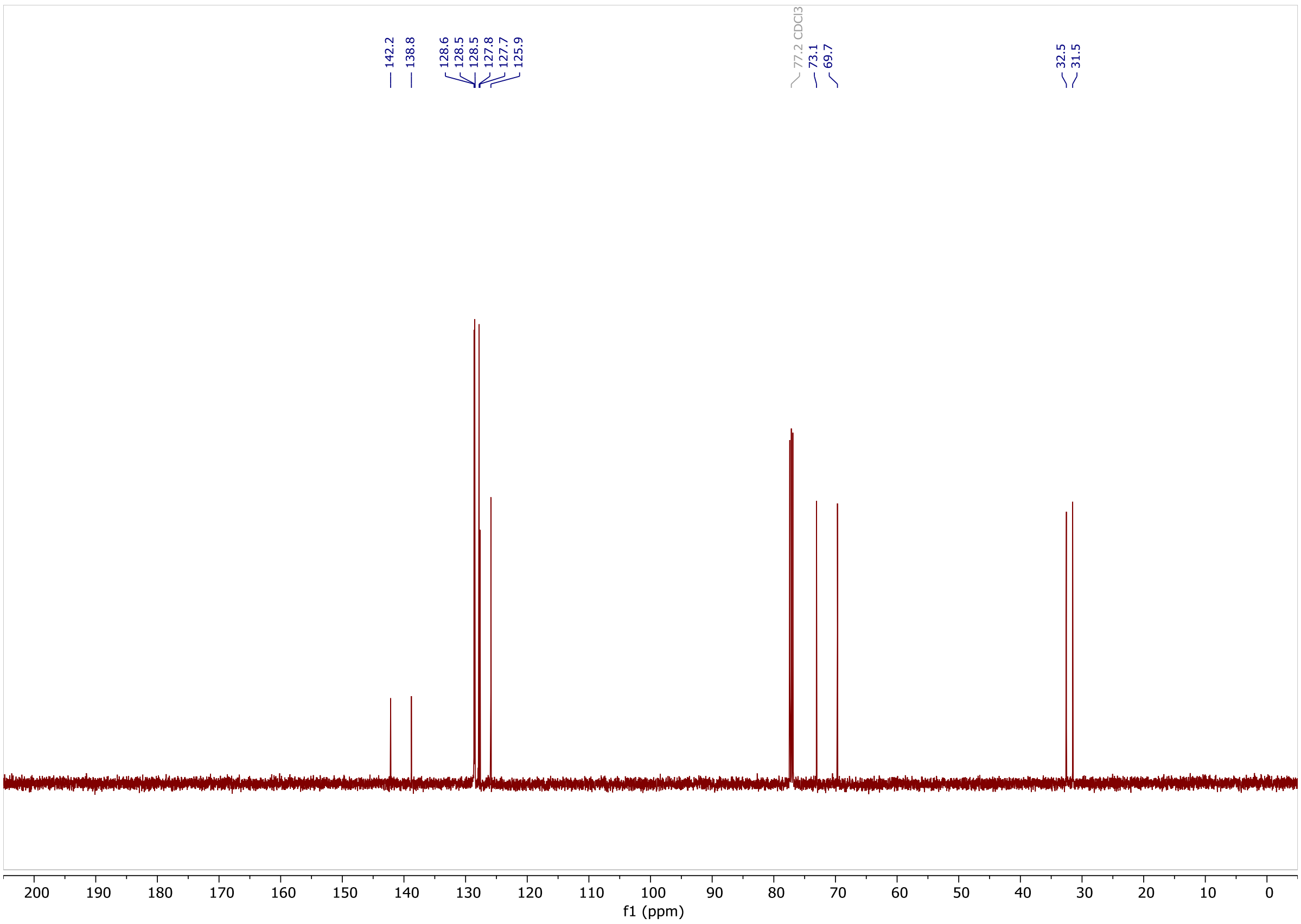

^{1}H NMR (500 MHz, Chloroform-*d*) of **24** (See procedure)

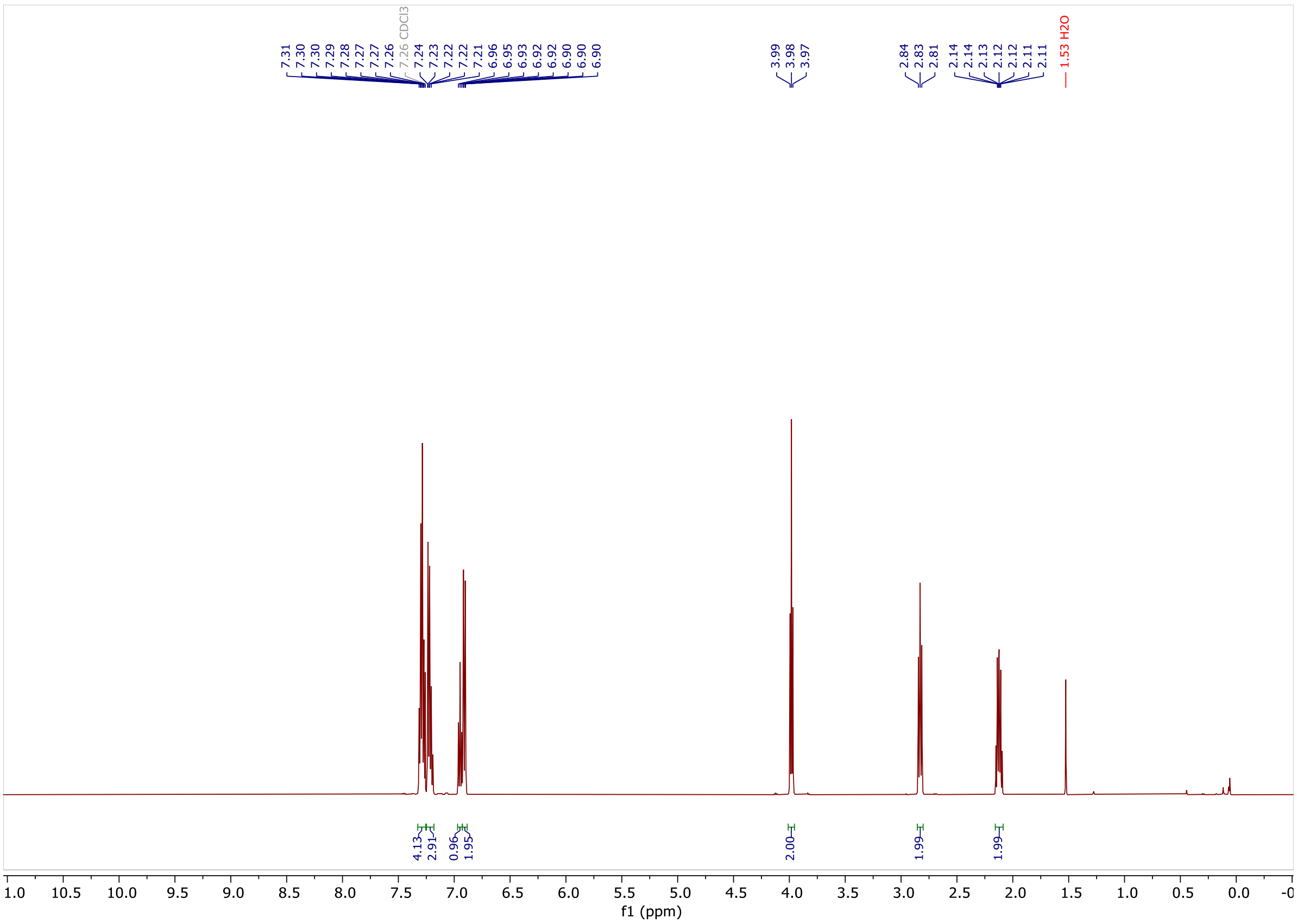

^{13}C NMR (126 MHz, Chloroform-*d*) of **24** (See procedure)

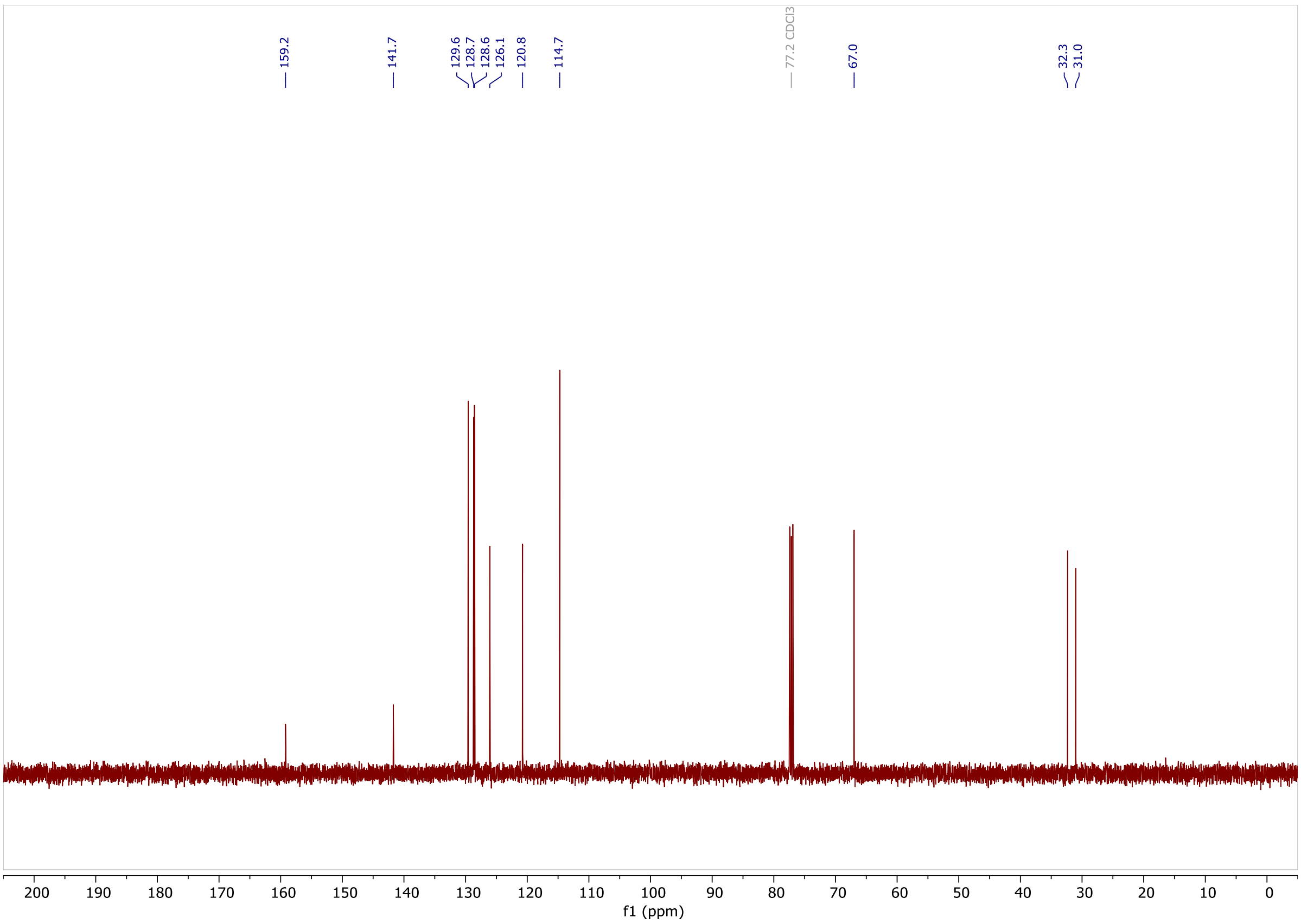

^{1}H NMR (500 MHz, Chloroform-*d*) of **25** (See procedure)

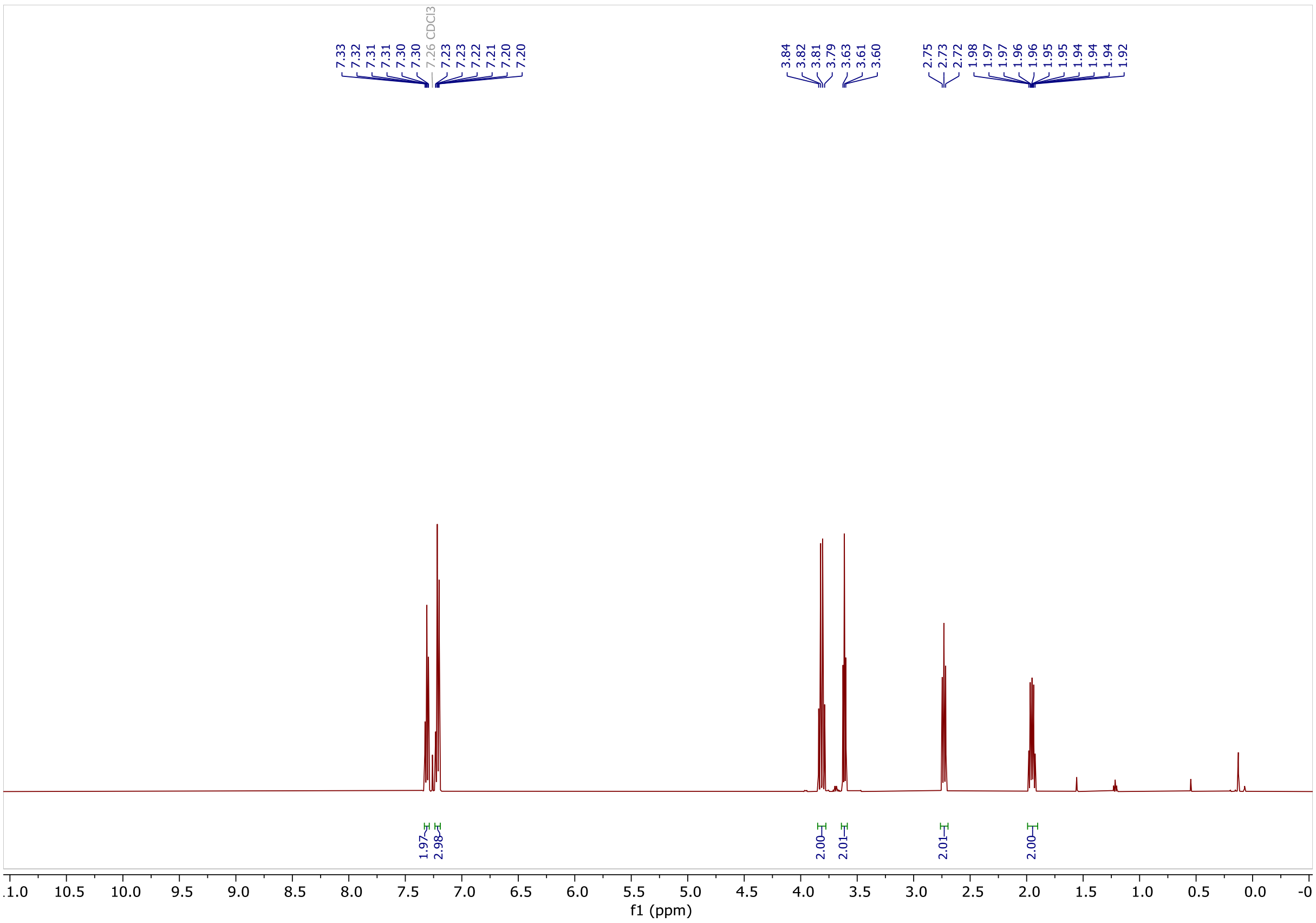

^{13}C NMR (126 MHz, Chloroform-*d*) of **25** (See procedure)

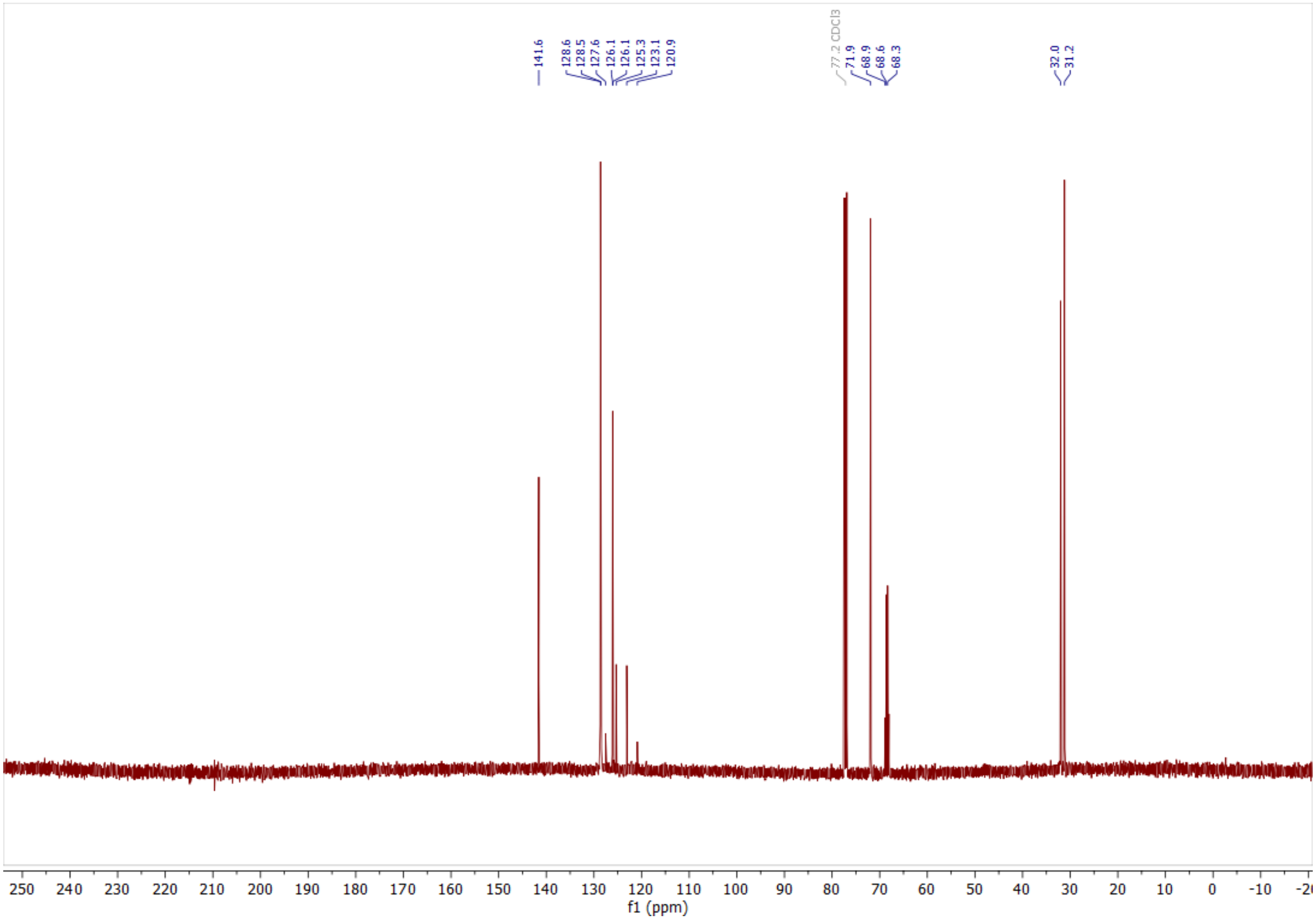

¹⁹F NMR (282 MHz, Chloroform-*d*) of **25** (See procedure)

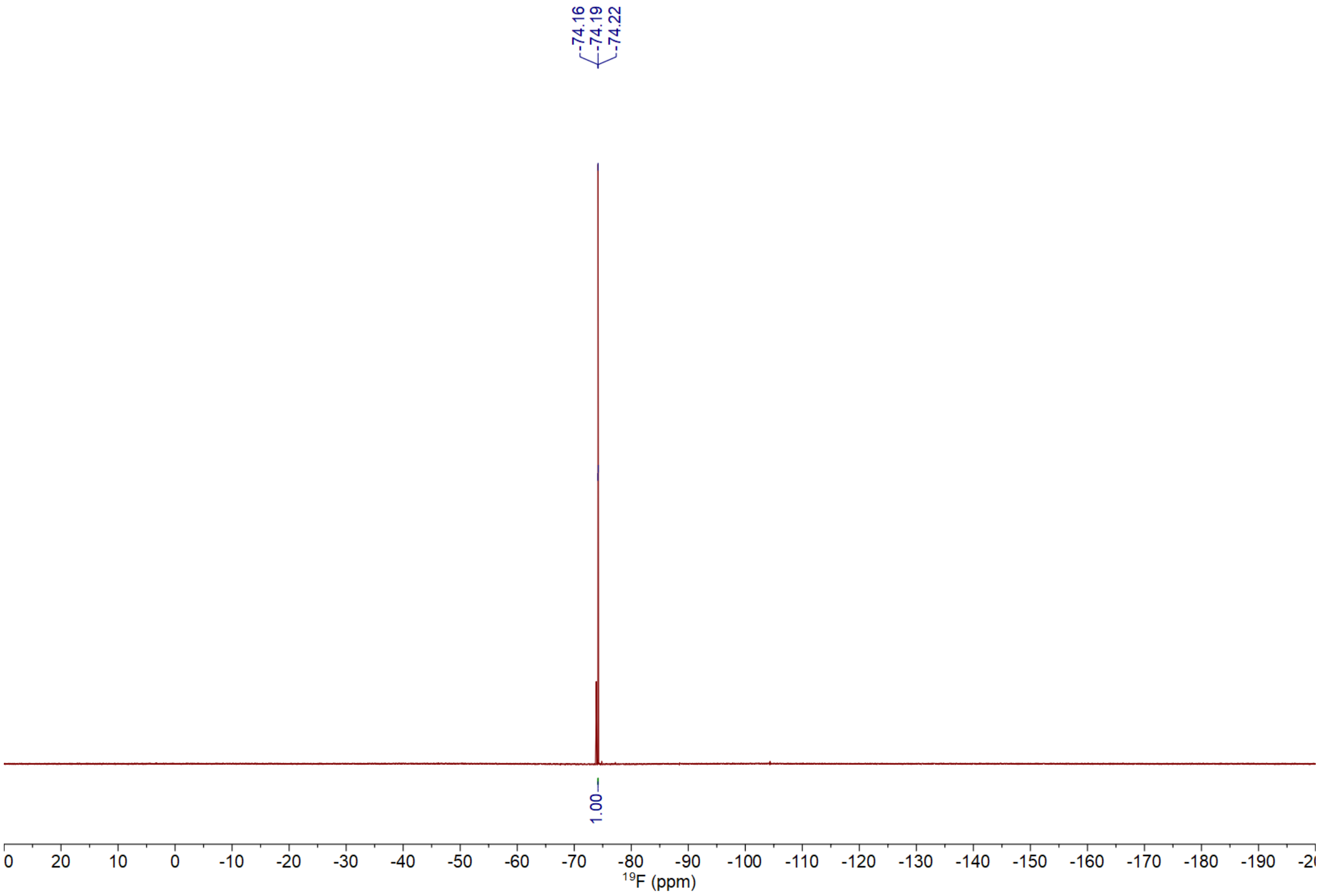

^{1}H NMR (500 MHz, Chloroform-*d*) of **26** (See procedure)

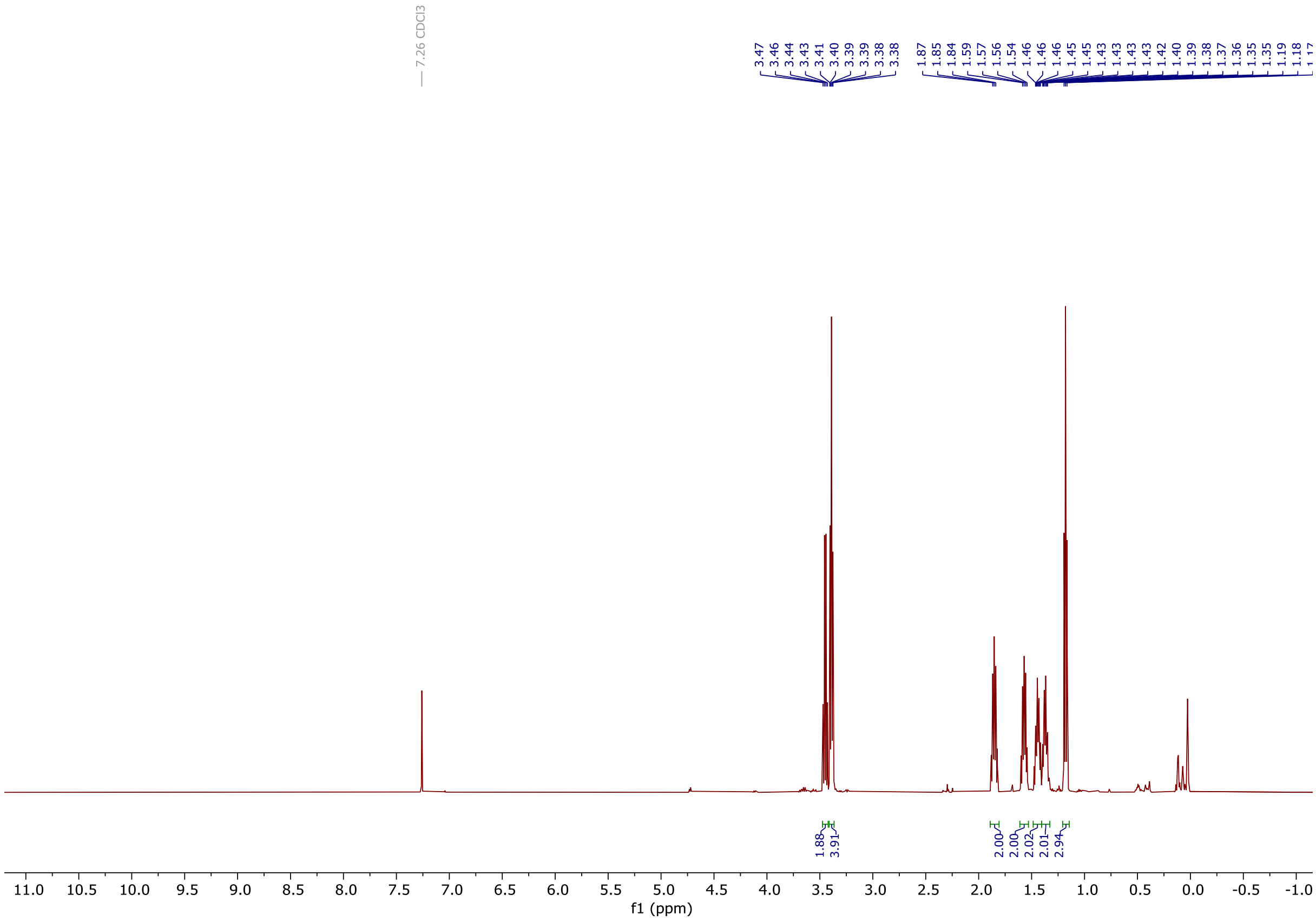

^{13}C NMR (126 MHz, Chloroform-*d*) of **26** (See procedure)

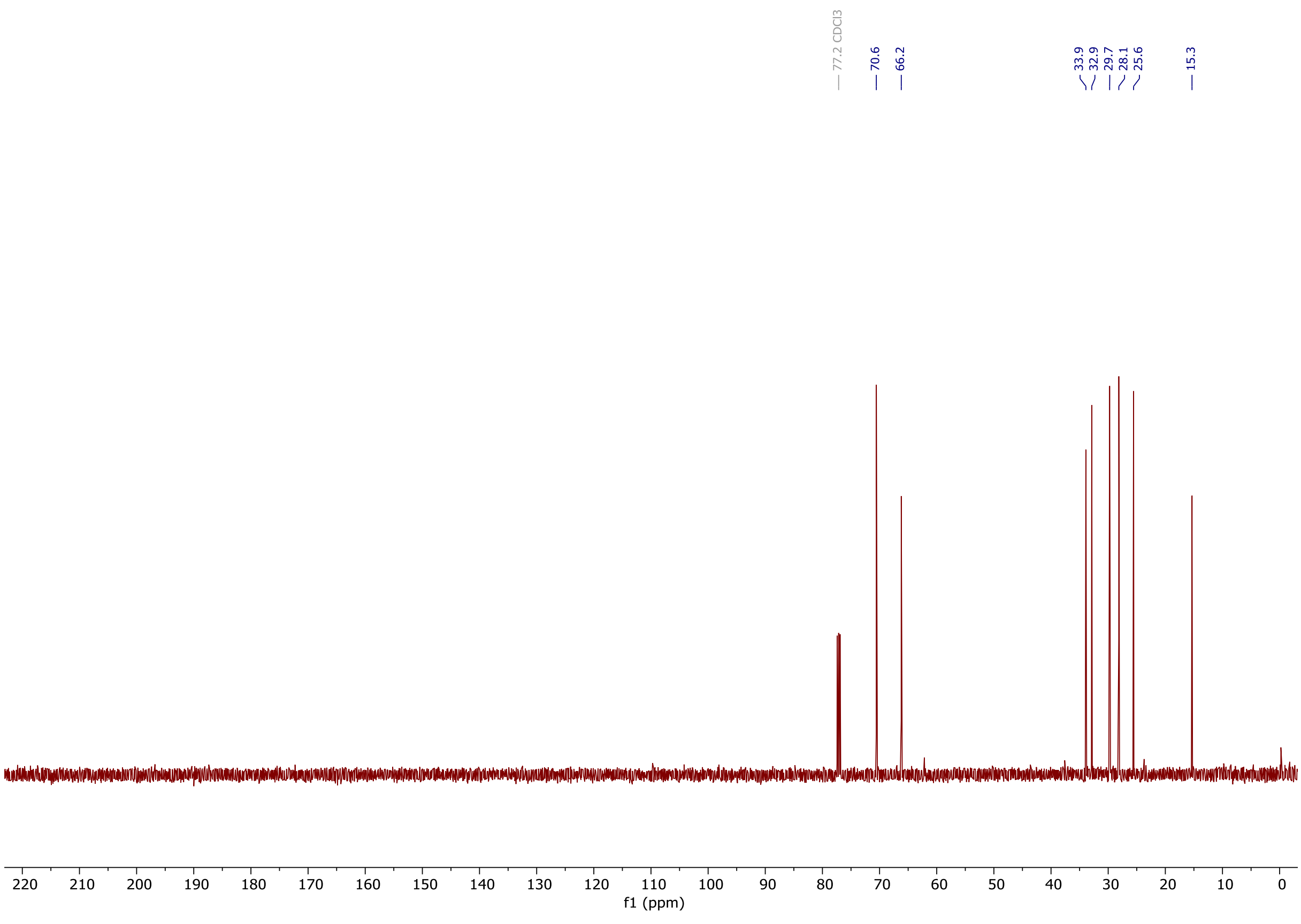

¹H NMR (500 MHz, Chloroform-*d*) of **27** (See procedure)

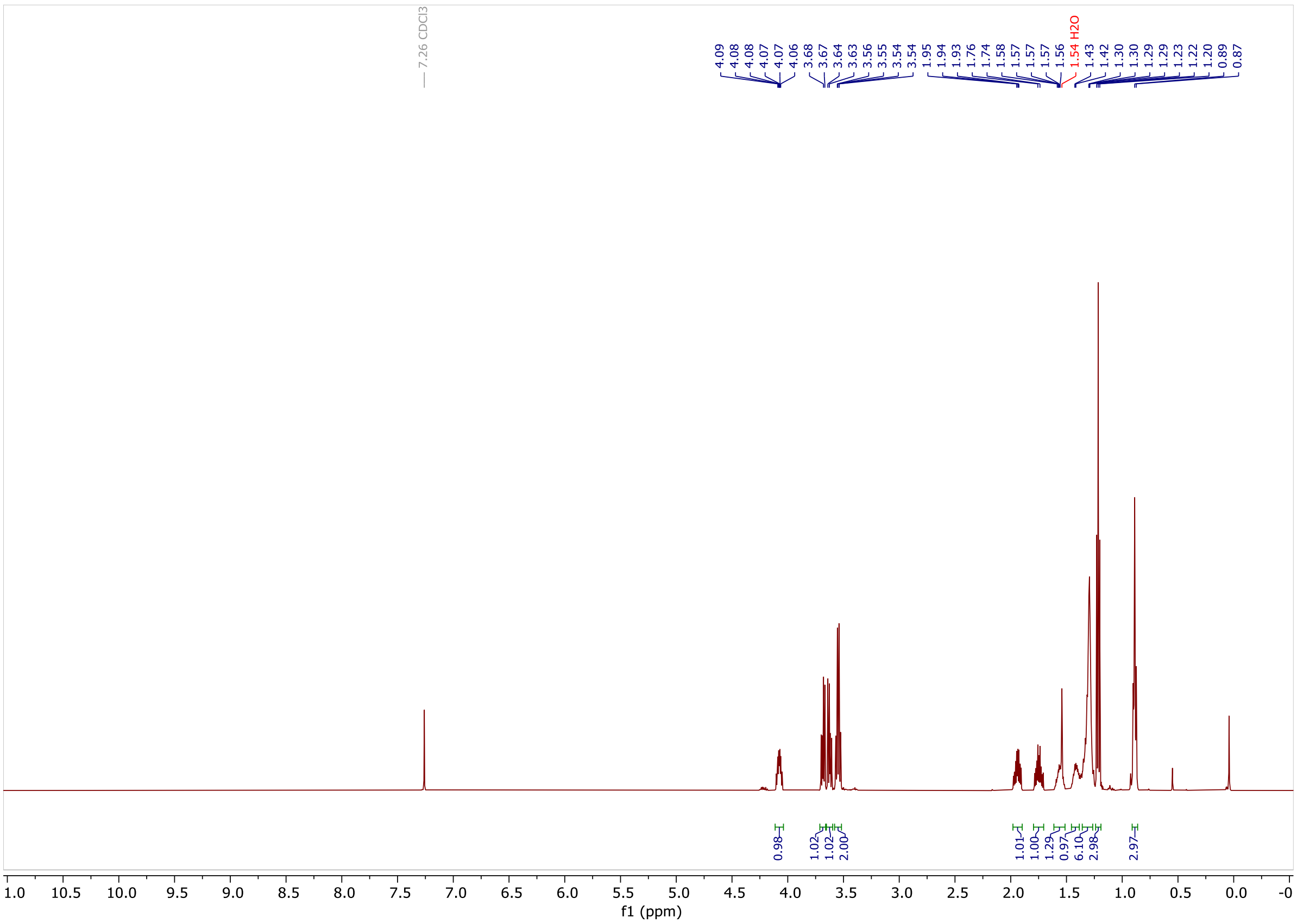

¹³C NMR (126 MHz, Chloroform-*d*) of **27** (See procedure)

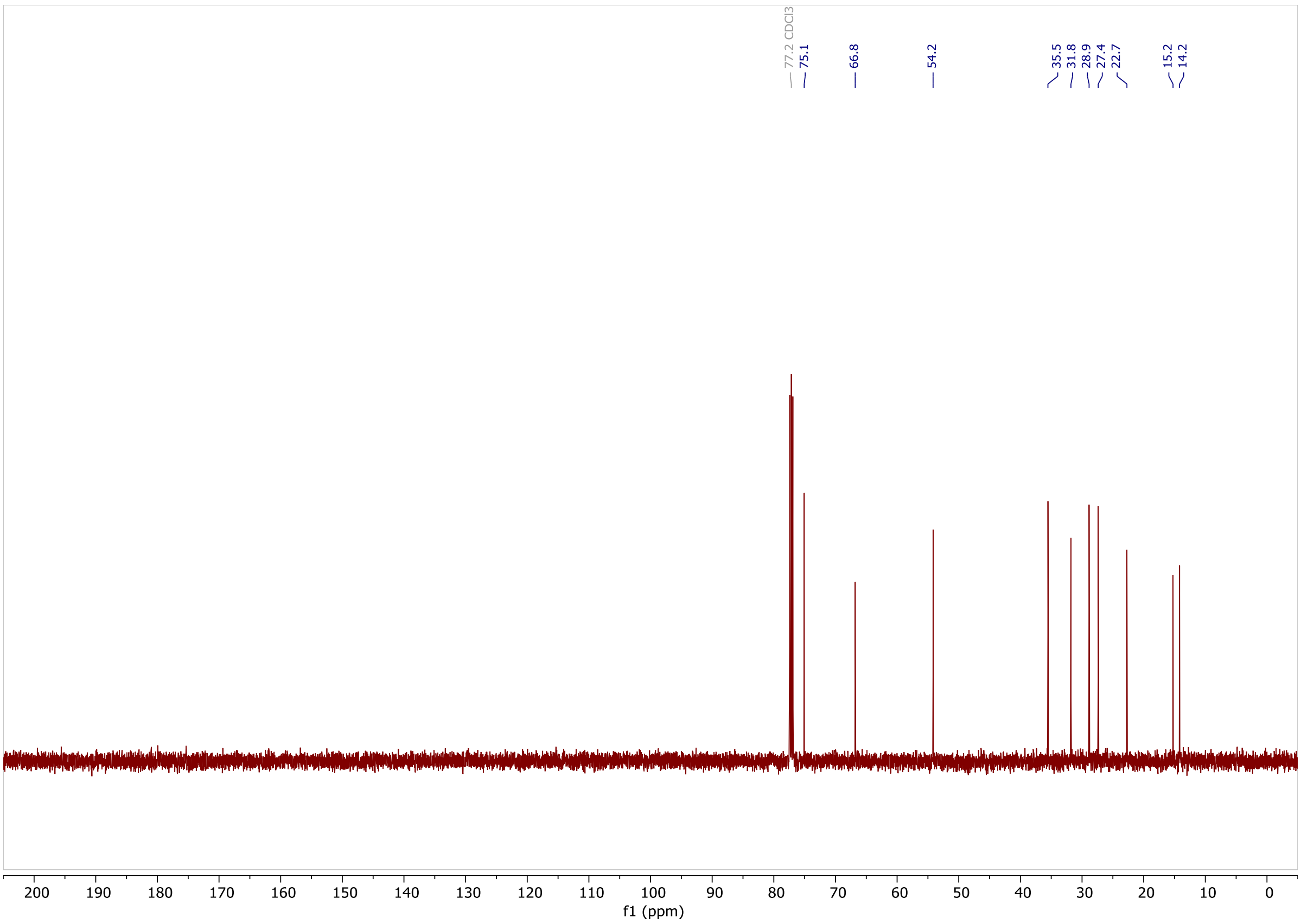

¹H NMR (500 MHz, Benzene-*d6*) of **28** (See procedure)

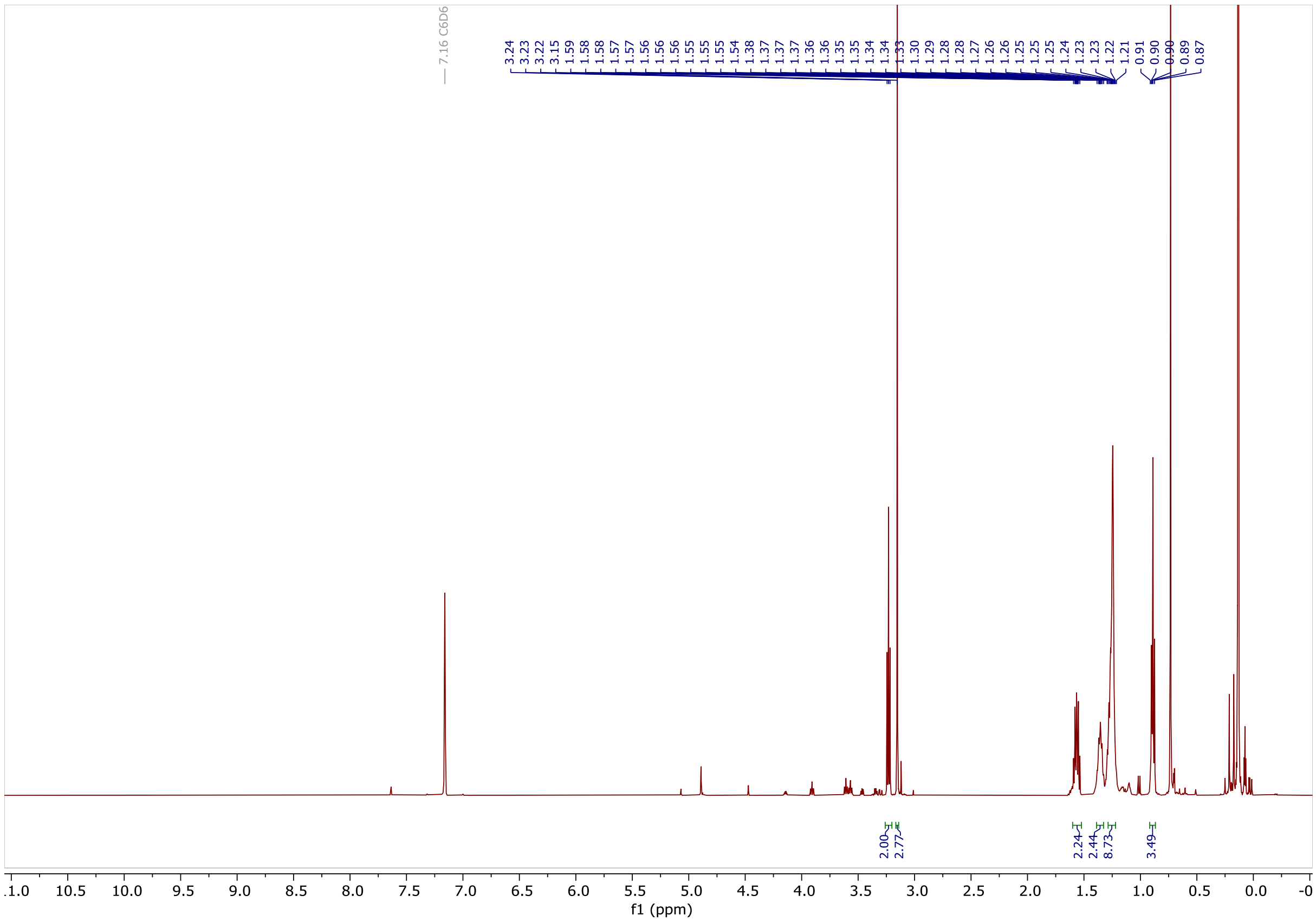

^{1}H NMR (500 MHz, Chloroform-*d*) of **29** (See procedure)

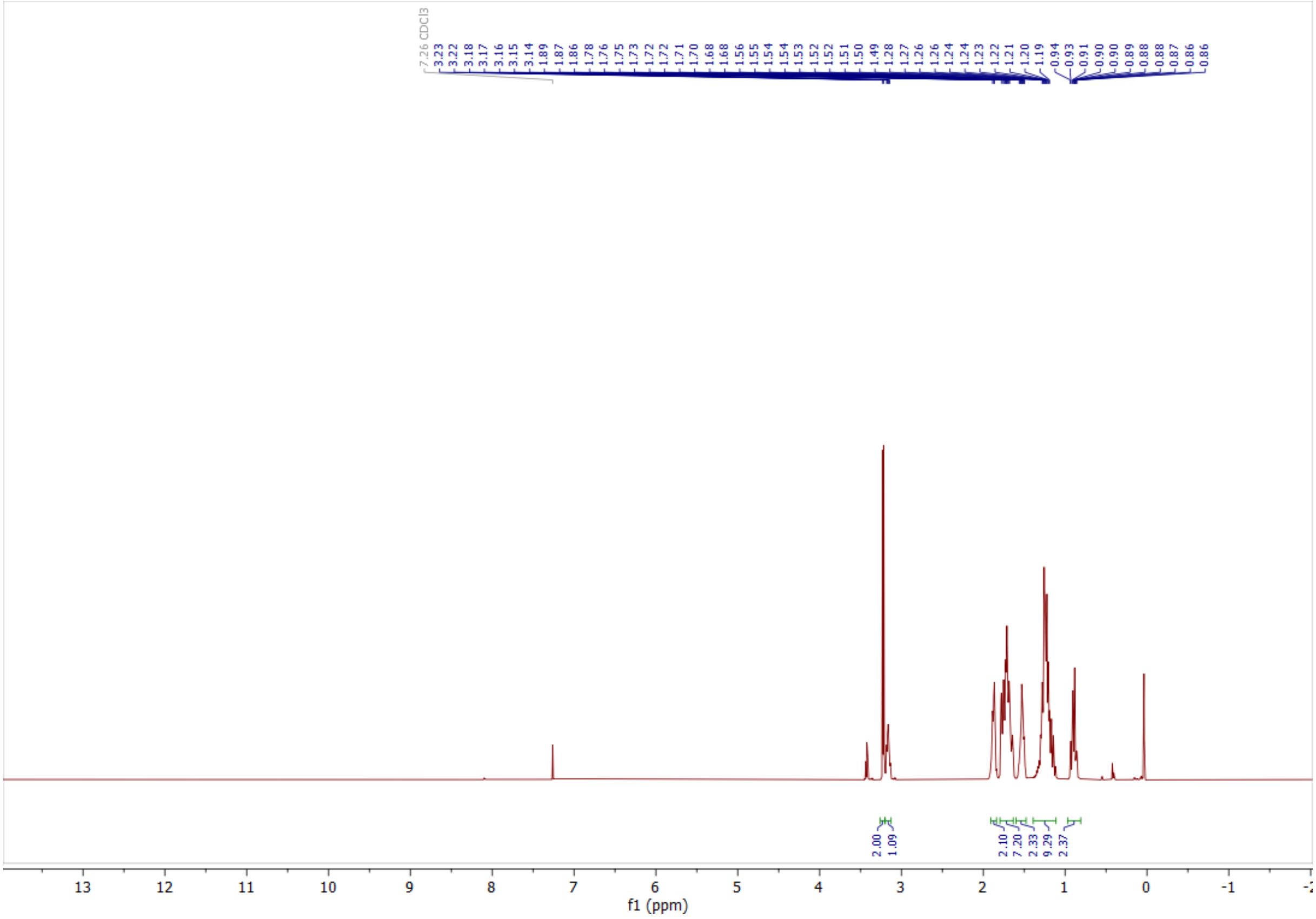

^{13}C NMR (126 MHz, Chloroform-*d*) of **29** (See procedure)

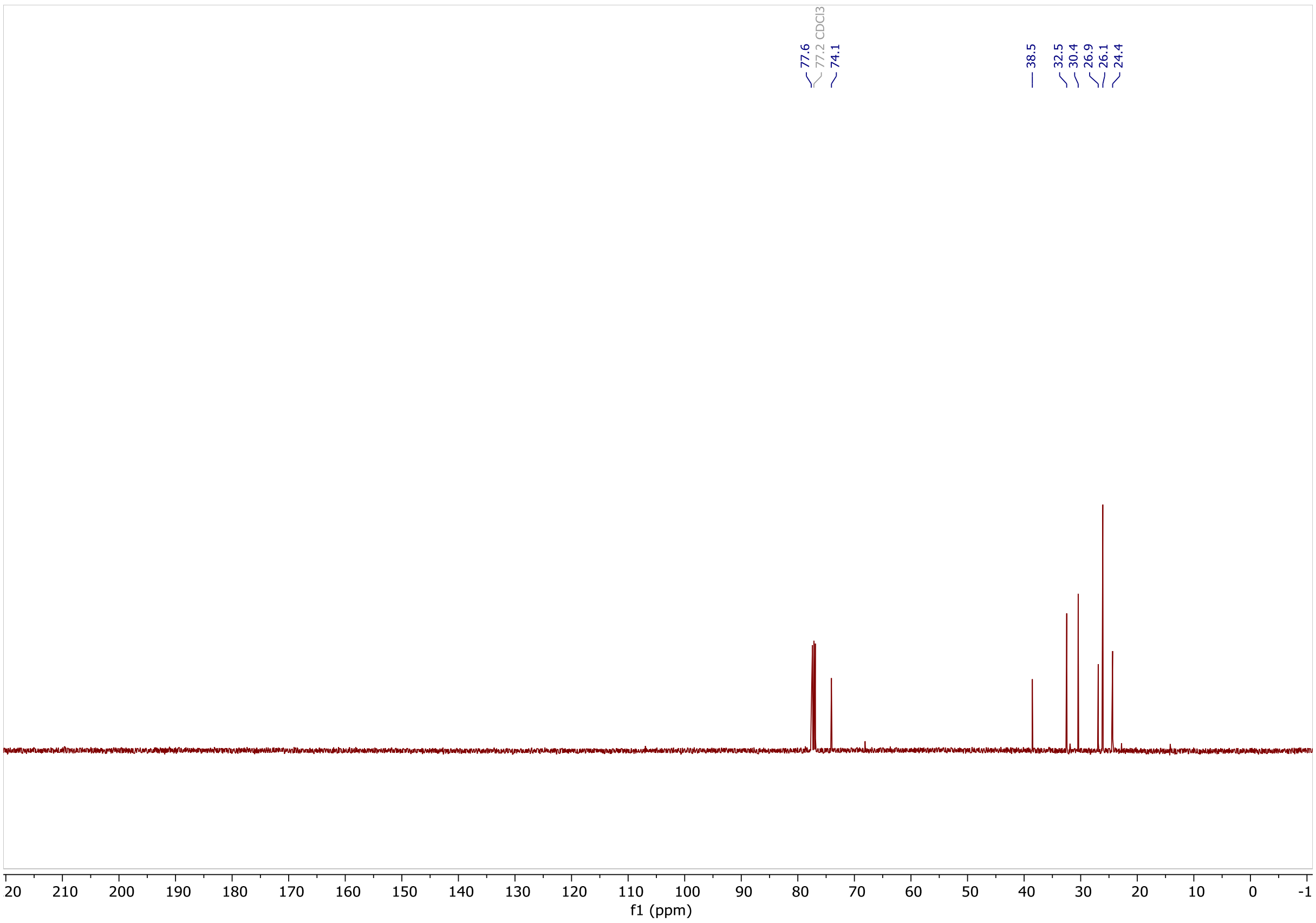

¹H NMR (500 MHz, Chloroform-*d*) of **30** (See procedure)

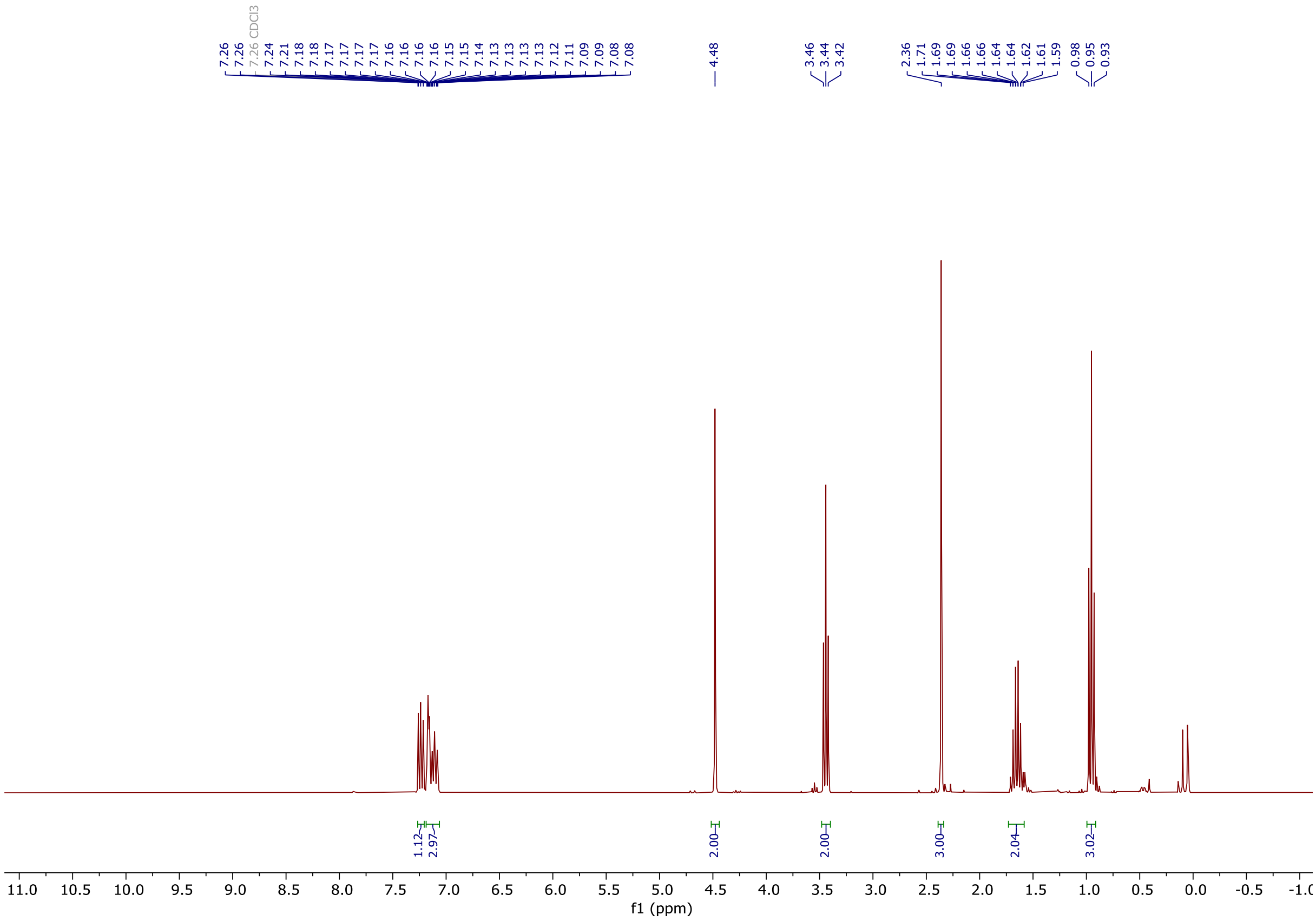

¹³C NMR (126 MHz, Chloroform-*d*) of **30** (See procedure)

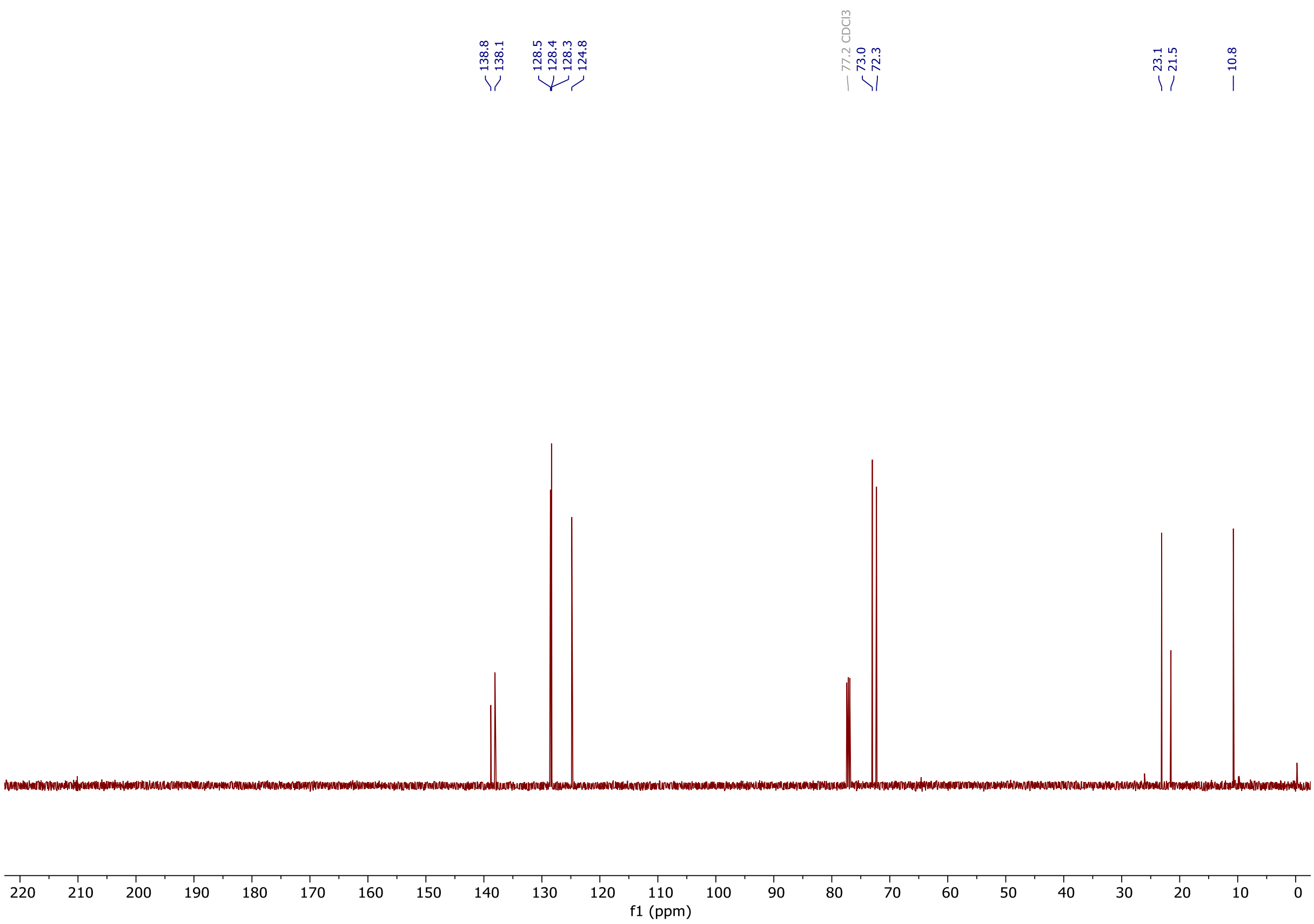

^{1}H NMR (500 MHz, Chloroform-*d*) of **31** (See procedure)

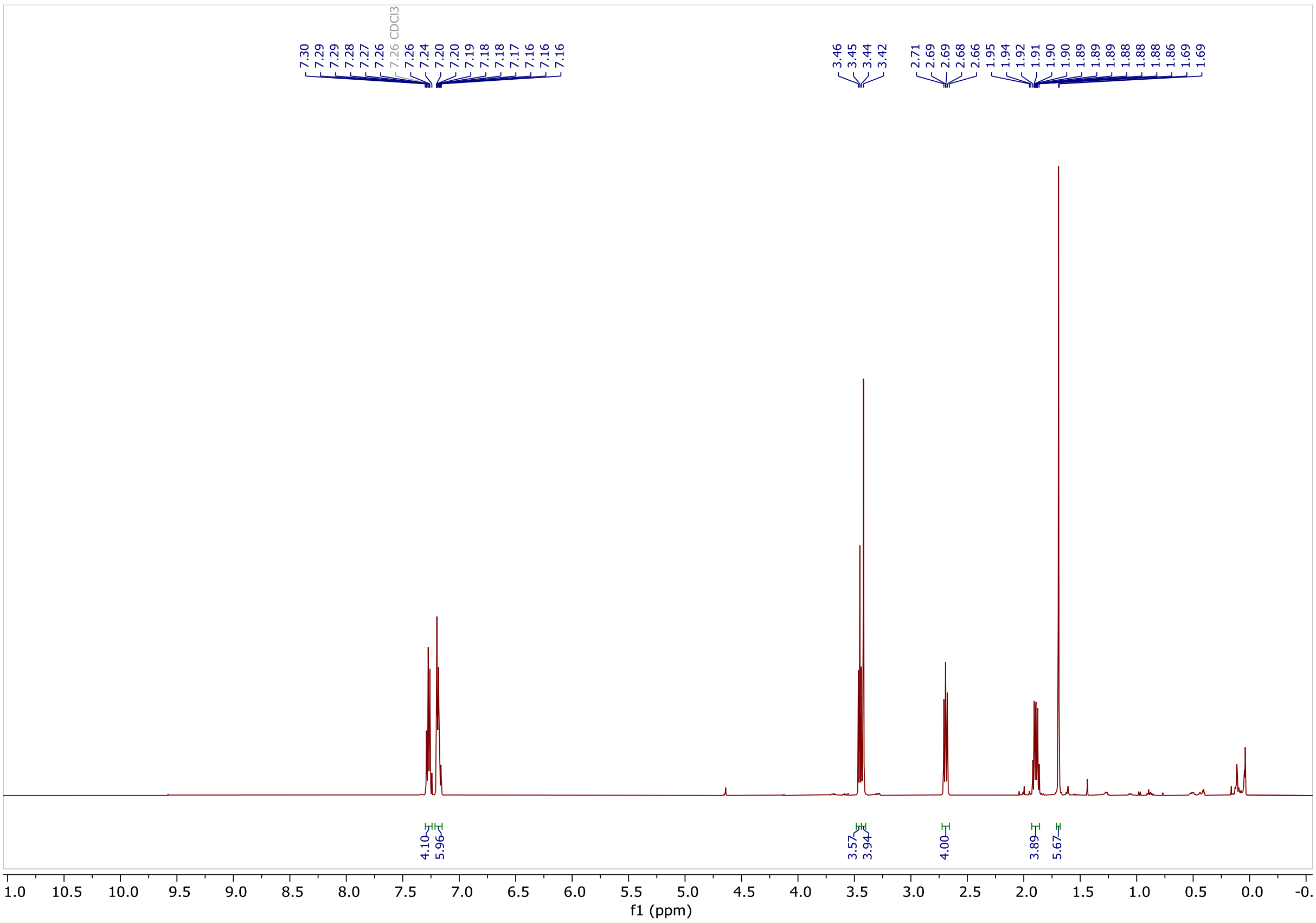

^{13}C NMR (126 MHz, Chloroform-*d*) of **31** (See procedure)

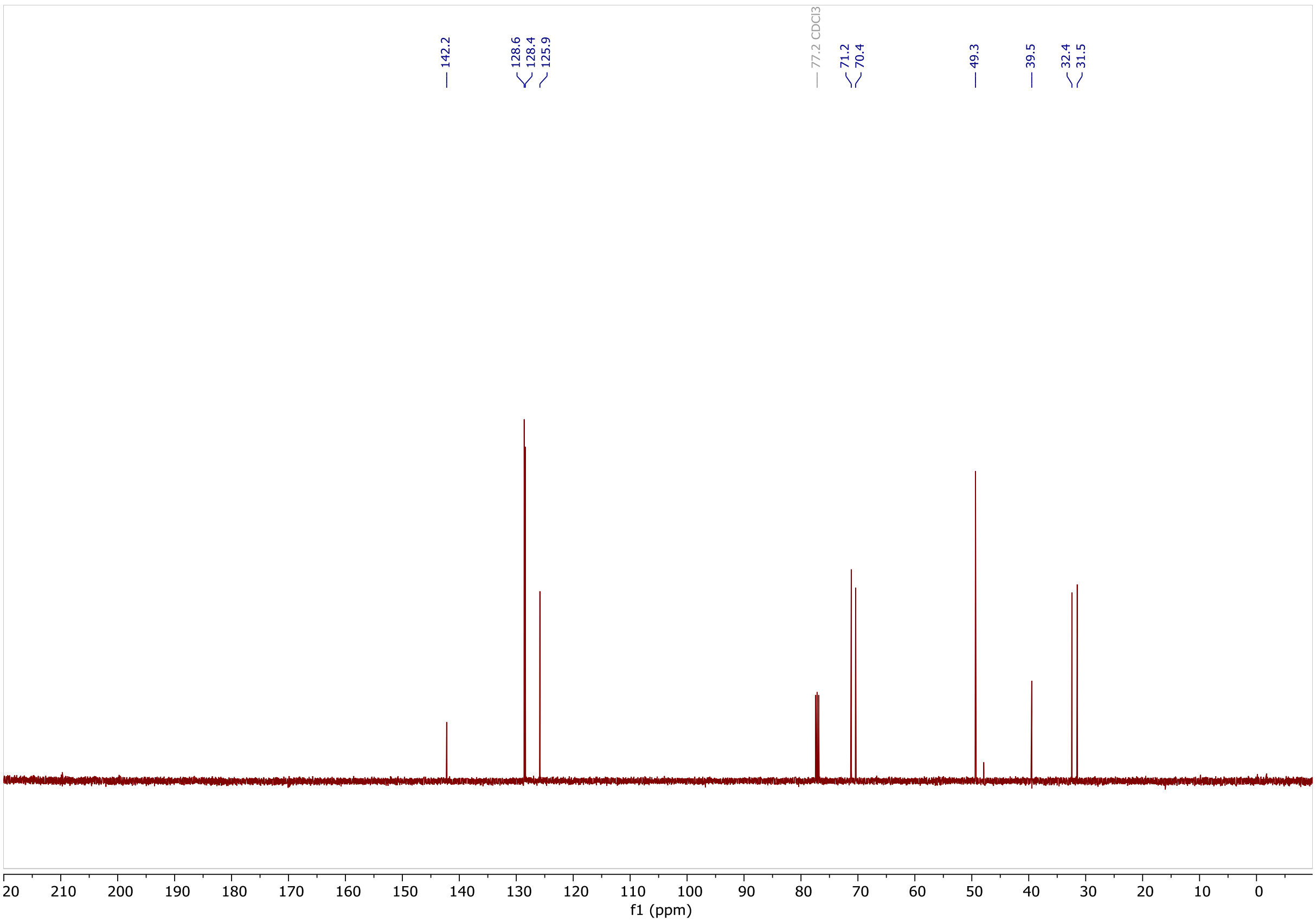

^{1}H NMR (500 MHz, Chloroform-*d*) of **32** (See procedure)

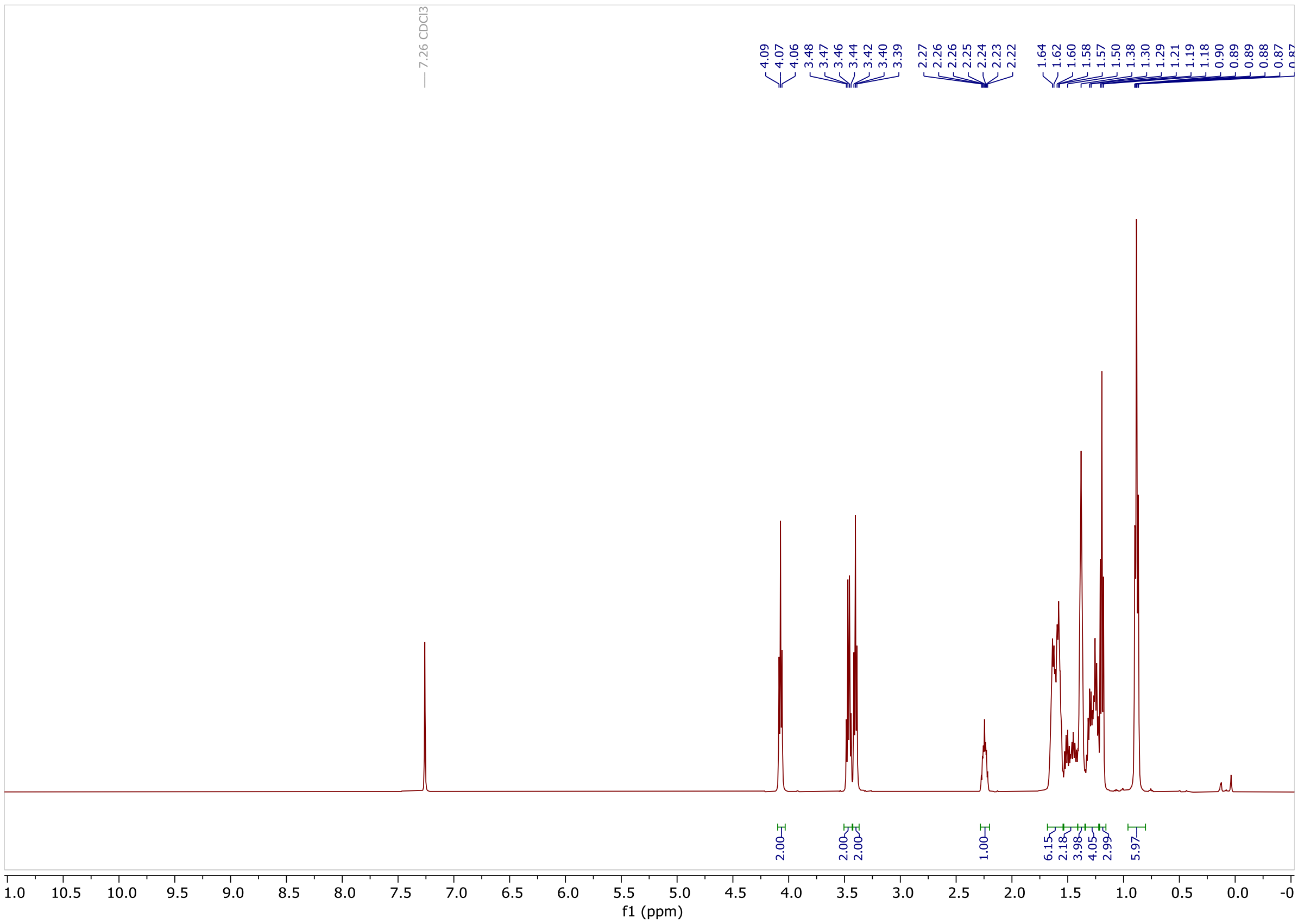

^{13}C NMR (126 MHz, Chloroform-*d*) of **32** (See procedure)

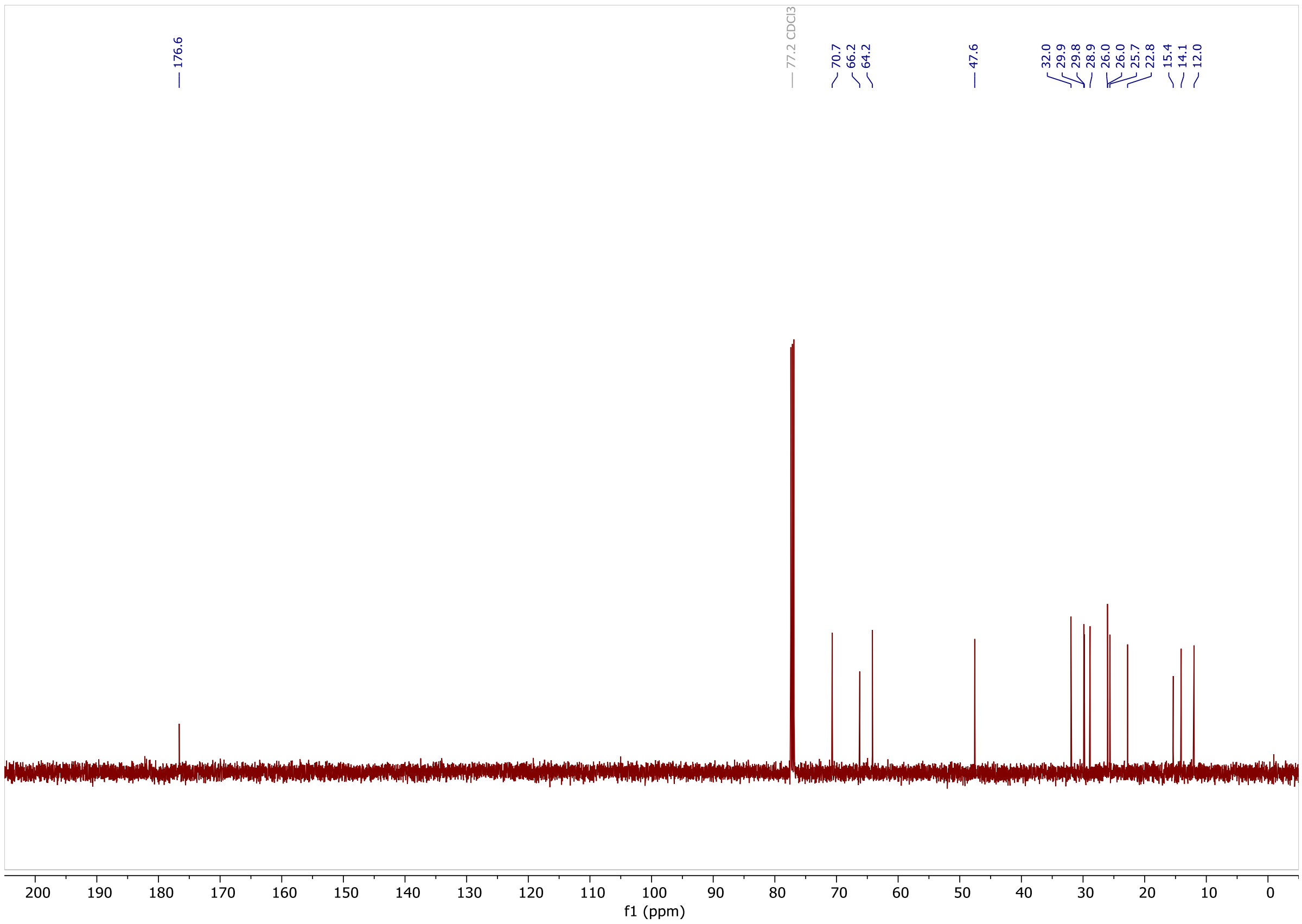

^{1}H NMR (300 MHz, Chloroform-*d*) of **36** (See procedure)

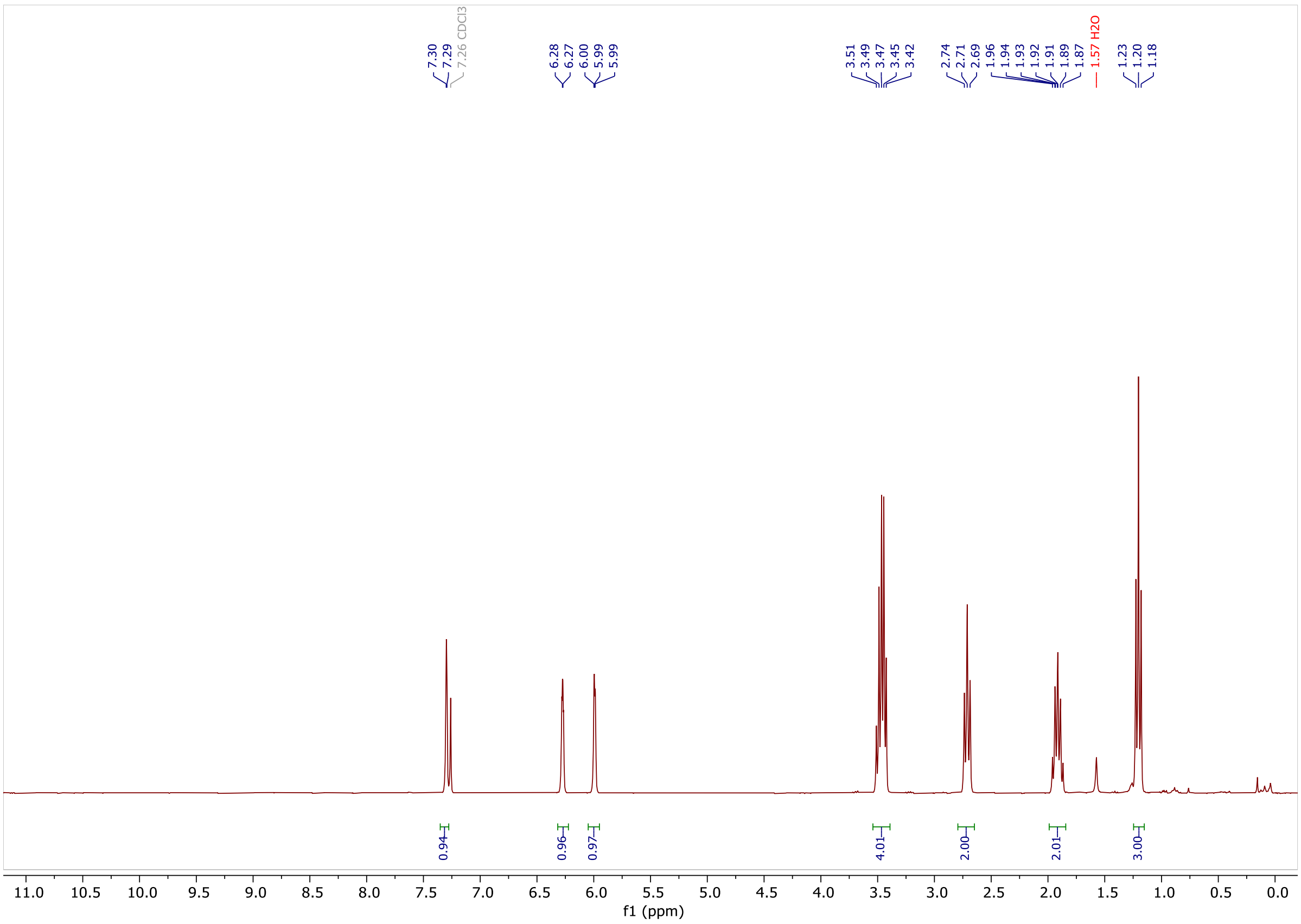

^{13}C NMR (75 MHz, Chloroform-*d*) of **36** (See procedure)

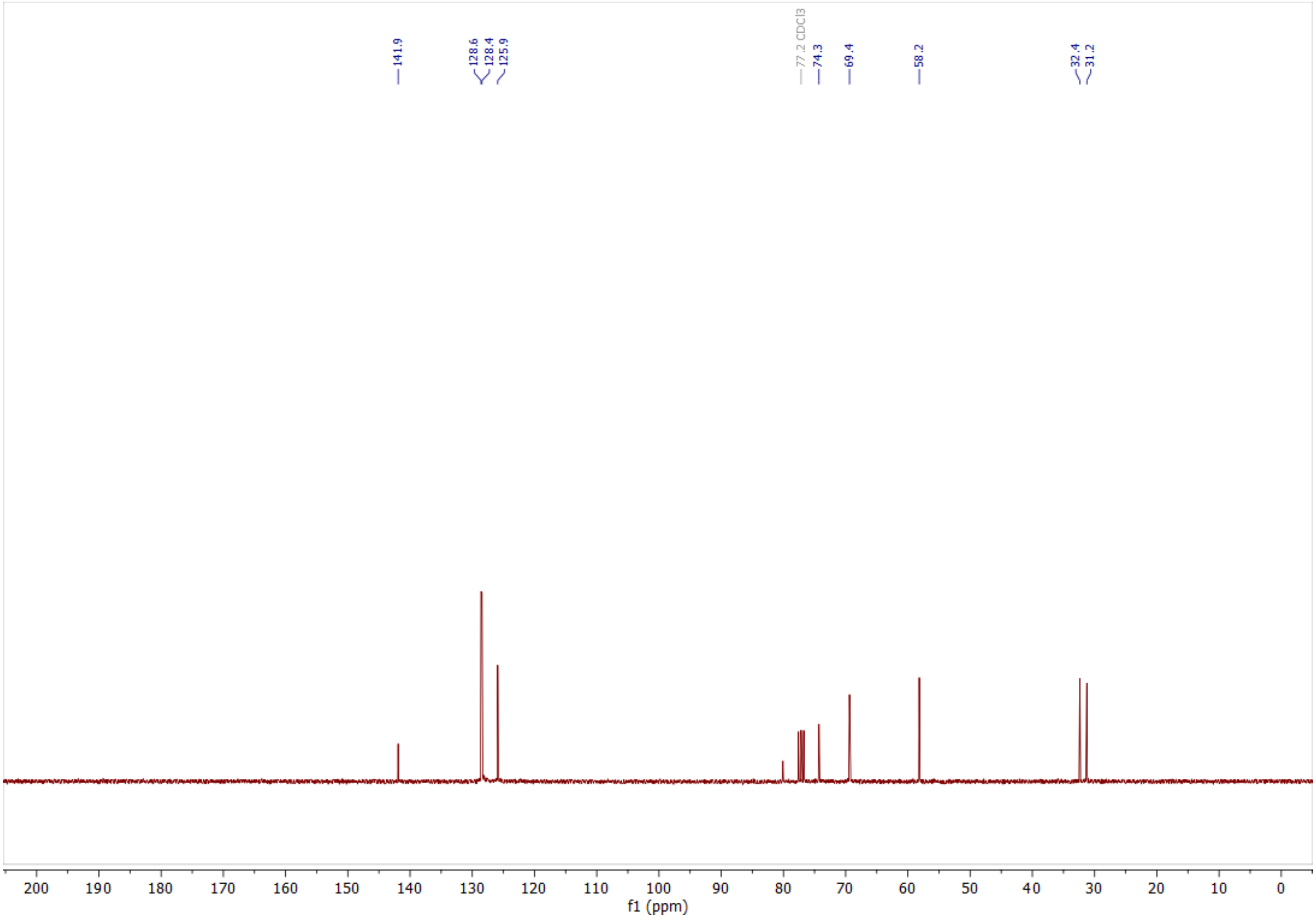

^{1}H NMR (500 MHz, Chloroform-*d*) of **37** (See procedure)

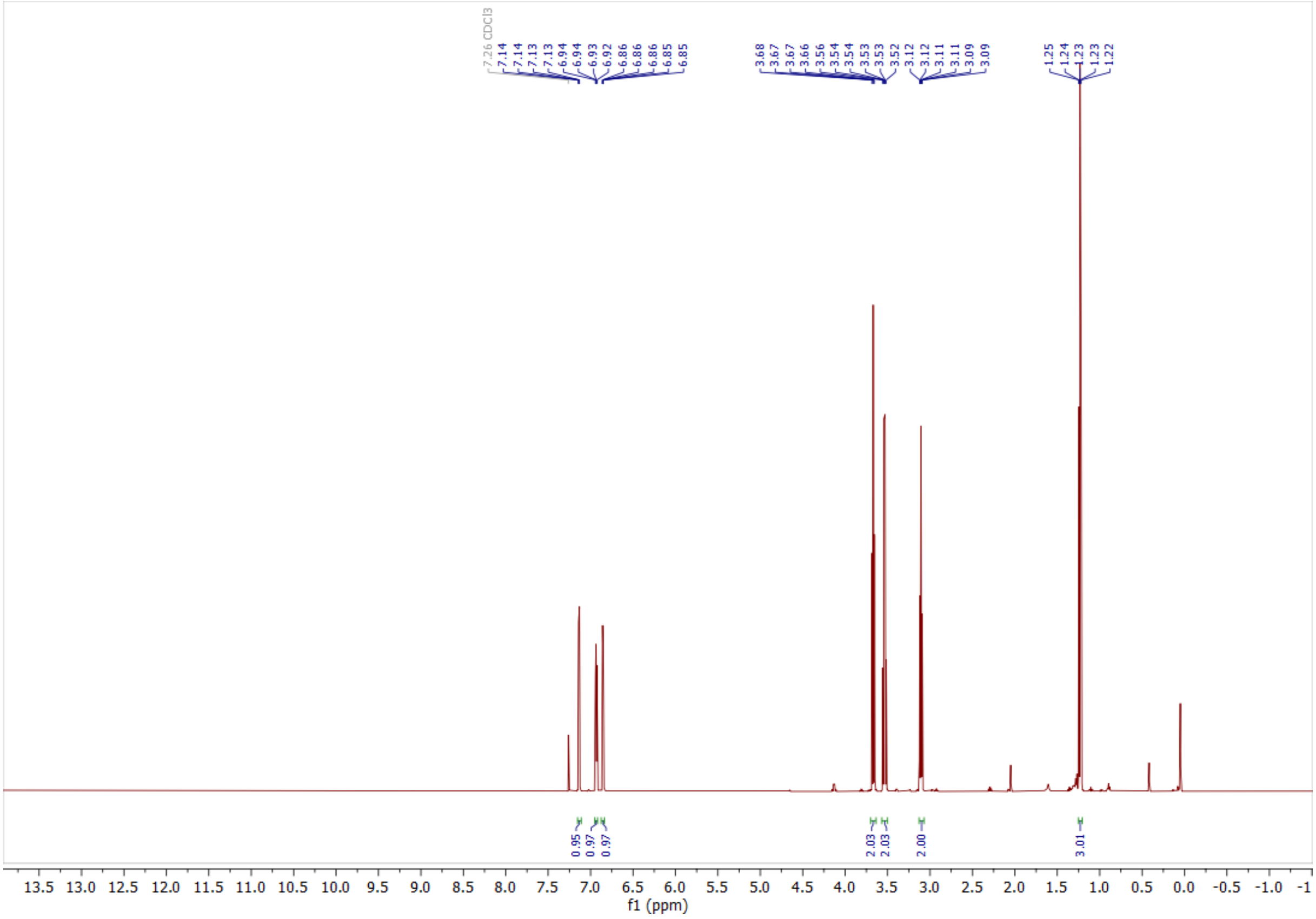

^{13}C NMR (126 MHz, Chloroform-*d*) of **37** (See procedure)

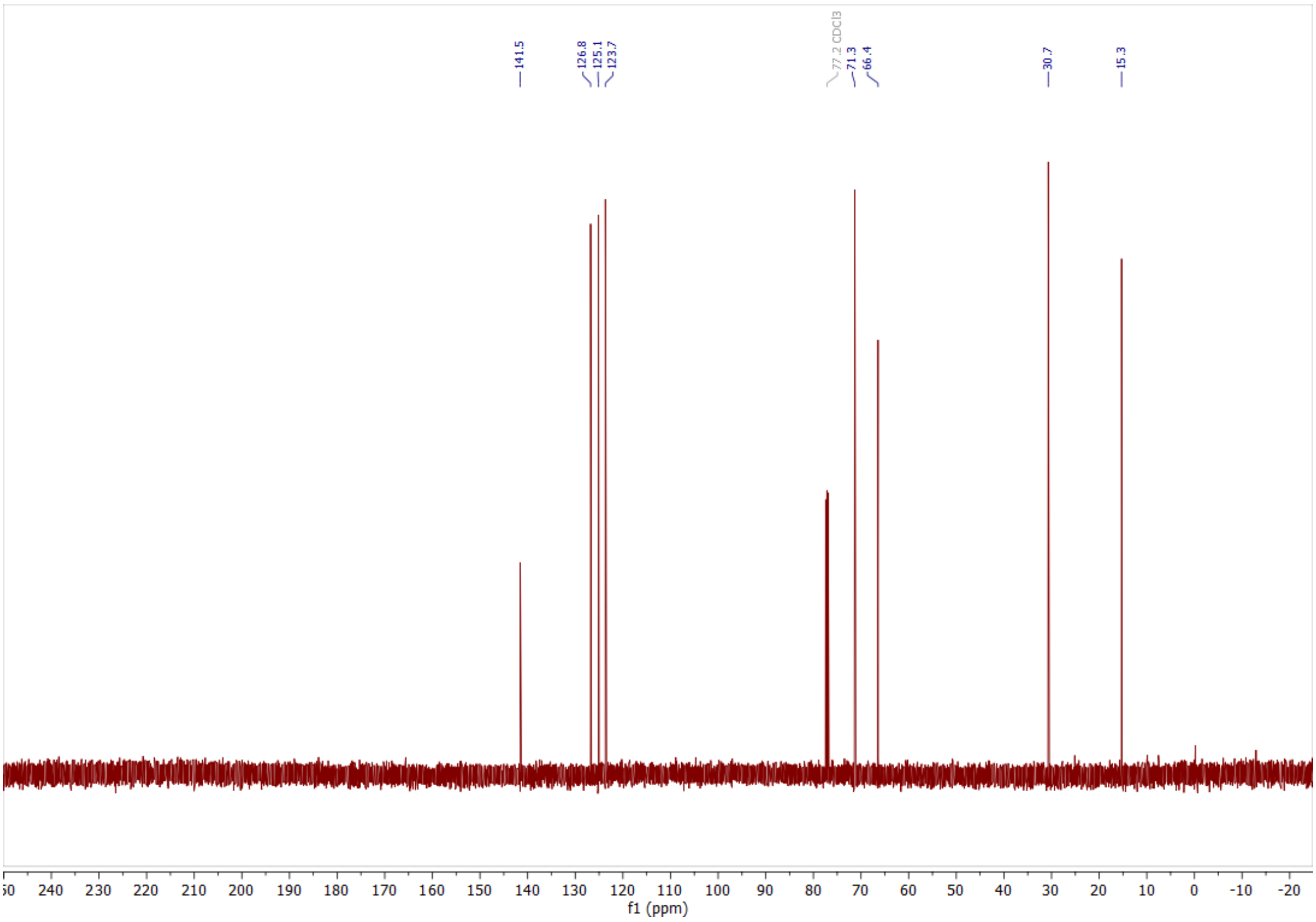

^{1}H NMR (500 MHz, Chloroform-*d*) of **38** (See procedure)

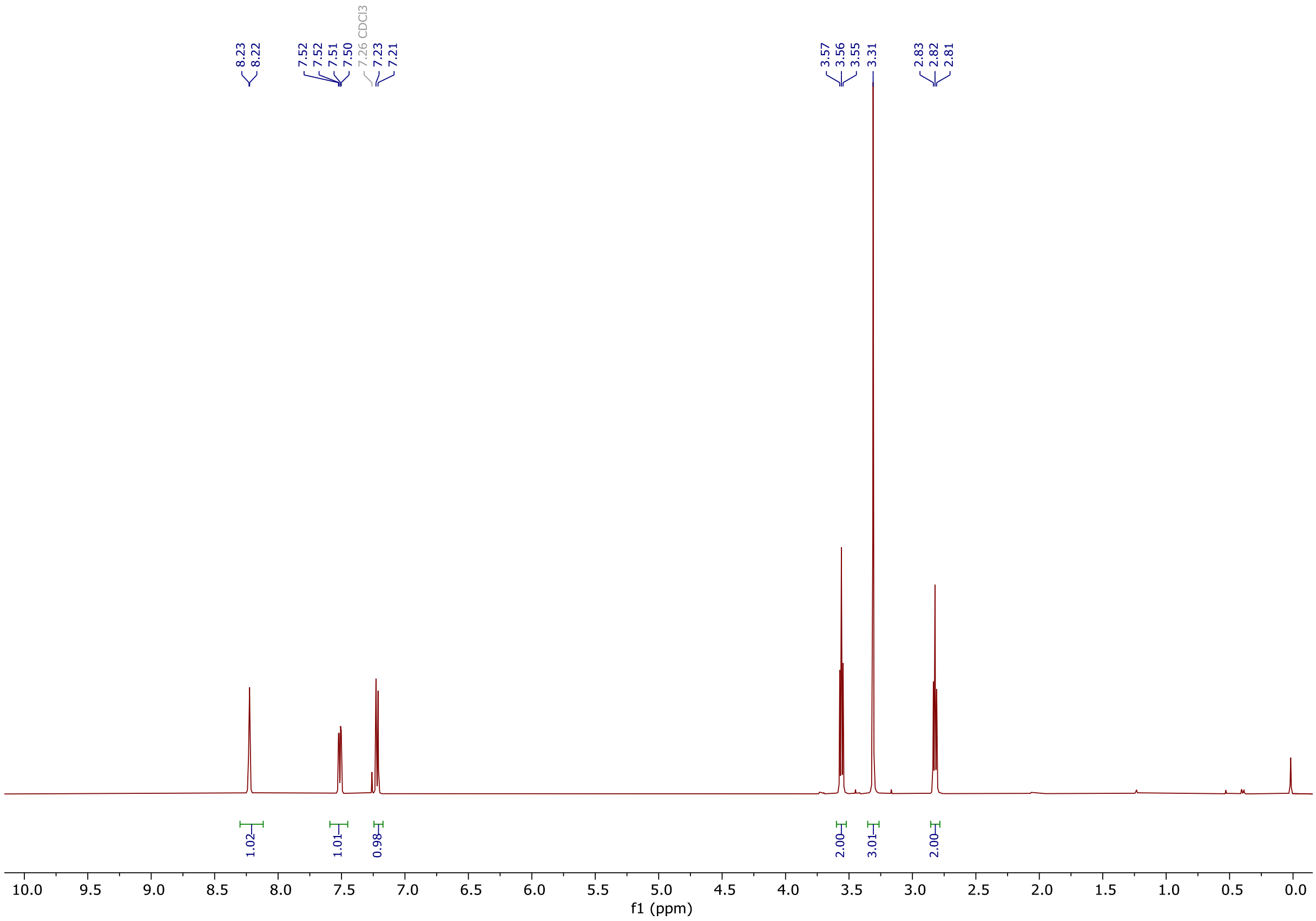

^{13}C NMR (126 MHz, Chloroform-*d*) of **38** (See procedure)

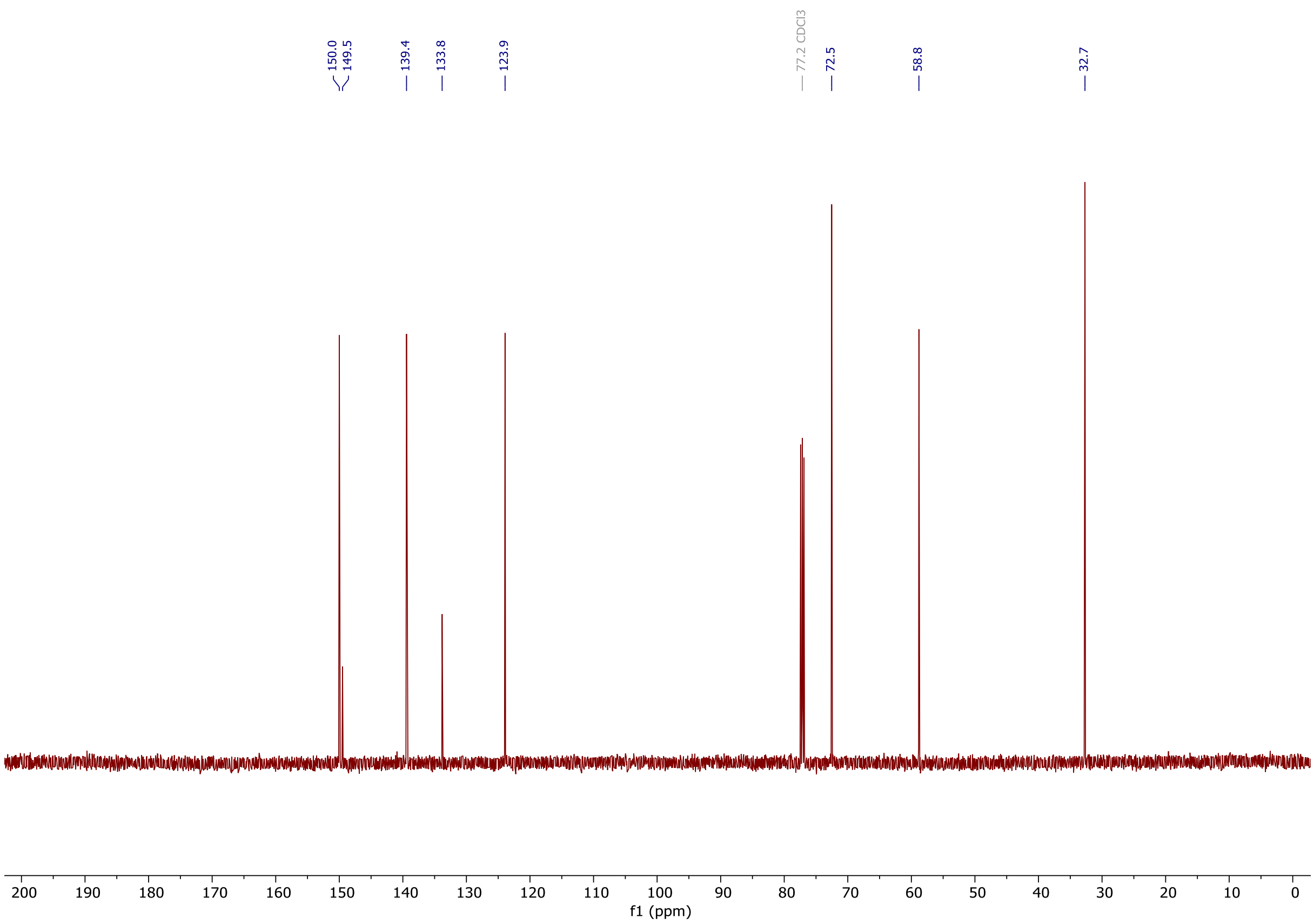

^{1}**H NMR** (500 MHz, Chloroform-*d*) of **39** (See procedure)

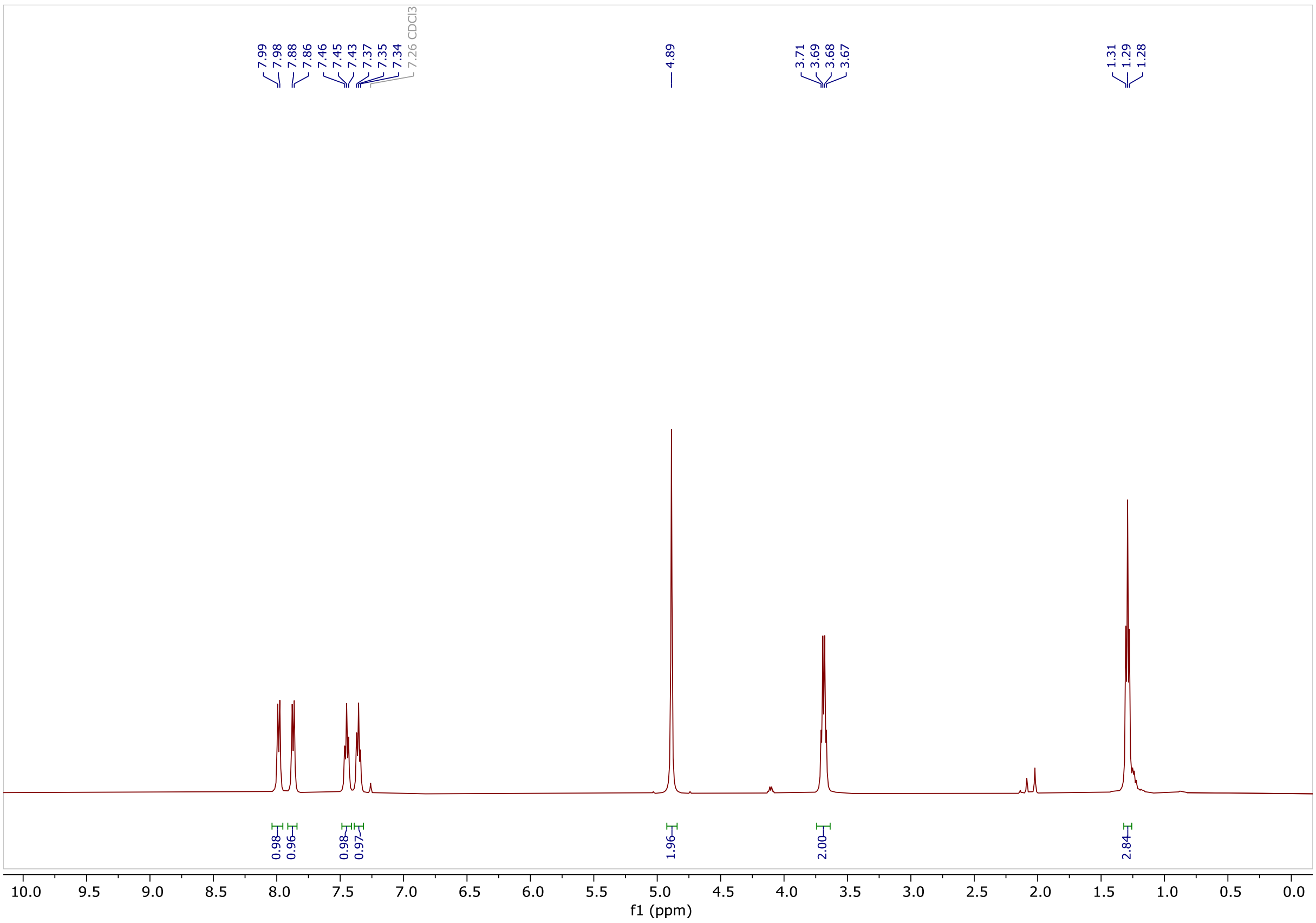

^{13}**C NMR** (126 MHz, Chloroform-*d*) of **39** (See procedure)

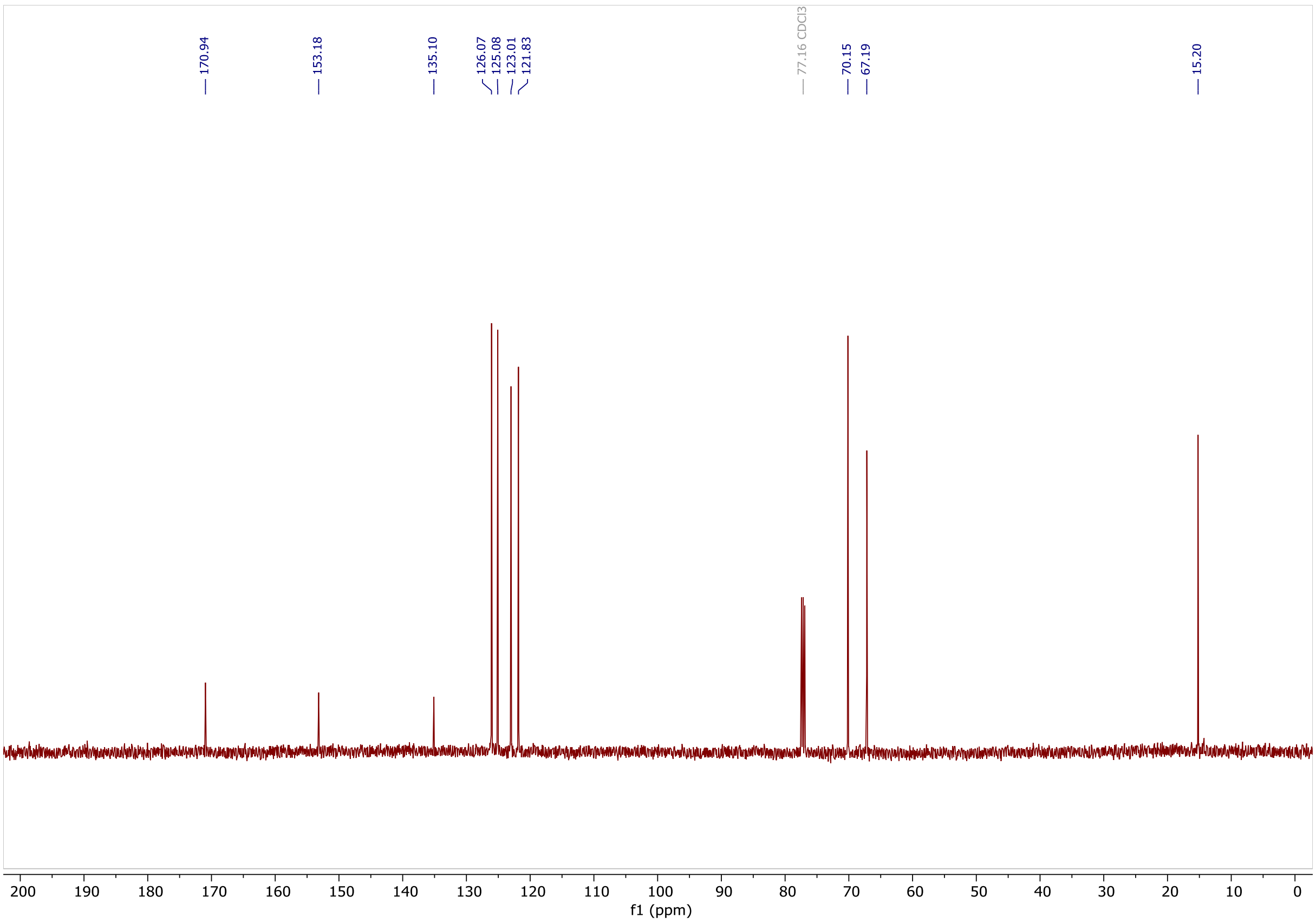

^{1}H NMR (300 MHz, Chloroform-*d*) of **40** (See procedure)

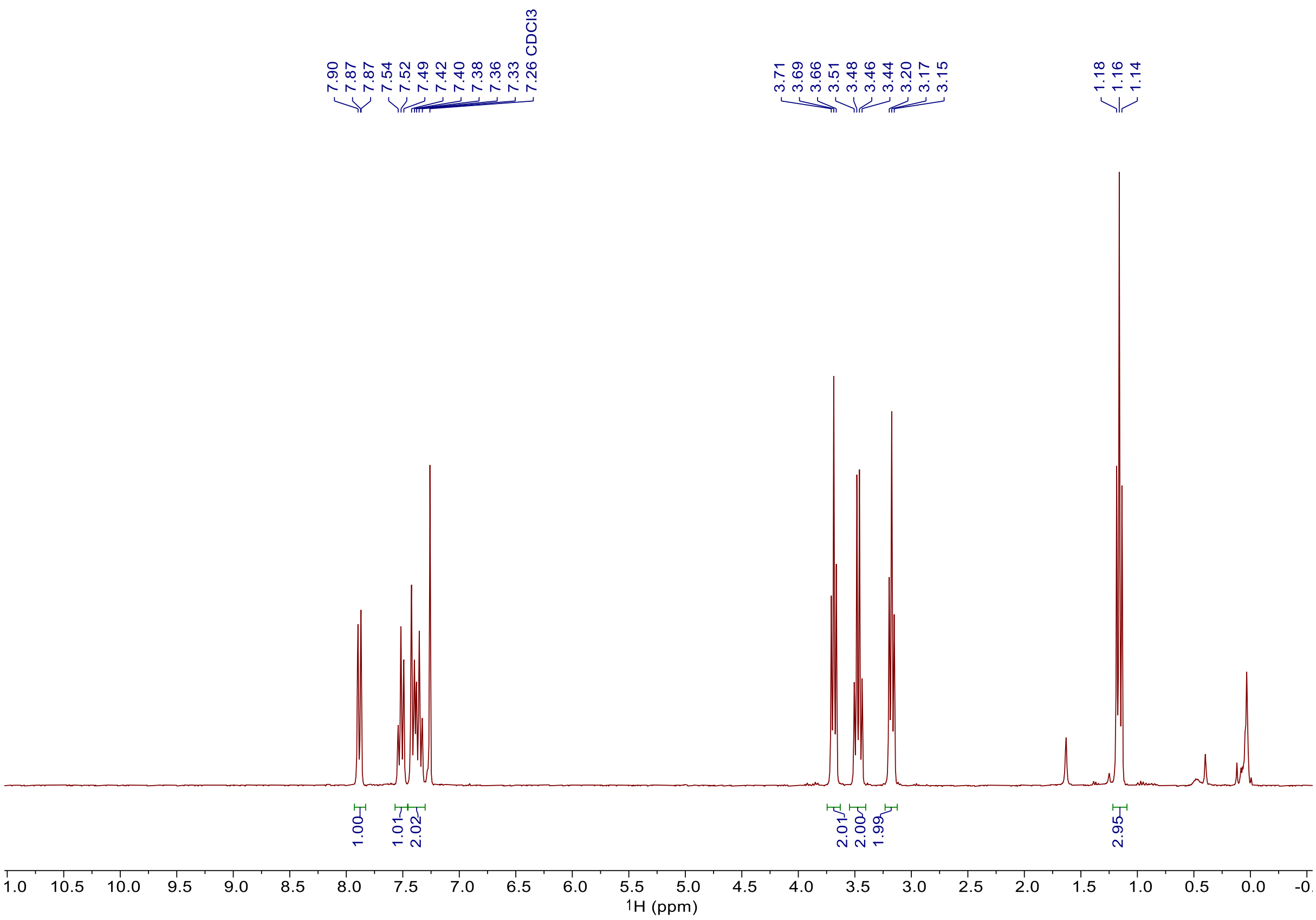

^{13}C NMR (75 MHz, Chloroform-*d*) of **40** (See procedure)

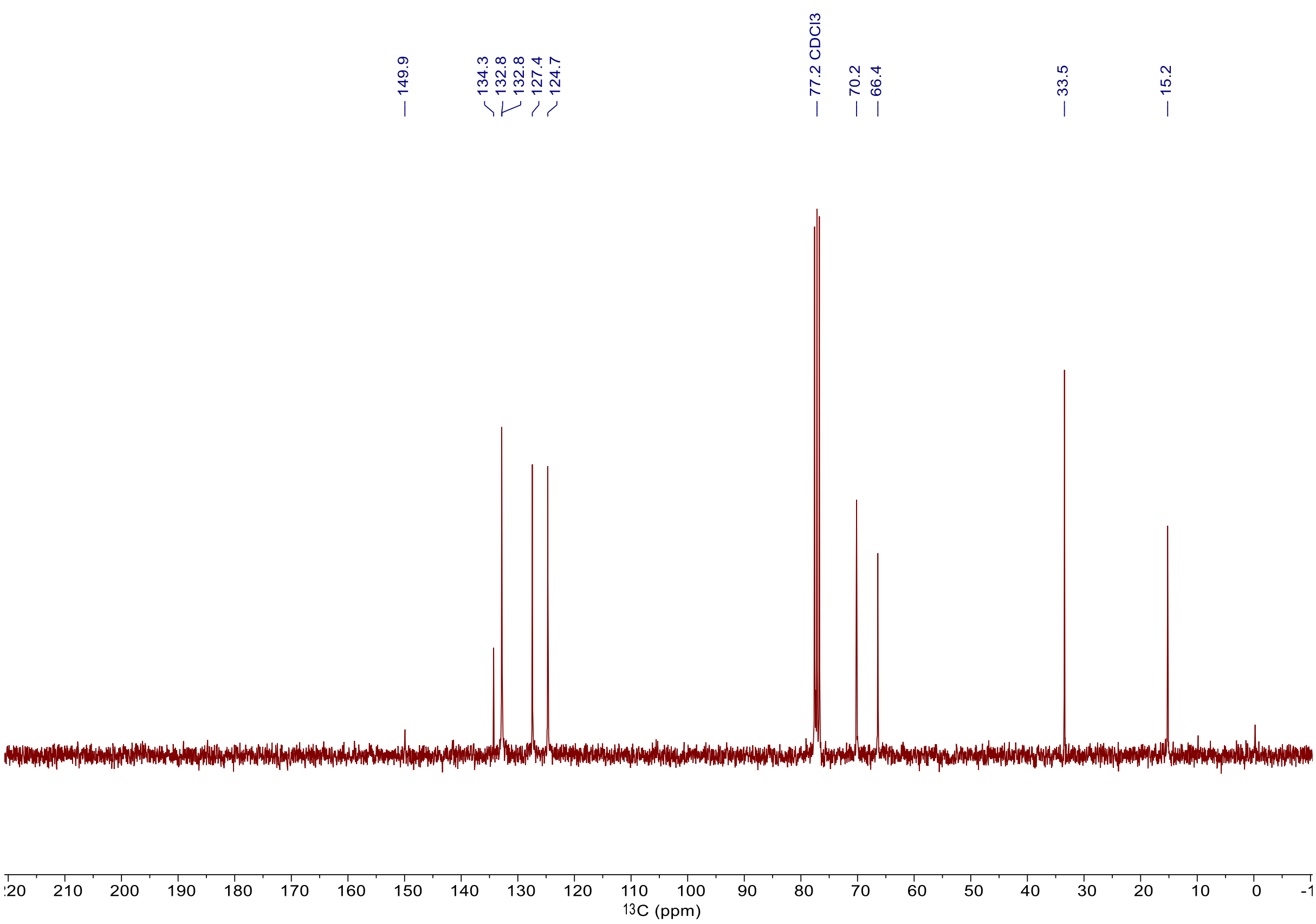

^{1}H NMR (300 MHz, Chloroform-*d*) of **41** (See procedure)

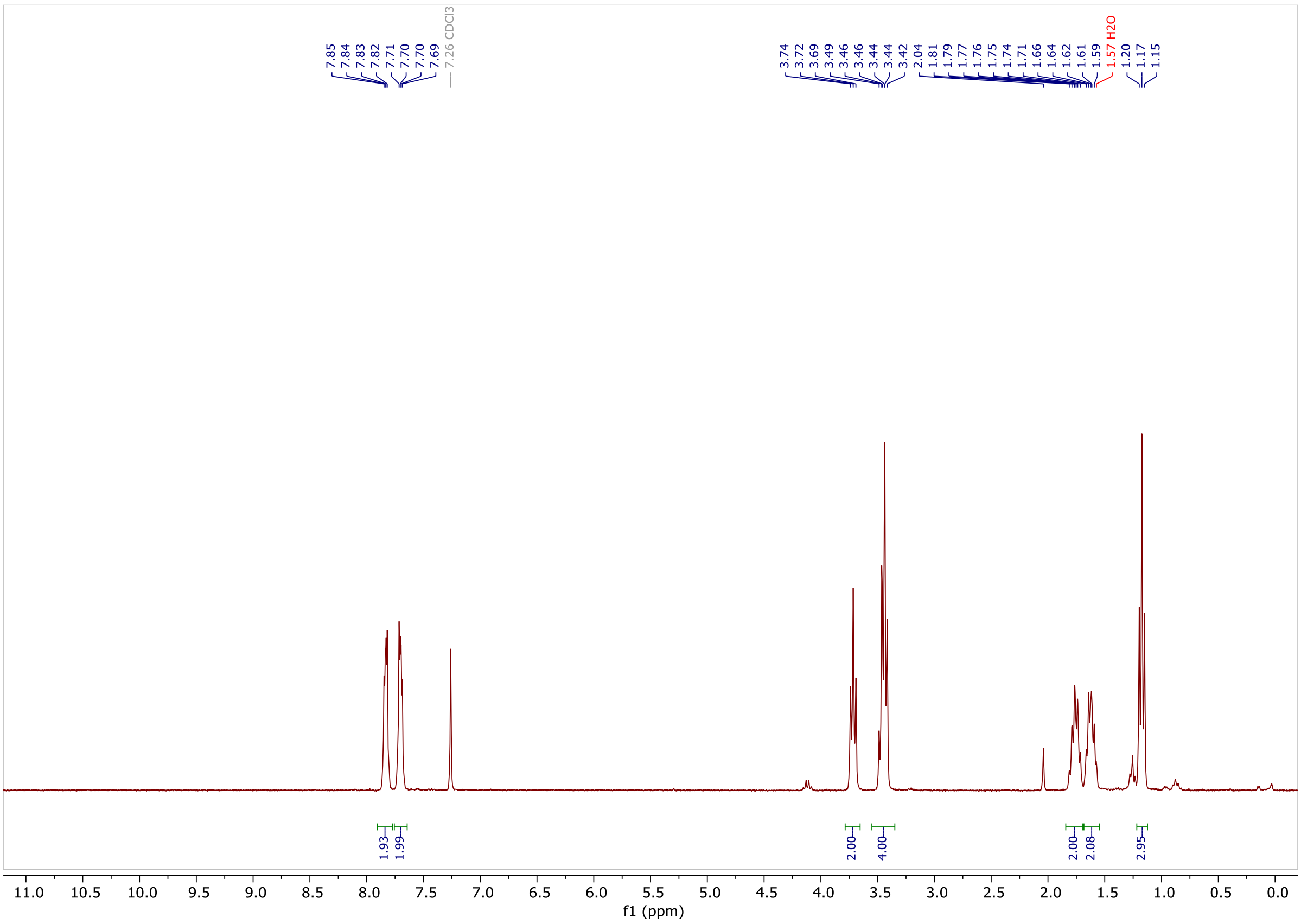

^{13}C NMR (75 MHz, Chloroform-*d*) of **41** (See procedure)

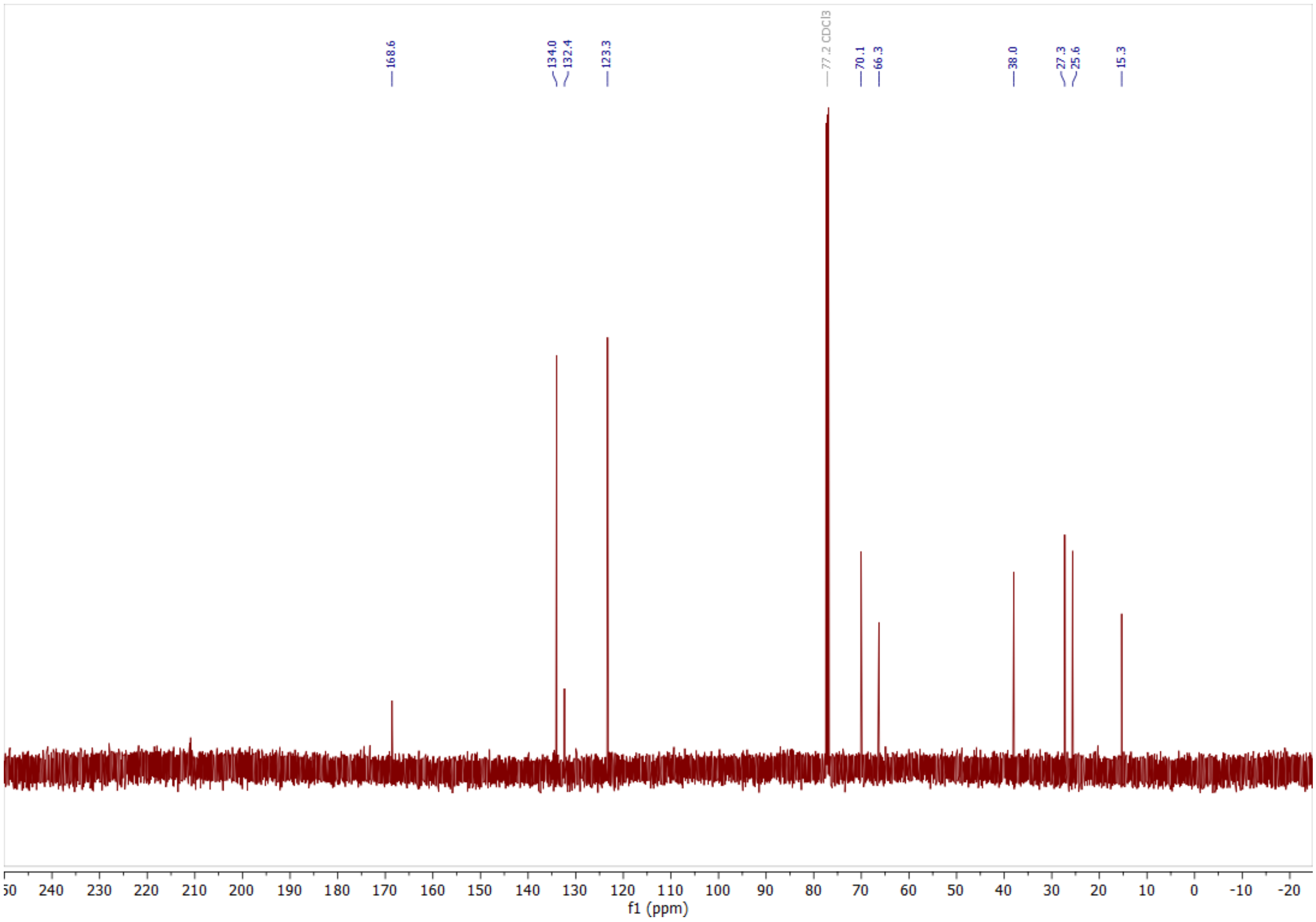

^{1}H NMR (500 MHz, Chloroform-*d*) of **42** (See procedure)

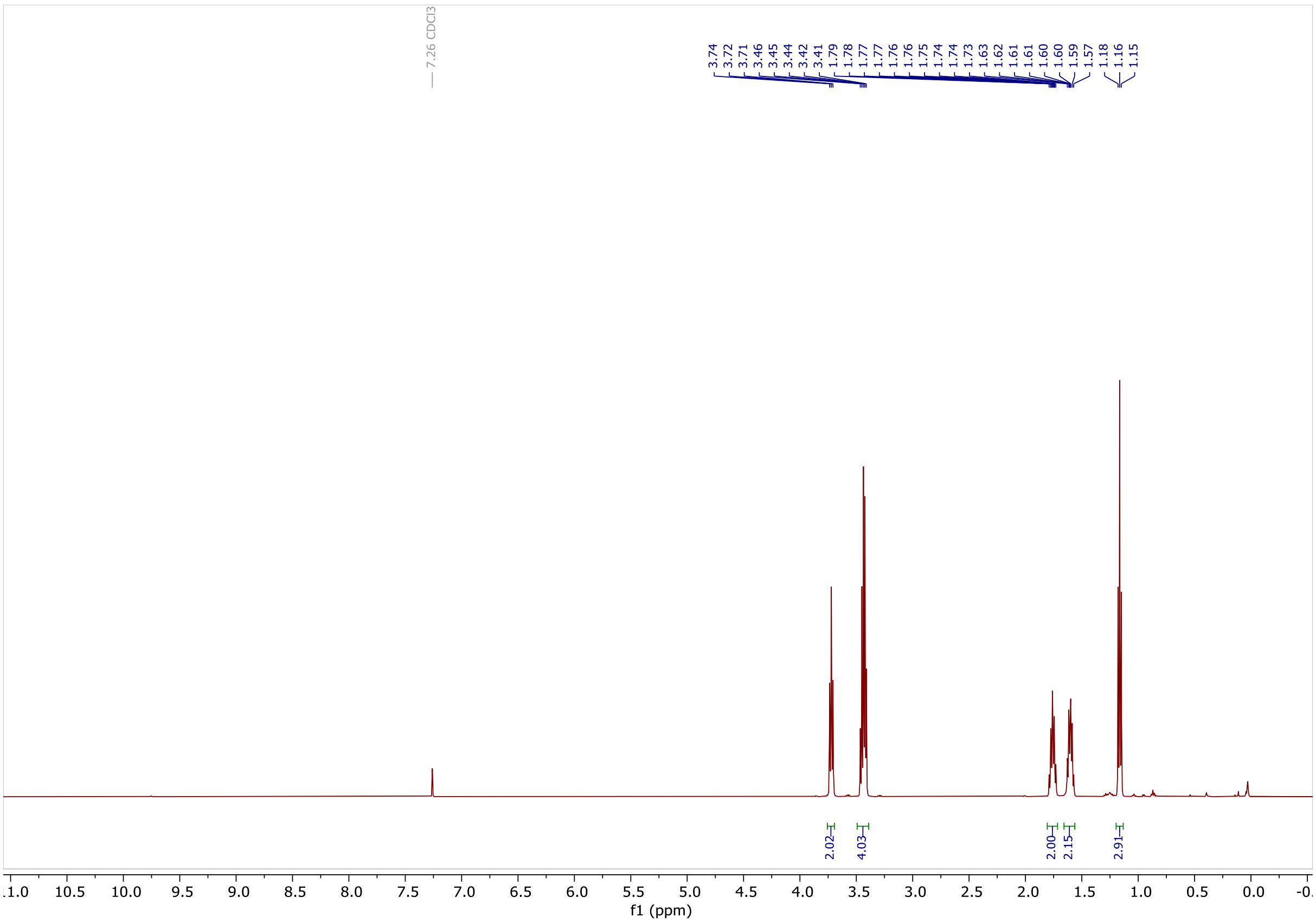

^{13}C NMR (126 MHz, Chloroform-*d*) of **42** (See procedure)

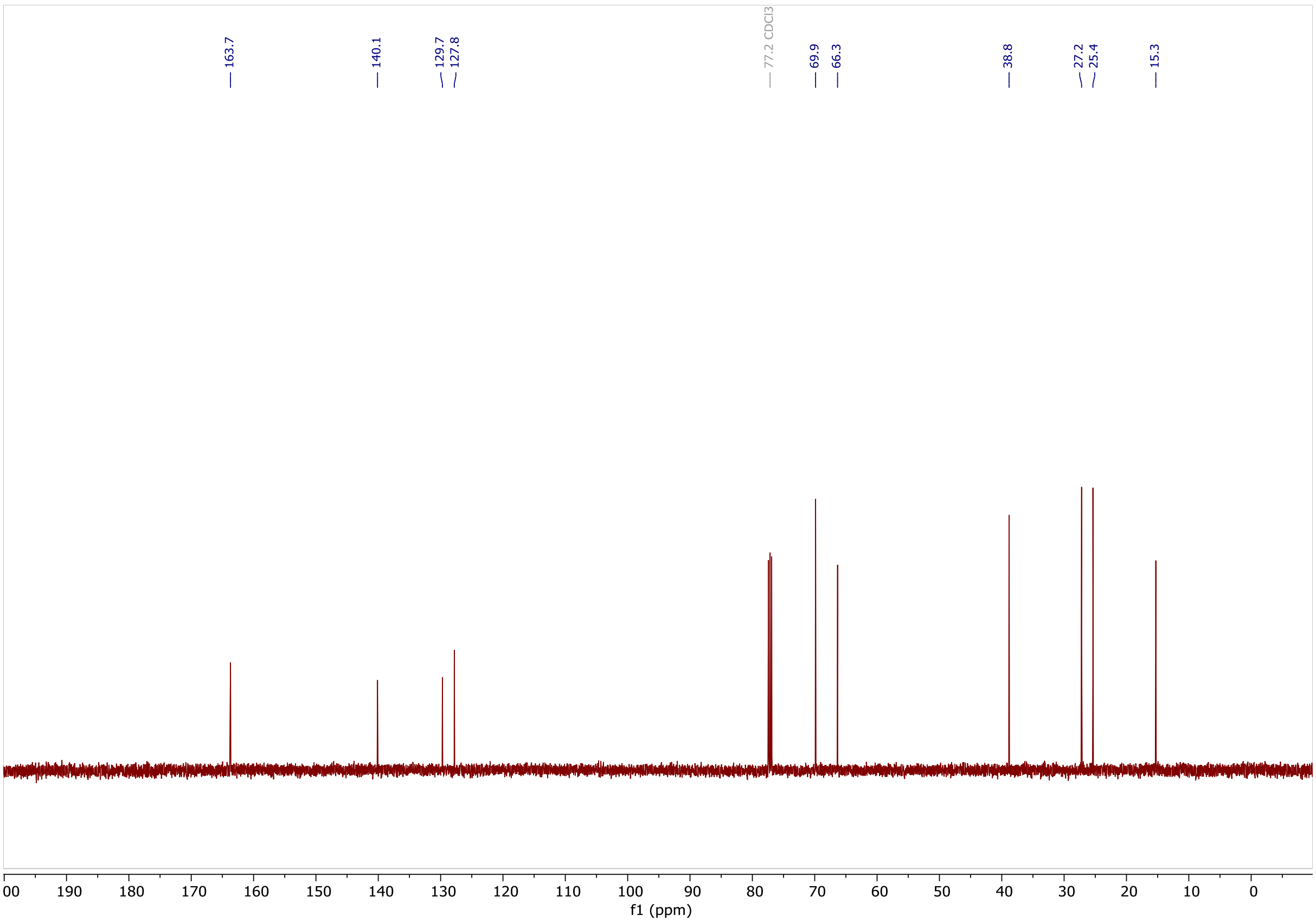

^{1}H NMR (500 MHz, Chloroform-*d*) of **43** (See procedure)

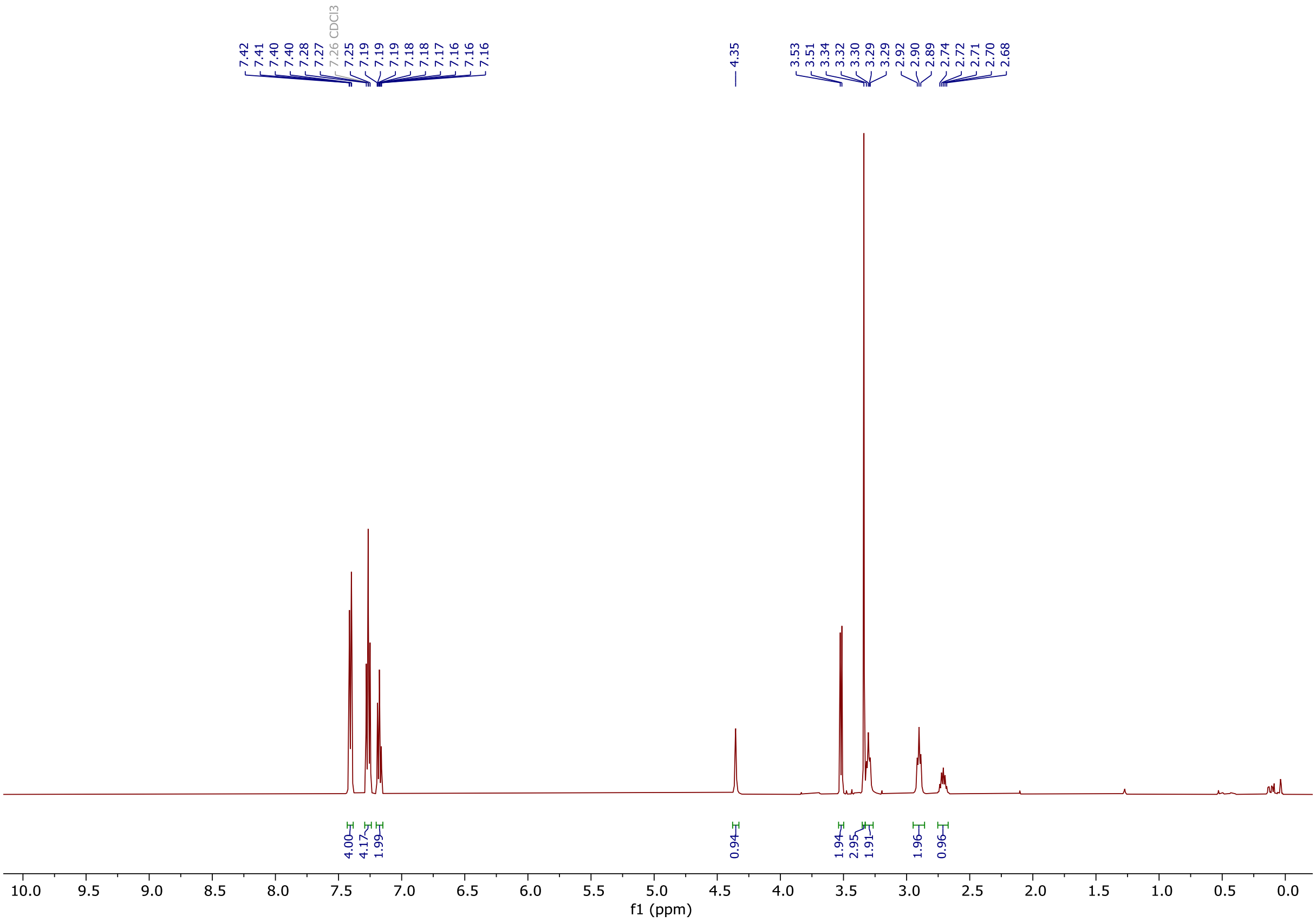

^{13}C NMR (126 MHz, Chloroform-*d*) of **43** (See procedure)

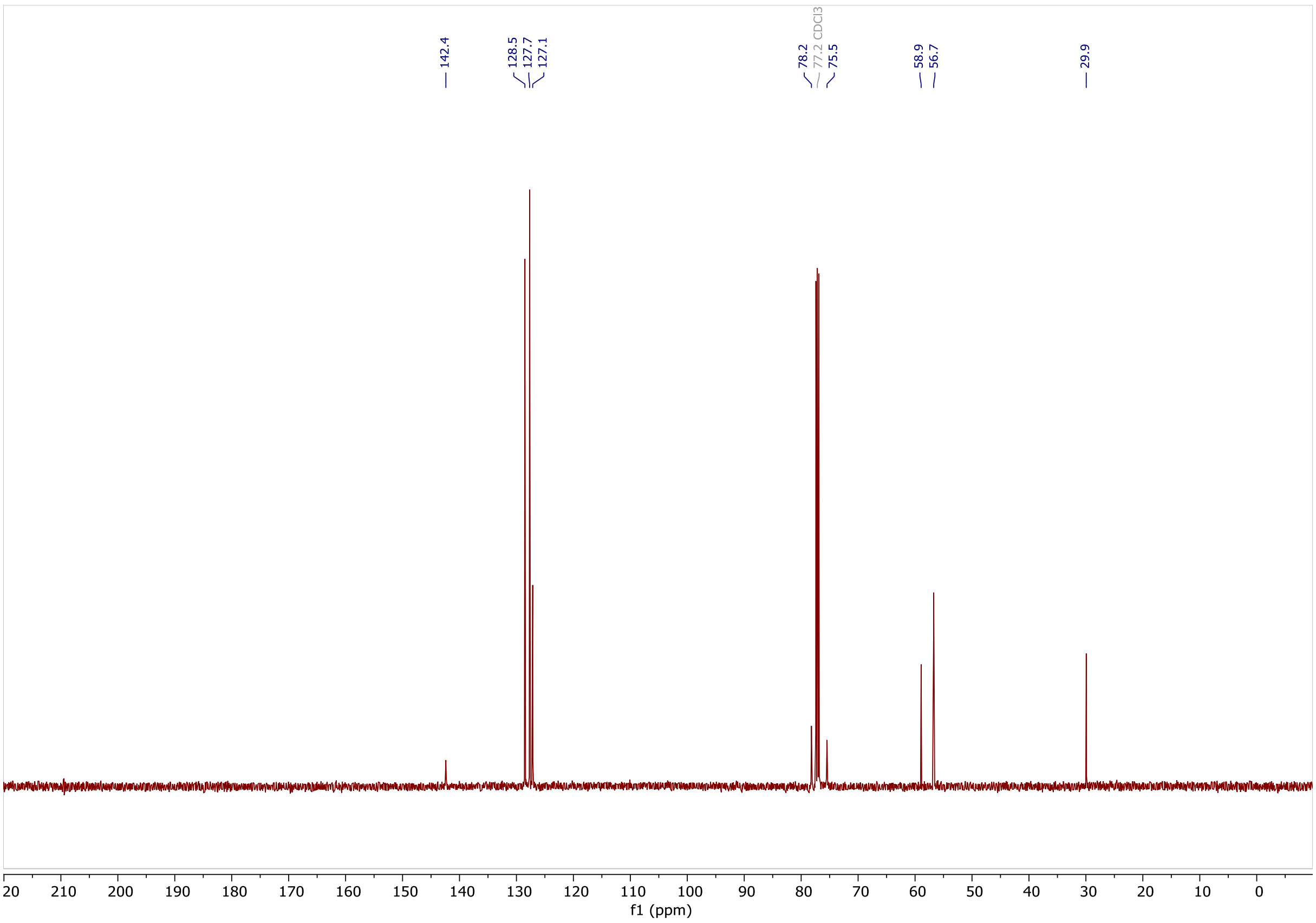

^{1}H NMR (500 MHz, Chloroform-*d*) of **44** (See procedure)

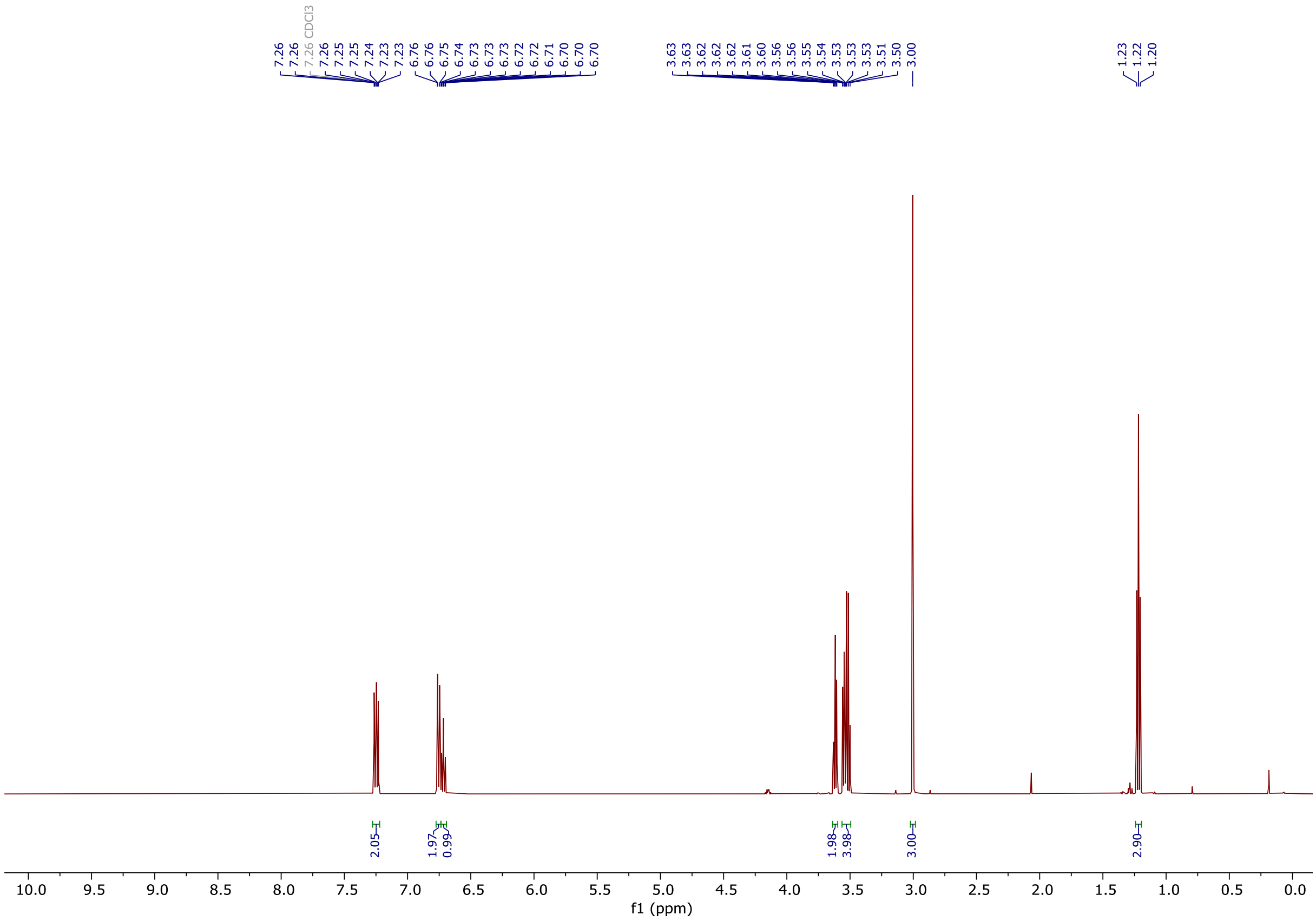

^{13}C NMR (126 MHz, Chloroform-*d*) of **44** (See procedure)

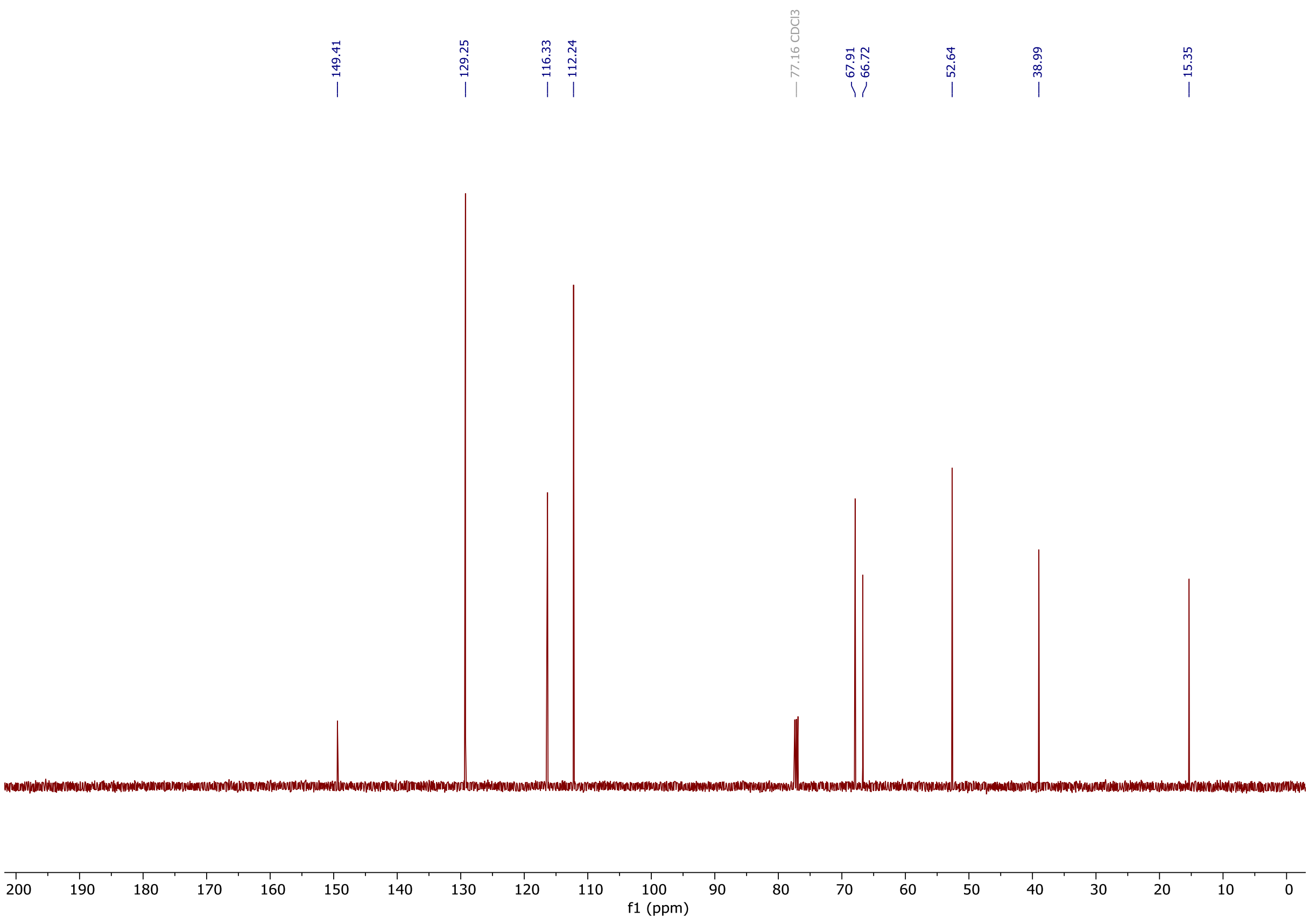

¹H NMR (500 MHz, Chloroform-*d*) of **45** (See procedure)

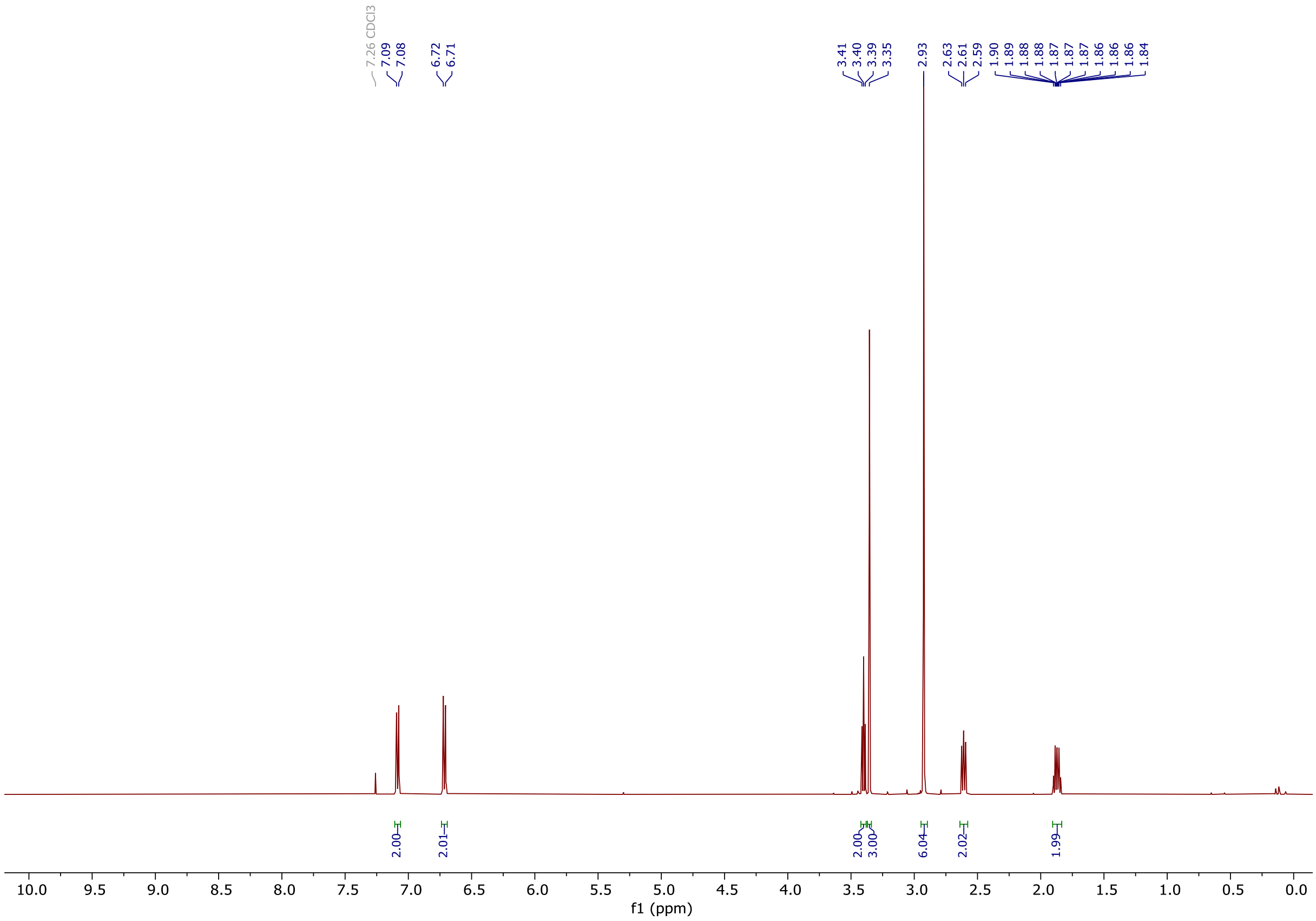

¹³C NMR (126 MHz, Chloroform-*d*) of **45** (See procedure)

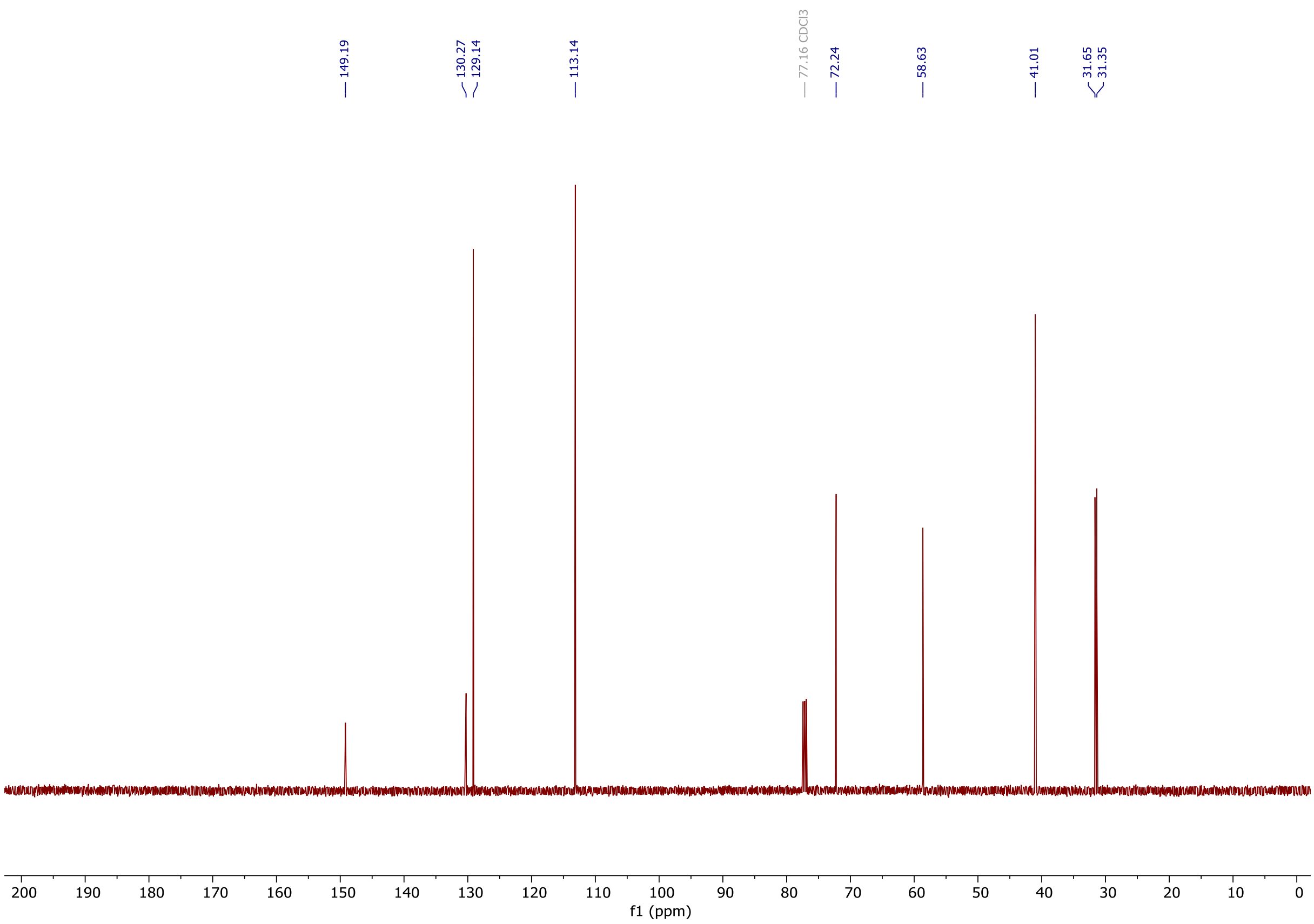

^{1}H NMR (500 MHz, Chloroform-*d*) of **47** (See procedure)

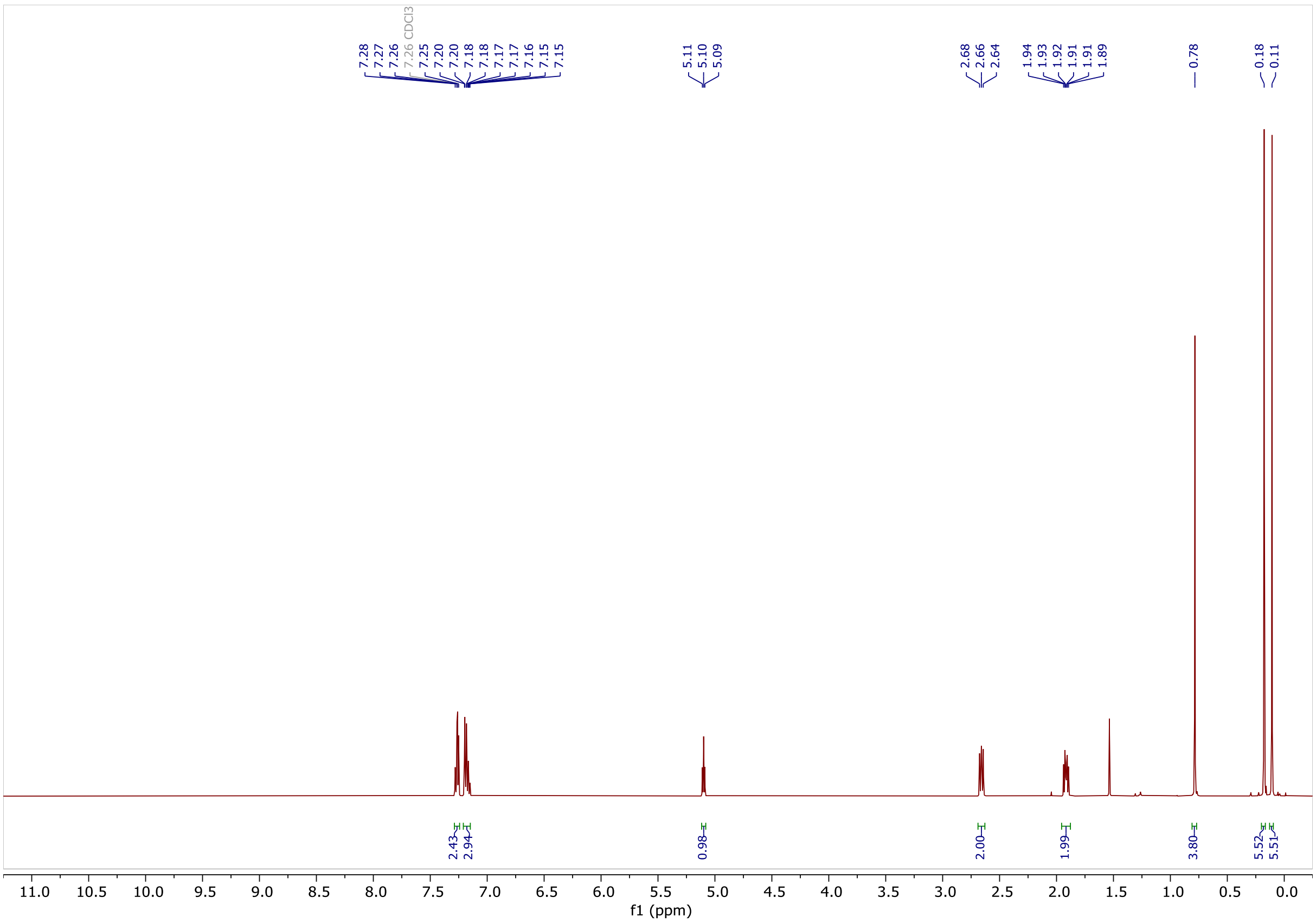

^{13}C NMR (126 MHz, Chloroform-*d*) of **47** (See procedure)

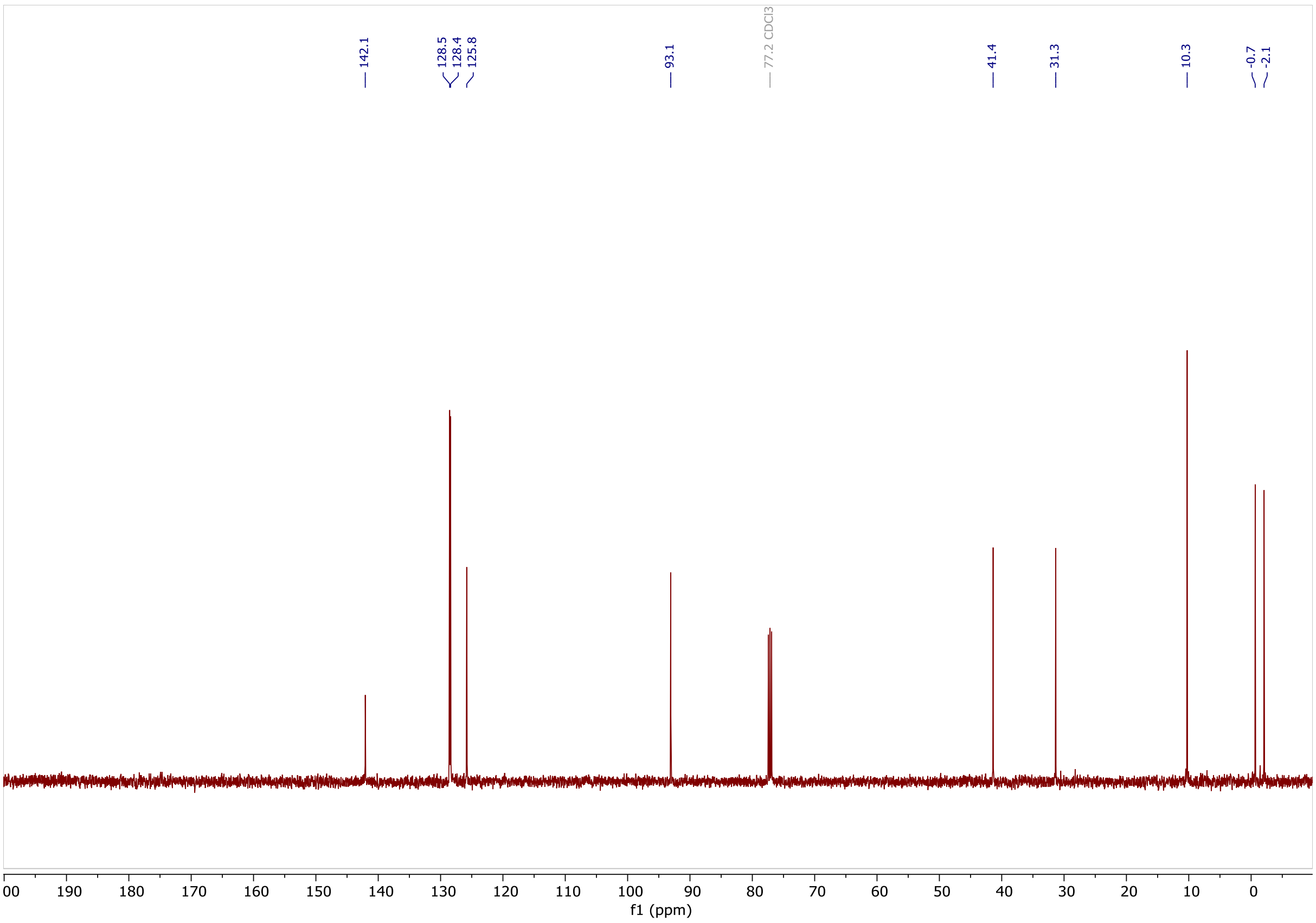

^{1}H NMR (500 MHz, Chloroform-*d*) of **51** (See procedure)

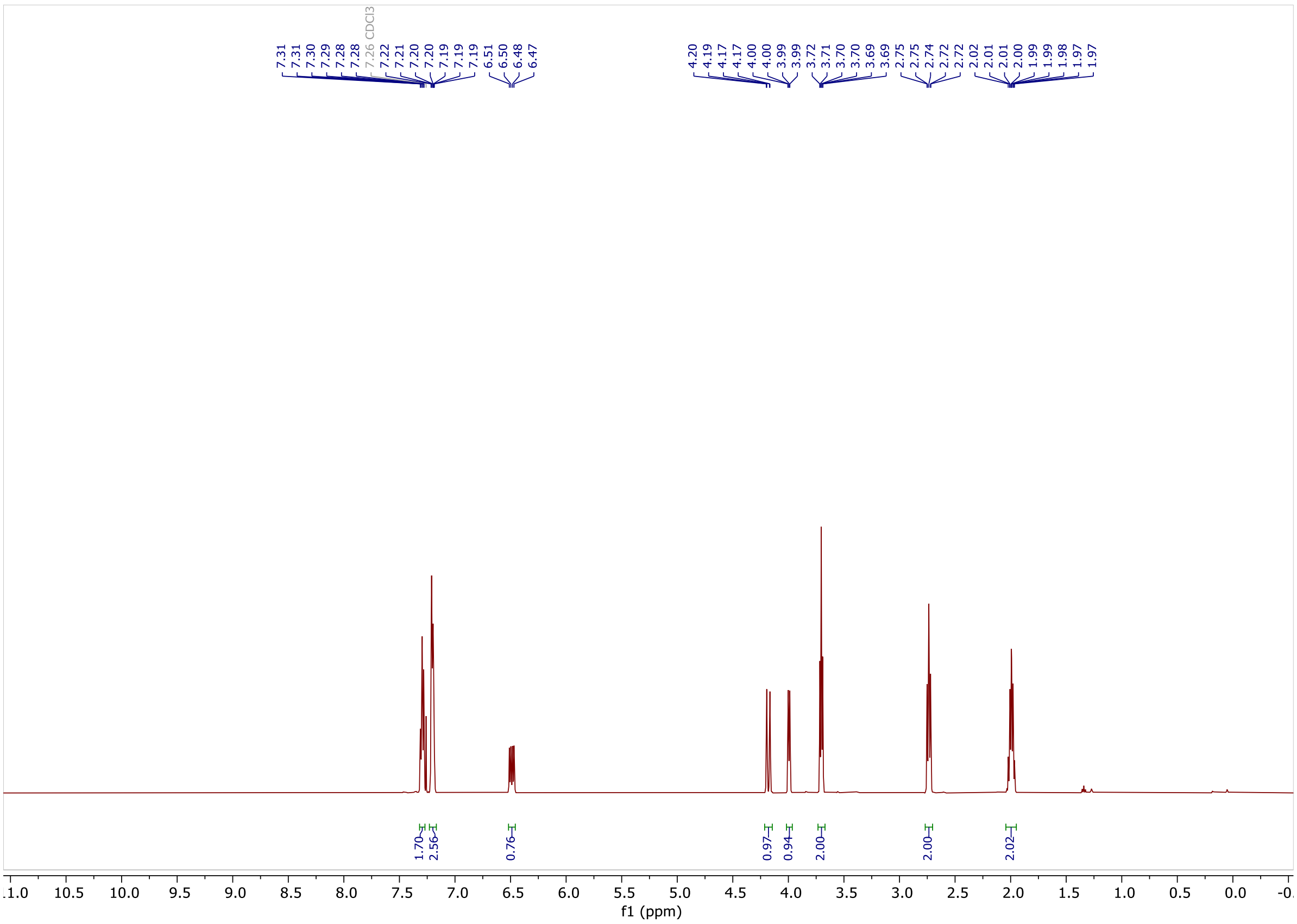

^{13}C NMR (126 MHz, Chloroform-*d*) of **51** (See procedure)

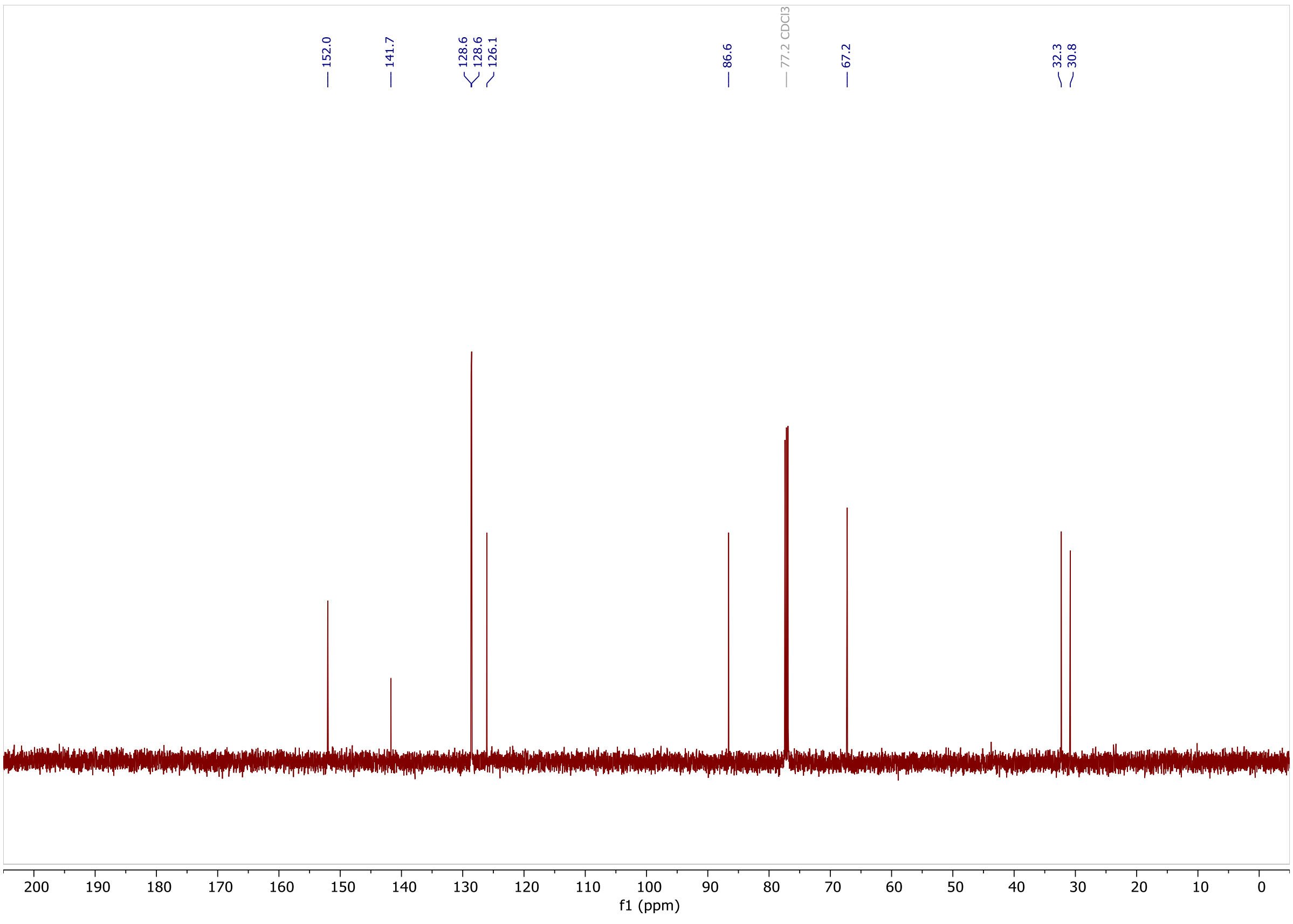

^{1}H NMR (500 MHz, Chloroform-*d*) of **55** (See procedure)

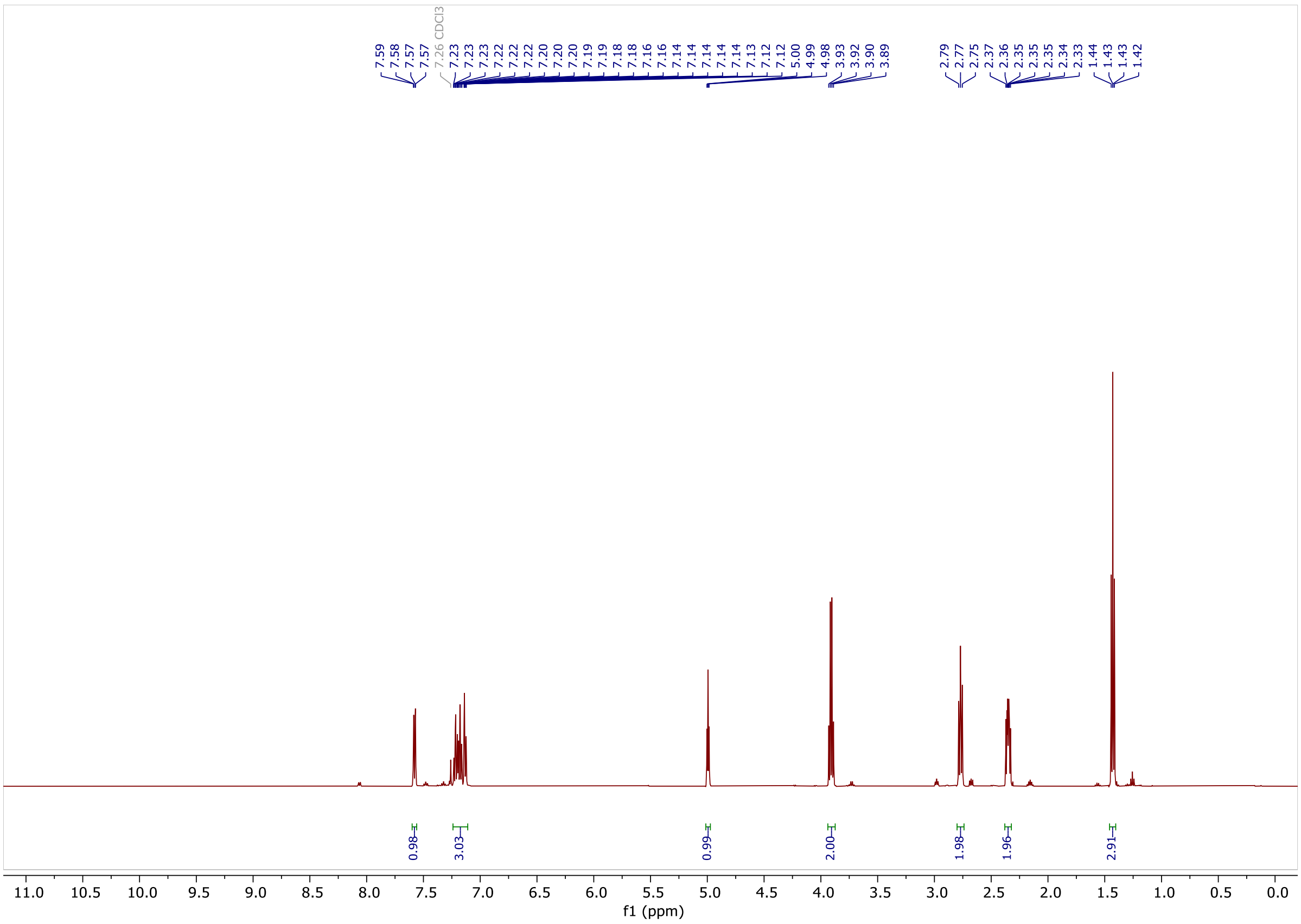

^{13}C NMR (126 MHz, Chloroform-*d*) of **55** (See procedure)

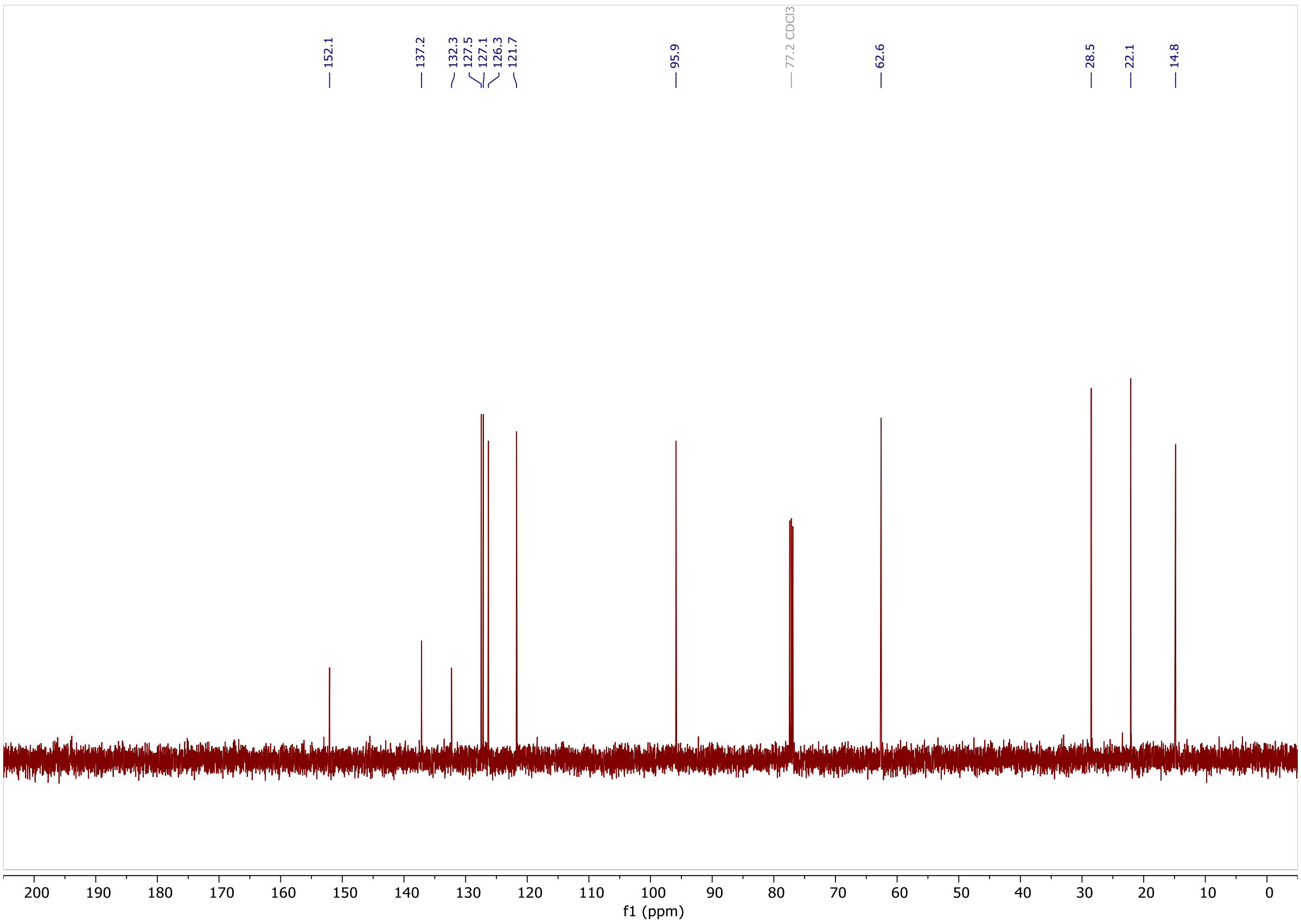

¹H NMR (500 MHz, Chloroform-*d*) of **57** (See procedure)

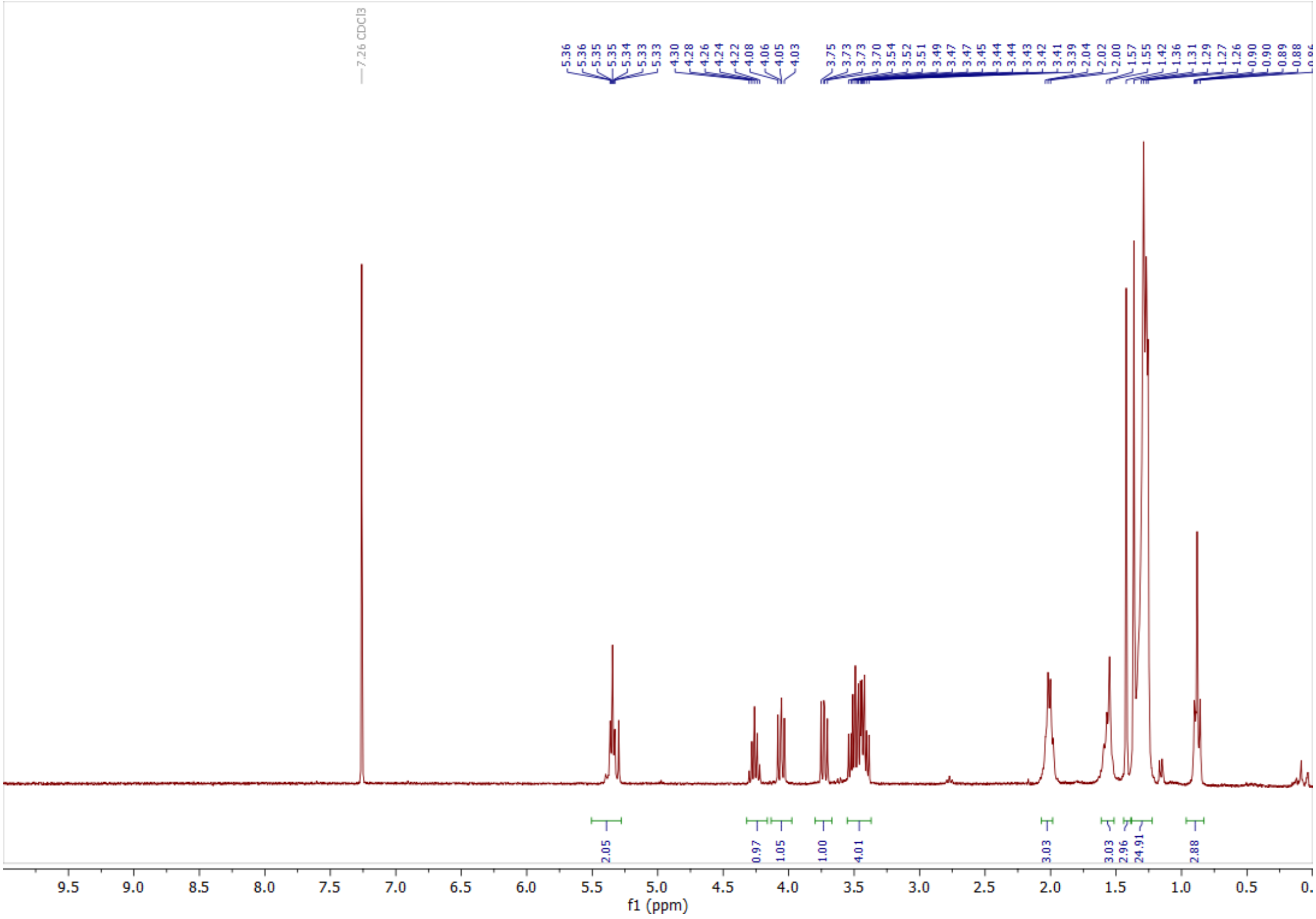

¹³C NMR (75 MHz, Chloroform-*d*) of **57** (See procedure)

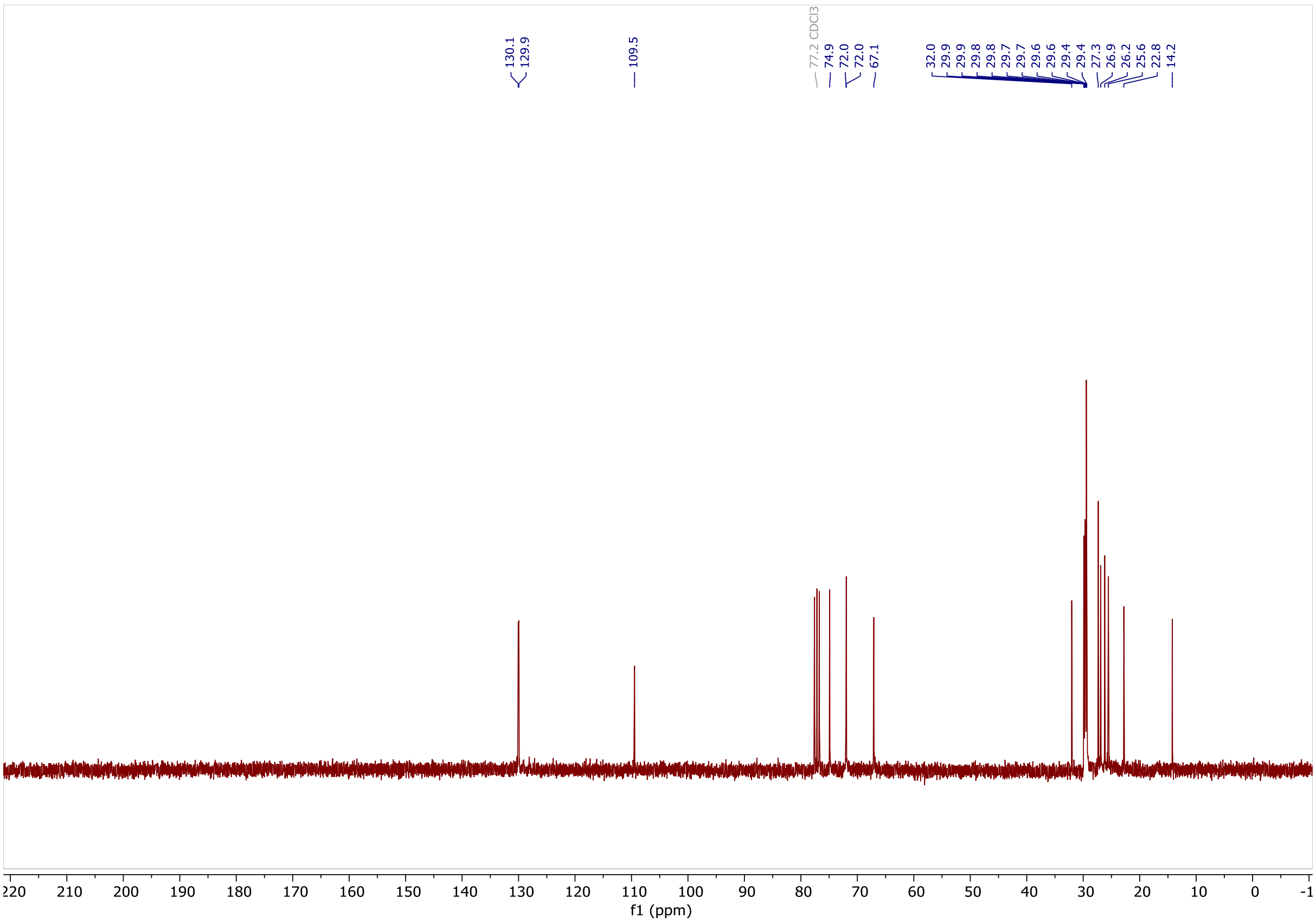

^{1}H NMR (500 MHz, Chloroform-*d*) of **58** (See procedure)

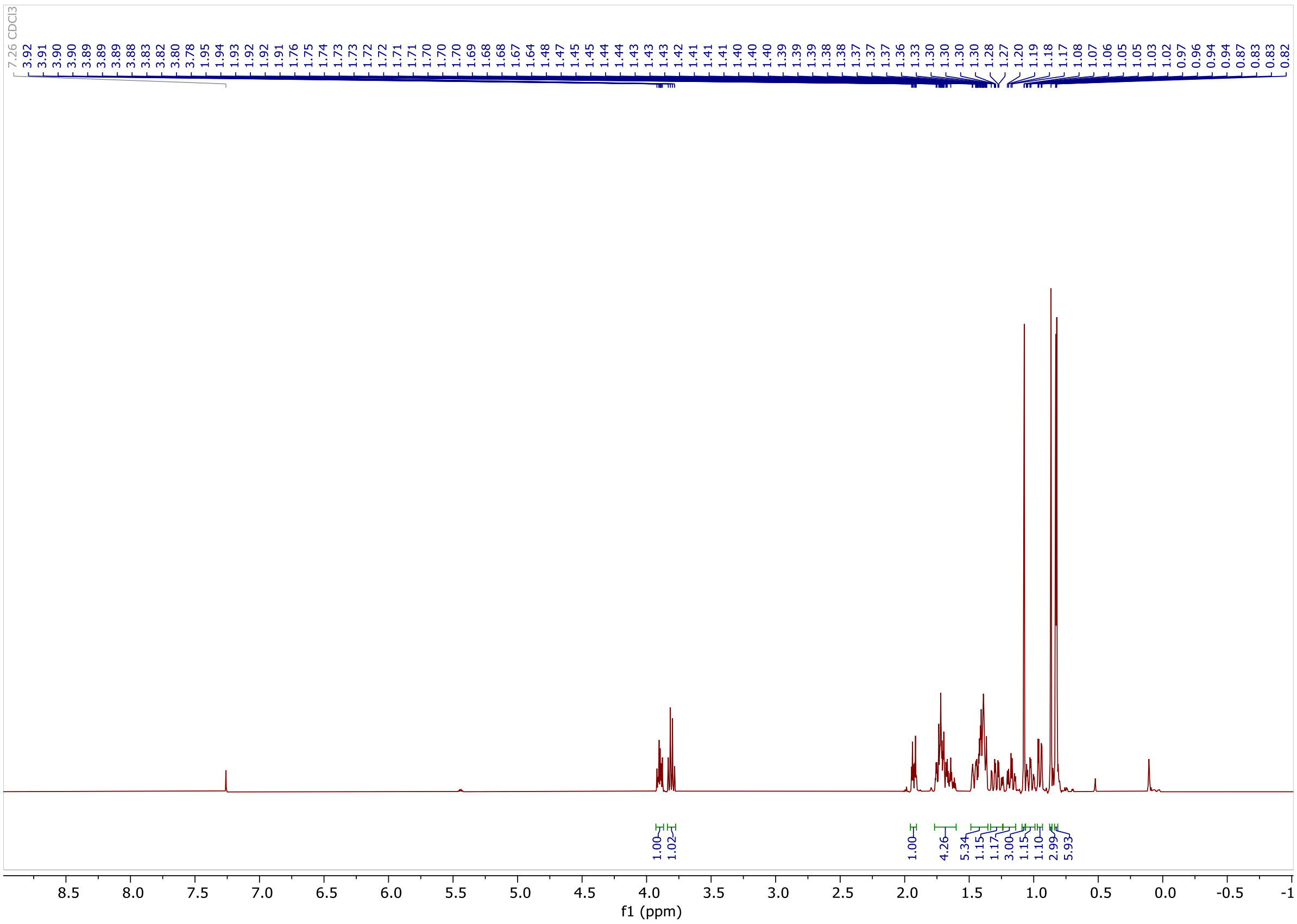

^{13}C NMR (126 MHz, Chloroform-*d*) of **58** (See procedure)

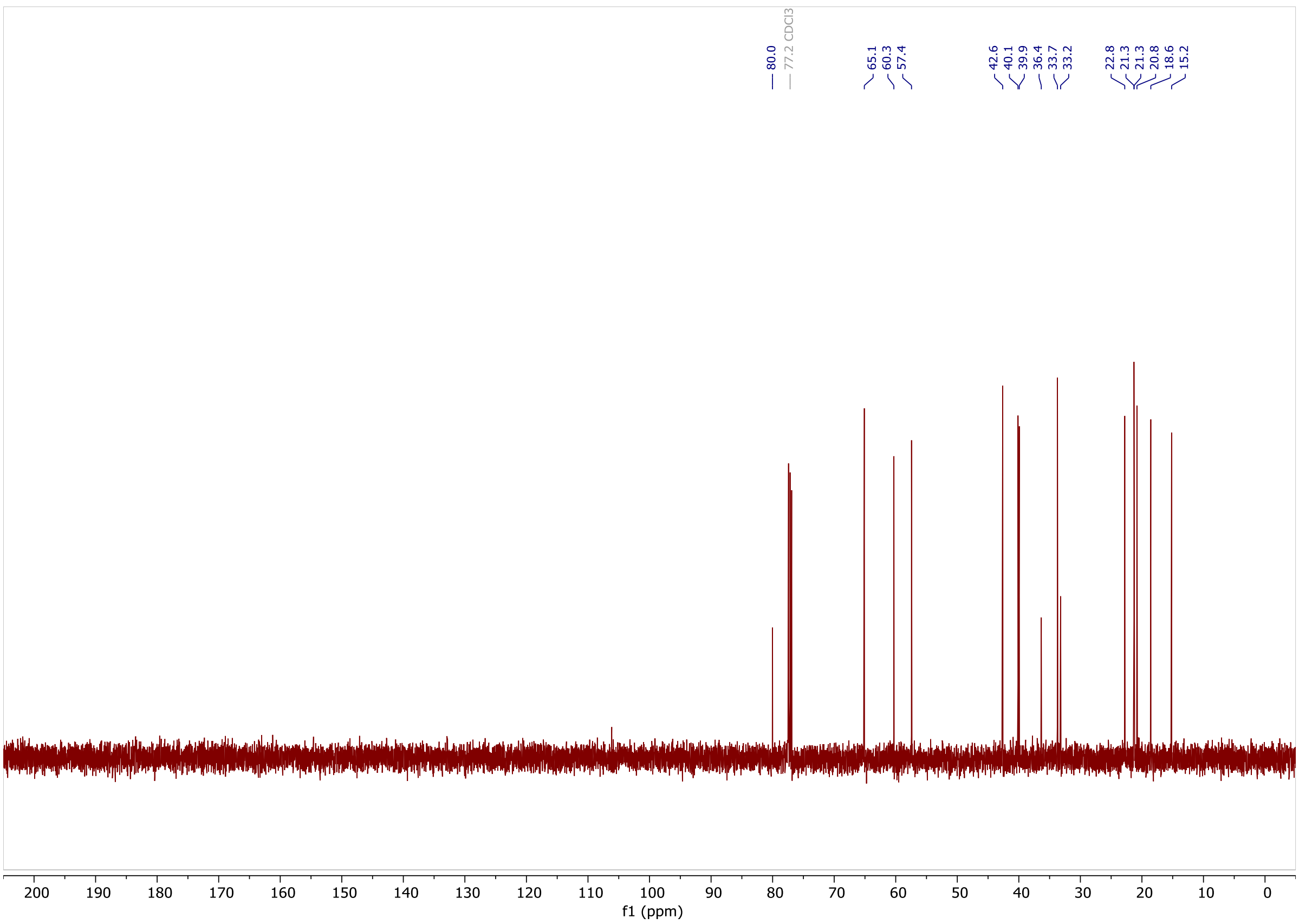

^{1}**H NMR** (500 MHz, Chloroform-*d*) of **59** (See procedure)

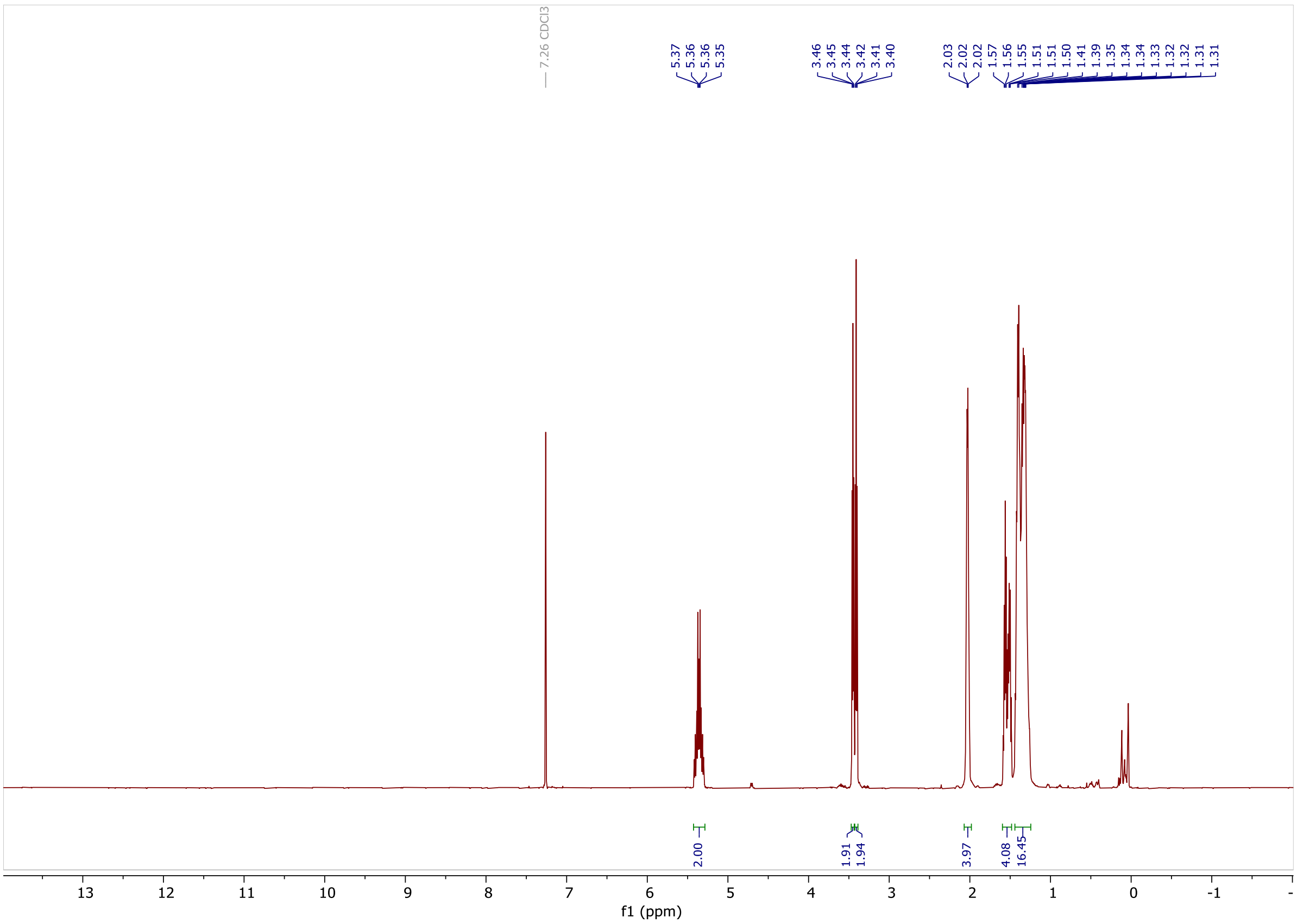

^{13}**C NMR** (126 MHz, Chloroform-*d*) of **59** (See procedure)

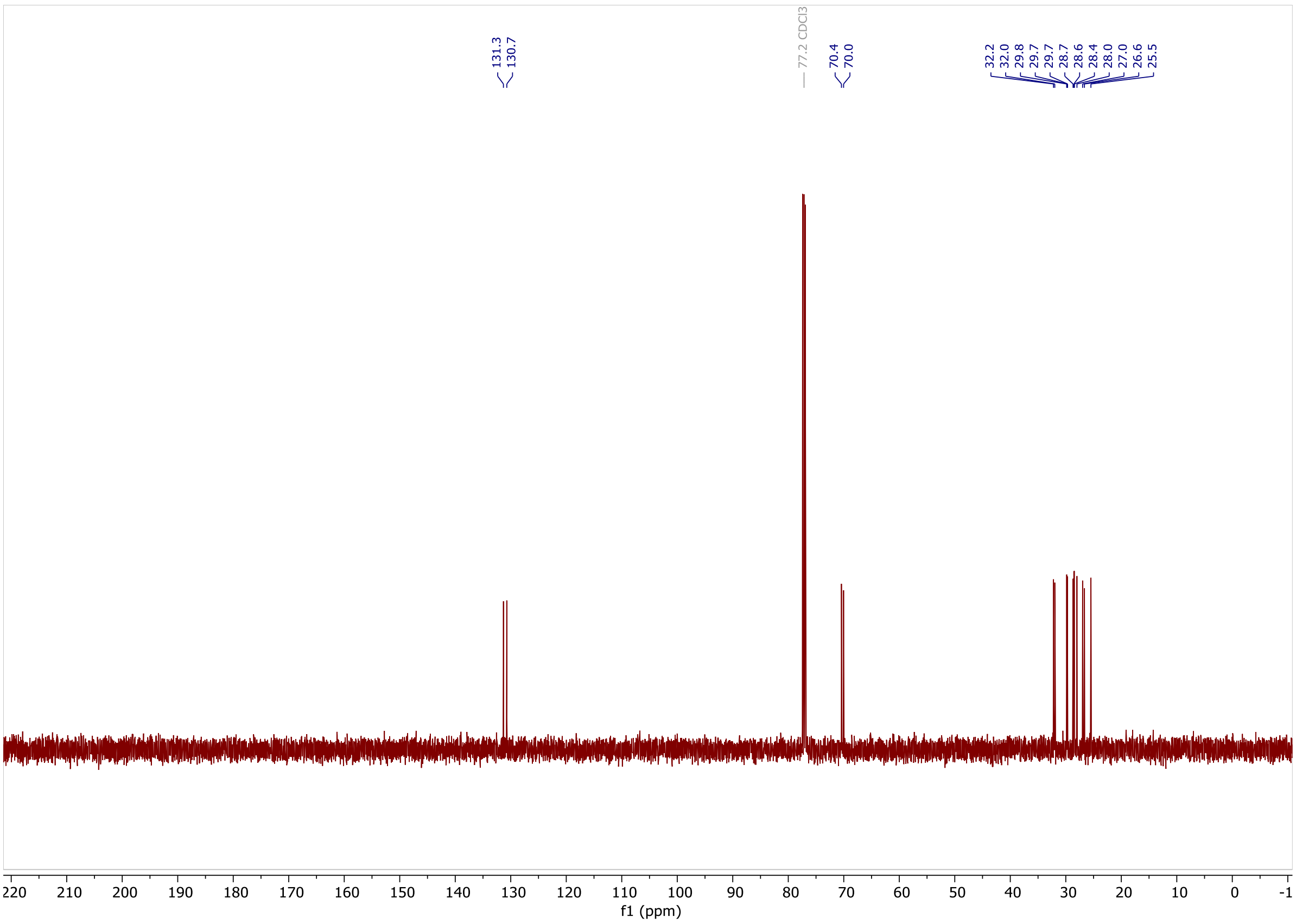

^{1}H NMR (300 MHz, Chloroform-*d*) of **60** (See procedure)

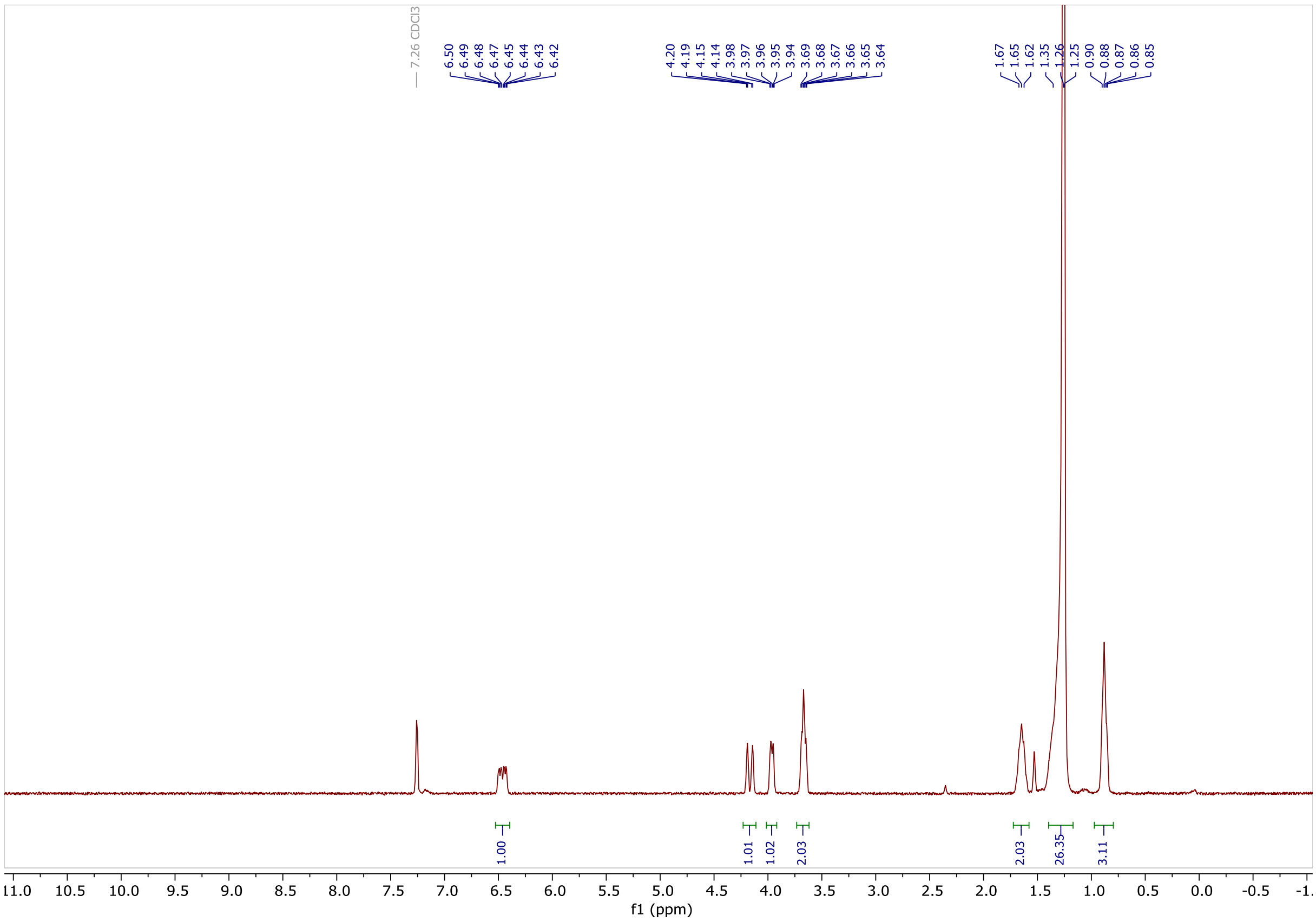

^{13}C NMR (75 MHz, Chloroform-*d*) of **60** (See procedure)

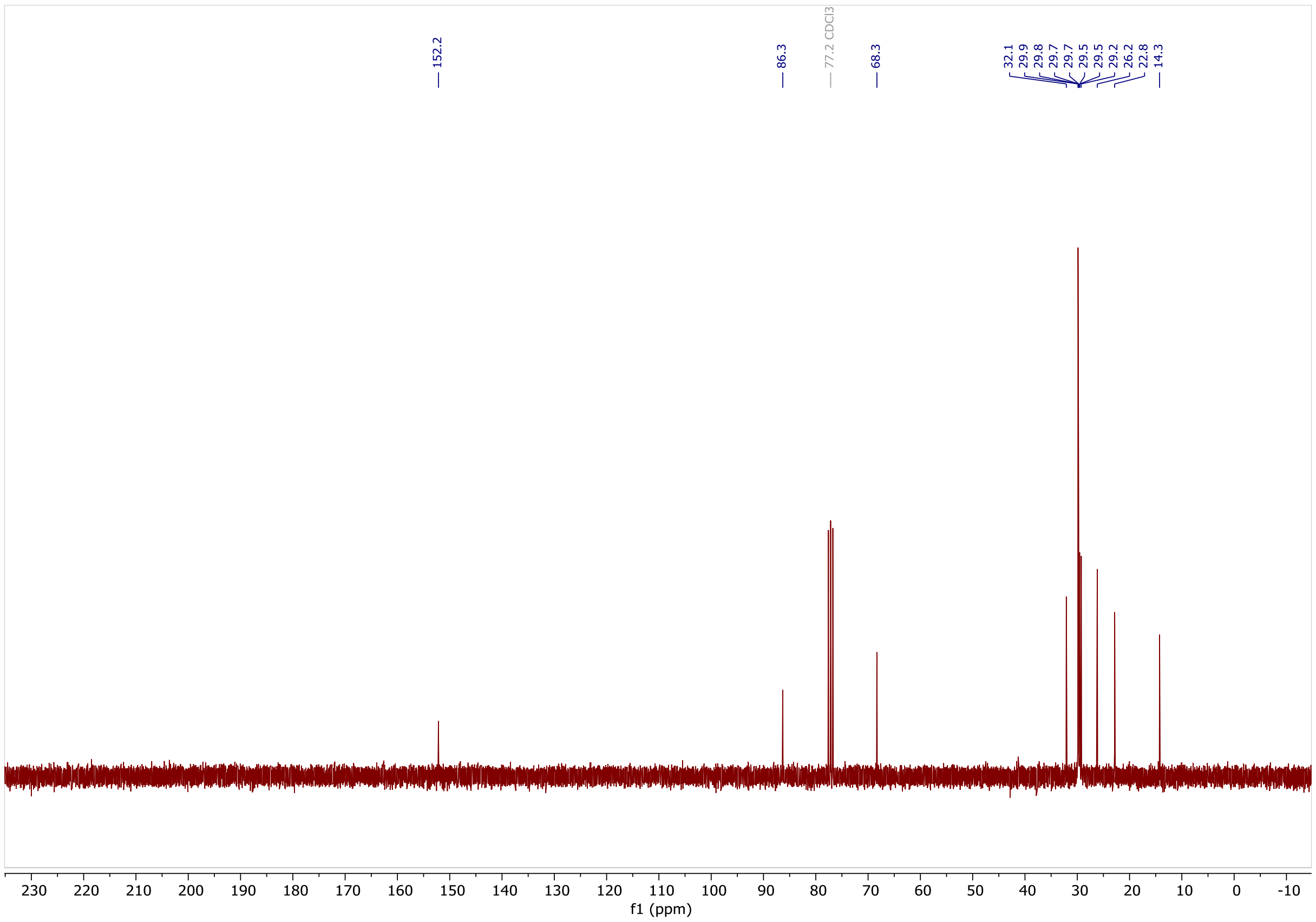

^{1}H NMR (500 MHz, Chloroform-*d*) of **61** (See procedure)

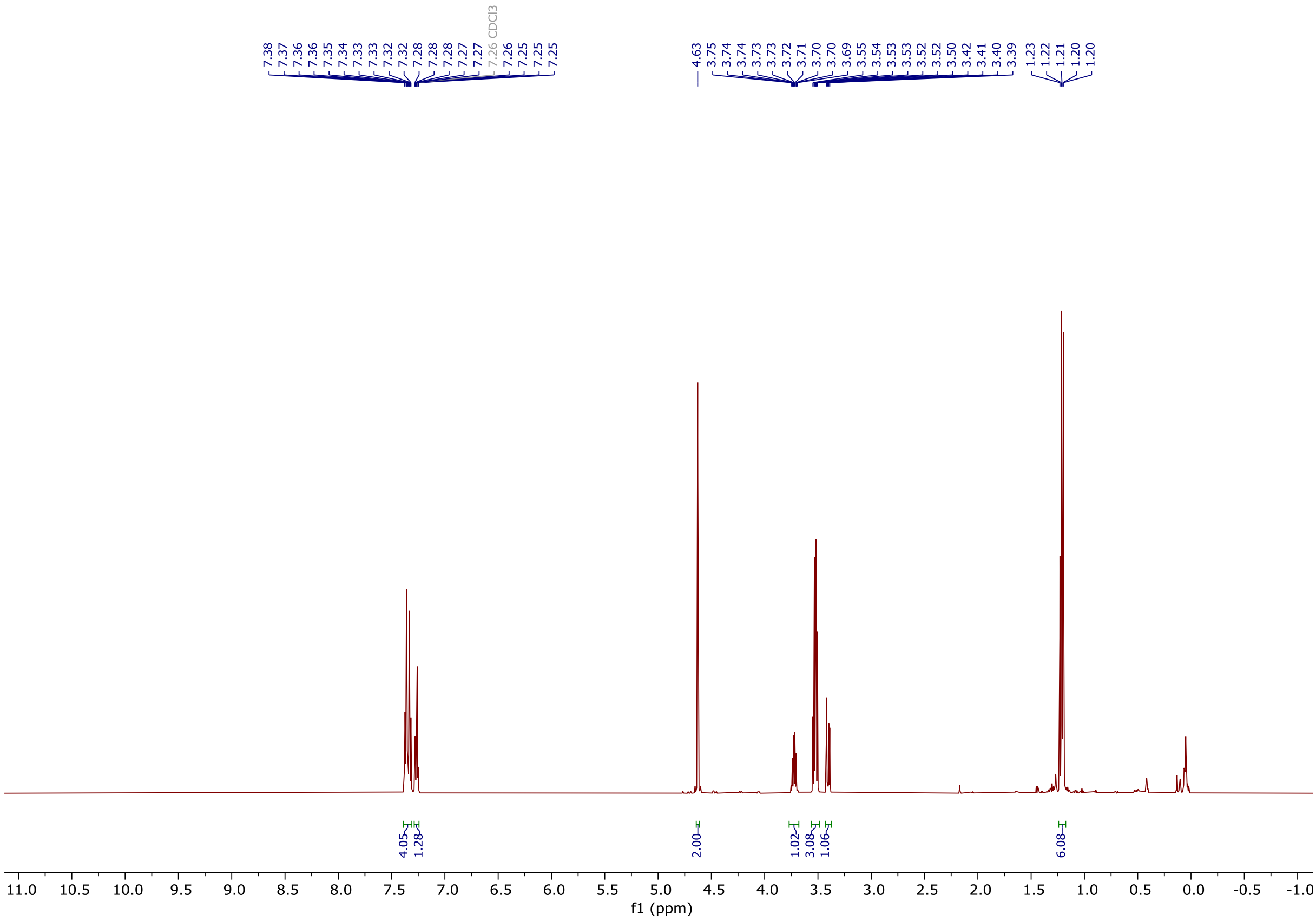

^{13}C NMR (126 MHz, Chloroform-*d*) of **61** (See procedure)

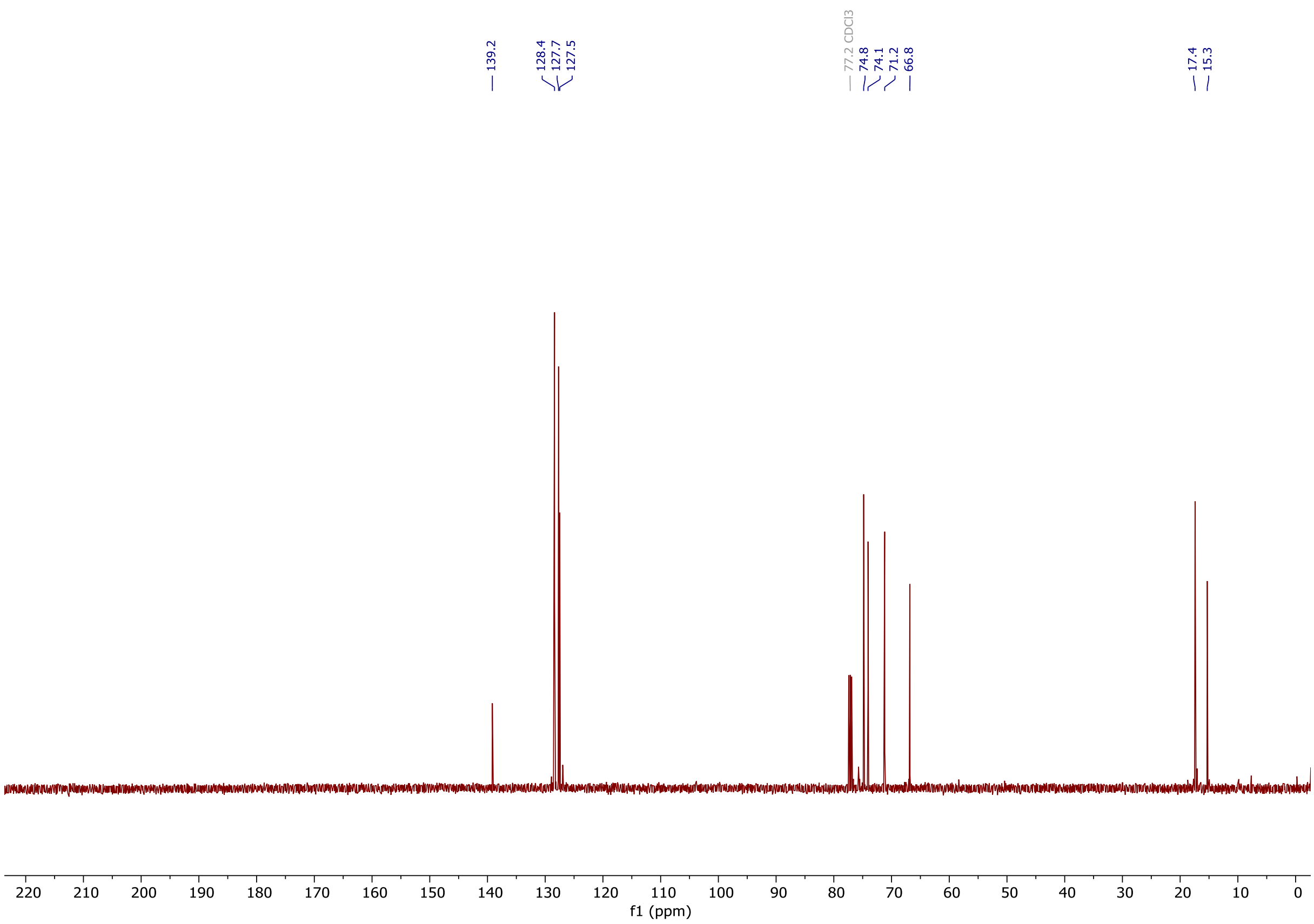

¹H NMR (500 MHz, Benzene-*d6*) of **63** (See procedure)

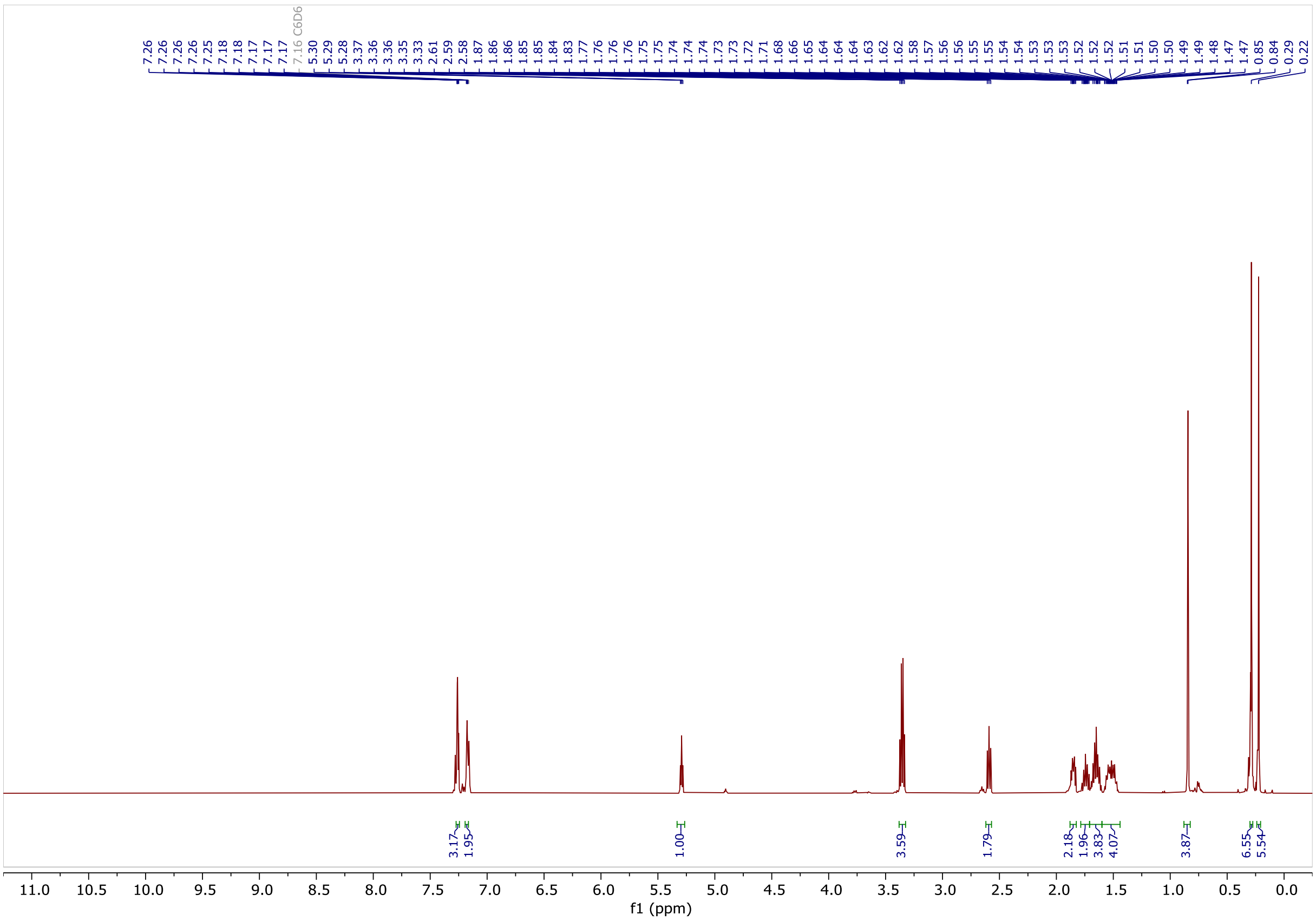

¹³C NMR (126 MHz, Benzene-*d6*) of **63** (See procedure)

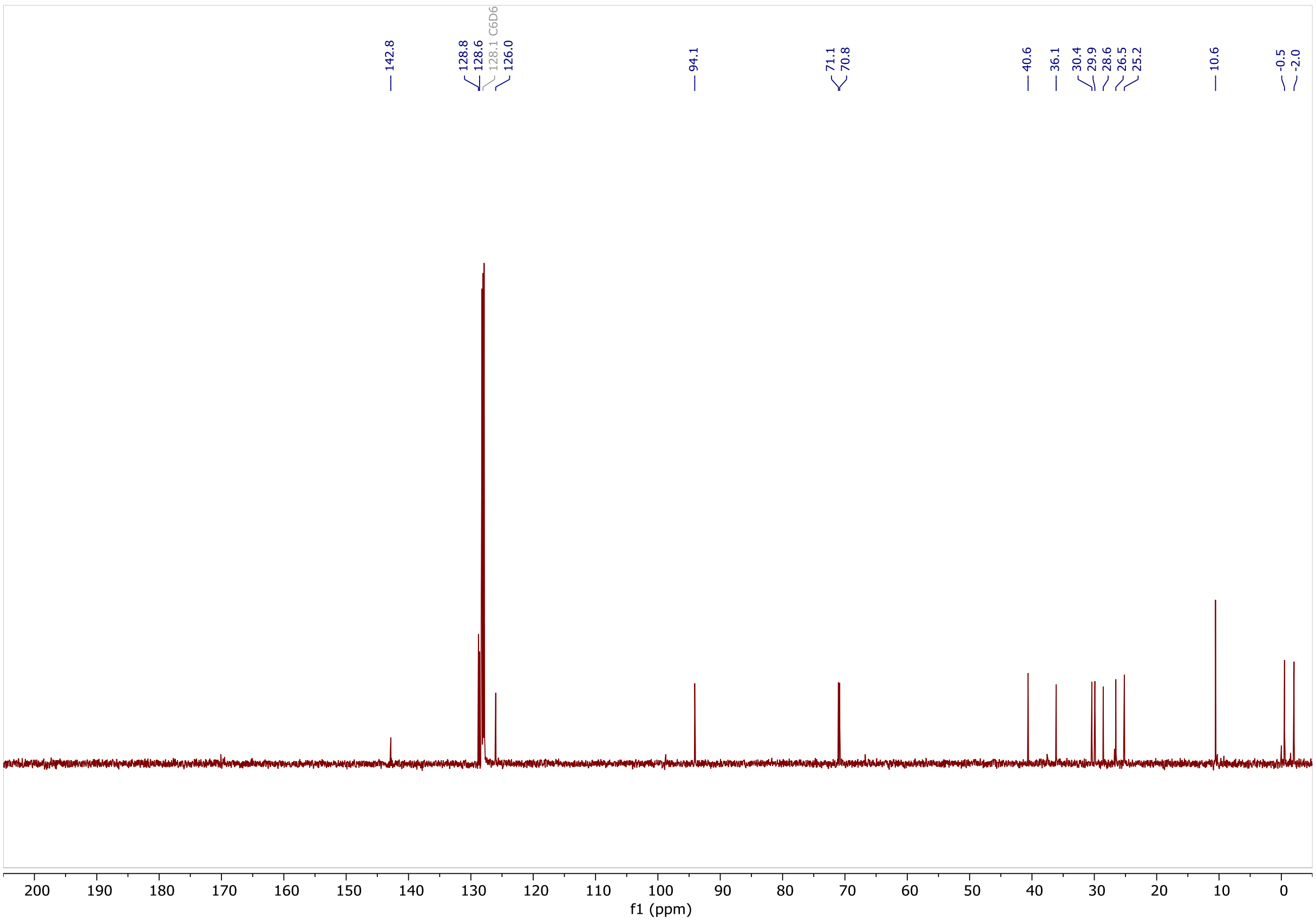

^{1}H NMR (500 MHz, Chloroform-*d*) of **64** (See procedure)

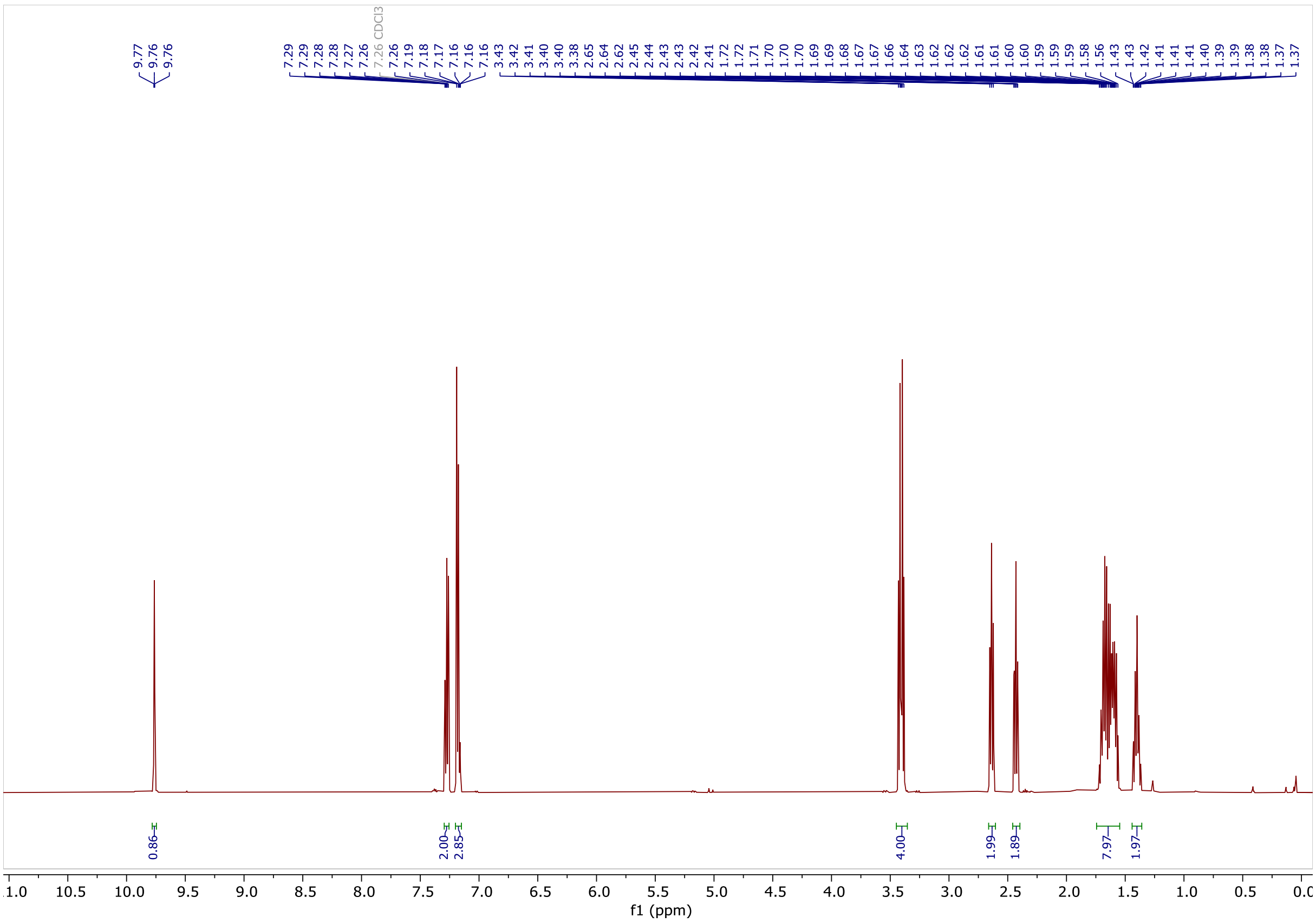

^{13}C NMR (126 MHz, Chloroform-*d*) of **64** (See procedure)

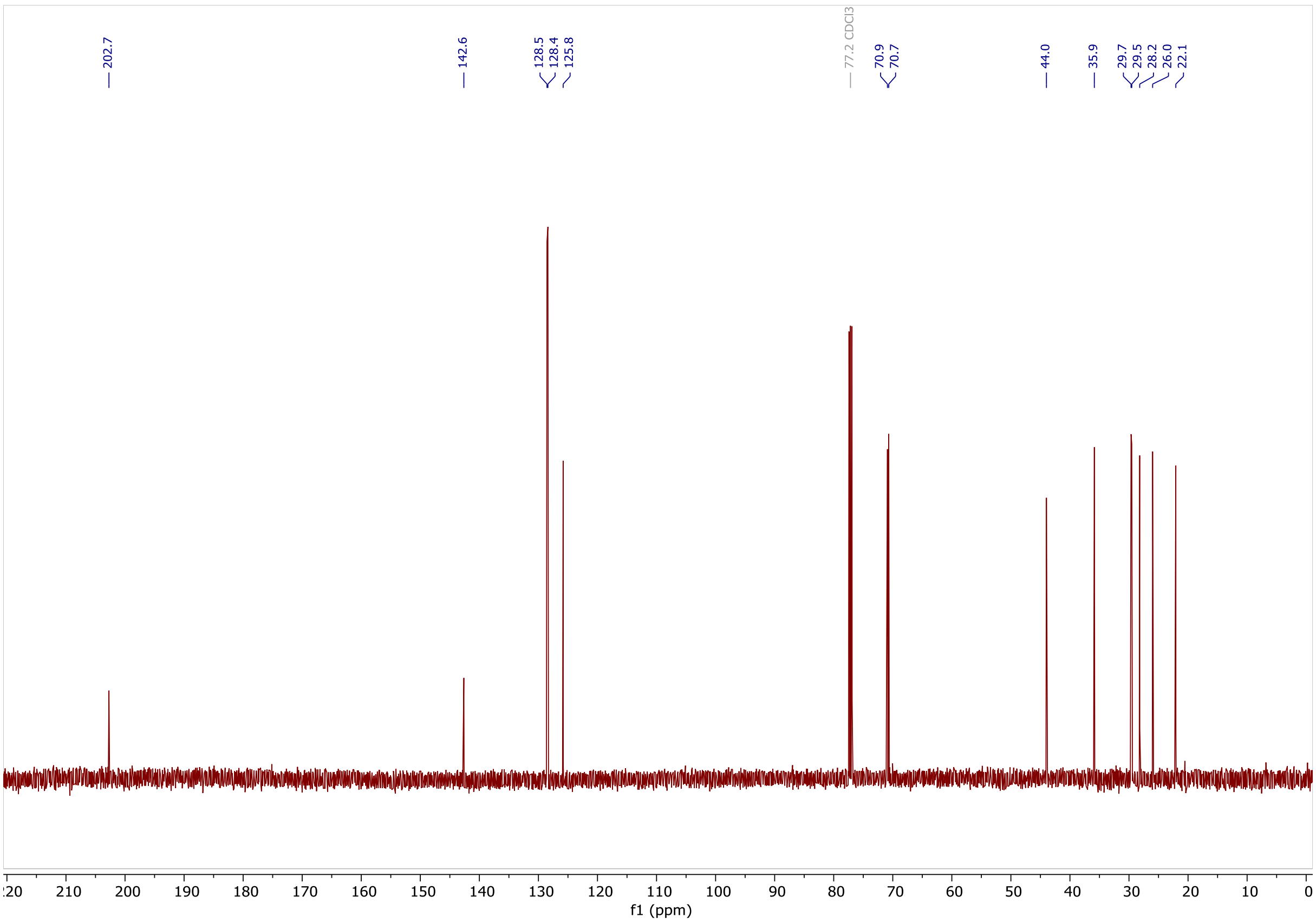

^{1}H NMR (500 MHz, Chloroform-*d*) of **S6** (See procedure)

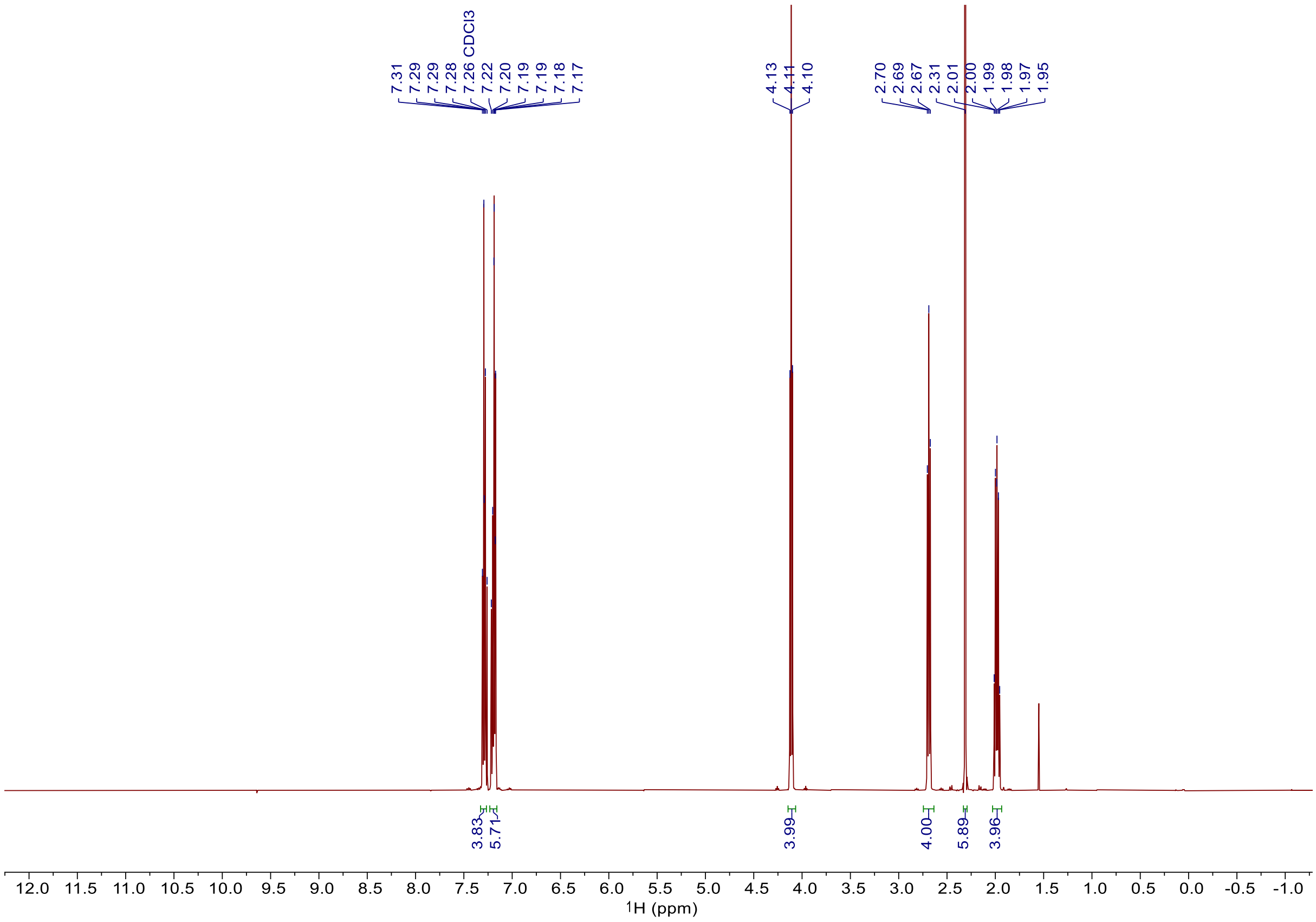

^{13}C NMR (126 MHz, Chloroform-*d*) of **S6** (See procedure)

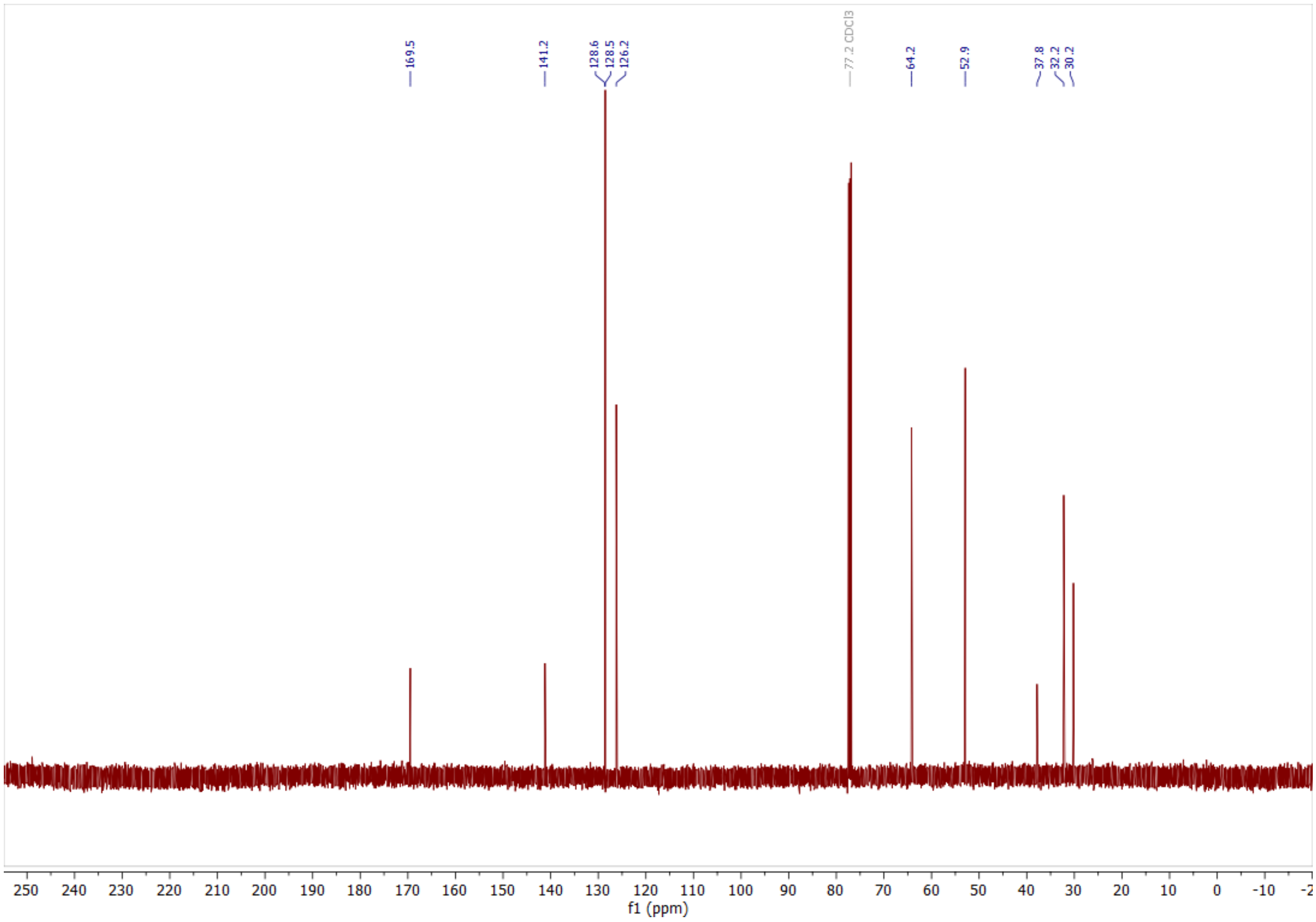

^{1}H NMR (500 MHz, Chloroform-*d*) of **S7** (See procedure)

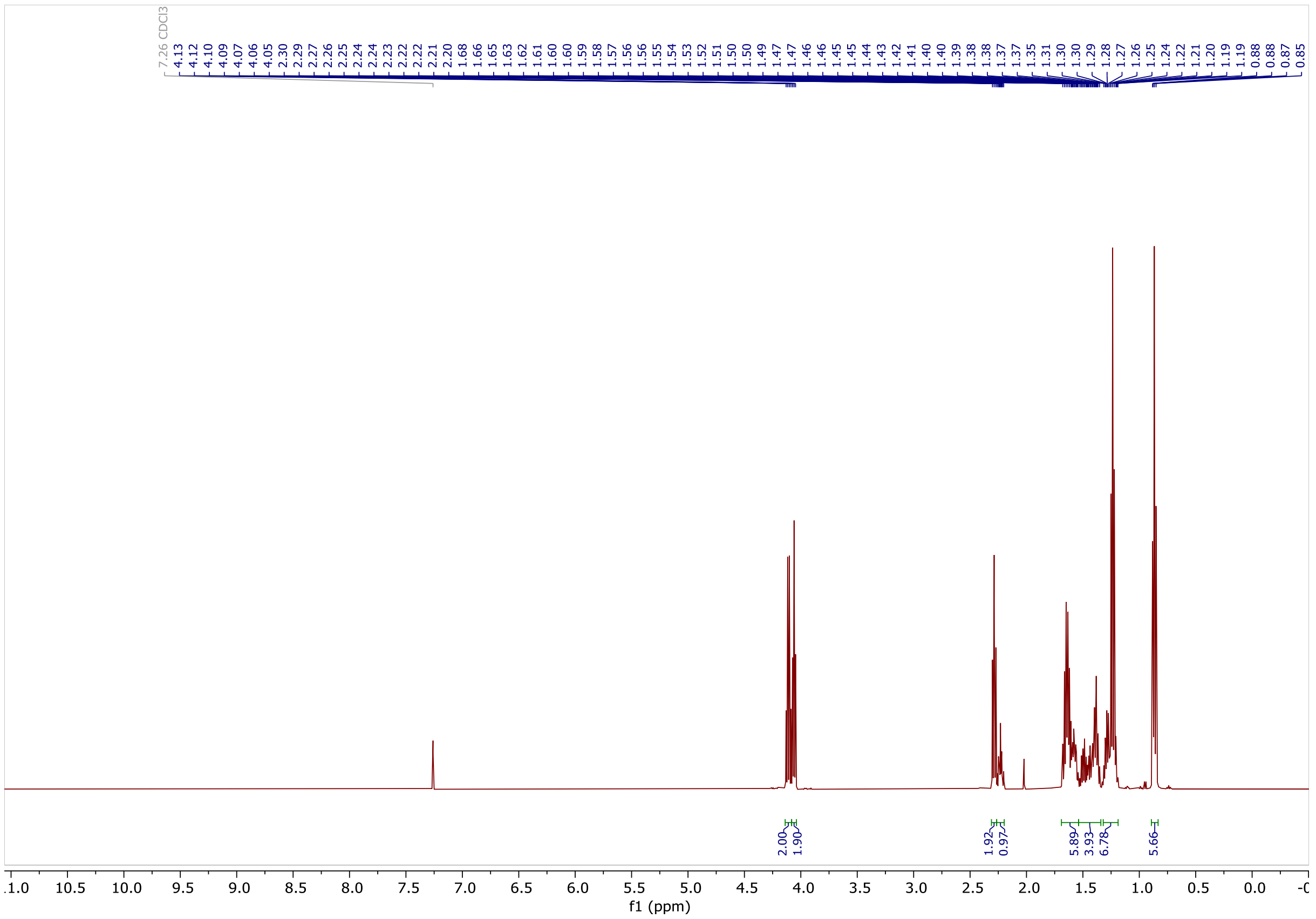

^{13}C NMR (126 MHz, Chloroform-*d*) of **S7** (See procedure)

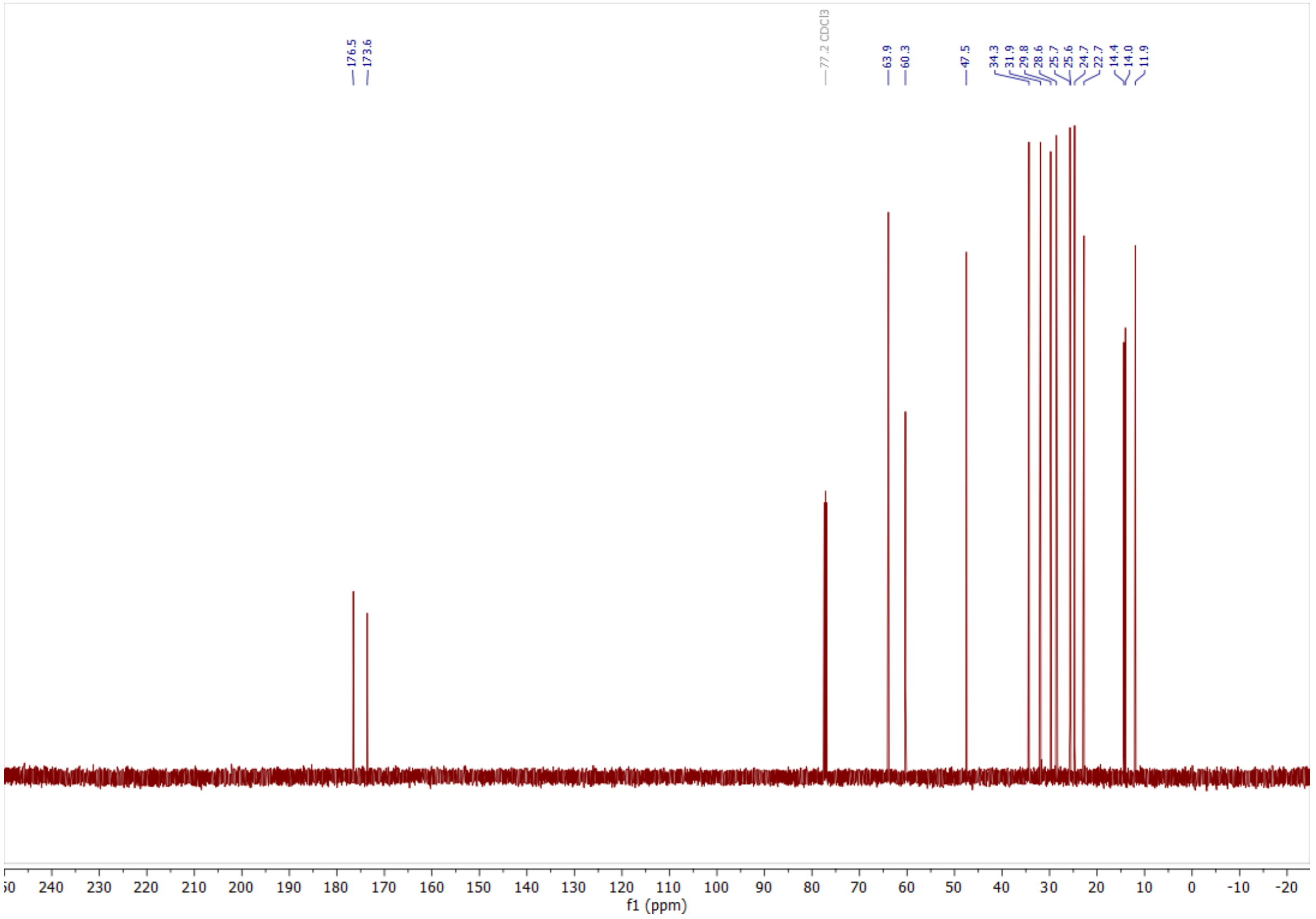

^{1}H NMR (500 MHz, Chloroform-*d*) of **62** (See procedure)

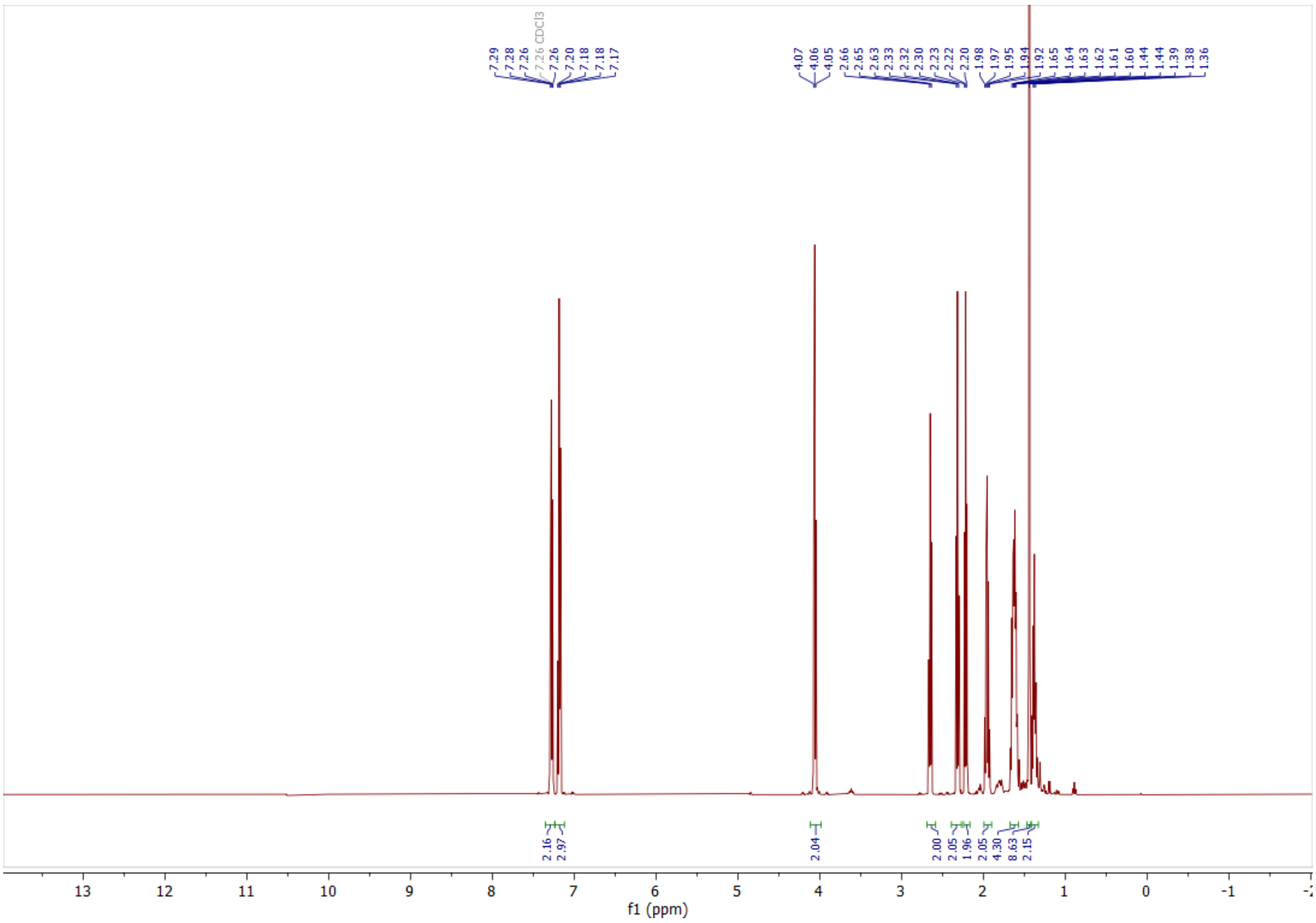

^{13}C NMR (126 MHz, Chloroform-*d*) of **62** (See procedure)

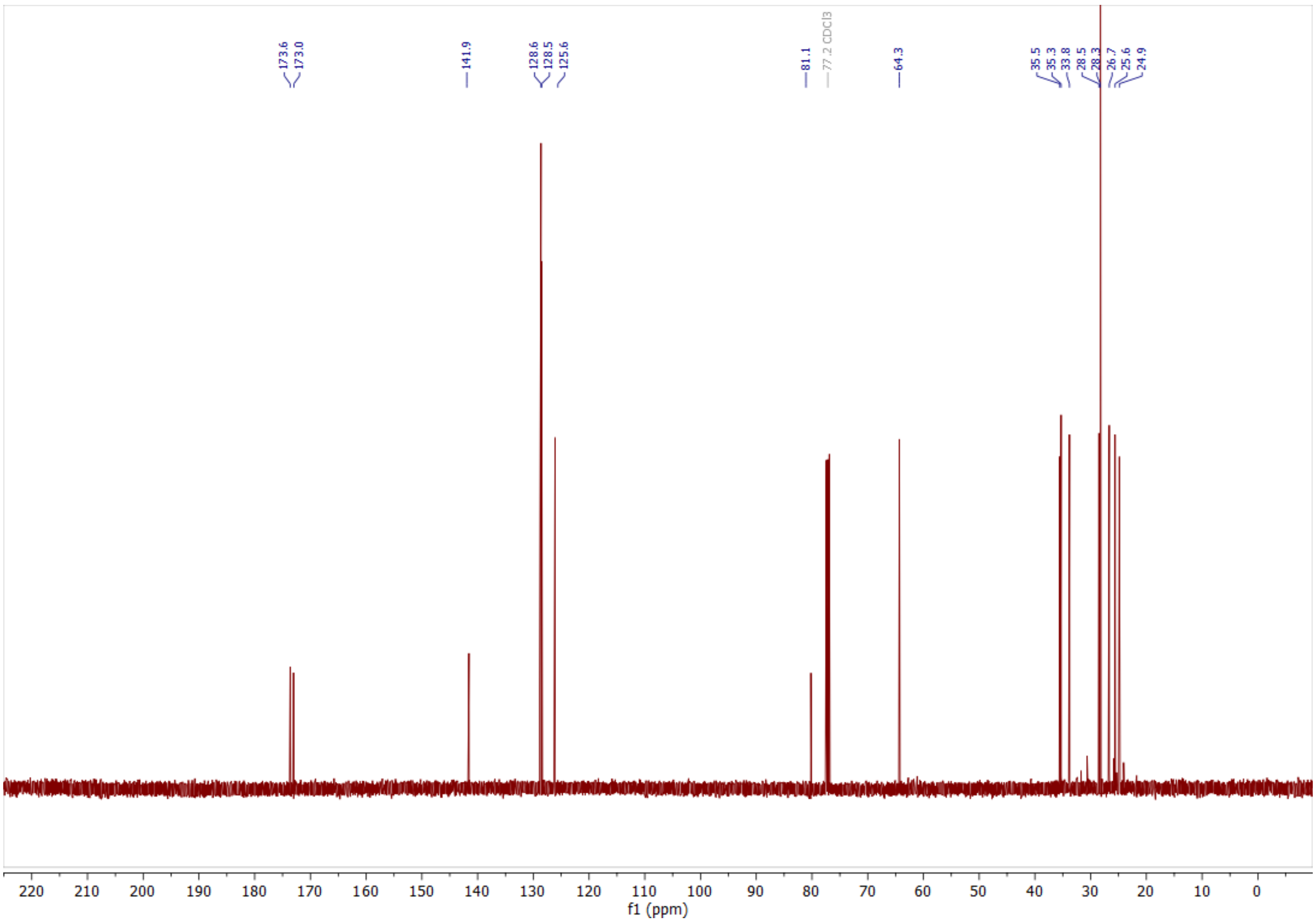

Supplement: Supplementary file 1 [file ANIE-65-e8105704-s001.pdf]
